# Extending the Structural Diversity of Labdane Diterpenoids from Marine-Derived Fungus *Talaromyces* sp. HDN151403 Using Heterologous Expression

**DOI:** 10.3390/md21120628

**Published:** 2023-12-03

**Authors:** Falei Zhang, Chuanteng Ma, Qian Che, Tianjiao Zhu, Guojian Zhang, Dehai Li

**Affiliations:** 1 Key Laboratory of Marine Drugs, Chinese Ministry of Education, School of Medicine and Pharmacy, Ocean University of China, Qingdao 266003, China; faleizhang@outlook.com (F.Z.); ma_chuanteng@163.com (C.M.); cheqian064@ouc.edu.cn (Q.C.); zhutj@ouc.edu.cn (T.Z.); zhangguojian@ouc.edu.cn (G.Z.); 2 Laboratory for Marine Drugs and Bioproducts, Laoshan Laboratory, Qingdao 266237, China; 3 Marine Biomedical Research Institute of Qingdao, Qingdao 266101, China; 4 Sanya Oceanographic Institute, Ocean University of China, Sanya 572025, China

**Keywords:** labdane diterpenoid, *Talaromyces* sp. HDN151403, heterologous expression, structural diversity, biosynthesis

## Abstract

Heterologous biosynthesis has become an effective means to activate fungal silent biosynthetic gene clusters (BGCs) and efficiently utilize fungal genetic resources. Herein, thirteen labdane diterpene derivatives, including five undescribed ones named talarobicins A–E (**3**–**7**), were discovered via heterologous expression of a silent BGC (*labd*) in *Aspergillus nidulans*. Their structures with absolute configurations were elucidated using extensive MS and NMR spectroscopic methods, as well as electronic circular dichroism (ECD) calculations. These labdanes belong to four skeleton types, and talarobicin B (**4**) is the first 3,18-*dinor*-2,3:4,18-*diseco*-labdane diterpene with the cleavage of the C2–C3 bond in ring A and the decarboxylation at C-3 and C-18. Talarobicin B (**4**) represents the key intermediate in the biosynthesis of penioxalicin and compound **13**. The combinatorial heterologous expression and feeding experiments revealed that the cytochrome P450 enzymes LabdC, LabdE, and LabdF were responsible for catalyzing various chemical reactions, such as oxidation, decarboxylation, and methylation. All of the compounds are noncytotoxic, and compounds **2** and **8** displayed inhibitory effects against methicillin-resistant coagulase-negative staphylococci (MRCNS) and *Bacillus cereus*.

## 1. Introduction

Labdane-related diterpenes are widely distributed secondary metabolites discovered from fungi, insects, higher plants, and marine organisms [1], possessing high structural diversity and a broad spectrum of biological activities [2,3,4,5,6,7,8,9,10]. The basic core structure of labdane diterpenes is composed of a fused decalin system (C1–10) and a branched six-carbon side chain (C11–16, with C16 attached to C13) at C9, generally with additional four carbons (C17–20) on C8, C4 (with C18 and C19 being attached), and C10 of the decalin system, respectively [6,11]. Originating from geranylgeranyl diphosphate (GGPP), two classes of specific diterpene synthases (diTPSs) are involved in the biosynthesis of labdane diterpenes [12,13,14,15,16]: (1) mostly the class II diTPS, copalyl pyrophosphate synthase (CPS), catalyzing the cyclization of GGPP to produce a bicyclic diphosphate intermediate (formed labdane- [17], clerodane- [18] and halimane-related [19] diterpenoids by removing the phosphate group, Figure S1), and (2) class I diTPS, converting into the final diterpenoids (including kaurane-, abietane-, pimarane-, beyerane-, atisane-, canssane-, stemodane-, and manoyl oxide-types) [20].

The reported class II labdane diTPSs are dual functional enzymes composed of terpene cyclase and prenyltransferase domain, for example, PvCPS (GenBank: LC316181.1) and PfCPS (JGI protein ID: 403578), which were discovered from *Penicillium verruculosum* TPU1311 and *Penicillium fellutanum* ATCC 48694, respectively [15]. The C-terminal prenyltransferase domain of PvCPS catalyzes the tandem condensation of C5 dimethylallyl diphosphate (DMAPP) with three successive isopentenyl diphosphates (IPPs) to form geranylgeranyl diphosphate (GGPP), which is subsequently cyclized to form copalyl diphosphate (CPP) catalyzed by the N-terminal terpene cyclase domain (Figure S1) [15]. Although the mechanism of diTPSs are insightfully understood, the secondary metabolites of the biosynthetic gene clusters (BGCs) harboring diTPS, such as PvCPS/PfCPS, especially those with various tailoring enzymes, are still poorly explored.

In this study, an attractive gene cluster (named *labd*) containing PvCPS homologous protein and various tailoring enzymes was found in the Antarctic sponge-derived fungus *Talaromyces* sp. HDN151403 based on bioinformatic analysis. Due to no labdane-related diterpene detected in the wild-type, reconstructing the entire biosynthetic pathway in genetically treatable fungal hosts may address this issue. Genetically tractable filamentous fungi such as *Aspergillus nidulans* have compatible transcription, translation, post-translational modification, and secretion machineries for the expression of foreign fungal genes [21], and therefore, the heterologous expression of the *labd* cluster in *Aspergillus nidulans* A1145 was attempted, which led to the production of 13 labdane diterpene derivatives, including five undescribed ones (namely talarobicins A–E). Structurally, these diterpenoids could be classified into four types, 3,18-*dinor*-2,3:4,18-*diseco*-, 18-*nor*-4,18-*seco*- and 3-*nor*-2,3-*seco*-labdane, and classical bicyclic labdane diterpenoids. Talarobicin B (**4**) is the first 3,18-*dinor*-2,3:4,18-*diseco*-labdane diterpene and served as the key intermediate in generation of labdane diterpenes with ring A cleaved and rearranged. Based on biosynthetic pathway reconstruction and feeding experiments in *A. nidulans*, the biosynthetic pathway of these compounds was proposed, which involved P450-catalyzed multiple oxidation.

## 2. Results and Discussion

### 2.1. Prediction of the Labdane-Related Diterpenes BGC in Talaromyces sp. HDN151403

**Figure 1:**
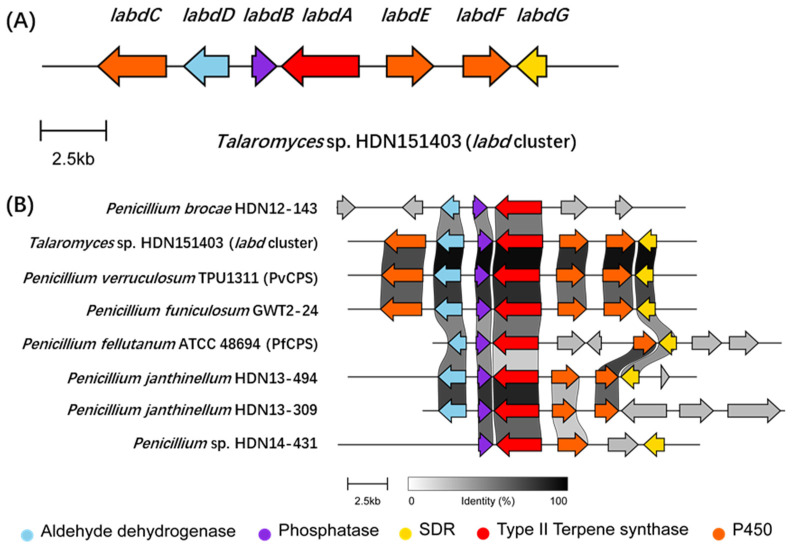
(**A**) The *labd* BGC cluster from *Talaromyces* sp. HDN151403. (**B**) Comparison of conserved gene clusters across different sequenced fungi were visualized via clinker [23]. Among them, the genome sequence of *P. verruculosum* TPU1311 (GenBank: GCA_001305275.1) was accessed from the NCBI database, and the genome sequence of *P. fellutanum* ATCC 48694 was accessed from the JGI Genome Portal [24].

By using the PvCPS gene (GenBank: LC316181.1) as a query to LocalBlast on our sequenced fungal genomes, a series of conservative homologous gene clusters were found in several fungi (Figure 1). The orthologous gene cluster was also conserved in *Talaromyces* and *Penicillium* fungi when using the remote search tool cblaster [22] to search the online database (Figures S2 and S3). These clusters contain two conserved genes encoding a phosphatase and a bifunctional terpene synthase, adjacent to various post-modification genes (Figure S3). Among them, a gene cluster (*labd*) from *Talaromyces* sp. HDN151403 is attractive for the abundant oxidoreductase. The *labd* gene cluster contains a bifunctional diterpene synthase LabdA, a phosphatase LabdB, a short-chain dehydrogenase/reductase (SDR) LabdG, an aldehyde dehydrogenase LabdD, two P450 monooxygenases LabdE and LabdF, and a dual domain P450-methyltransferase gene *labdC* (Figure 1, Table S3). Detailed bioinformatics analysis indicate that the diterpene synthase LabdA possesses both type II terpene cyclase and prenyltransferase domains with 89% identity to PvCPS and 59% identity to PfCPS. The sequence similarity search results indicate that the P450s LabdC, LabdE, and LabdF have high similarity with many sequences in the public database, but the functions are unknown (Figures S6–S8). Further phylogenetic analysis indicated that LabdE and LabdF may have the ability to catalyze multiple oxidation reactions (Figure S4), while the domain analysis of LabdC suggested that it may have both P450 and methyltransferase catalytic activities (Figure S5).

### 2.2. Heterologous Expression the labd BGC in A. nidulans A1145

**Figure 2:**
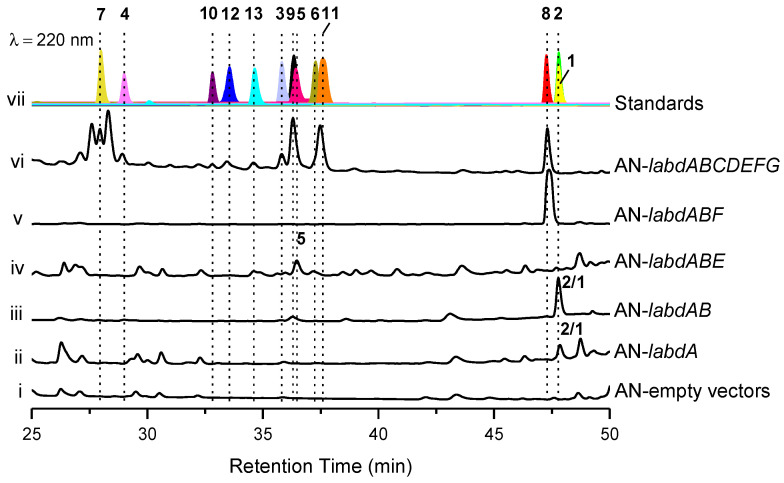
Expression of the *labd* cluster in *Aspergillus nidulans* (AN). HPLC traces of the EtOAc extracts derived from the *A. nidulans* wild-type and mutant strains (i−vi). The chromatograms were recorded at 220 nm in comparison with the separated and purified compounds (vii, separated compounds marked with numbers **1**–**13** and different colors).

**Figure 3:**
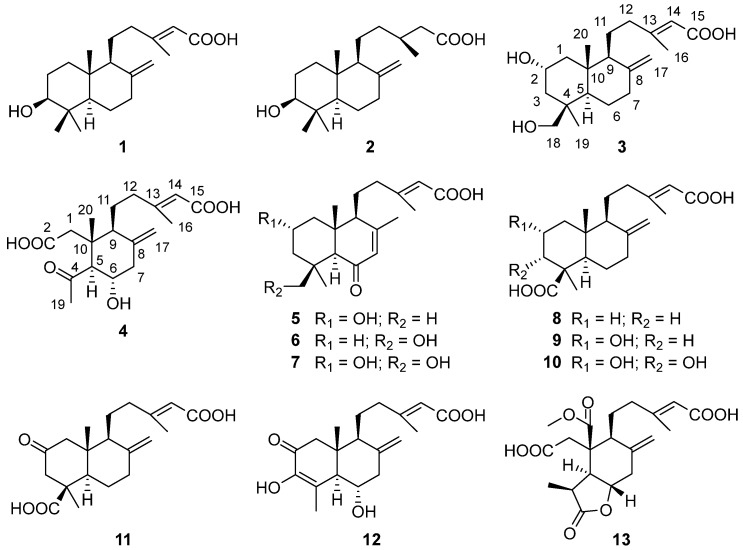
Chemical structures of **1**–**13**.

The reverse transcriptase PCR (RT-PCR) results show that the *labd* gene cluster was not expressed under laboratory culture conditions (Figure S9). To activate this cryptic gene cluster, we expressed the genes in the engineered *A. nidulans* A1145. The transformants of *A. nidulans* were grown on solid CD-ST medium for 5 days and extracted with ethyl acetate (EtOAc). Two compounds (**1** and **2**) were obtained from the core genes transformant AN-*labdAB*, and eleven compounds (**3**–**13**), including five undescribed ones, talarobicins A–E (**3**–**7**), were obtained from the whole genes transformant AN-*labdABCDEFG* (Figure 2 and Figure 3, Figure S10).

Compound **3** was obtained as a colorless powder. The molecular formula was determined to be C_20_H_32_O_4_ (five degrees of unsaturation) on the basis of the cationic molecular ion peak at *m/z* 354.2648 [M + NH_4_]^+^ in the high-resolution electrospray ionization mass spectrometry (HRESIMS) (Figure S18). The ^1^H NMR spectrum of **3** (Table 1, Figure S12) displayed three olefinic protons (*δ*_H_ 5.62, d, *J* = 1.2 Hz; *δ*_H_ 4.90, s; *δ*_H_ 4.57, s), two oxygenated methylene protons (*δ*_H_ 3.61, d, *J* = 11.5 Hz; *δ*_H_ 3.29, d, *J* = 11.5 Hz), one oxygenated methine proton (*δ*_H_ 3.81, m), and three methyl groups (*δ*_H_ 2.14, d, *J* = 1.2 Hz; *δ*_H_ 1.01, s; *δ*_H_ 0.74, s). The ^13^C and heteronuclear single quantum coherence (HSQC) NMR experiments showed 20 carbon resonances, including five non-protonated carbons (one carbonyl at *δ*_C_ 170.2, two olefinic carbons at *δ*_C_ 148.7 and 161.9, and two sp3 carbons at *δ*_C_ 41.5 and 41.8), eight methylenes (one olefinic carbon at *δ*_C_ 107.8, one oxygenated carbon at *δ*_C_ 65.5, and six sp3 methylene carbons at *δ*_C_ 23.0, 25.2, 39.5, 40.7, 45.0, and 49.0), four methines (one olefinic carbon at *δ*_C_ 116.8, one oxygenated carbon at *δ*_C_ 65.5, and two sp3 carbons at *δ*_C_ 56.9 and 57.4), and three methyls (*δ*_C_ 16.7, 18.9, 28.1) (Table 1, Figures S13 and S14).

**Figure 4:**
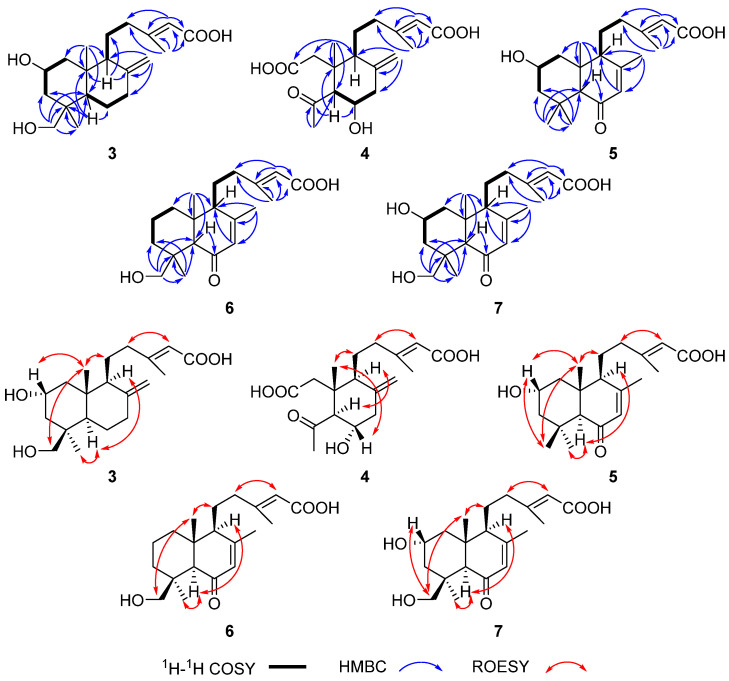
Key HMBC, ^1^H-^1^H COSY and ROESY correlations of **3**–**7**.

The planar structure of **3** was determined via detailed analysis of its two-dimensional (2D) NMR data (Figures S14–S17). Firstly, the ^1^H-^1^H correlation spectroscopy (COSY) spectrum revealed three partial structures, as shown in Figure 4, on the basis of the correlations between H_2_-1 and H-2, H-2 and H_2_-3, H_2_-6 and H_2_-7, H-9 and H_2_-11, and H_2_-11 and H_2_-12. Further analysis of the heteronuclear multiple-bond connectivity (HMBC) spectrum key correlations from H-5 to C-4/C-6/C-10, from H_2_-18 and Me-19 to C-3/C-4/C-5, from H-9 to C-8/C-10/C-11, from Me-20 to C-1/C-5/C-9/C-10, from H_2_-17 to C-7/C-8/C-9, and from Me-16 to C-12/C-13/C-14/C-15, along with H-14 to C-12/C-13/C-15, revealed the formation of a labdane-related diterpene skeleton deriving from (+)-CPP [15]. The chemical shifts of the carbonyl carbon at C-15 (*δ*_C_ 170.2) and the two oxygenated carbons, including C-2 (*δ*_C_ 65.5) and C-18 (*δ*_C_ 65.5), located the corresponding positions of the carboxyl group and the remaining two hydroxy groups, respectively.

The relative configuration of **3** was deduced via the rotating frame Overhauser effect spectroscopy (ROESY) experiment (Figure 4 and Figure S17). The ROESY correlations of H_2_-18/Me-20/H_2_-11 and the ROESY correlation between Me-20 and H-2 indicated that H_2_-18, Me-20, H_2_-11, and H-2 were at the same side (β-orientation). The ROESY correlations of Me-19/H-5/H-9 suggested that Me-19, H-5, and H-9 were on the same side (α-orientation). The double bond between C-13 and C-14 was assigned *E*-geometry as a result of the ROESY correlation between H-14 and H_2_-12. 

As for the absolute configuration of **3**, ECD calculations of the enantiomers (2R*,4S*,5R*,9S*,10R*)-**3** and (2S*,4R*,5S*,9R*,10S*)-**3** were carried out using B3LYP/6-31G(d)-GD3BJ optimized geometries at the B3LYP/6-311+g(d,p) level in methanol. The experimental ECD spectrum of **3** showed an ECD curve with a positive Cotton effect at 223 nm (Figure S11). The calculated ECD spectrum for (2R*,4S*,5R*,9S*,10R*)-**3** showed a similar ECD curve with Cotton effects at 225 (+) nm and 259 (−) nm, allowing the assignment of the 2*R*,4*S*,5*R*,9*S*,10*R* configuration for **3** (Figure S11). 

Compound **4** was isolated as a colorless powder. HRESIMS analysis established the molecular formula of **4** to be C_18_H_26_O_6_, indicating six degrees of unsaturation (Figure S26). The ^1^H NMR spectrum of **4** displayed three olefinic protons (*δ*_H_ 5.64, d, *J* = 1.5 Hz; *δ*_H_ 5.08, s; *δ*_H_ 4.75, s), one oxygenated methine proton (*δ*_H_ 3.85, td, *J* = 11.0, 5.0 Hz), and three methyl groups (*δ*_H_ 2.32, s; *δ*_H_ 2.15, d, *J* = 1.5 Hz; *δ*_H_ 0.81, s) (Table 1, Figure S20). Based on the analysis of ^13^C NMR and HSQC spectra (Table 1, Figures S21 and S22), 18 carbons could be designated as three methyls, five methylenes (one olefinic carbon at *δ*_C_ 111.0 and four sp3 methylene carbons at *δ*_C_ 23.8, 40.7, 42.9, and 47.6), four methines (one olefinic carbon at *δ*_C_ 116.8, one oxygenated carbon at *δ*_C_ 71.5, and two sp3 carbons at *δ*_C_ 49.5 and 63.6), and six non-protonated carbons (three carbonyls at *δ*_C_ 215.1, 174.3, and 170.3, two olefinic carbons at *δ*_C_ 145.3 and 161.8, and one sp3 carbons at *δ*_C_ 42.0), respectively.

The planar structure of **4** was determined via careful analysis of its 2D NMR data (Figures S22–S25). The ^1^H-^1^H COSY spectrum revealed two partial structures, as shown in Figure 4. The key HMBC correlations from H-5 to C-4/C-6/C-10, from Me-19 to C-4/C-5, from Me-20 to C-1/C-2/C-5/C-9/C-10, and from Me-16 to C-12/C-13/C-14/C-15, along with from H-14 to C-12/C-13/C-15, revealed the formation of a ring-opening labdane-related diterpene skeleton. The relative configuration of **4** was deduced via the ROESY experiment (Figure 4 and Figure S25). Specifically, the ROESY correlations between Me-20 and H-6, Me-20 and H_2_-11, and H-5 and H-9 indicated that Me-20, H_2_-11, and H-6 were at the same side (β-orientation) while H-5 and H-9 were on the other side (α-orientation). The ROESY correlation between H-14 and H_2_-12 indicated that the double bond between C-13 and C-14 was *E*-geometry. Finally, the calculated ECD spectrum for (5S*,6S*,9S*,10R*)-**4** showed a similar ECD curve with that of **4**. Thus, the absolute configuration of **4** was determined as 5*S*,6*S*,9*S*,10*R* (Figure S11).

The molecular formulas of compounds **5**–**7** were determined to be C_20_H_30_O_4_, C_20_H_30_O_4_, and C_20_H_30_O_5_ on the basis of HRESIMS data (Figures S34, S42, and S50), respectively. The similarity of the NMR data of **5**–**7** and callilongisin D [25] suggested that these compounds were close analogues. The ^1^H-^1^H COSY correlations of H-1/H-2/H-3, the HMBC correlations from Me-18 and Me-19 to C-3/C-4/C-5, and the HMBC correlations from Me-20 to C-1/C-5/C-9/C-10, along with the C-2 chemical shift of **5** (*δ* 64.6) (Table 2), which was downfield compared to that of callilongisin D (*δ* 18.3), indicated the presence of a -OH group substituted at C-2 in **5** (Figures S31 and S32). The shift value of C-18 (*δ* 63.7) in **6** was downfield compared to that of the same carbon in callilongisin D (*δ* 33.6), suggesting that a -OH group was substituted at C-18 in **6**, as evidenced by the HMBC correlation from H_2_-18 to C-3/C-4/C-5/C-19 (Figure S39). The C-2 chemical shift of **7** (*δ* 64.1) was downfield compared to that of callilongisin D (*δ* 18.3), and the shift value of C-18 (*δ* 64.3) was downfield compared to that of the same carbon in callilongisin D (*δ* 33.6), indicating that a -OH group was substituted at C-2 and C-18 of **7**, respectively. The ROESY correlations of Me-20/H-2, Me-20/Me-18, Me-18/H-2, Me-20/H_2_-11, Me-19/H-5, and H-5/H-9 in **5**, of Me-20/H_2_-18, Me-20/H_2_-11, Me-19/H-5, and H-5/H-9 in **6**, and of Me-20/H-2, Me-20/H_2_-18, H_2_-18/H-2, Me-20/H_2_-11, Me-19/H-5, and H-5/H-9 in **7** indicated that the stereostructure of **5**–**7** is highly similar (Figure 4, Figures S33, S41, and S49). The ROESY correlation between H-14 and H_2_-12 indicated that the double bond between C-13 and C-14 of **5**–**7** was *E*-geometry. Finally, the good agreement between the experimental and calculated ECD curves evidenced the absolute configuration of **5**–**7** as 2*S*,5*S*,9*S*,10*R*, 4*S*,5*R*,9*S*,10*R*, and 2*R*,4*S*,5*R*,9*S*,10*R*, respectively (Figure S11).

According to the comparison of NMR and MS data with those reported in the literature, the eight known compounds obtained in this study were identified as 3β-hydroxy-8(17),13(*E*)-labdadien-15-oic acid (**1**) [26], 3β-hydroxy-labd-8(17)-en-15-oic acid (**2**) [27], labda-8(17),13(*E*)-diene-15,19-dioic acid (**8**) [28], 2α-hydroxy-labda-8(17),13(*E*)-diene-15,19-dioic acid (**9**) [29], paecilomycine B (**10**) [29], penicichrysogene B (**11**) [30], penitholabene (**12**) [31], and 5-(4-carboxy-3-methyl-3-buten-1-yl)octahydro-4-(methoxycarbonyl)-3-methyl-6-methylene-2-oxo-4-benzofuranacetic acid (**13**) [32].

All of the isolated compounds were evaluated for their antimicrobial activity against methicillin-resistant coagulase-negative staphylococci (MRCNS), methicillin-resistant *Staphylococcus aureus* (MRSA), *Acinetobacter baumannii*, *Bacillus cereus*, *Pseudomonas aeruginosa*, *S. aureus*, and *Canidia albicans*. Compounds **2** and **8** displayed weak inhibitory activity against *B. cereus* (MIC 128 µg/mL) and MRCNS (MIC 128 µg/mL), respectively. Additionally, these compounds were not cytotoxic against the K562, ASPC-1, MDA-MB-231, H69AR, and L-02 cell lines, with inhibition lower than 50% at a concentration of 30 μM.

### 2.3. Heterologous Reconstitution of the Labdane Diterpenes Biosynthetic Pathway

**Figure 5:**
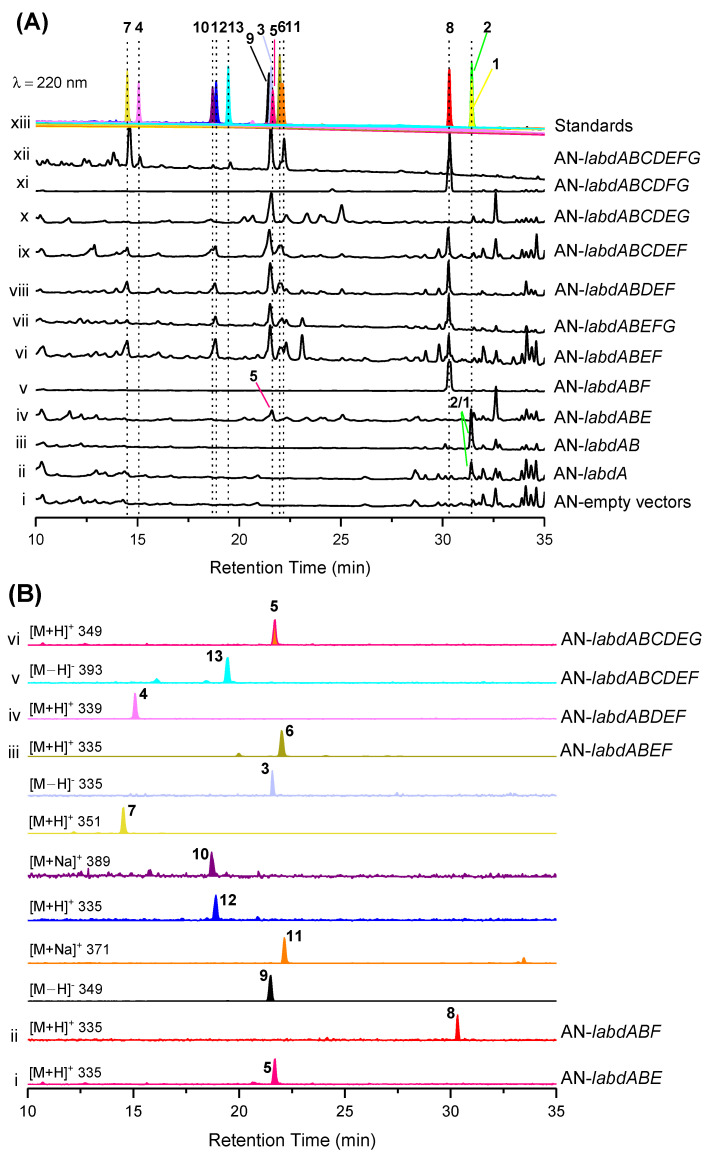
Combinatorial heterologous expression of the *labd* cluster in *A. nidulans* (AN). (**A**) LC-MS profiles of the EtOAc extracts derived from the *A. nidulans* transformants (i–xii) in comparison with the separated and purified compounds (xiii). (**B**) LC-MS traces of extracts from mutant strains (i–vi). The chromatograms were recorded at 220 nm on the Shimadzu UFLC system connected in series with the Shimadzu LCMS-2020 mass spectrometer. Separated compounds marked with numbers **1**–**13** and different colors.

To investigate the biosynthetic pathway of isolated compounds, we sought to reconstitute its biosynthesis in *A. nidulans*. Firstly, compounds **1** and **2** were produced from the transformant AN-*labdAB* (Figure 5A, trace iii). Structurally, **2** is the product of the reduction of the C13/C14 double bond of **1**, which is inferred to be catalyzed by the endogenous oxidoreductase of *A. nidulans*. Subsequently, **5** and **8** were produced from the two three-gene-expressing transformants AN-*labdABE* and AN-*labdABF*, respectively (Figure 5A, traces iv and v). Compounds **3** and **6**–**12** were produced by the four-gene-expressing transformants *labdABEF* (Figure 5B, trace iii). In addition, trace amounts of **4** and **13** were produced by strains AN-*labdABDEF* and AN-*labdABCDEF*, respectively (Figure 5B, traces iv and v). Only **5** was produced from the transformant strain AN-*labdABCDEG* (Figure 5B, trace vi), while **8** was extensively produced in the strain AN-*labdABCDFG* (Figure 5A, trace xi), indicating that the two genes *labdE* and *labdF* catalyze the most critical reaction in the biosynthesis pathway of other compounds.

**Figure 6:**
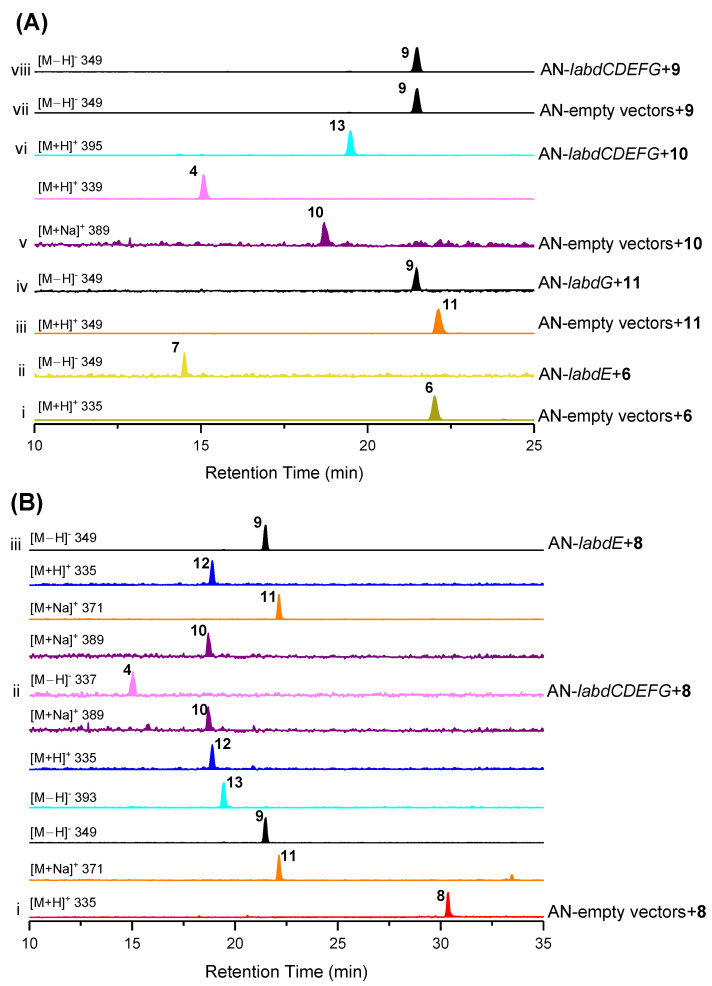
Dissecting the biosynthetic pathway of isolated compounds. (**A**,**B**) LC-MS traces of extracts from *A. nidulans* mutants after feeding with compounds **6**, **8**, **9**, **10**, and **11**. The chromatograms in all cases are extracted from mass spectra of the base peak for each compound. Separated compounds marked with numbers **4** and **6**–**13** and different colors.

Next, we aimed to reconstitute the complete biosynthetic pathway using *A. nidulans* feeding experiments. As shown in Figure 6A,B, LabdE can catalyze the conversion from **6** to **7** and **8** to **9**–**12**, respectively, indicating that LabdE is a multifunctional cytochrome P450 enzyme that catalyzes multiple oxidization. The strain AN-*labdG* fed **11** and produced **9**, indicating that LabdG can catalyze the reduction of the ketone group at C-2 in **11** (Figure 6A, traces iii and iv). To identify whether **4** and **13** are products of the *labd* gene cluster, strain AN-*labdCDEFG* was constructed and fed **8** and **9** separately. As a result, only **8** was successfully converted to **4** and **9**–**13** (Figure 6B, traces i and ii). Aside from that, **4** and **13** were also successfully produced from strain AN-*labdCDEFG* fed **10** (Figure 6A, traces v and vi), confirming that **10** was the direct precursor for the synthesis of **4** and **13**.

### 2.4. The Proposed Biosynthetic Pathways

**Scheme 1:**
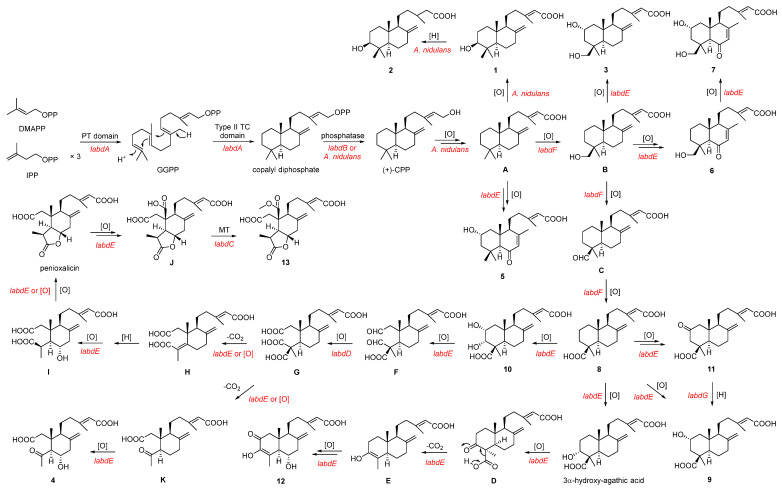
Proposed biosynthetic pathways of compounds **1**–**13**.

On the basis of the heterologous reconstitution of **1**–**13**, a plausible biogenetic pathway is proposed (Scheme 1). Firstly, the PT domain of LabdA catalyzes the condensation of DMAPP with three successively added IPPs to form GGPP, which is then cyclized to form copalyl diphosphate by the type II TC domain of LabdA [15]. After that, an endogenous phosphatase in *A. nidulans* or the phosphatase LabdB could convert copalyl diphosphate into (+)-CPP. The (+)-CPP is converted into compound **1** through intermediate **A** under the catalysis of endogenous oxidase in *A. nidulans*. Moreover, **1** can be converted into **2** under the catalysis of endogenous reductase in *A. nidulans*. Compound **5** is derived from the multi-step oxidation of intermediate **A** catalyzed by the P450 LabdE. The P450 LabdF can catalyze the hydroxylation of Me-18 in **A**, followed by oxidation to aldehydes and finally, dehydrogenates to **8**. During this cascade reaction, LabdE can catalyze the continued oxidation reaction of hydroxylation intermediate **B** to generate **3**, **6**, and **7**. 

On the one hand, compound **8** can be converted to **9**–**12** under the catalysis of LabdE. Compound **11** can also be reduced to **9** under the catalysis of the SDR LabdG. In the process of converting **8** to **12**, it is speculated that LabdE catalyzes multi-step reactions, including oxidation and decarboxylation. On the other hand, **10** undergoes a ring-opening reaction under the catalysis of LabdE to form aldehyde intermediate **F**, which is catalyzed by aldehyde dehydrogenase LabdD to form intermediate **G**. Since **G** is unstable, it will oxidize and decarboxylate spontaneously or under the catalysis of LabdE to produce **4**. The Intermediate **H** is catalyzed by LabdE to form **I**, which can further spontaneously or enzymatically form the compound penioxalicin. Bioinformatic analysis showed that the two P450s LabdE and LabdF share 39% and 20% identity to LabE, which is responsible for oxidizing the C-20 methyl group of raimonol in *Streptomyces* sp. KIB 015 to an aldehyde or carboxyl group, respectively [33]. Therefore, it is speculated that penioxalicin undergoes multi-step oxidation and methylation reactions under the catalysis of LabdE and LabdC, respectively, to generate **13**.

## 3. Materials and Methods

### 3.1. General Experimental Procedures

NMR spectra were recorded on a JEOLJN M-ECP 600 spectrometer (JEOL, Tokyo, Japan), an Agilent DD2-500 spectrometer, or a Bruker Avance NEO 400 MHz spectrometer (Bruker, Karlsruhe, Germany) using tetramethylsilane (TMS) as an internal standard. Specific rotations were obtained on a JASCO P-1020 digital polarimeter. UV-vis spectra were recorded on UFLC system (Shimadzu, Tokyo, Japan). HRESIMS spectra were obtained on a Thermo Scientific LTQ Orbitrap XL mass spectrometer (Thermo Fisher Scientific, Bremen, Germany) or Micromass Q-TOF ULTIMA GLOBAL GAA076 LC mass spectrometer (Waters Aisa Ltd., Singapore). A JASCO J-715 spectropolarimeter (JASCO, Tokyo, Japan) was used to obtain ECD spectra. LC−MS analyses were performed using a UFLC system (Shimadzu, Tokyo, Japan) with a C18 column (Shimadzu, 4.6 mm × 150 mm, 5 μm, 1 mL/min) coupled to a LCMS-2020 mass spectrometer (Shimadzu, Tokyo, Japan). Column chromatography (CC) was performed with silica gel (200–300 mesh, Qingdao Marine Chemical Ltd., Qingdao, China), SiliaSphere C18 (Octadecylsilyl, ODS) monomeric (SiliCycle Inc., Quebec, Canada, 50 μm, 120 A), and Sephadex LH-20 (GE Healthcare, Uppsala, Sweden). MPLC was performed using a C18 column (Welch Materials Inc., Ultimate^®^ XB-C18, 21.2 mm × 250 mm, 5 μm, 20 mL/min). Fractions were monitored via thin layer chromatography (TLC) (GF 254, Qingdao Haiyang Chemical Co., Ltd., Qingdao, China). The compounds were purified using a HPLC Hitachi 1110 system equipped with a 1430 PDA detector or HPLC system (PuriMaster-2000, Shanghai Kezhe Biochemical Technology Co., Ltd., Shanghai, China) and a C18 column (SilGreen, 10 mm × 250 mm, 5 μm, 3 mL/min).

### 3.2. Materials and Culture Conditions

The fungal strains used in this study are listed in Table S1. The fungus, *Talaromyces* sp. HDN151403, was isolated from an unidentified sponge sample collected from Prydz Bay, Antarctica (−624 m, 75.5° E, 69.7° S) and identified based on internal transcribed spacer DNA sequencing (GenBank: MW888514) [34]. *Talaromyces* sp. HDN151403 was cultured on potato dextrose agar (PDA, BD) at 28 °C for 5–7 days, and then the mycelia were transferred into potato dextrose broth (PDB, BD) for the isolation of genomic DNA, the isolation of total RNA, or the OSMAC analysis of secondary metabolites. *Escherichia coli* XL1-Blue was used for DNA manipulation. Heterologous expression was carried out with *Aspergillus nidulans* A1145 as the host. *Saccharomyces cerevisiae* BJ5464-NpgA was used for plasmid construction.

### 3.3. Gene Cloning, Plasmid Construction, and Genetic Manipulation

The plasmids utilized in this work are listed in Table S1. The oligonucleotide sequences for PCR primers are given in Table S2. The Q5^®^ High-fidelity DNA Polymerase and Restriction endonucleases used for all DNA manipulation were from New England Biolabs (NEB). The Frozen-EZ Yeast Transformation II Kit (Zymo Research) and the Zymoprep Yeast Plasmid Miniprep I Kit (Zymo Research) were used for transformation and plasmid recombination in yeast. 

Mycelia were collected from the liquid medium PDB via centrifugation. After grinding the mycelia in liquid nitrogen with a mortar, genomic DNA was extracted using cetyltrimethylammonium bromide (CTAB) buffer and phenol–chloroform extraction methods. All seven genes and their native terminators (300–500 bp downstream from the stop codon) were amplified via PCR using the gDNA of the original host as the template. Then, the recombinant plasmids were constructed according to the description from Yee and Tang [35]. The *glaA*, *gpdA*, and *amyB* promoters were amplified from vectors pANU, pANR, and pANP by using primer pairs *glaA*-F/*glaA*-R, *gpdA*-F/*gpdA*-R, and *amyB*-F/*amyB*-R, respectively. The *labd* genes were amplified via PCR using primer pairs as listed in Table S1 and then combined with the *Pac*I-linearized vector pANU, pANP, or pANR in vivo using yeast homologous recombination to yield several plasmids. The obtained yeast colonies were characterized using PCR. Yeast plasmids were isolated by using a Zymoprep Yeast Plasmid Miniprep I Kit and transformed into *E. coli* XL1 Blue. All the plasmids were confirmed via restriction enzyme digestion and DNA sequencing.

### 3.4. RNA Extraction and Reverse Transcriptase PCR (RT-PCR)

The RNA manipulations were carried out using the Direct-zol™ RNA Microprep Kit (Zymo Research) and the HiScript^®^ II 1st Strand cDNA Synthesis Kit (Vazyme Biotech Co., Ltd., Nanjing, China). The spores of *Talaromyces* sp. HDN151403 were inoculated into 100 mL of several media and cultivated at 28 °C for 14 days under dark conditions with two replicates each. Then, the mycelia were harvested and the total RNA was extracted using the Direct-zol™ RNA Microprep Kit according to the instructions. To evaluate the transcription of the *labd* gene cluster under different fermentation conditions, single-stranded cDNAs were synthesized using the HiScript^®^ II 1st Strand cDNA Synthesis Kit. The synthetic cDNA was used as a template to amplify gene fragments via PCR using primer pairs *labdA*-F/*labdA*-R, *labdB*-F/*labdB*-R, *labdC*-F/*labdC*-R, *labdD*-F/*labdD*-R, *labdE*-F/*labdE*-R, *labdF*-F/*labdF*-R, and *labdG*-F/*labdG*-R. The target gene fragment synthesized via PCR using gDNA as a template was used as a control. Then, we checked whether the target gene was expressed via DNA sequencing. The PCR primer pairs are listed in Table S2.

### 3.5. Transformation of A. nidulans

*A. nidulans* A1145 was used as the recipient host. Fungal protoplast preparation and transformation were carried out as described by Yee and Tang [35]. PEG-mediated protoplast transformation was employed to construct the 12 *A. nidulans* transformant strains AN-*labdA*, AN-*labdE*, AN-*labdG*, AN-*labdAB*, AN-*labdABE*, AN-*labdABF*, AN-*labdABEF*, AN-*labdABDEF*, AN-*labdABEFG*, AN-*labdABCDEF*, AN-*labdABCDEG*, and AN-*labdABCDEFG*. Transformation with three empty pANU, pANR, and pANP vectors was used as the control (AN-empty vectors). Transformants were verified using PCR.

### 3.6. Fermentation and LC/LC−MS Analysis

*A. nidulans* transformants were cultivated on solid CD-ST medium (20 g/L starch, 10 g/L casein hydrolysate (acid), 50 mL/L nitrate salts, 1 mL/L trace elements, 20 g/L agar) at 28 °C for 5 days. Then, the cultures were extracted three times with ethyl acetate (EtOAc). The organic phase was evaporated to dryness and re-dissolved in 200 μL of MeOH/H_2_O (90:10). Then, 50 μL of dissolved extract was injected for high-performance liquid chromatography–photodiode array detection-mass spectrometry (HPLC-DAD-MS) analysis (C18 column, Shimadzu, 4.6 mm × 150 mm, 5 μm, 1 mL/min); the samples were separated on a linear gradient of 15–50% acetonitrile (CH_3_CN) in water (0.1% trifluoroacetic acid) for 25 min at a flow rate of 1 mL/min, followed by a linear gradient of 50–100% acetonitrile (CH_3_CN) in water (0.1% trifluoroacetic acid) for 10 min, and then followed by isocratic 100% CH_3_CN for 5 min. For LC analysis (C18 column, Shimadzu, 4.6 mm × 150 mm, 5 μm, 1 mL/min), samples were separated on a linear gradient of 5−50% acetonitrile (CH_3_CN) in water (0.1% trifluoroacetic acid) for 40 min at a flow rate of 1 mL/min, followed by a linear gradient of 50–100% acetonitrile (CH_3_CN) in water (0.1% trifluoroacetic acid) for 10 min, and then followed by isocratic 100% CH_3_CN for 5 min.

### 3.7. Extraction, Isolation, and Purification

The large-scale solid fermentation culture (CD-ST medium, 5 L) of the transformant strain AN-*labdAB* was extracted four times with EtOAc. The crude extract (9 g) was subjected to a C18 column using a stepped gradient elution of MeOH/H_2_O, yielding five subfractions (Fr.1–Fr.5, 30% to 70%). Fr.3 was purified on a preparative C18 HPLC column with a gradient of MeOH/H_2_O (72:28) to yield **2** (10.5 mg, retention time (*t*_R_) = 22 min). Fr.4 was purified on a preparative C18 HPLC column with a gradient of MeOH/H_2_O (75:25) to yield **1** (12.0 mg, *t*_R_ = 16.5 min).

The large-scale solid fermentation culture (CD-ST medium, 20 L) of the transformant strain AN-*labdABCDEFG* was extracted four times with EtOAc. The crude extract (20 g) was subjected to a C18 column using a stepped gradient elution of MeOH/H_2_O, yielding six subfractions (Fr.1–Fr.6, 30% to 80%). Fractions were monitored via TLC and LC–MS. Fr.2 was purified on a preparative C18 HPLC column with a gradient of MeOH/H_2_O (48:52) to yield **13** (4.0 mg, *t*_R_ = 19.0 min). Fr.3 was purified on a preparative C18 HPLC column with a linear gradient of MeOH/H_2_O (40:60-90:10) to yield two subfractions (Fr.3.1 and Fr.3.2). Then, Fr.3.1 was purified on a preparative C18 HPLC column with a gradient of MeOH/H_2_O (58:42) to yield **7** (22.5 mg, *t*_R_ = 11.9 min) and **4** (1.7 mg, *t*_R_ = 14.4 min). Fr.4 was purified on a preparative C18 HPLC column with a gradient of MeCN/H_2_O (30:70) to yield **12** (5.5 mg, *t*_R_ = 23.5 min), **10** (6.3 mg, *t*_R_ = 25 min), **5** (6.7 mg, *t*_R_ = 38 min), **6** (11.0 mg, *t*_R_ = 44.5 min), and **11** (45.4 mg, *t*_R_ = 47.5 min). Fr.5 was purified on a preparative C18 HPLC column with a gradient of MeCN/H_2_O (35:65) to yield **9** (64.8 mg, *t*_R_ = 25.0 min) and **3** (18.5 mg, *t*_R_ = 27.1 min). Fr.6 was purified on a preparative C18 HPLC column with a gradient of MeCN/H_2_O (50:50) to yield **8** (51.7 mg, *t*_R_ = 31.2 min).

Talarobicin A (**3**): colorless powder; ${\left[\alpha \right]}_{D}^{25}$ +24.24, (*c* 0.21, CH_3_OH); UV (CH_3_OH) *λ*_max_ (log *ε*) 223 (3.13) nm; ECD (1.25 × 10^−3^ M, MeOH) *λ*_max_ (∆*ε*) 223 (+22.24) nm; ^1^H NMR (500 MHz) and ^13^C NMR (125 MHz) data (CD_3_OD), see Table 1; positive ion HRESIMS *m/z* 354.2648 [M + NH_4_]^+^ (calcd. for C_20_H_36_O_4_N^+^, 354.2639).

Talarobicin B (**4**): colorless oil; ${\left[\alpha \right]}_{D}^{25}$ +9.90, (*c* 0.03, CH_3_OH); UV (CH_3_OH) *λ*_max_ (log *ε*) 225 (2.71) nm; ECD (1.14 × 10^−3^ M, MeOH) *λ*_max_ (∆*ε*) 219 (+8.28), 288 (−2.57) nm; ^1^H NMR (500 MHz) and ^13^C NMR (125 MHz) data (CD_3_OD), see Table 1; positive ion HRESIMS *m/z* 339.1793 [M + H]^+^ (calcd. for C_18_H_27_O_6_^+^, 339.1802).

Talarobicin C (**5**): colorless powder; ${\left[\alpha \right]}_{D}^{25}$ +0.90, (*c* 0.03, CH_3_OH); UV (CH_3_OH) *λ*_max_ (log *ε*) 232 (2.92) nm; ECD (1.32 × 10^−3^ M, MeOH) *λ*_max_ (∆*ε*) 221 (−1.99), 240 (−3.94), 329 (+0.91) nm; ^1^H NMR (500 MHz) and ^13^C NMR (125 MHz) data (CD_3_OD), see Table 2; positive ion HRESIMS *m/z* 335.2210 [M + H]^+^ (calcd. for C_20_H_31_O_4_^+^, 335.2217).

Talarobicin D (**6**): colorless powder; ${\left[\alpha \right]}_{D}^{25}$ +6.00, (*c* 0.07, CH_3_OH); UV (CH_3_OH) *λ*_max_ (log *ε*) 227 (3.48) nm; ECD (1.16 × 10^−3^ M, MeOH) *λ*_max_ (∆*ε*) 224 (−2.48), 243 (−6.67), 320 (+1.51) nm; ^1^H NMR (500 MHz) and ^13^C NMR (125 MHz) data (CD_3_OD), see Table 2; positive ion HRESIMS *m/z* 335.2209 [M + H]^+^ (calcd. for C_20_H_31_O_4_^+^, 335.2217).

Talarobicin E (**7**): yellowish oil; ${\left[\alpha \right]}_{D}^{25}$ +0.34 (*c* 0.29, CH_3_OH); UV (CH_3_OH) *λ*_max_ (log *ε*) 230 (3.80) nm; ECD (1.28 × 10^−3^ M, MeOH) *λ*_max_ (∆*ε*) 218 (−2.08), 242 (−8.42), 329 (+2.04) nm; ^1^H NMR (500 MHz) and ^13^C NMR (125 MHz) data (CD_3_OD), see Table 2; positive ion HRESIMS *m/z* 351.2156 [M + H]^+^ (calcd. for C_20_H_31_O_5_^+^, 351.2166).

### 3.8. A. nidulans Feeding Experiments

The *A. nidulans* transformant strain was grown on CD-ST liquid culture (50 mL) at 28 °C 180 rpm. After growth for 2 days, substrate (1–2 mg) dissolved in DMSO (50 μL) was added to the culture. After further incubation for 3 days, the mycelium was collected via filtration and extracted with acetone, while the media was extracted with ethyl acetate. The crude extracts were reduced to dryness in vacuo and re-dissolved in methanol for LC–MS analysis.

### 3.9. ECD Calculations

Conformation searches based on molecular mechanics with MMFF force fields were performed for stereoisomers to obtain stable conformers. All the stable conformers were further optimized using the density functional theory (DFT) method at the B3LYP/6-31G(d)-GD3BJ level via Gaussian 16 program package [36]. The ECD were calculated using time-dependent density functional theory (TDDFT) at a B3LYP/6-311+G(d,p) level in methanol with IEFPCM model. The calculated ECD curves were all generated using the SpecDis 1.71 program package, and the calculated ECD data of all conformers were Boltzmann averaged using Gibbs free energy [37].

### 3.10. Antimicrobial Activity Assay

The isolated compounds were assayed against the pathogenic bacteria methicillin-resistant coagulase-negative staphylococci (MRCNS), methicillin-resistant *Staphylococcus aureus* (MRSA), *Acinetobacter baumannii*, *Bacillus cereus*, *Pseudomonas aeruginosa*, and *S. aureus*, as well as the pathogenic fungus *Canidia albicans*. The detailed methodologies for biological testing were performed as previously described [38,39]. Ciprofloxacin or Nystatin was used as the positive control.

### 3.11. Cytotoxicity Assay

Five human cancer cell lines, K562 (using the MTT method), ASPC-1, MDA-MB-231, H69AR, and L-02 (using the SRB method) cells, were used in the cytotoxic assay. Adriamycin (Sigma-Aldrich Inc., Washington, USA) was used as the positive control. The detailed methodologies for biological testing were performed as previously described [40,41].

## 4. Conclusions

Fungi-derived secondary metabolites have always been an important source of natural medicines [42]. However, the success rate for discovering new compounds from fungi is hindered by the gene being silent or poorly expressed under laboratory conditions [43]. In this study, a total of 13 labdane diterpenes belonging to four skeleton types, including five undescribed analogs, were obtained by heterologous expression in *A. nidulans*. Compounds **3**–**13** were all produced under the catalysis of tailoring enzymes in the BGC *labd*. Moreover, in the in vivo conversion process of isolated compounds, the three P450s LabdC, LabdE, and LabdF were disclosed for the first time to be responsible for catalyzing multi-step chemical reactions, such as hydroxylation, methylation, decarboxylation, etc. It is remarkable that **4** possessed a unique 3,18-dinor-2,3:4,18-*diseco*-labdane core structure which could serve as a key intermediate in the biosynthetic route of a group of ring A-cleaved and rearranged labdane diterpene derivatives, such as penioxalicin and **13**.

## Figures and Tables

**Table 1:** ^1^H and ^13^C NMR spectroscopic data for compounds **3** and **4** in CD_3_OD (125 and 500 MHz).

No.	3		4	
	*δ*_C_, Mult	*δ*_H_ (*J* in Hz)	*δ*_C_, Mult	*δ*_H_ (*J* in Hz)
1a	49.0, CH_2_	2.08, m	42.9, CH_2_	2.49, d (15.7)
1b		1.00, m		2.14, d (15.7)
2	65.5, CH	3.81, m	174.3, C	–
3a	45.0, CH_2_	2.17, m	–	–
3b		0.89, m		
4	41.5, C	–	215.1, C	–
5	56.9, CH	1.26, m	63.6, CH	3.39, d (11.0)
6a	25.2, CH_2_	1.85, m	71.5, CH	3.85, td (11.0, 5.0)
6b		1.34, m		
7a	39.5, CH_2_	2.42, m	47.6, CH_2_	2.65, dd (12.0, 5.0)
7b		1.96, m		2.02, overlapped
8	148.7, C	–	145.3, C	–
9	57.4, CH	1.70, m	49.5, CH	2.27, m
10	41.8, C	–	42.0, C	–
11a	23.0, CH_2_	1.75, m	23.8, CH_2_	1.82, m
11b		1.57, m		1.58, m
12a	40.7, CH_2_	2.31, m	40.7, CH_2_	2.29, m
12b		2.02, m		2.02, overlapped
13	161.9, C	–	161.8, C	–
14	116.8, CH	5.62, d (1.2)	116.8, CH	5.64, d (1.5)
15	170.2, C	–	170.3, C	–
16	18.9, CH_3_	2.14, d (1.2)	19.0, CH_3_	2.15 d (1.5)
17a	107.8, CH_2_	4.90, s	111.0, CH_2_	5.08, s
17b		4.57, s		4.75, s
18a	65.5, CH_2_	3.61, d (11.5)	–	–
18b		3.29, d (11.5)		
19	28.1, CH_3_	1.01, s	35.2, CH_3_	2.32, s
20	16.7, CH_3_	0.74, s	17.9, CH_3_	0.81, s

**Table 2:** ^1^H and ^13^C NMR spectroscopic data for compounds **5**–**7** in CD_3_OD (125 and 500 MHz).

No.	5		6		7	
	*δ*_C_, Mult	*δ*_H_ (*J* in Hz)	*δ*_C_, Mult	*δ*_H_ (*J* in Hz)	*δ*_C_, Mult	*δ*_H_ (*J* in Hz)
1a	48.4, CH_2_	2.16, m	39.6, CH_2_	1.91, m	48.3, CH_2_	2.17, m
1b		1.22, m		1.35, m		1.25, m
2a	64.6, CH	3.83 m	18.8, CH_2_	1.57, m	64.1, CH	3.82, m
2b				1.50, m		
3a	52.6, CH_2_	1.65, m	37.2, CH_2_	1.94, m	45.6, CH_2_	2.25, m
3b		1.14, m		0.96, m		0.91, m
4	35.1, C	–	39.0, C	–	41.0, C	–
5	63.6, CH	2.21, s	65.5, CH	2.43, s	64.6, CH	2.44, s
6	202.0, C	–	203.0, C	–	202.1, C	–
7	129.2, CH	5.77, s	128.8, CH	5.76, s	129.0, CH	5.78, s
8	161.9, C	–	162.6, C	–	162.4, C	–
9	57.0, CH	2.32, m	57.2, CH	2.27, m	57.2, CH	2.35, m
10	45.8, C	–	44.5, C	–	45.7, C	–
11a	26.5, CH_2_	1.69, m	26.5, CH_2_	1.66, m	26.6, CH_2_	1.70, m
11b				1.61, m		
12a	43.9, CH_2_	2.49, m	43.9, CH_2_	2.48, m	43.9, CH_2_	2.49, m
12b		2.27, m		2.25, m		2.29, m
13	160.5, C	–	160.6, C	–	160.5, C	–
14	117.5, CH	5.74, d (1.2)	117.5, CH	5.73 d (1.2)	117.5, CH	5.74, d (1.2)
15	170.1, C	–	170.1, C	–	170.0, C	–
16	19.0, CH_3_	2.20, d (1.2)	19.0, CH_3_	2.18, d (1.2)	19.0, CH_3_	2.20, d (1.2)
17	22.3, CH_3_	2.02 s	22.4, CH_3_	2.01, s	22.3, CH_3_	2.02, s
18a	22.9, CH_3_	1.20, s	63.7, CH_2_	4.10, d (11.0)	64.3, CH_2_	4.11, d (11.0)
18b				3.63, d (11.0)		3.57, d (11.0)
19	33.8, CH_3_	1.17, s	27.3, CH_3_	1.15, s	27.5, CH_3_	1.20, s
20	15.9, CH_3_	0.91, s	15.8, CH_3_	0.84, s	16.7, CH_3_	0.88, s

## Data Availability

The data presented in this study are available in this article and the supplementary materials.

## Supplementary Materials

The following supporting information can be downloaded at https://www.mdpi.com/article/10.3390/md21120628/s1: Table S1. Fungal strains and plasmids used in this study; Table S2. PCR primer sets utilized in this study; Table S3. Annotation of each gene in the *labd* cluster from *Talaromyces* sp. HDN151403; Table S4. ^1^H and ^13^C NMR spectroscopic data for compounds **1** and **2** in CD_3_OD (125 and 500 MHz); Table S5. ^1^H and ^13^C NMR spectroscopic data for compounds **8**–**10** in CD_3_OD (125 and 500 MHz); Table S6. ^1^H and ^13^C NMR spectroscopic data for compounds **11**–**13** in CD_3_OD (125 and 500 MHz); Figure S1. Representative labdane diterpenoid biosynthetic gene clusters and their biosynthetic pathways in Actinomycetes, fungi, and plants; Figure S2. Cluster heatmap visualization of cblaster search results using the *labd* cluster to query the fungal genomes from online database; Figure S3. Visualization of gene clusters homologous to *labd* cluster searched with cblaster using clinker tool; Figure S4. Neighbor joining method-based phylogenetic analysis of P450s from Ascomycetes fungi using MEGA X 10.2.2 software; Figure S5. Results of protein family classification analysis of LabdC using InterPro database; Figure S6. Protein sequence alignment of LabdC with other homologs using Blastp (protein–protein BLAST); Figure S7. Protein sequence alignment of LabdE with other homologs using Blastp (protein–protein BLAST); Figure S8. Protein sequence alignment of LabdF with other homologs using Blastp (protein–protein BLAST); Figure S9. RT-PCR results of *labd* cluster of *Talaromyces* sp. HDN 151403 under different laboratory culture conditions; Figure S10. UV-vis spectra of purified compounds; Figure S11. Comparison of calculated and experimental ECD spectra of **3**–**7** in methanol; Figures S12–S19: NMR, HRESIMS, and UV spectra of compound **3**; Figures S20–S27: NMR, HRESIMS, and UV spectra of compound **4**; Figures S28–S35: NMR, HRESIMS, and UV spectra of compound **5**; Figures S36–S43: NMR, HRESIMS, and UV spectra of compound **6**; Figures S44–S51: NMR, HRESIMS, and UV spectra of compound **7**; Figures S52 and S53: ^1^H and ^13^C NMR spectra of compound **1**; Figures S54 and S55: ^1^H and ^13^C NMR spectra of compound **2**; Figures S56 and S57: ^1^H and ^13^C NMR spectra of compound **8**; Figures S58 and S59: ^1^H and ^13^C NMR spectra of compound **9**; Figures S60 and S61: ^1^H and ^13^C NMR spectra of compound **10**; Figures S62 and S63: ^1^H and ^13^C NMR spectra of compound **11**; Figures S64 and S65: ^1^H and ^13^C NMR spectra of compound **12**; Figures S66 and S67: ^1^H and ^13^C NMR spectra of compound **13**; Figures S68–S72: Main conformers of **3**–**7** in ECD calculations and the energy analysis for optimized geometries of dominant conformers at B3LYP/6-31G(d)-GD3BJ level in the gas phase; Supplementary S1. The Z-matrices of (2*R**,4*S**,5*R**,9*S**,10*R**)-**3** optimized at B3LYP/6-31G(d)-GD3BJ level via Gaussian; Supplementary S2. The Z-matrices of (5*S**,6*S**,9*S**,10*R**)-**4** optimized at B3LYP/6-31G(d)-GD3BJ level via Gaussian; Supplementary S3. The Z-matrices of (2*S**,5*S**,9*S**,10*R**)-**5** optimized at B3LYP/6-31G(d)-GD3BJ level via Gaussian; Supplementary S4. The Z-matrices of (4*S**,5*R**,9*S**,10*R**)-**6** optimized at B3LYP/6-31G(d)-GD3BJ level by via Gaussian; Supplementary S5. The Z-matrices of (2*R**,4*S**,5*R**,9*S**,10*R**)-**7** optimized at B3LYP/6-31G(d)-GD3BJ level via Gaussian.
