# Supplementary material for: Extending the Structural Diversity of Labdane Diterpenoids from Marine-Derived Fungus Talaromyces sp. HDN151403 Using Heterologous Expression
Source: Mar Drugs. 2023 Dec 3;21(12):628. doi: 10.3390/md21120628 (PMC10744899; doi:10.3390/md21120628)
Supplement: Supplementary file 1 [file marinedrugs-21-00628-s001.zip › marinedrugs-2742813-supplementary.pdf]

Extending the structural diversity of labdane diterpenoids from marine derived fungus *Talaromyces* sp. HDN151403 using heterologous expression

Falei Zhang [1,#], Chuanteng Ma [1,#], Qian Che [1], Tianjiao Zhu [1], Guojian Zhang [1,2,3] and Dehai Li [1,2,*]

Contents

Table S1. Fungal strains and plasmids used in this study 4
Table S2. PCR primer sets utilized in this study 6
Table S3. Annotation of each gene in the *labd* cluster from *Talaromyces* sp. HDN151403. 8
Table S4. ^{1}H and ^{13}C NMR Spectroscopic Data for Compounds **1** and **2** in CD_3OD (125 and 500 MHz) 9
Table S5. ^{1}H and ^{13}C NMR Spectroscopic Data for Compounds **8–10** in CD_3OD (125 and 500 MHz) 10
Table S6. ^{1}H and ^{13}C NMR Spectroscopic Data for Compounds **11–13** in CD_3OD (125 and 500 MHz) 11
Figure S1. Representative labdane diterpenoid biosynthetic gene clusters and their biosynthetic pathways in Actinomycetes, fungi and plants. 12
Figure S2. Cluster heatmap visualization of cblaster[9] search results using the *labd* cluster to query the fungal genomes from online database 14
Figure S3. Visualization of gene clusters homologous to *labd* cluster searched by cblaster using clinker tool.[10] 15
Figure S4. Neighbor joining method based phylogenetic analysis of P450s from Ascomycetes fungi using MEGA X software 16
Figure S5. Results of protein family classification analysis of LabdC using InterPro database 17
Figure S6. Protein sequence alignment of LabdC with other homologs using Blastp (protein-protein BLAST). 18
Figure S7. Protein sequence alignment of LabdE with other homologs using Blastp (protein-protein BLAST). 19
Figure S8. Protein sequence alignment of LabdF with other homologs using Blastp (protein-protein BLAST). 20
Figure S9. RT-PCR results of *labd* cluster of *Talaromyces* sp. HDN 151403 under different laboratory culture conditions 21
Figure S10. UV-vis spectra of purified compounds. 22
Figure S11. Comparison of calculated and experimental ECD spectra of **3–7** in methanol 23
Figure S12. ^{1}H NMR of **3** in CD_3OD. 24
Figure S13. ^{13}C NMR of **3** in CD_3OD. 24
Figure S14. HSQC of **3** in CD_3OD. 25
Figure S15. HMBC of **3** in CD_3OD. 25
Figure S16. ^{1}H-^{1}H COSY of **3** in CD_3OD. 26
Figure S17. ROESY of **3** in CD_3OD. 26
Figure S18. HRESIMS of **3** 27
Figure S19. UV spectrum of **3**. 27
Figure S20. ^{1}H NMR of **4** in CD_3OD. 28
Figure S21. ^{13}C NMR and DEPT of **4** in CD_3OD. 28

Figure S22. HSQC of **4** in CD_3OD. 29
Figure S23. HMBC of **4** in CD_3OD. 29
Figure S24. ^1H-^1H COSY of **4** in CD_3OD. 30
Figure S25. ROESY of **4** in CD_3OD. 30
Figure S26. HRESIMS of **4** 31
Figure S27. UV spectrum of **4**. 31
Figure S28. ^1H NMR of **5** in CD_3OD. 32
Figure S29. ^{13}C NMR of **5** in CD_3OD. 32
Figure S30. HSQC of **5** in CD_3OD. 33
Figure S31. HMBC of **5** in CD_3OD. 33
Figure S32. ^1H-^1H COSY of **5** in CD_3OD. 34
Figure S33. ROESY of **5** in CD_3OD. 34
Figure S34. HRESIMS of **5** 35
Figure S35. UV spectrum of **5**. 35
Figure S36. ^1H NMR of **6** in CD_3OD. 36
Figure S37. ^{13}C NMR of **6** in CD_3OD. 36
Figure S38. HSQC of **6** in CD_3OD. 37
Figure S39. HMBC of **6** in CD_3OD. 37
Figure S40. ^1H-^1H COSY of **6** in CD_3OD. 38
Figure S41. ROESY of **6** in CD_3OD. 38
Figure S42. HRESIMS of **6** 39
Figure S43. UV spectrum of **6**. 39
Figure S44. ^1H NMR of **7** in CD_3OD. 40
Figure S45. ^{13}C NMR of **7** in CD_3OD. 40
Figure S46. HSQC of **7** in CD_3OD. 41
Figure S47. HMBC of **7** in CD_3OD. 41
Figure S48. ^1H-^1H COSY of **7** in CD_3OD. 42
Figure S49. ROESY of **7** in CD_3OD. 42
Figure S50. HRESIMS of **7** 43
Figure S51. UV spectrum of **7**. 43
Figure S52. ^1H NMR of **1** in CD_3OD. 44
Figure S53. ^{13}C NMR of **1** in CD_3OD. 44
Figure S54. ^1H NMR of **2** in CD_3OD. 45
Figure S55. ^{13}C NMR of **2** in CD_3OD. 45
Figure S56. ^1H NMR of **8** in CD_3OD. 46
Figure S57. ^{13}C NMR of **8** in CD_3OD. 46
Figure S58. ^1H NMR of **9** in CD_3OD. 47
Figure S59. ^{13}C NMR of **9** in CD_3OD. 47
Figure S60. ^1H NMR of **10** in CD_3OD. 48
Figure S61. ^{13}C NMR of **10** in CD_3OD. 48
Figure S62. ^1H NMR of **11** in CD_3OD. 49
Figure S63. ^{13}C NMR of **11** in CD_3OD. 49
Figure S64. ^1H NMR of **12** in CD_3OD. 50
Figure S65. ^{13}C NMR of **12** in CD_3OD. 50

Figure S66. ^{1}H NMR of **13** in CD_3OD. 51
Figure S67. ^{13}C NMR of **13** in CD_3OD. 51
Figure S68. Main conformers of (2*R**,4*S**,5*R**,9*S**,10*R**)-**3** in ECD calculations and the energy analysis for optimized geometries of dominant conformers at B3LYP/6-31G(d)-GD3BJ level in the gas phase 52
Figure S69. Main conformers of (5*S**,6*S**,9*S**,10*R**)-**4** in ECD calculations and the energy analysis for optimized geometries of dominant conformers at B3LYP/6-31G(d)-GD3BJ level in the gas phase 54
Figure S70. Main conformers of (2*S**,5*S**,9*S**,10*R**)-**5** in ECD calculations and the energy analysis for optimized geometries of dominant conformers at B3LYP/6-31G(d)-GD3BJ level in the gas phase 55
Figure S71. Main conformers of (4*S**,5*R**,9*S**,10*R**)-**6** in ECD calculations and the energy analysis for optimized geometries of dominant conformers at B3LYP/6-31G(d)-GD3BJ level in the gas phase 56
Figure S72. Main conformers of (2*R**,4*S**,5*R**,9*S**,10*R**)-**7** in ECD calculations and the energy analysis for optimized geometries of dominant conformers at B3LYP/6-31G(d)-GD3BJ level in the gas phase 58
Supplementary S1. The Z-matrices of (2*R**,4*S**,5*R**,9*S**,10*R**)-**3** optimized at B3LYP/6-31G(d)-GD3BJ level by Gaussian 60
Supplementary S2. The Z-matrices of (5*S**,6*S**,9*S**,10*R**)-**4** optimized at B3LYP/6-31G(d)-GD3BJ level by Gaussian. 66
Supplementary S3. The Z-matrices of (2*S**,5*S**,9*S**,10*R**)-**5** optimized at B3LYP/6-31G(d)-GD3BJ level by Gaussian. 68
Supplementary S4. The Z-matrices of (4*S**,5*R**,9*S**,10*R**)-**6** optimized at B3LYP/6-31G(d)-GD3BJ level by Gaussian. 72
Supplementary S5. The Z-matrices of (2*R**,4*S**,5*R**,9*S**,10*R**)-**7** optimized at B3LYP/6-31G(d)-GD3BJ level by Gaussian 78

Table S1. Fungal strains and plasmids used in this study.

Strain/plasmid	Primer name	Descrption	Reference
Talaromyces sp. HDN151403		Antarctica sponge-derived fungus, GenBank: MW888514	[34]
E. coli XL1-blue		*recA1 endA1 gyrA96 thi-1 hsdR17supE44 relA1 lac* [*F' pro AB lac IqZΔM15 Tn10* (*Tetr*)]	This study
Saccharomyces cerevisiae BJ5464-NpgA		(*MATα ura3-52 his3-Δ200 leu2-Δ1 trp1 pep4::HIS3 prb1 Δ1.6R can1 GAL*)	This study
Aspergillus nidulans A1145		*pyrG89*; *pyroA4*; *nkuA::argB*; *riboB2*	This study
AN-empty vectors		Harboring three empty pANU, pANR, and pANP vectors	This study
AN-*labdA*		Harboring the plasmid pANU-*labdA*	This study
AN-*labdAB*		Harboring the plasmids pANU-*labdA* and pANP-*labdB*	This study
AN-*labdABE*		Harboring the plasmids pANU-*labdA*-*labdE* and pANP-*labdB*	This study
AN-*labdABF*		Harboring the plasmids pANU-*labdA*-*labdF* and pANP-*labdB*	This study
AN-*labdABEF*		Harboring the plasmids pANU-*labdA*-*labdE*-*labdF* and pANP-*labdB*	This study
AN-*labdABEFG*		Harboring the plasmids pANU-*labdA*-*labdE*-*labdF*, pANP-*labdB* and pANR-*labdG*	This study
AN-*labdABDEFG*		Harboring the plasmids pANU-*labdA*-*labdE*-*labdF*, pANP-*labdB* and pANR-*labdD*-*labdG*	This study
AN-*labdABCDEG*		Harboring the plasmids pANU-*labdA*-*labdE*, pANP-*labdB* and pANR-*labdC*-*labdD*-*labdG*	This study
AN-*labdABCDEFG*		Harboring the plasmids pANU-*labdA*-*labdE*-*labdF*, pANP-*labdB* and pANR-*labdC*-*labdD*-*labdG*	This study
AN-*labdCDEFG*		Harboring the plasmids pANU-*labdE*-*labdF* and pANR-*labdC*-*labdD*-*labdG*	This study
AN-*labdE*		Harboring the plasmids pANU-*labdE*	This study
AN-*labdG*		Harboring the plasmids pANR-*labdG*	This study
pANR		*E. coli*-yeast-fungal shuttle vectors, ampicillin resistance, selection is by *riboB*.	[35]
pANP		*E. coli*-yeast-fungal shuttle vectors, ampicillin resistance, selection is by *pyroA*.	[35]
pANU		*E. coli*-yeast-fungal shuttle vectors, ampicillin resistance, selection is by *pyrG*.	[35]
pANU-*labdA*	U-*labdA*-F1/U-*labdA*-R1	*labdA* gDNA with downstream 400 bp used the promoter *glaA* in *Pac*I-linearized pANU	This study
pANU-*labdA*-*labdE*-*labdF*	U-*labdA*-F2/U-*labdA*-R2 U-*labdE*-F1/U-*labdE*-R1	*labdA* gDNA with downstream 400 bp, *labdE* gDNA with downstream 500 bp and *labdF* gDNA with	This study

	U-*labdF*-F1/U-*labdF*-R1	downstream 400 bp in *Pac*I-linearized pANU	
pANU-*labdA*-*labdE*	U-*labdA*-F2/U-*labdA*-R2<br>U-*labdE*-F1/U-*labdE*-R2	*labdA* gDNA with downstream 400 bp and *labdE* gDNA with downstream 500 bp in *Pac*I-linearized pANU	This study
pANU-*labdA*-*labdF*	U-*labdA*-F2/U-*labdA*-R2<br>U-*labdF*-F2/U-*labdF*-R1	*labdA* gDNA with downstream 400 bp and *labdF* gDNA with downstream 400 bp in *Pac*I-linearized pANU	This study
pANU-*labdE*-*labdF*	U-*labdE*-F2/U-*labdE*-R1<br>U-*labdF*-F1/U-*labdF*-R1	*labdE* gDNA with downstream 500 bp and *labdF* gDNA with downstream 400 bp in *Pac*I-linearized pANU	This study
pANU-*labdE*	U-*labdE*-F2/U-*labdE*-R2	*labdE* gDNA with downstream 500 bp in *Pac*I-linearized pANU	This study
pANR-*labdC*-*labdD*-*labdG*	R-*labdC*-F1/R-*labdC*-R1<br>R-*labdD*-F1/R-*labdD*-R1<br>R-*labdG*-F1/R-*labdG*-R1	*labdC* gDNA with downstream 500 bp, *labdD* gDNA with downstream 500 bp and *labdG* gDNA with downstream 500 bp in *Pac*I-linearized pANR	This study
pANR-*labdD*-*labdG*	R-*labdD*-F2/R-*labdD*-R1<br>R-*labdG*-F1/R-*labdG*-R1	*labdD* gDNA with downstream 500 bp and *labdG* gDNA with downstream 500 bp in *Pac*I-linearized pANR	This study
pANR-*labdG*	R-*labdG*-F1/R-*labdG*-R2	*labdG* gDNA with downstream 500 bp in *Pac*I-linearized pANR	This study
pANP-*labdB*	P-*labdB*-F1/P-*labdB*-R1	*labdB* gDNA with downstream 500 bp used the promoter *amyB* in *Pac*I-linearized pANP	This study

Table S2. PCR primer sets utilized in this study.

Primer name	Oligonucleotide sequence (5′→3′)[a]
amyB-F	GATTAAAGGTGCCGAACGAGC
amyB-R	AAATGCCTTCTGTGGGGTTTATTG
glaA-F	CCTGATCTTCCGAACTGGTCG
glaA-R	TGCTGAGGTGTAATGATGCTGG
gpdA-F	ACTCCGGTGAATTGATTTGGGTG
gpaA-R	TGTTTAGATGTGTCTATGTGGCGG
labdA-F	ATGGGCCCAATGGATTTGC
labdA-R	TCATAACTCAAGGGACATGCGG
labdB-F	ATGTCTAATGACACCACTACCACGG
labdB-R	TCAATTCGTAATGGCCAGTGTATGAC
labdC-F	ATGATCACAGATCATGTCTCCCAGC
labdC-R	CCAAGCGGCGTAGTATGCC
labdD-F	GTCCAGTATGACAATGCTGCAG
labdD-R	GCGTGCCTAGAACAGCGATAG
labdE-F	ATGGCTCCCAGGATCAGTC
labdE-R	TCAAGCTTTTGTAGCTCTGGTGG
labdF-F	ATGTCCACCGCAAAGCGTG
labdF-R	TCACACAAGTATCGGTCGTAGTCG
labdG-F	ATGGCATCTAAACTGCCCGC
labdG-R	TCAATCCAAGGGTTTGTGAGG
ITS-1	TCCGTAGGTGAACCTGCGG
ITS-4	TCCTCCGCTTATTGATATGC
Tubulin-F	ATTTGCCATCTTCACATCGGTCAGGCTG
Tubulin-R	TTAGTACTCGGCATCACCCTCGACATCC
U-*labdA*-F1	ATCCCCAGCATCATTACACCTCAGCATTAATTAAGGCATGGGCCCAATGGATTTGCAAG
U-*labdA*-R1	CTGCAGCCCGGGGGATCCACTAGTTCTAGAGCGGCCGGTACCAGACGACACGATAGTTG
U-*labdA*-F2	CTGAGCTTCATCCCCAGCATCATTACACCTCAGCATTAATATGGGCCCAATGGATTTGC
U-*labdA*-R2	CTTGGGTCTCTCCCGTCACCCAAATCAATTCACCGGAGTGGTACCAGACGACACGATAG
P-*labdB*-F1	TTCTCTGAACAATAAACCCCACAGAAGGCATTTTTAATATGTCTAATGACACCACTACC
P-*labdB*-R1	CGATAAGCTTGATATCGAATTCCTGCAGCCCGGGGGATCTTGAATCTGAAGTCCACGGC
U-*labdE*-F1	TTGACTAACCATTACCCCGCCACATAGACACATCTAAACAATGGCTCCCAGGATCAGTC
U-*labdE*-F2	CTGAGCTTCATCCCCAGCATCATTACACCTCAGCATTAATATGGCTCCCAGGATCAGTC
U-*labdE*-R1	ATTGTTATATCATTTATAGCTCGTTCGGCACCTTTAATCTAGGTTGGATGACGTGCCAG
U-*labdE*-R2	TCCTGCAGCCCGGGGGATCCACTAGTTCTAGAGCGGCCTAGGTTGGATGACGTGCCAGG
U-*labdF*-F1	AGCTCTCCCTTCTCTGAACAATAAACCCCACAGAAGGCATTTATGTCCACCGCAAAGCG
U-*labdF*-F2	CTCTCCCTTCTCTGAACAATAAACCCCACAGAAGGCATTTATGTCCACCGCAAAGCGTG
U-*labdF*-R1	CCTGCAGCCCGGGGGATCCACTAGTTCTAGAGCGGCCGCGTATCATGTGTAGCACCATC
R-*labdC*-F1	GCCTGAGCTTCATCCCCAGCATCATTACACCTCAGCAATGATCACAGATCATGTCTCCC
R-*labdC*-R1	TGTTATATCATTTATAGCTCGTTCGGCACCTTTAATCCCTGACGGCGATACATACCTAG
R-*labdD*-F1	CTCCCTTCTCTGAACAATAAACCCCACAGAAGGCATTTATGTCTACTGCGAAAGTCCAG
R-*labdD*-F2	TGAGCTTCATCCCCAGCATCATTACACCTCAGCAATGTCTACTGCGAAAGTCCAGTATG
R-*labdD*-R1	GATAAGCTTGATATCGAATTCCTGCAGCCCGGGGGATCCGAGCGTTACCTCACTTACAG

R-*labdG*-F1	CCATTACCCCGCCACATAGACACATCTAAACATTAATGACACAATTGCCTTCAAGATGC
R-*labdG*-R1	GTCATAGGTCGCCAGGTACGACCAGTTCGGAAGATCAGGCCTGAACGATGGATGAAGCC
R-*labdG*-R2	CGATAAGCTTGATATCGAATTCCTGCAGCCCGGGGGATCCCTGAACGATGGATGAAGCC

[a] The underlined sequence is a homologous recombination binding region.

Table S3. Annotation of each gene in the *labd* cluster from *Talaromyces* sp. HDN151403.

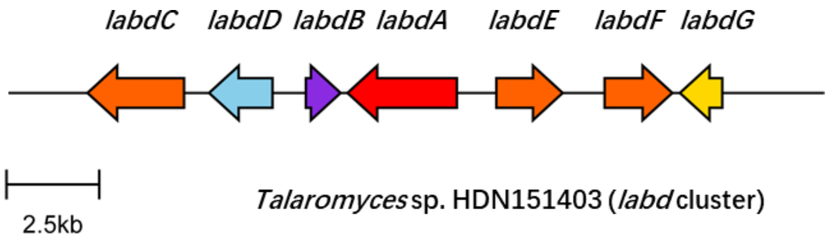

Genes	Size	Translate	Putative Function	Closest Homolog (Origin, Accession No.)	Identity (%)
labdC	780	–	Bifunctional P450/methyltransferase.	*Talaromyces verruculosus*, KUL89355.1	758/780(97%)
labdD	509	–	Aldehyde dehydrogenase	*Talaromyces pinophilus*, GAM41999.1	484/509(95%)
labdB	311	+	Tyrosine phosphatase	*Talaromyces verruculosus*, KUL89334.1	300/311(96%)
labdA	960	–	Type II terpene cyclase	*Talaromyces verruculosus*, A0A348FUE1.1	860/963(89%)
labdE	501	+	P450	*Talaromyces pinophilus*, KAF3385867.1	500/501(99%)
labdF	542	+	P450	*Talaromyces verruculosus*, KUL89225.1	527/542(97%)
labdG	314	–	SDR	*Talaromyces verruculosus*, KUL89357.1	308/314(98%)

Table S4. ^1H and ^{13}C NMR Spectroscopic Data for Compounds **1** and **2** in CD_3OD (125 and 500 MHz).

no.	**1**		**2**	
	**δ_C, mult**	**δ_H (J in Hz)**	**δ_C, mult**	**δ_H (J in Hz)**
1a	38.3, CH_2	1.81, m	38.3, CH_2	1.81, m
1b		1.19, m		1.19, m
2	28.6, CH_2	1.65 m	28.6, CH_2	1.65, m
3	79.4, CH	3.20, m	79.4, CH	3.20, m
4	40.2, C	–	40.2, C	–
5	56.0, CH	1.12, dd (12.5, 2.8)	56.0, CH	1.12, dd (12.5, 3.3)
6a	25.3, CH_2	1.76, m	25.2, CH_2	1.76, m
6b		1.41, m		1.40, m
7a	39.3, CH_2	2.40, m	39.4, CH_2	2.40, m
7b		1.98, m		1.98, m
8	149.3, C	–	149.5, C	–
9	57.1, CH	1.61, m	58.3, CH	1.56, m
10	40.4, C	–	40.5, C	–
11a	22.8, CH_2	1.68, m	22.2, CH_2	1.54, m
11b		1.56, m		1.33, m
12a	40.7, CH_2	2.31, m	37.0, CH_2	1.51, m
12b		2.01, m		1.02, m
13	161.9, C	–	32.2, CH	1.89, m
14a	116.8, CH	5.61, s	42.5, CH_2	2.28, dd (14.7, 6.6)
14b				2.06, dd (14.7, 8.0)
15	170.3, C	–	177.2, C	–
16	18.9, CH_3	2.13, d (1.2)	20.4, CH_3	0.96, d (6.6)
17a	107.2, CH_2	4.89, s	107.2, CH_2	4.83, s
17b		4.55, s		4.52, s
18	16.1, CH_3	0.77, s	16.1, CH_2	0.77, s
19	28.9, CH_3	0.98, s	28.9, CH_3	0.98, s
20	15.0, CH_3	0.73, s	15.0, CH_3	0.72, s

Table S5. ^{1}H and ^{13}C NMR Spectroscopic Data for Compounds **8–10** in CD_3OD (125 and 500 MHz).

no.	**8**		**9**		**10**	
	**δ_C, mult**	**δ_H (J in Hz)**	**δ_C, mult**	**δ_H (J in Hz)**	**δ_C, mult**	**δ_H (J in Hz)**
1a	40.8, CH_2	1.85–1.94, m	47.5, CH_2	2.37–2.44, m	41.8, CH_2	1.70–1.79, m
1b		1.05–1.15, m		0.98–1.04, m		1.47, t (12.2)
2a	21.2, CH_2	1.85–1.94, m	65.5, CH	4.11, m	67.7, CH	4.16, m
2b		1.49–1.57 m				
3a	39.9, CH_2	2.11–2.15, m	48.9, CH_2	1.99–2.06, m	75.1, CH	3.96, d (2.6)
3b		1.05–1.15, m		0.98–1.04, m		
4	45.1, C	–	46.0, C	–	49.8, C	–
5	57.4, CH	1.36, m	56.7, CH	1.34, dd (12.5, 2.9)	48.9, CH	1.70–1.79, m
6a	27.6, CH_2	1.99–2.04, m	27.1, CH_2	1.99–2.06, m	26.7, CH_2	1.86–1.95, m
6b		1.85–1.94, m		1.83, m		
7a	40.4, CH_2	2.42, m	39.6, CH_2	2.37–2.44, m	39.7, CH_2	2.42, m
7b		1.85–1.94, m		1.94, m		1.86–1.95, m
8	149.4, C	–	148.8, C	–	149.0, C	–
9	56.6, CH	1.64, dd (11.0, 1.7)	56.5, CH	1.69, m	56.4, CH	1.70–1.79, m
10	41.5, C	–	42.4, C	–	41.7, C	–
11a	22.9, CH_2	1.73, m	23.0, CH_2	1.76, m	23.0, CH_2	1.70–1.79, m
11b		1.49–1.57 m		1.57, m		1.57, m
12a	39.3, CH_2	2.30, m	40.7, CH_2	2.31, m	40.7, CH_2	2.31, m
12b		1.99–2.04, m		2.11, m		2.04, m
13	162.0, C	–	161.9, C	–	161.9, C	–
14	116.7, CH	5.61, d (1.2)	116.8, CH	5.62, d (1.2)	116.9, CH	5.62, s
15	170.2, C	–	170.2, C	–	170.3, C	–
16	18.9, CH_3	2.13, d (1.2)	18.9, CH_3	2.14, d (1.2)	18.9, CH_3	2.14, d (1.2)
17a	106.9, CH_2	4.89, s	107.6, CH_2	4.93, s	107.4, CH_2	4.92, s
17b		4.54, s		4.57, s		4.56, s
18	181.2, C	–	180.5, C	–	180.3, C	–
19	29.6, CH_3	1.20, s	29.5, CH_3	1.26, s	25.0, CH_3	1.30, s
20	13.4, CH_3	0.65, s	14.2, CH_3	0.66, s	14.2, CH_3	0.67, s

Table S6. ^{1}H and ^{13}C NMR Spectroscopic Data for Compounds **11–13** in CD_3OD (125 and 500 MHz).

no.	**11**		**12**		**13**	
	**δ_C, mult**	**δ_H (J in Hz)**	**δ_C, mult**	**δ_H (J in Hz)**	**δ_C, mult**	**δ_H (J in Hz)**
1a	54.8, CH_2	2.49, m	51.5, CH_2	2.62, m	38.5, CH_2	3.09, d (15.0)
1b		2.27–2.34, m		2.36, m		2.71, d (15.0)
2	212.0, C	–	194.5, C	–	173.9, C	–
3a	52.2, CH_2	2.75, dd (14.0, 2.1)	146.4, C	–	–	–
3b		2.27–2.34, m				
4	48.9, C	–	134.7, C	–	39.3, CH	2.89, p (7.5)
5	56.1, CH	1.92, m	55.0, CH	2.58, d (11.0)	52.9, CH	2.66, dd (11.4, 7.5)
6a	26.9, CH_2	2.15, m	70.2, CH	3.85, td (11.0, 5.1)	78.7, CH	4.99, td (11.4, 4.8)
6b		1.84, m				
7a	39.0, CH_2	2.45, m	48.8, CH_2	2.77, m	43.0, CH_2	3.03, dd (11.4, 4.8)
7b		2.06, m		2.15, m		2.25, t (11.4)
8	148.2, C	–	145.0, C	–	142.8, C	–
9	55.4, CH	2.00, m	53.0, CH	2.02, m	49.5, CH	2.48, d (10.6)
10	45.0, C	–	45.0, C	–	51.9, C	–
11a	22.9, CH_2	1.59, m	23.0, CH_2	1.62, m	24.7, CH_2	1.86, m
11b						1.46, m
12a	40.4, CH_2	2.27–2.34, m	40.3, CH_2	2.36, m	40.5, CH_2	2.34, m
12b		2.06, m		2.11, m		2.10, m
13	161.5, C	–	161.3, C	–	160.8, C	–
14	117.0, CH	5.62, d (1.2)	117.0, CH	5.65, s	117.1, CH	5.62, s
15	170.1, C	–	170.1, C	–	170.1, C	–
16	18.9, CH_3	2.13, d (1.2)	18.8, CH_3	2.16, d (1.2)	18.9, CH_3	2.15, d (1.2)
17a	108.1, CH_2	4.98, s	110.0, CH_2	5.08, s	114.3, CH_2	5.20, s
17b		4.62, s		4.72, s		4.88, s
18	179.5, C	–	–	–	180.9, C	–
19	28.3, CH_3	1.35, s	15.3, CH_3	2.10, d (2.0)	10.4, CH_3	1.07, d (7.5)
20	14.2, CH_3	0.66, s	14.0, CH_3	0.71, s	173.3, C	–
-COOC*H*$_3$	–	–	–	–	52.2, CH_3	3.65, s

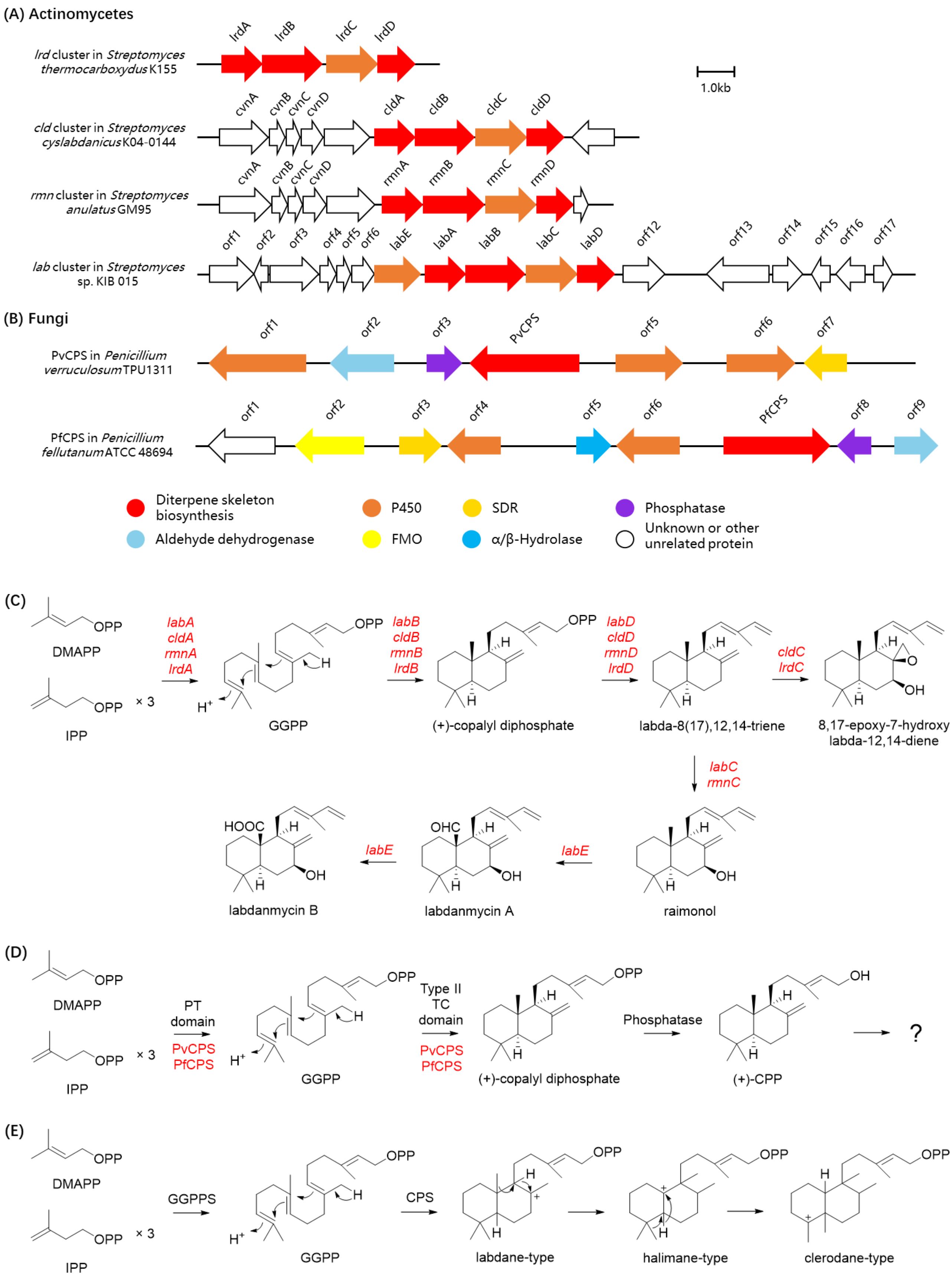

Figure S1. Representative labdane diterpenoid biosynthetic gene clusters and their biosynthetic pathways in Actinomycetes, fungi and plants. (A) The *lrd*, *cld*, *rmn* and *lab* BGCs clusters in *Streptomyces*. [16, 20,33]. (B) The BGCs

containing the bifunctional terpene synthase PvCPS and PfCPS in *Penicillium*. [15]. (C) Proposed biosynthetic pathway of labdane-type bicyclic diterpenes catalyzed by *lrd*, *cld*, *rmn* and *lab* BGCs. [16,20,33]. (D) The reactions catalyzed by PvCPS and PfCPS. (E) Biosynthetic pathway of halimane and clerodane diterpenes in plants. [7,18].

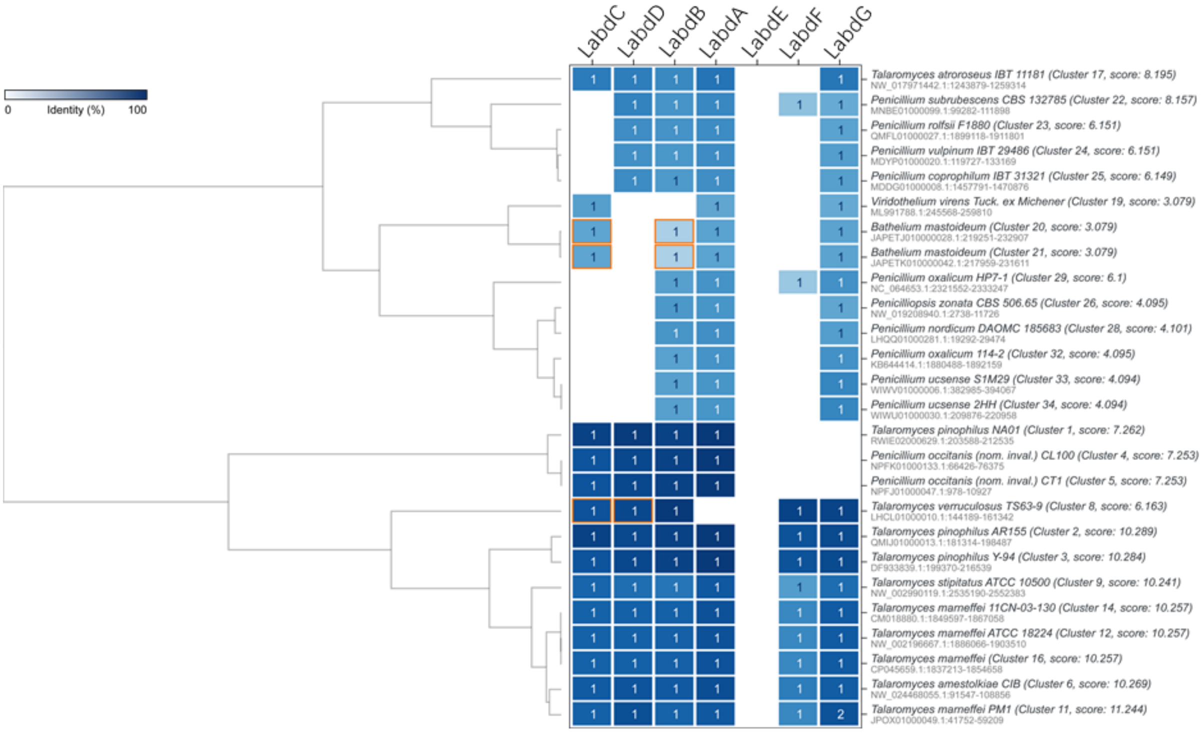

Figure S2. Cluster heatmap visualization of cblaster[9] search results using the *labd* cluster to query the fungal genomes from online database.

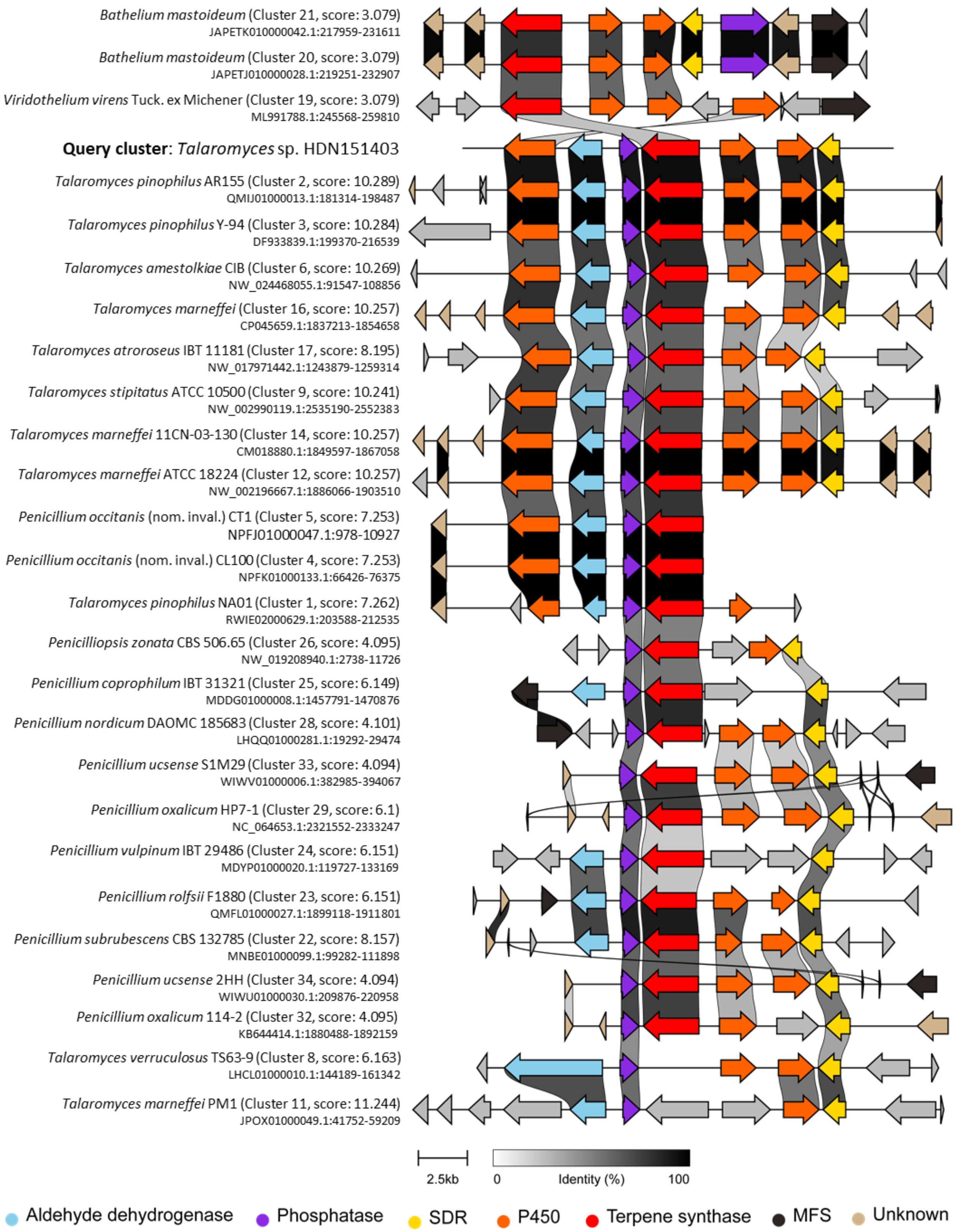

Figure S3. Visualization of gene clusters homologous to *labd* cluster searched by cblaster using clinker tool. [23].

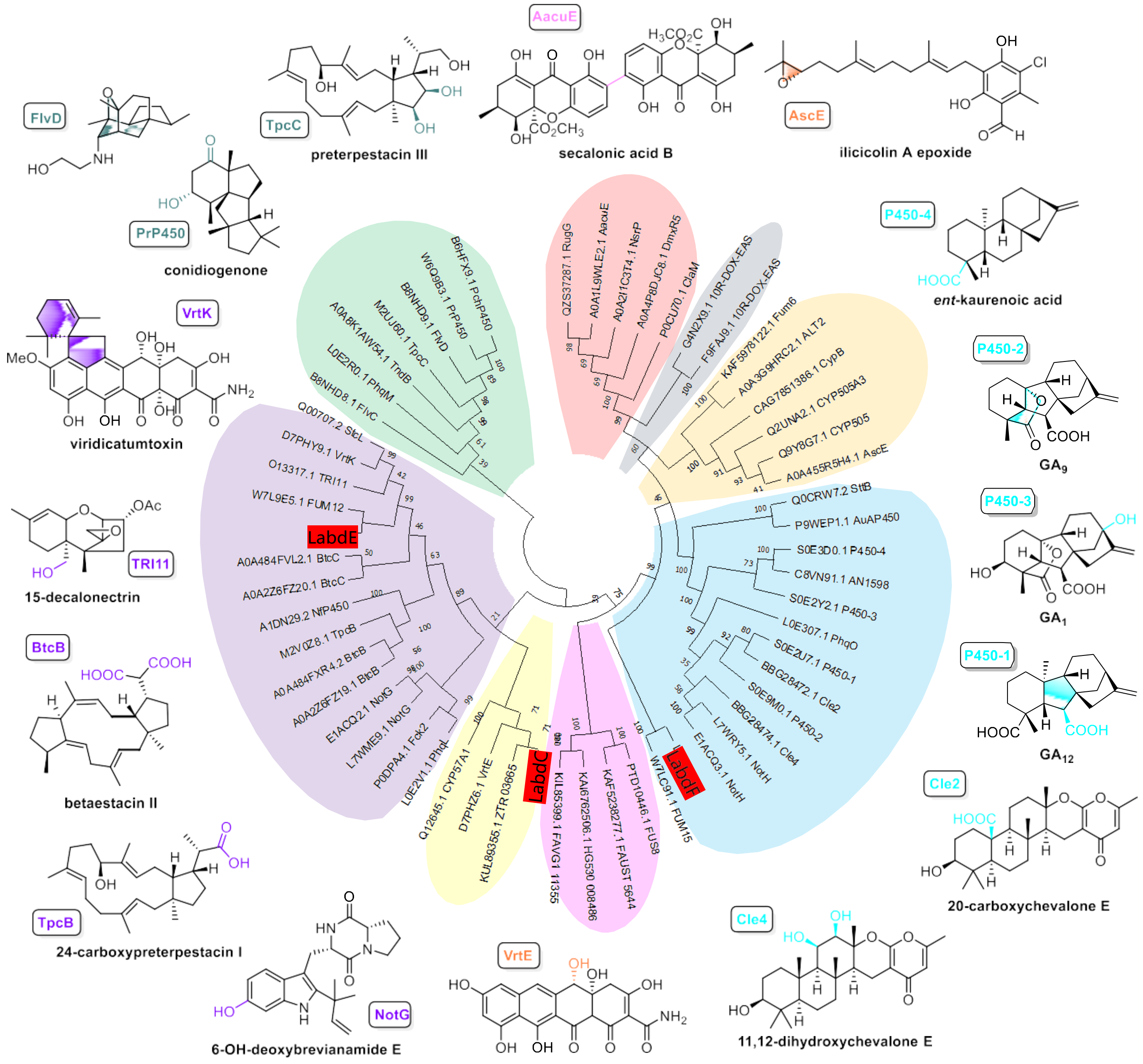

Figure S4. Neighbor joining method based phylogenetic analysis of P450s from Ascomycetes fungi using MEGA X software. The bootstrap consensus tree inferred from 1000 replicates is taken to represent the evolutionary history of the taxa analyzed. Protein names and accession numbers are shown at the leaves of the tree. The representative structures are listed in the figure, and the key catalytic sites of the relevant P450 enzymes are highlighted in different colors.

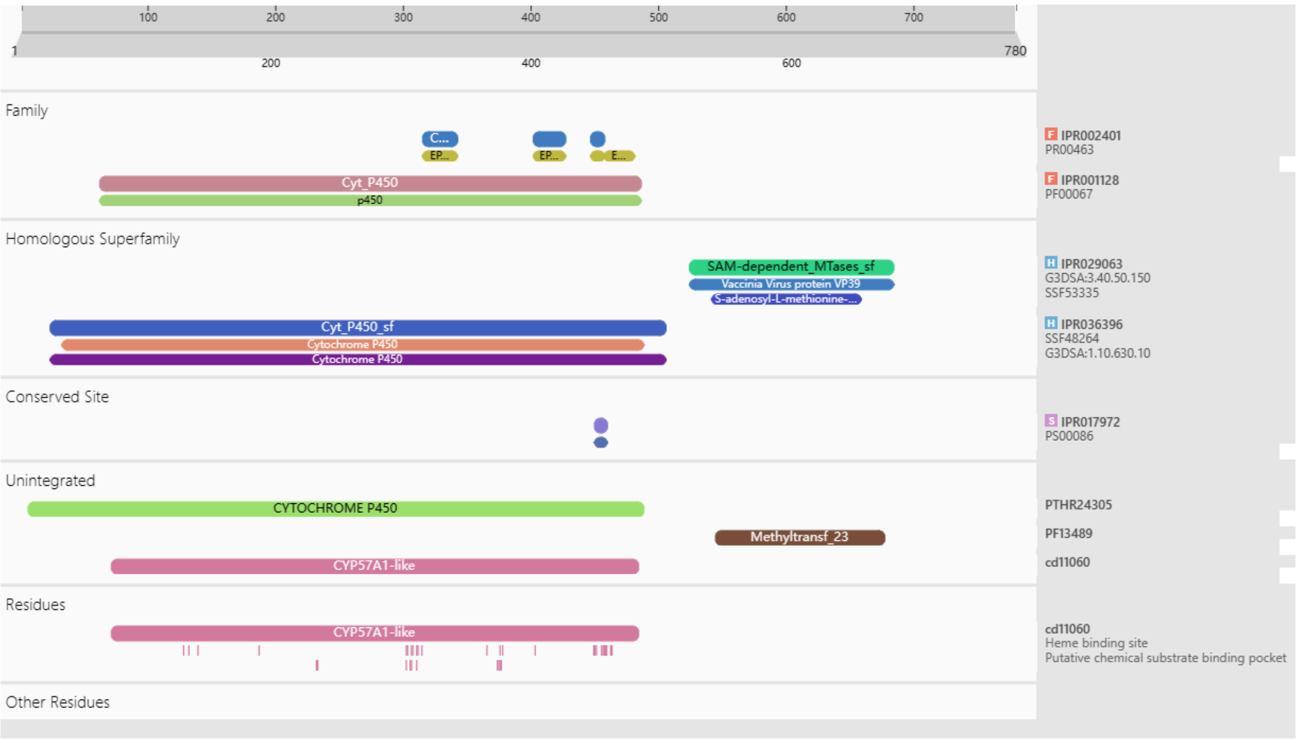

Figure S5. Results of protein family classification analysis of LabdC using InterPro database. The results showed that LabdC is a bifunctional P450 enzyme, including two functional domains of cytochrome P450 and methyltransferase.

Description	Scientific Name	Max Score	Total Score	Query Cover	E value	Per. Ident	Acc. Len	Accession
hypothetical protein ZTR_03665 [Talaromyces verruculosus]	Talaromyces verruculosus	1578	1578	100%	0.0	97.18%	1333	KUL89355.1
hypothetical protein TCE0_043r15581 [Talaromyces pinophilus]	Talaromyces pinophilus	1562	1562	100%	0.0	96.04%	782	GAM41998.1
Cytochrome P450 [Penicillium occitanis (nom. inval.)]	Penicillium occitanis (nom. inval.)	1561	1561	100%	0.0	95.78%	782	PCG99752.1
Cytochrome P450 monooxygenase vrtE [Talaromyces pinophilus]	Talaromyces pinophilus	1538	1538	100%	0.0	92.45%	808	KAF3385863.1
uncharacterized protein BHQ10_008362 [Talaromyces amestolkiae]	Talaromyces amestolkiae	1521	1521	100%	0.0	93.09%	782	XP_040736864.1
uncharacterized protein EYB26_009327 [Talaromyces marneffei]	Talaromyces marneffei	1447	1447	99%	0.0	89.74%	782	XP_054124552.1
benzoate 4-monooxygenase cytochrome P450, putative [Talaromyces stipitatus ATCC ...	Talaromyces stipitatus ATCC 10500	1444	1444	100%	0.0	87.47%	782	XP_002486679.1
hypothetical protein EIK77_007927 [Talaromyces pinophilus]	Talaromyces pinophilus	960	960	61%	0.0	91.49%	517	KAI7970747.1
hypothetical protein UA08_07234 [Talaromyces atroroseus]	Talaromyces atroroseus	776	1115	84%	0.0	80.17%	692	XP_020117855.1
cytochrome P450 [Viridothelium virens]	Viridothelium virens	650	650	62%	0.0	61.81%	720	KAF2235944.1
hypothetical protein M1822_007084 [Bathelium mastoideum]	Bathelium mastoideum	643	643	63%	0.0	60.45%	810	KAI9681732.1
hypothetical protein M1821_005805 [Bathelium mastoideum]	Bathelium mastoideum	642	642	63%	0.0	60.45%	810	KAI9654811.1
uncharacterized protein PgNI_02220 [Pyricularia grisea]	Pyricularia grisea	545	545	62%	0.0	49.80%	501	XP_030986481.1
hypothetical protein FGRMN_6520 [Fusarium graminum]	Fusarium graminum	545	545	60%	0.0	50.42%	523	KAF4993391.1
uncharacterized protein FTOL_07655 [Fusarium torulosum]	Fusarium torulosum	541	541	59%	0.0	49.89%	480	SPJ79264.1
uncharacterized protein PpBr36_10745 [Pyricularia pennisetigena]	Pyricularia pennisetigena	532	532	60%	9e-179	51.58%	501	XP_029743706.1
benzoate 4-monooxygenase cytochrome P450 [Pyricularia oryzae]	Pyricularia oryzae	527	527	59%	9e-177	50.53%	501	KAI7909201.1
benzoate 4-monooxygenase cytochrome P450 [Pyricularia oryzae 70-15]	Pyricularia oryzae 70-15	527	527	59%	1e-176	50.53%	508	XP_003720475.1
hypothetical protein MCOR01_010310 [Pyricularia oryzae]	Pyricularia oryzae	525	525	59%	6e-176	50.32%	501	KAH8838880.1
hypothetical protein PspLS_10363 [Pyricularia sp. CBS 133598]	Pyricularia sp. CBS 133598	468	468	59%	2e-154	47.77%	467	TLD18038.1
hypothetical protein F66182_12136 [Fusarium sp. NRRL 66182]	Fusarium sp. NRRL 66182	444	444	28%	9e-149	95.43%	219	KAF5016246.1
hypothetical protein M1820_009678 [Bogoriella megaspora]	Bogoriella megaspora	456	456	47%	8e-147	55.65%	646	KAI9691406.1
benzoate 4-monooxygenase cytochrome P450 [Hyaloscypha variabilis F]	Hyaloscypha variabilis F	384	384	59%	3e-121	40.94%	489	PMD45842.1
cytochrome P450 [Hyaloscypha hepaticicola]	Hyaloscypha hepaticicola	378	378	60%	9e-119	39.62%	495	PMD22400.1
hypothetical protein B0A52_00440 [Exophiala mesophila]	Exophiala mesophila	364	364	62%	3e-113	38.49%	506	RVX76083.1
hypothetical protein AYO20_04127 [Fonsecaea nubica]	Fonsecaea nubica	362	362	61%	2e-112	37.92%	519	XP_022501523.1
hypothetical protein B0A52_07104 [Exophiala mesophila]	Exophiala mesophila	353	353	61%	3e-109	36.76%	497	RVX68677.1
hypothetical protein FE257_012986 [Aspergillus nanangensis]	Aspergillus nanangensis	353	353	61%	6e-109	35.88%	513	KAF9885369.1
hypothetical protein A1O9_04703 [Exophiala aquamarina CBS 119918]	Exophiala aquamarina CBS 119918	351	351	62%	2e-108	36.73%	499	XP_013262445.1

Figure S6. Protein sequence alignment of LabdC with other homologs using Blastp (protein-protein BLAST).

Description	Scientific Name	Max Score	Total Score	Query Cover	E value	Per. Ident	Acc. Len	Accession
Isotrichodermin C-15 hydroxylase [Talaromyces pinophilus]	Talaromyces pinophilus	1040	1040	100%	0.0	99.80%	501	KAF3385867.1
hypothetical protein PENOC_085340 [Penicillium occitanis (nom. inval.)]	Penicillium occitanis (nom. inval.)	1038	1038	100%	0.0	99.40%	501	PCG93888.1
hypothetical protein ZTR_03649 [Talaromyces verruculosus]	Talaromyces verruculosus	1037	1037	100%	0.0	99.60%	501	KUL89333.1
uncharacterized protein BHQ10_008366 [Talaromyces amestolkiae]	Talaromyces amestolkiae	1031	1031	100%	0.0	98.60%	503	XP_040736868.1
uncharacterized protein EYB26_009323 [Talaromyces marneffei]	Talaromyces marneffei	996	996	100%	0.0	94.21%	501	XP_054124548.1
cytochrome P450, putative [Talaromyces stipitatus ATCC 10500]	Talaromyces stipitatus ATCC 10500	985	985	99%	0.0	94.79%	500	XP_002486675.1
hypothetical protein UA08_07230 [Talaromyces atroroseus]	Talaromyces atroroseus	942	942	100%	0.0	92.02%	501	XP_020117851.1
Isotrichodermin C-15 hydroxylase [Penicillium rolfsii]	Penicillium rolfsii	906	906	97%	0.0	87.12%	497	KAF3385030.1
Averantin hydroxylase [Penicillium vulpinum]	Penicillium vulpinum	902	902	98%	0.0	86.38%	497	XP_057110490.1
Averantin hydroxylase [Penicillium subrubescens]	Penicillium subrubescens	899	899	97%	0.0	86.91%	497	XP_057003734.1
Averantin hydroxylase [Penicillium oxalicum]	Penicillium oxalicum	899	899	98%	0.0	86.76%	497	XP_049969383.1
Averantin hydroxylase [Penicillium cataractarum]	Penicillium cataractarum	898	898	97%	0.0	86.71%	497	XP_056554907.1
hypothetical protein PDE_07206 [Penicillium oxalicum 114-2]	Penicillium oxalicum 114-2	898	898	98%	0.0	86.56%	497	EPS32246.1
Averantin hydroxylase [Penicillium tannophilum]	Penicillium tannophilum	897	897	97%	0.0	86.71%	497	KAJ5904104.1
Cytochrome P450 monooxygenase [Penicillium ucsense]	Penicillium ucsense	893	893	98%	0.0	85.98%	497	KAF7719414.1
Averantin hydroxylase [Penicillium odoratum]	Penicillium odoratum	893	893	98%	0.0	84.99%	504	XP_056993152.1
Averantin hydroxylase [Penicillium robsamsonii]	Penicillium robsamsonii	892	892	98%	0.0	85.57%	497	XP_057088019.1
Averantin hydroxylase [Penicillium glabrum]	Penicillium glabrum	892	892	97%	0.0	86.09%	497	KAJ5553235.1
Averantin hydroxylase [Penicillium diatomitis]	Penicillium diatomitis	891	891	98%	0.0	85.77%	497	XP_056791296.1
Averantin hydroxylase [Penicillium longicatenatum]	Penicillium longicatenatum	890	890	97%	0.0	87.09%	497	KAJ5664045.1
Averantin hydroxylase [Penicillium longicatenatum]	Penicillium longicatenatum	890	890	97%	0.0	87.09%	497	XP_056977175.1
Averantin hydroxylase [Penicillium concentricum]	Penicillium concentricum	889	889	98%	0.0	85.37%	497	XP_056582103.1
Averantin hydroxylase [Penicillium lividum]	Penicillium lividum	889	889	98%	0.0	84.58%	504	KAJ5627861.1
Averantin hydroxylase [Penicillium samsonianum]	Penicillium samsonianum	885	885	97%	0.0	85.10%	497	XP_057135627.1
hypothetical protein ASPZODRAFT_1503734 [Penicilliopsis zonata CBS 506.65]	Penicilliopsis zonata CBS 506.65	885	885	94%	0.0	87.26%	471	XP_022576702.1
Averantin hydroxylase [Penicillium verhagenii]	Penicillium verhagenii	884	884	96%	0.0	86.16%	497	XP_057016147.1
Averantin hydroxylase [Penicillium glabrum]	Penicillium glabrum	882	882	97%	0.0	84.88%	504	KAJ5554181.1
Averantin hydroxylase [Penicillium sp. IBT 16267x]	Penicillium sp. IBT 16267x	881	881	94%	0.0	87.74%	497	KAJ6109479.1
Averantin hydroxylase [Penicillium sp. IBT 35674x]	Penicillium sp. IBT 35674x	879	879	94%	0.0	87.13%	497	KAJ5987131.1
Averantin hydroxylase [Penicillium sp. IBT 35674x]	Penicillium sp. IBT 35674x	879	879	94%	0.0	87.13%	497	KAJ5987131.1
hypothetical protein ACN38_g11222 [Penicillium nordicum]	Penicillium nordicum	879	879	97%	0.0	84.29%	497	KOS37964.1
Averantin hydroxylase [Penicillium verrucosum]	Penicillium verrucosum	878	878	97%	0.0	84.29%	497	XP_057076769.1
Averantin hydroxylase [Penicillium verhagenii]	Penicillium verhagenii	877	877	96%	0.0	86.36%	497	KAJ5937840.1
Averantin hydroxylase [Penicillium angulare]	Penicillium angulare	874	874	96%	0.0	85.30%	498	KAJ5114237.1
hypothetical protein VN97_g8291 [Penicillium thymicola]	Penicillium thymicola	874	874	97%	0.0	84.29%	497	KAJ9485073.1
Averantin hydroxylase [Penicillium pulvis]	Penicillium pulvis	801	801	87%	0.0	86.10%	468	XP_056924516.1
hypothetical protein PENCOP_c008G02740 [Penicillium coprophilum]	Penicillium coprophilum	786	786	84%	0.0	87.91%	446	OQE38664.1
putative cytochrome P450 [Viridothelium virens]	Viridothelium virens	755	755	92%	0.0	76.61%	505	KAF2235947.1
hypothetical protein M1820_009681 [Bogoriella megaspora]	Bogoriella megaspora	753	753	98%	0.0	72.76%	505	KAI9691409.1
hypothetical protein M1821_005808 [Bathelium mastoideum]	Bathelium mastoideum	746	746	93%	0.0	75.11%	498	KAI9654814.1
hypothetical protein M1822_007087 [Bathelium mastoideum]	Bathelium mastoideum	745	745	93%	0.0	75.11%	498	KAI9681735.1
Isotrichodermin C-15 hydroxylase [Penicillium subrubescens]	Penicillium subrubescens	695	695	76%	0.0	86.20%	402	OKP14338.1
hypothetical protein EIK77_007931 [Talaromyces pinophilus]	Talaromyces pinophilus	686	686	66%	0.0	97.94%	359	KAI7970751.1
uncharacterized protein N7510_007632 [Penicillium lagena]	Penicillium lagena	530	530	98%	0.0	53.55%	498	XP_056832600.1
Cytochrome p450 protein [Lasiodiplodia theobromae]	Lasiodiplodia theobromae	525	525	96%	3e-180	52.47%	491	XP_035367558.1
Cytochrome P450 monooxygenase hmp1 [Lasiodiplodia hormozganensis]	Lasiodiplodia hormozganensis	524	524	96%	1e-179	52.36%	491	KAK0647576.1
Cytochrome p450 protein [Neofusicoccum parvum]	Neofusicoccum parvum	516	516	94%	3e-176	51.26%	529	GME59351.1
putative cytochrome p450 protein [Neofusicoccum parvum UCRNP2]	Neofusicoccum parvum UCRNP2	515	515	94%	1e-175	51.26%	529	EOD48095.1
Cytochrome p450 protein [Neofusicoccum parvum]	Neofusicoccum parvum	513	513	94%	5e-175	51.05%	529	GME34285.1
putative cytochrome P450 [Pestalotiopsis sp. NC0098]	Pestalotiopsis sp. NC0098	508	508	92%	1e-173	51.84%	488	KAI0146810.1
putative cytochrome P450 [Corynespora cassiicola Philippines]	Corynespora cassiicola Philippines	505	505	97%	2e-172	51.53%	490	PSN63043.1
hypothetical protein PFICI_06539 [Pestalotiopsis fici W106-1]	Pestalotiopsis fici W106-1	503	503	93%	2e-171	51.17%	490	XP_007833311.1
Cytochrome p450 protein [Lasiodiplodia theobromae]	Lasiodiplodia theobromae	502	502	96%	2e-171	49.38%	476	XP_035368502.1
Heterokaryon incompatibility [Botryosphaeria dothidea]	Botryosphaeria dothidea	493	493	93%	2e-167	51.06%	499	KAF4300746.1
hypothetical protein PFICI_06539 [Neofusicoccum parvum]	Neofusicoccum parvum	492	492	93%	3e-167	49.89%	494	GME59698.1
hypothetical protein S40288_03084 [Stachybotrys chartarum IBT 40288]	Stachybotrys chartarum IBT 40288	493	493	92%	3e-167	50.64%	500	KFA77429.1
hypothetical protein S7711_03465 [Stachybotrys chartarum IBT 7711]	Stachybotrys chartarum IBT 7711	491	491	92%	8e-167	50.43%	500	KEY64172.1
Isotrichodermin C-15 hydroxylase [Diplodia seriata]	Diplodia seriata	488	488	96%	9e-166	49.48%	491	OMP83594.1

Figure S7. Protein sequence alignment of LabdE with other homologs using Blastp (protein-protein BLAST).

Description	Scientific Name	Max Score	Total Score	Query Cover	E value	Per. Ident	Acc. Len	Accession
hypothetical protein ZTR_03650 [Talaromyces verruculosus]	Talaromyces verruculosus	1086	1086	100%	0.0	97.23%	542	KUL89225.1
hypothetical protein PENOC_085350 [Penicillium occitanis (nom. inval.)]	Penicillium occitanis (nom. inval.)	1068	1068	100%	0.0	95.39%	542	PCG93889.1
hypothetical protein TCE0_043f15589 [Talaromyces pinophilus]	Talaromyces pinophilus	1060	1060	100%	0.0	95.20%	540	GAM42003.1
uncharacterized protein BHQ10_008367 [Talaromyces amestolkiae]	Talaromyces amestolkiae	1021	1021	100%	0.0	90.28%	545	XP_040736869.1
uncharacterized protein EYB26_009322 [Talaromyces marneffei]	Talaromyces marneffei	1005	1005	100%	0.0	88.56%	542	XP_054124547.1
cytochrome P450, putative [Talaromyces stipitatus ATCC 10500]	Talaromyces stipitatus ATCC 10500	929	929	100%	0.0	82.87%	543	XP_002486674.1
hypothetical protein UA08_07229 [Talaromyces atroroseus]	Talaromyces atroroseus	831	831	94%	0.0	77.93%	518	XP_020117852.1
Cytochrome P450 3A12 [Talaromyces pinophilus]	Talaromyces pinophilus	802	802	77%	0.0	93.57%	422	KAF3385868.1
hypothetical protein N7451_011497 [Penicillium sp. IBT 35674x]	Penicillium sp. IBT 35674x	751	751	99%	0.0	67.96%	534	KAJ5987132.1
hypothetical protein N7486_001713 [Penicillium sp. IBT 16267x]	Penicillium sp. IBT 16267x	751	751	99%	0.0	67.47%	534	KAJ6109478.1
hypothetical protein N7494_002614 [Penicillium glabrum]	Penicillium glabrum	749	749	99%	0.0	67.28%	534	KAJ5553236.1
hypothetical protein N7504_006488 [Penicillium tannophilum]	Penicillium tannophilum	749	749	99%	0.0	67.65%	544	KAJ5904105.1
uncharacterized protein N7503_004055 [Penicillium pulvis]	Penicillium pulvis	748	748	99%	0.0	67.46%	534	XP_056924517.1
uncharacterized protein N7520_009929 [Penicillium odoratum]	Penicillium odoratum	748	748	99%	0.0	67.83%	537	XP_056993151.1
hypothetical protein N7513_004139 [Penicillium glabrum]	Penicillium glabrum	748	748	99%	0.0	67.10%	534	KAJ5554180.1
hypothetical protein N7454_004183 [Penicillium verhagenii]	Penicillium verhagenii	748	748	99%	0.0	67.78%	536	KAJ5937841.1
uncharacterized protein N7496_006566 [Penicillium cataractarum]	Penicillium cataractarum	743	743	99%	0.0	67.03%	535	XP_056554908.1
hypothetical protein N7490_010090 [Penicillium lividum]	Penicillium lividum	739	739	99%	0.0	68.26%	537	KAJ5627862.1
uncharacterized protein N7473_011946 [Penicillium subrubescens]	Penicillium subrubescens	739	739	99%	0.0	66.98%	535	XP_057003735.1
uncharacterized protein N7471_008649 [Penicillium samsonianum]	Penicillium samsonianum	738	738	99%	0.0	67.53%	540	XP_057135628.1
uncharacterized protein N7479_003469 [Penicillium vulpinum]	Penicillium vulpinum	738	738	99%	0.0	67.89%	540	XP_057110491.1
uncharacterized protein N7517_000237 [Penicillium concentricum]	Penicillium concentricum	732	732	99%	0.0	67.71%	540	XP_056582102.1
Cytochrome P450 monooxygenase [Penicillium ucsense]	Penicillium ucsense	731	731	99%	0.0	66.61%	539	KAF7719415.1
Cytochrome P450 3A13 [Penicillium subrubescens]	Penicillium subrubescens	728	728	99%	0.0	64.58%	553	OKP14337.1
hypothetical protein POX_d05589 [Penicillium oxalicum]	Penicillium oxalicum	727	727	98%	0.0	66.85%	535	XP_049969382.1
uncharacterized protein N7516_002167 [Penicillium verrucosum]	Penicillium verrucosum	726	726	99%	0.0	66.91%	540	XP_057076770.1
uncharacterized protein ASPVEDRAFT_138421 [Aspergillus versicolor CBS 583.65]	Aspergillus versicolor CBS 583.65	725	725	99%	0.0	65.99%	541	XP_040671551.1
hypothetical protein N7507_004777 [Penicillium longicatenatum]	Penicillium longicatenatum	724	724	90%	0.0	70.06%	508	KAJ5664046.1
uncharacterized protein N7447_004402 [Penicillium robsamsonii]	Penicillium robsamsonii	720	720	99%	0.0	67.53%	540	XP_057088018.1
uncharacterized protein N7447_004402 [Penicillium robsamsonii]	Penicillium robsamsonii	720	720	99%	0.0	67.53%	540	XP_057088018.1
hypothetical protein PDE_07207 [Penicillium oxalicum 114-2]	Penicillium oxalicum 114-2	716	716	92%	0.0	69.50%	506	EPS32247.1
hypothetical protein N7456_002772 [Penicillium angulare]	Penicillium angulare	700	700	98%	0.0	67.22%	537	KAJ5114238.1
uncharacterized protein N7539_004152 [Penicillium diatomitis]	Penicillium diatomitis	691	691	88%	0.0	70.15%	494	XP_056791295.1
uncharacterized protein N7466_011027 [Penicillium verhagenii]	Penicillium verhagenii	668	668	89%	0.0	66.74%	494	XP_057016148.1
hypothetical protein ACN38_g11223 [Penicillium nordicum]	Penicillium nordicum	646	646	89%	0.0	65.92%	489	KOS37976.1
hypothetical protein VN97_g8292 [Penicillium thymicola]	Penicillium thymicola	642	642	89%	0.0	65.78%	489	KAJ9485072.1
hypothetical protein EIK77_007932 [Talaromyces pinophilus]	Talaromyces pinophilus	638	638	59%	0.0	95.65%	336	KAI7970752.1
hypothetical protein ASPZODRAFT_77483 [Penicilliopsis zonata CBS 506.65]	Penicilliopsis zonata CBS 506.65	634	634	92%	0.0	61.90%	514	XP_022576701.1
putative cytochrome P450 [Viridothelium virens]	Viridothelium virens	580	580	91%	0.0	58.37%	496	KAF2235946.1
cytochrome p450 [Lasallia pustulata]	Lasallia pustulata	568	568	96%	0.0	51.87%	543	SLM39940.1
cytochrome P450 [Lasallia pustulata]	Lasallia pustulata	565	565	96%	0.0	51.50%	543	KAA6413515.1
hypothetical protein [Hypocenomyce scalaris]	Hypocenomyce scalaris	555	555	97%	0.0	52.04%	545	MCJ1304961.1
hypothetical protein LQ347_000766 [Umbilicaria vellea]	Umbilicaria vellea	555	555	93%	0.0	52.05%	545	KAI4135313.1
hypothetical protein M1821_005807 [Bathelium mastoideum]	Bathelium mastoideum	552	552	86%	0.0	57.69%	463	KAI9654813.1
hypothetical protein M1820_009680 [Bogoriella megaspora]	Bogoriella megaspora	550	550	86%	0.0	56.84%	466	KAI9691408.1
hypothetical protein [Lignoscripta atroalba]	Lignoscripta atroalba	545	545	96%	0.0	50.18%	548	MCJ1254213.1
hypothetical protein [Acarospora aff. strigata]	Acarospora aff. strigata	541	541	99%	0.0	49.18%	547	MCJ1362699.1
cytochrome P450 [Saccharata proteae CBS 121410]	Saccharata proteae CBS 121410	538	538	93%	0.0	51.76%	542	KAF2092013.1
hypothetical protein B0A49_00372 [Cryomyces minteri]	Cryomyces minteri	537	537	99%	0.0	51.38%	541	TKA81886.1
uncharacterized protein HO173_001655 [Letharia columbiana]	Letharia columbiana	536	536	97%	0.0	49.81%	546	XP_037169314.1
hypothetical protein [Lobaria immixta]	Lobaria immixta	535	535	98%	0.0	47.91%	547	MCJ1266096.1
hypothetical protein [Xylographa bjoerkii]	Xylographa bjoerkii	535	535	91%	0.0	50.79%	548	MCJ1387575.1
hypothetical protein [Ptychographa xylographoides]	Ptychographa xylographoides	533	533	98%	0.0	49.36%	548	MCJ1406239.1
hypothetical protein [Xylographa carneopallida]	Xylographa carneopallida	533	533	96%	0.0	48.89%	548	MCJ1295844.1
hypothetical protein L6R40_003711 [Xanthomendoza cf. fulva]	Xanthomendoza cf. fulva	532	532	95%	0.0	50.19%	547	KAI4243020.1
Cytochrome P450 3A13 [Penicillium rolfsii]	Penicillium rolfsii	528	528	69%	0.0	67.09%	392	KAF3385029.1
hypothetical protein [Xylographa vitiligo]	Xylographa vitiligo	530	530	95%	1e-180	48.69%	548	MCJ1316429.1
hypothetical protein M1833_006363 [Piccolia ochrophora]	Piccolia ochrophora	529	529	91%	3e-180	51.29%	544	KAI9796358.1
uncharacterized protein HO133_007892 [Letharia lupina]	Letharia lupina	528	528	97%	4e-180	49.26%	546	XP_037156096.1

Figure S8. Protein sequence alignment of LabdF with other homologs using Blastp (protein-protein BLAST).

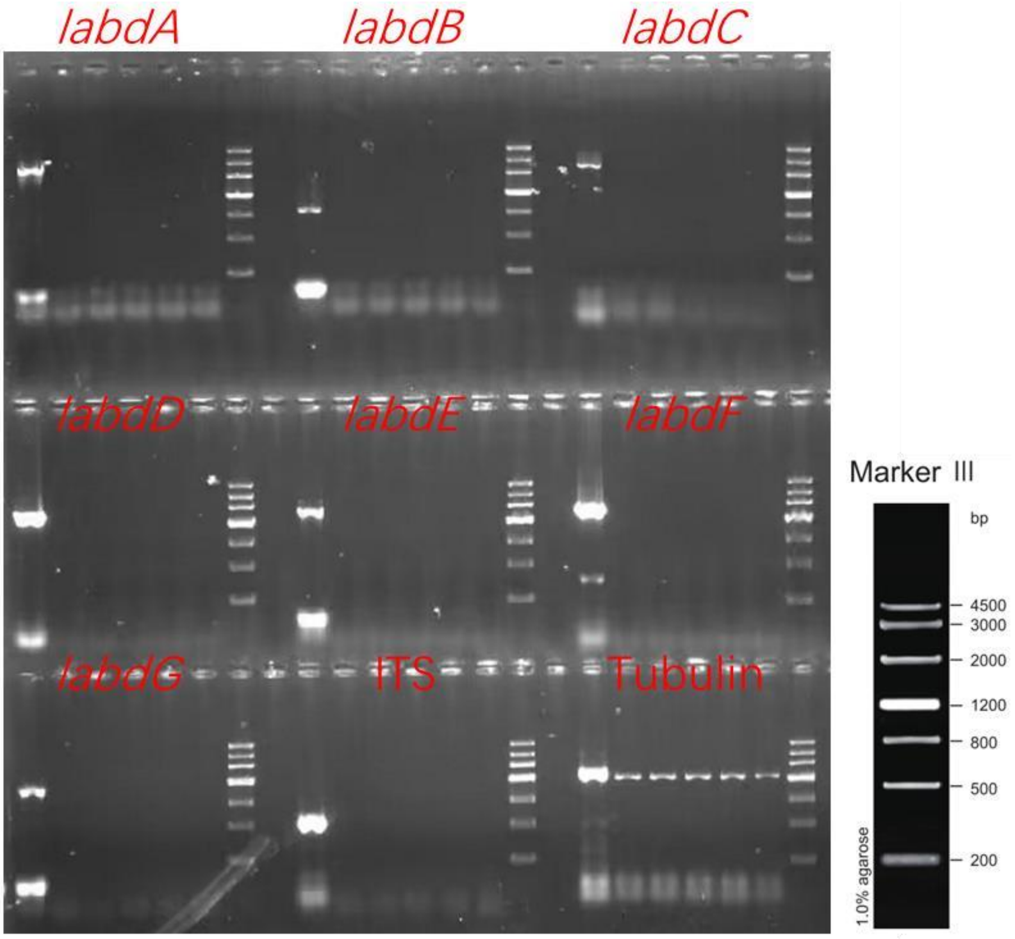

Figure S9. RT-PCR results of *labd* cluster of *Talaromyces* sp. HDN 151403 under different laboratory culture conditions. The PCR templates from left to right are: gDNA of *Talaromyces* sp. HDN 151403, cDNA of *Talaromyces* sp. HDN 151403 from five different media: PDB, fungal modified No. 2 medium, rice medium, glycerol medium, and trypticase (tryptic) soy broth.

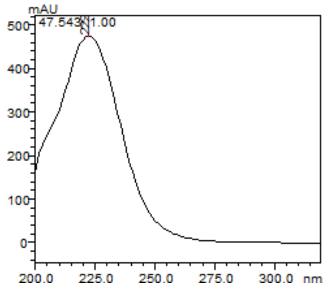

1

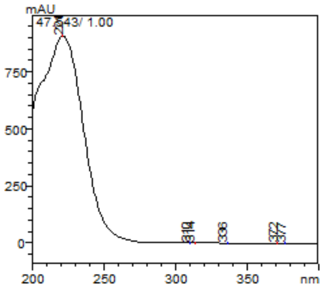

2

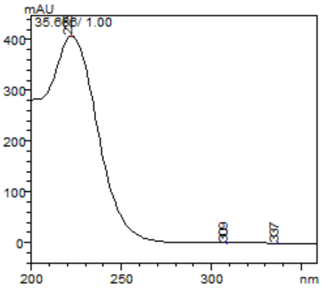

3

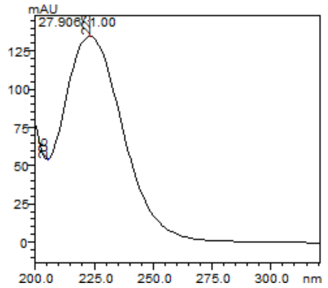

4

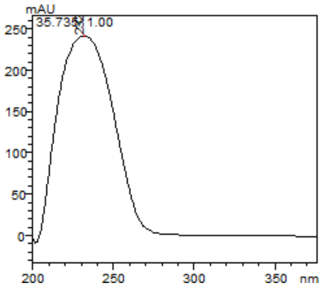

5

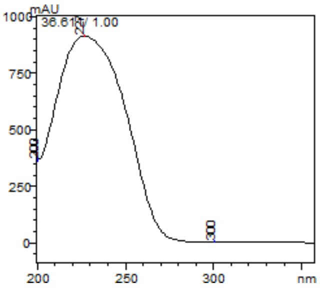

6

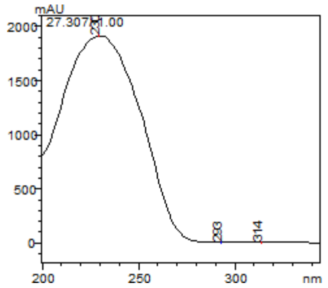

7

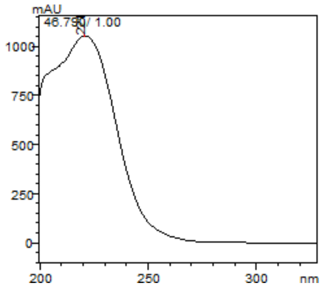

8

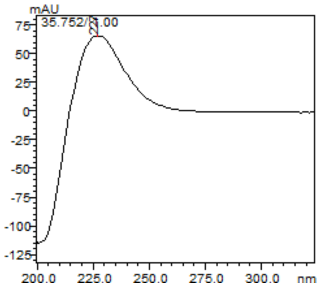

9

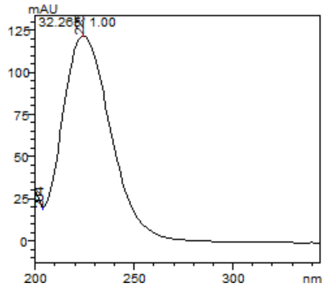

10

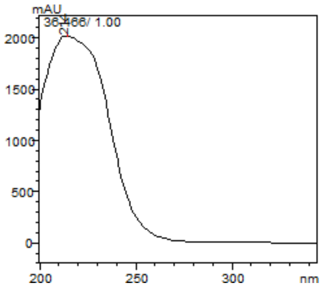

11

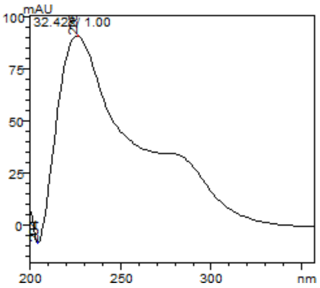

12

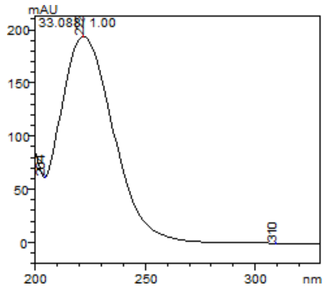

13

Figure S10. UV-vis spectra of purified compounds. All spectra were measured with a Shimadzu UFLC system equipped with a variable wavelength detector.

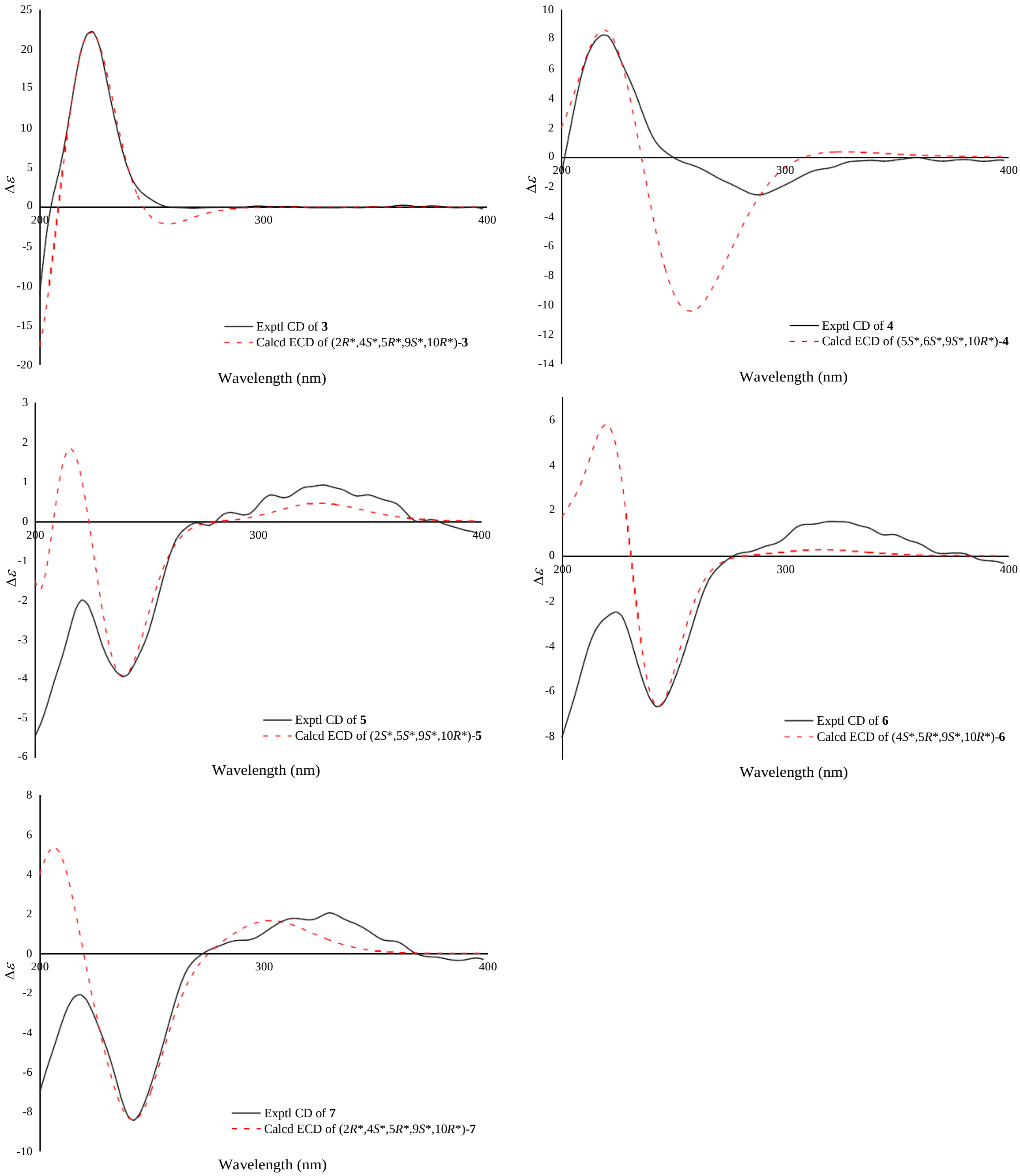

Figure S11. Comparison of calculated and experimental ECD spectra of **3–7** in methanol.

Figure S12. ^{1}H NMR of **3** in CD_3OD.

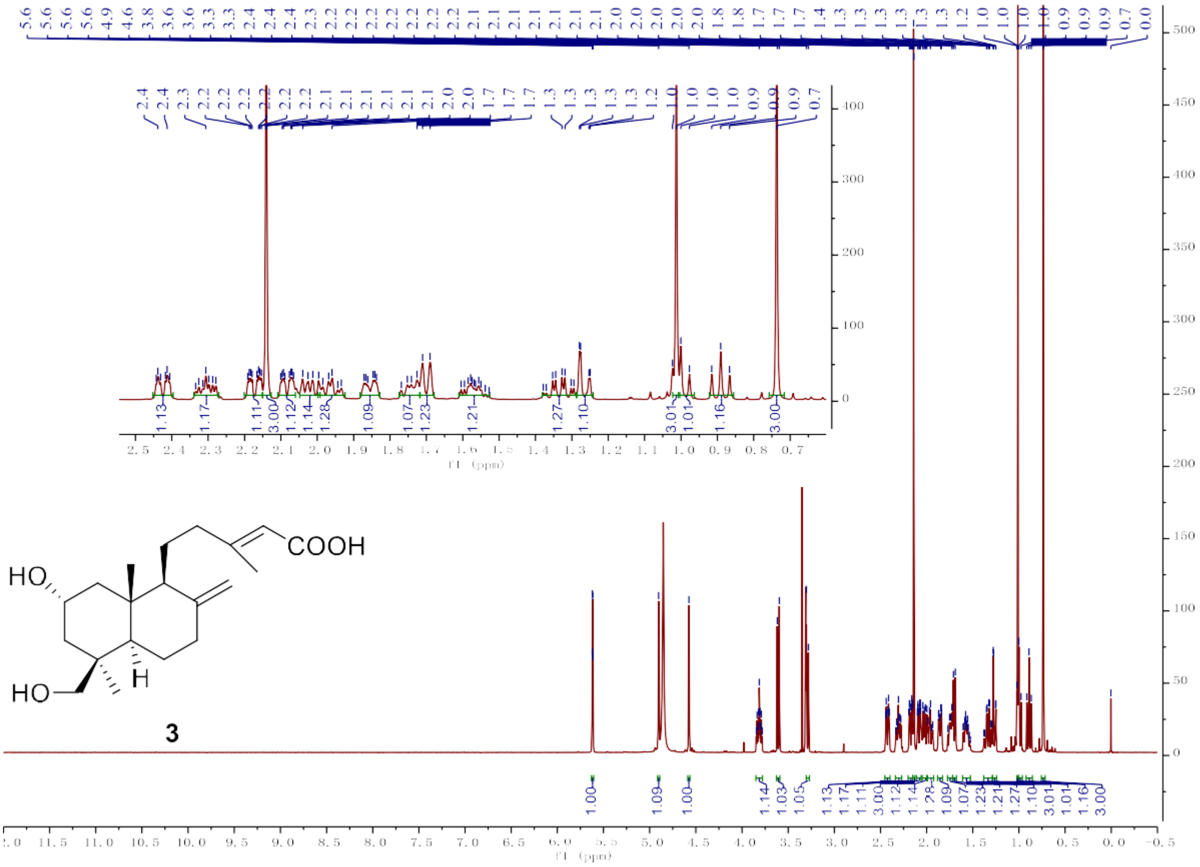

Figure S13. ^{13}C NMR of **3** in CD_3OD.

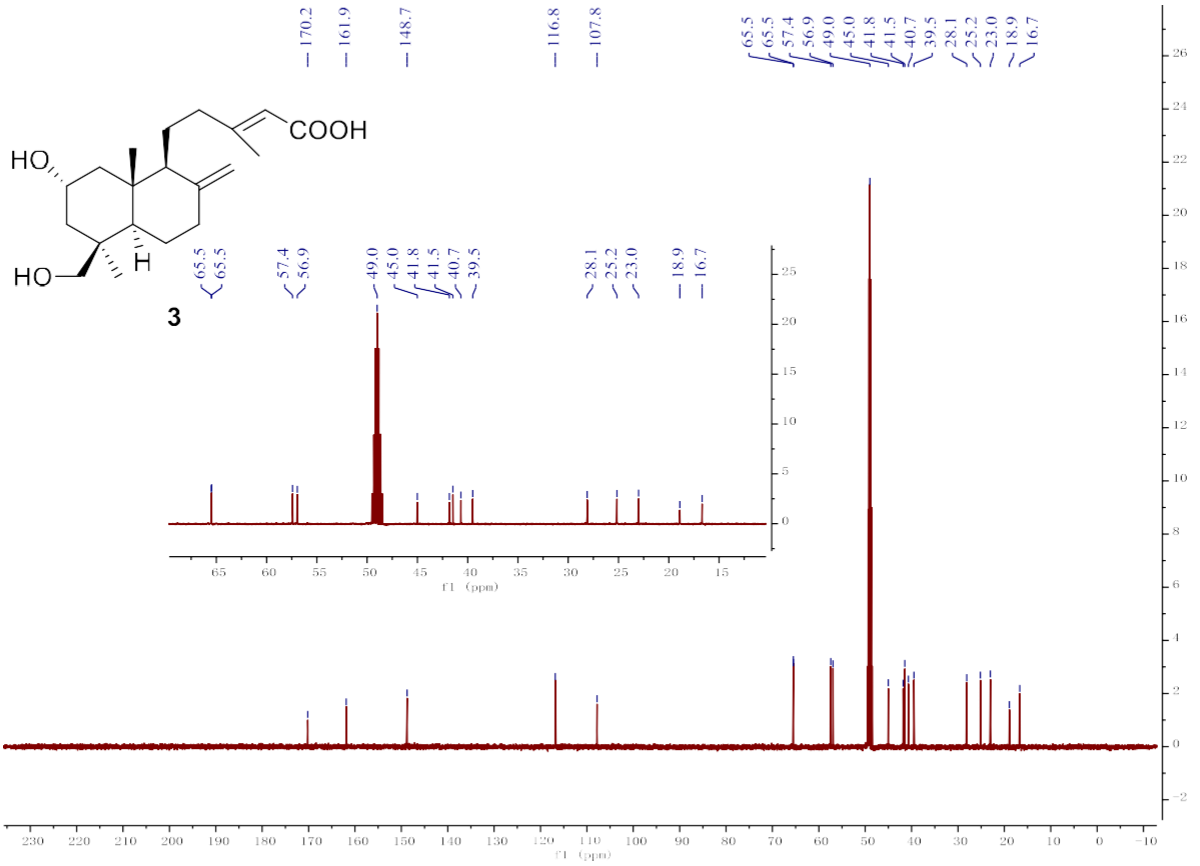

Figure S14. HSQC of **3** in CD_3OD.

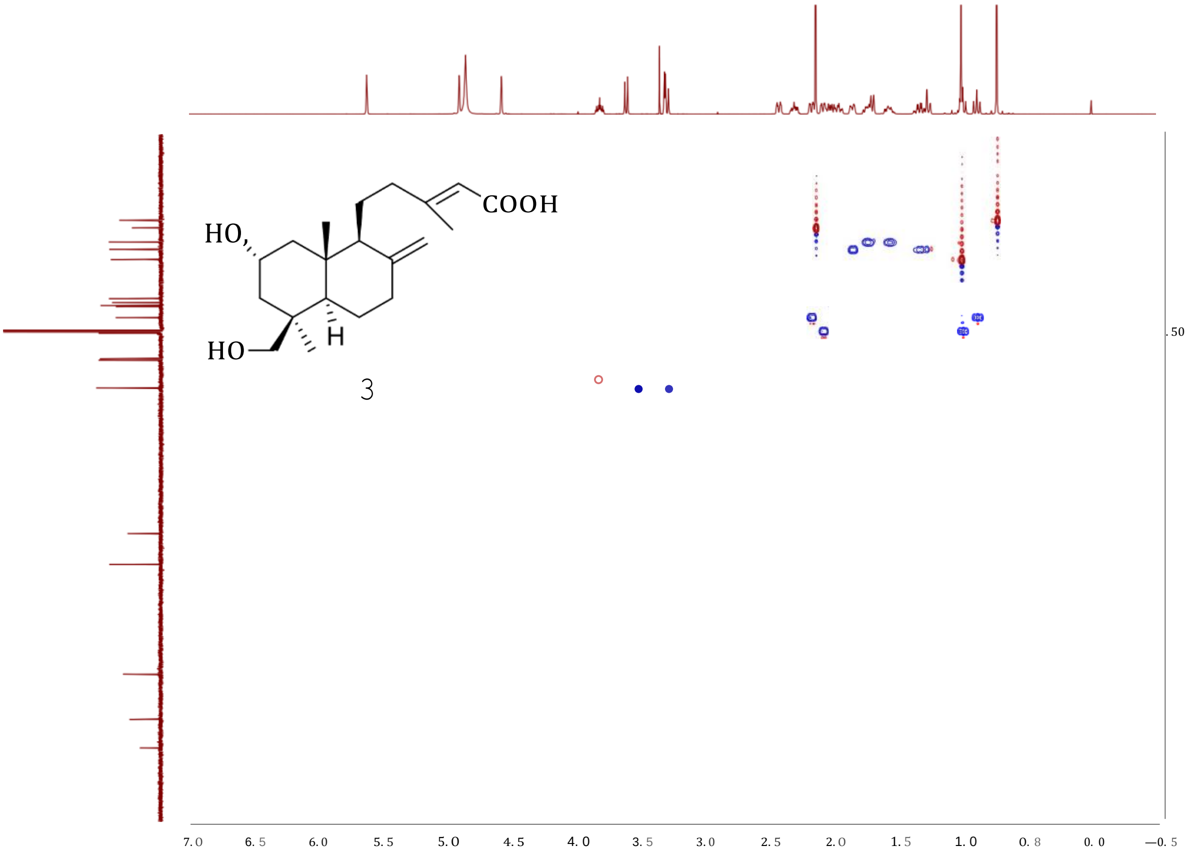

Figure S15. HMBC of **3** in CD_3OD.

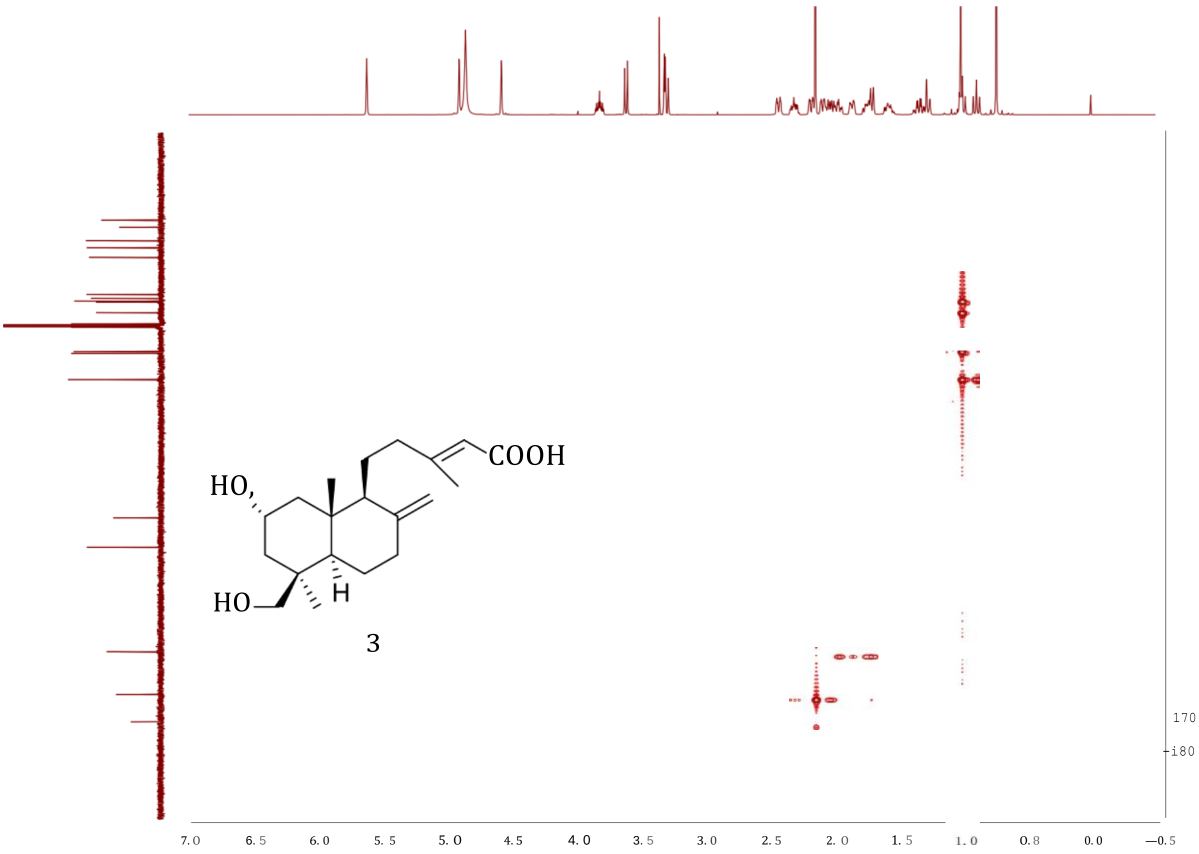

Figure S16. ^{1}H-^{1}H COSY of **3** in CD_3OD.

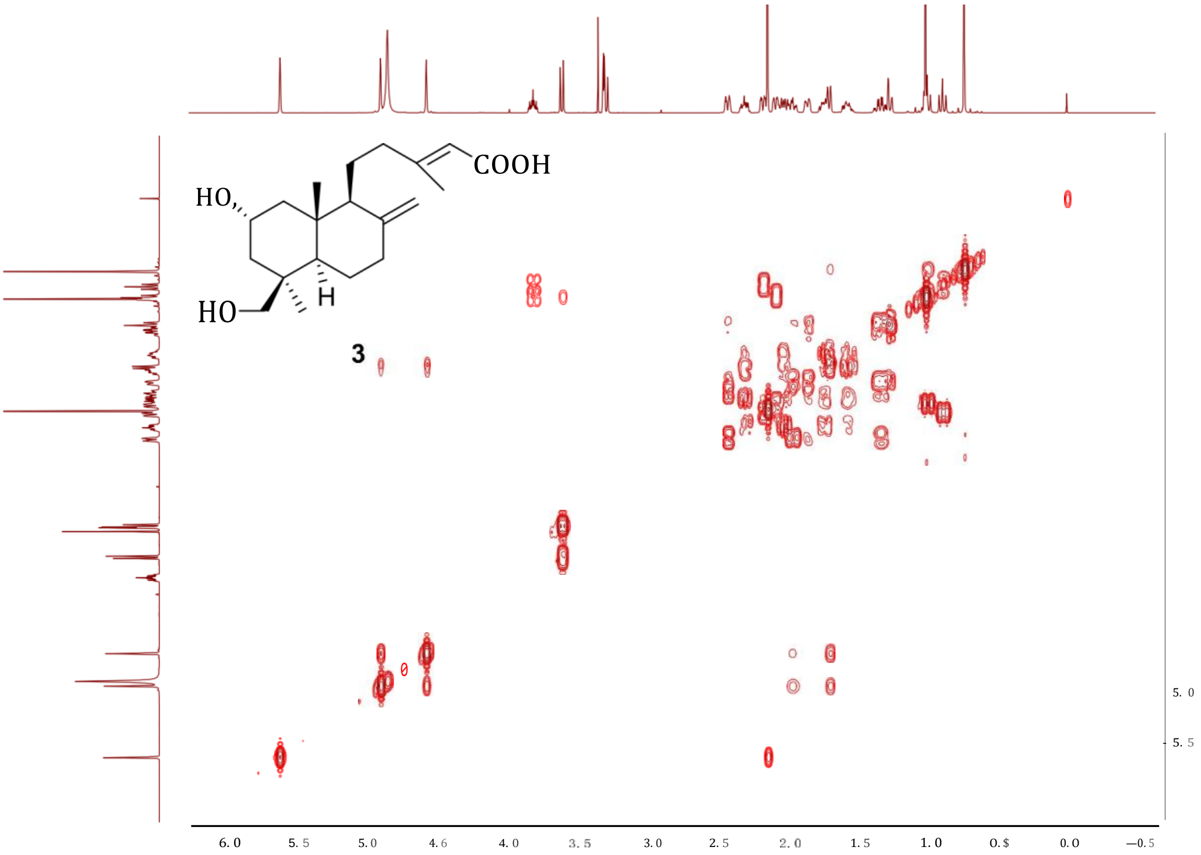

Figure S17. ROESY of **3** in CD_3OD.

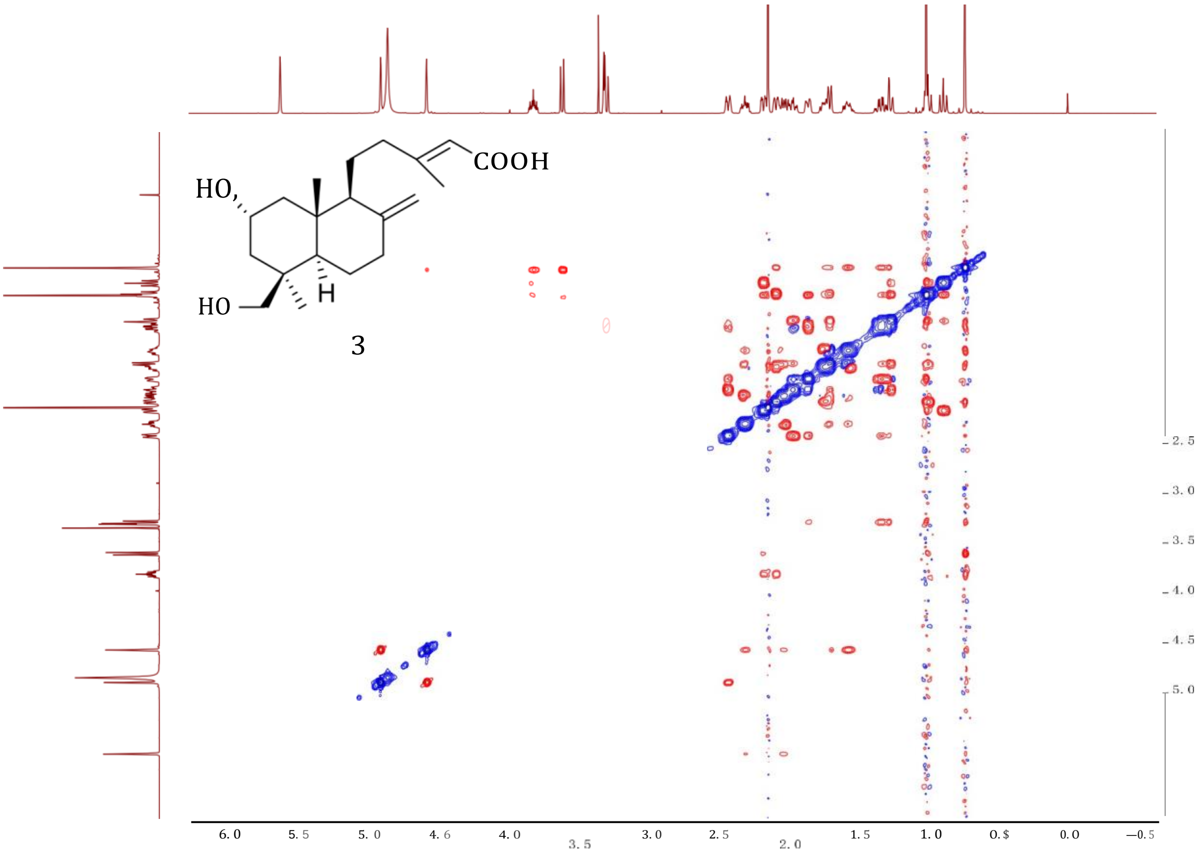

Figure S18. HRESIMS of **3**.

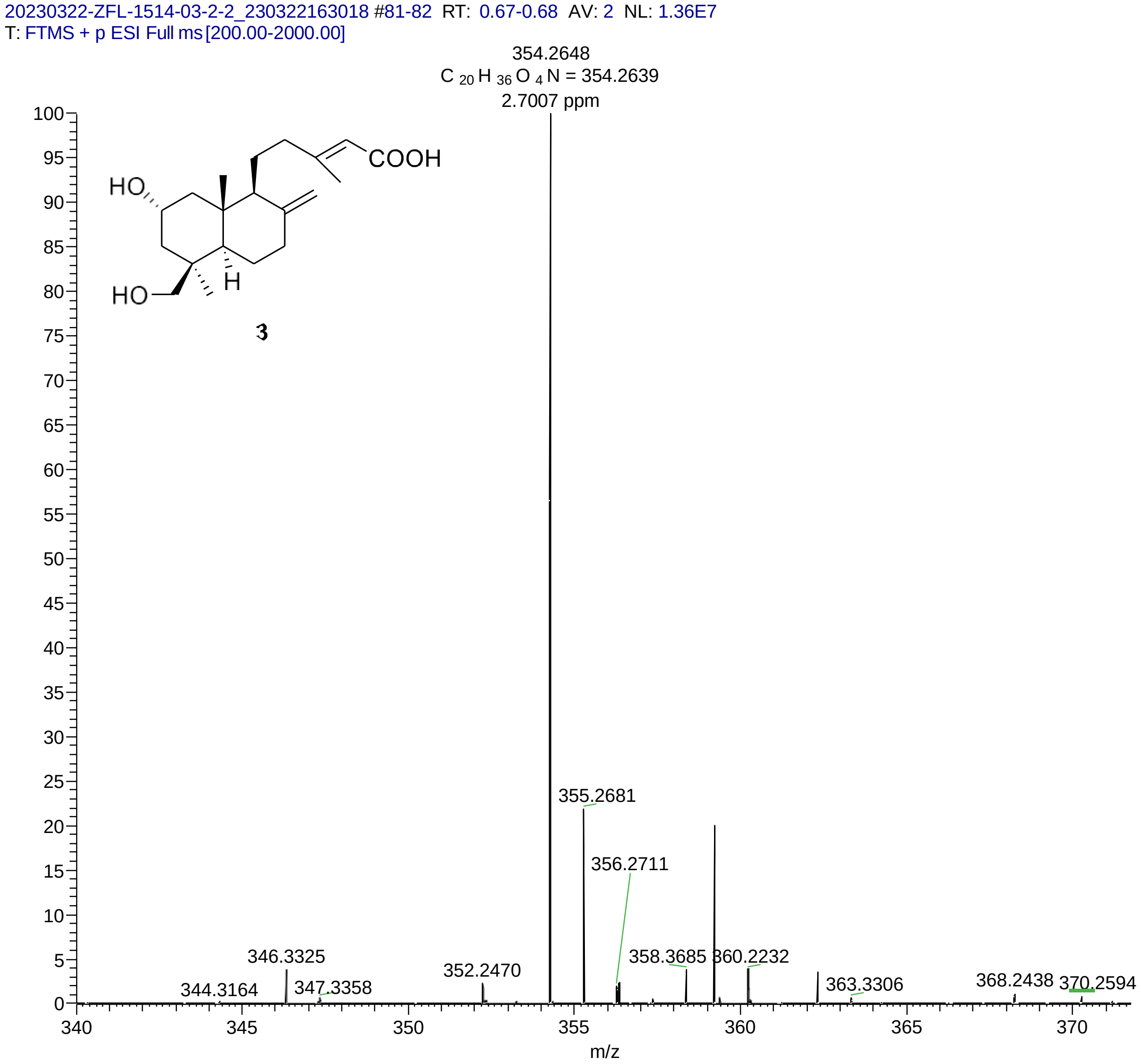

Figure S19. UV spectrum of **3**.

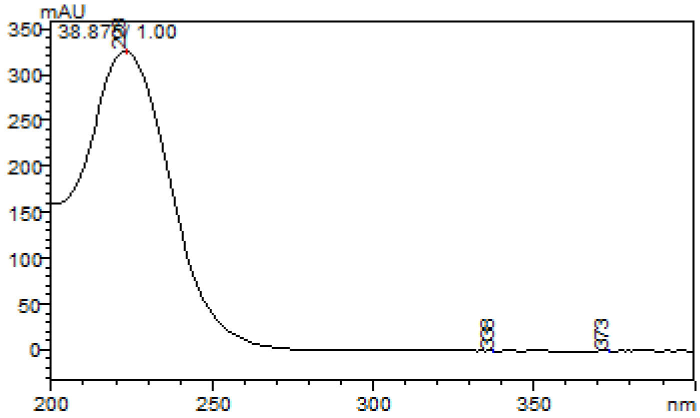

Figure S20. ^{1}H NMR of **4** in CD_3OD.

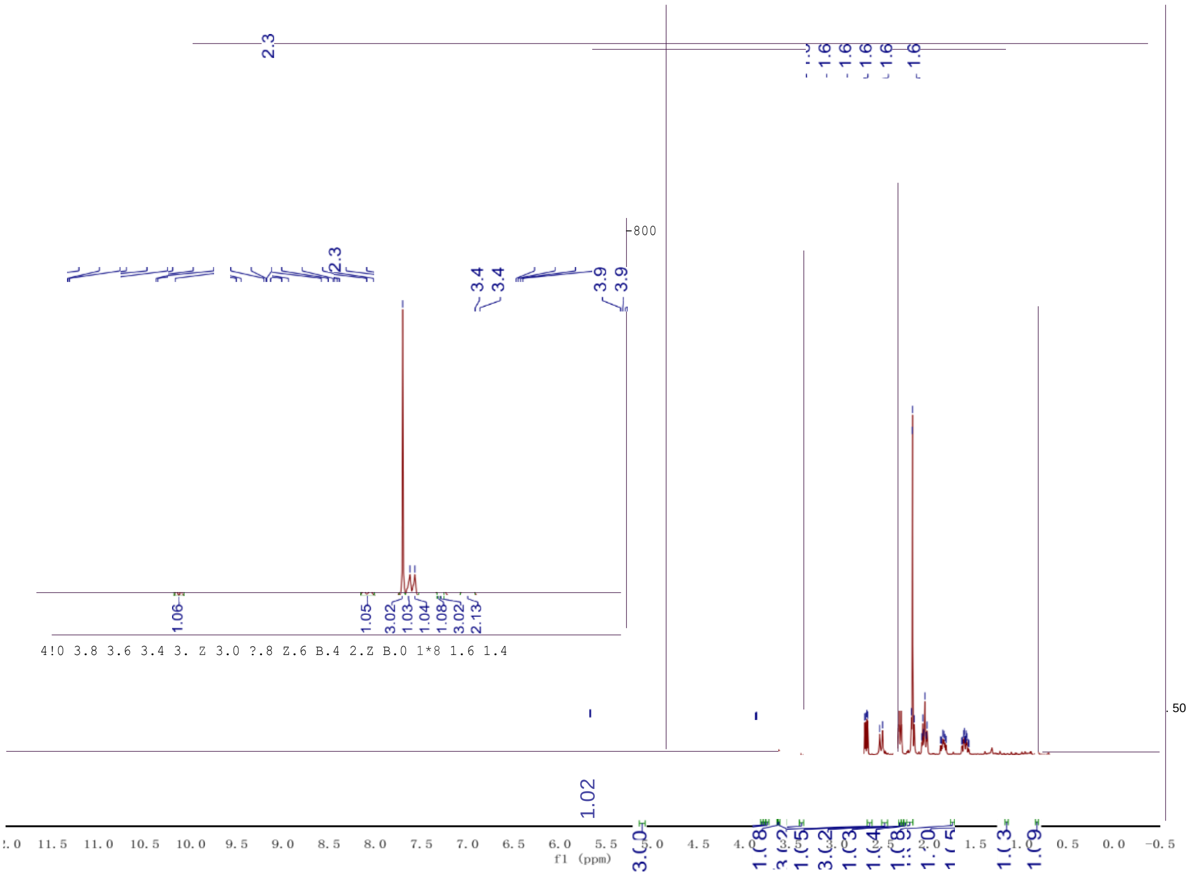

Figure S21. ^{13}C NMR and DEPT of **4** in CD_3OD.

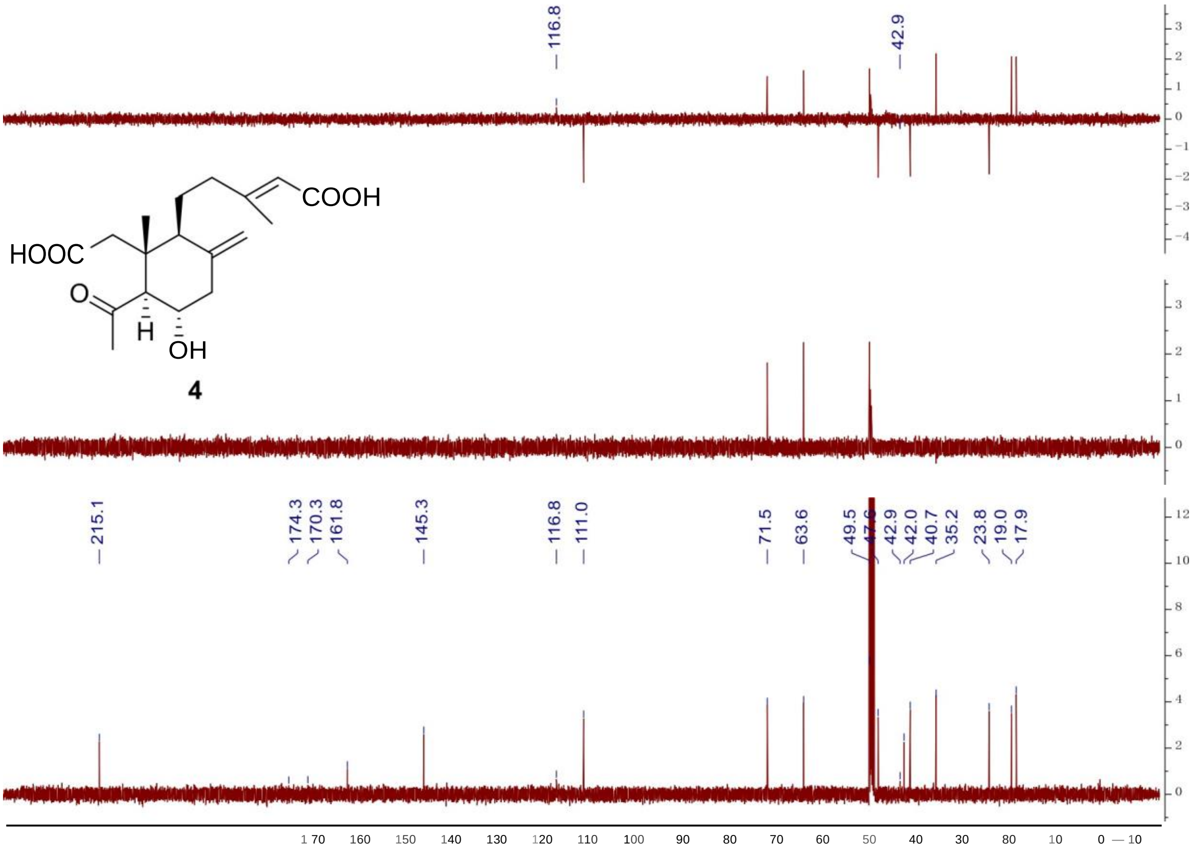

Figure S22. HSQC of **4** in CD_3OD.

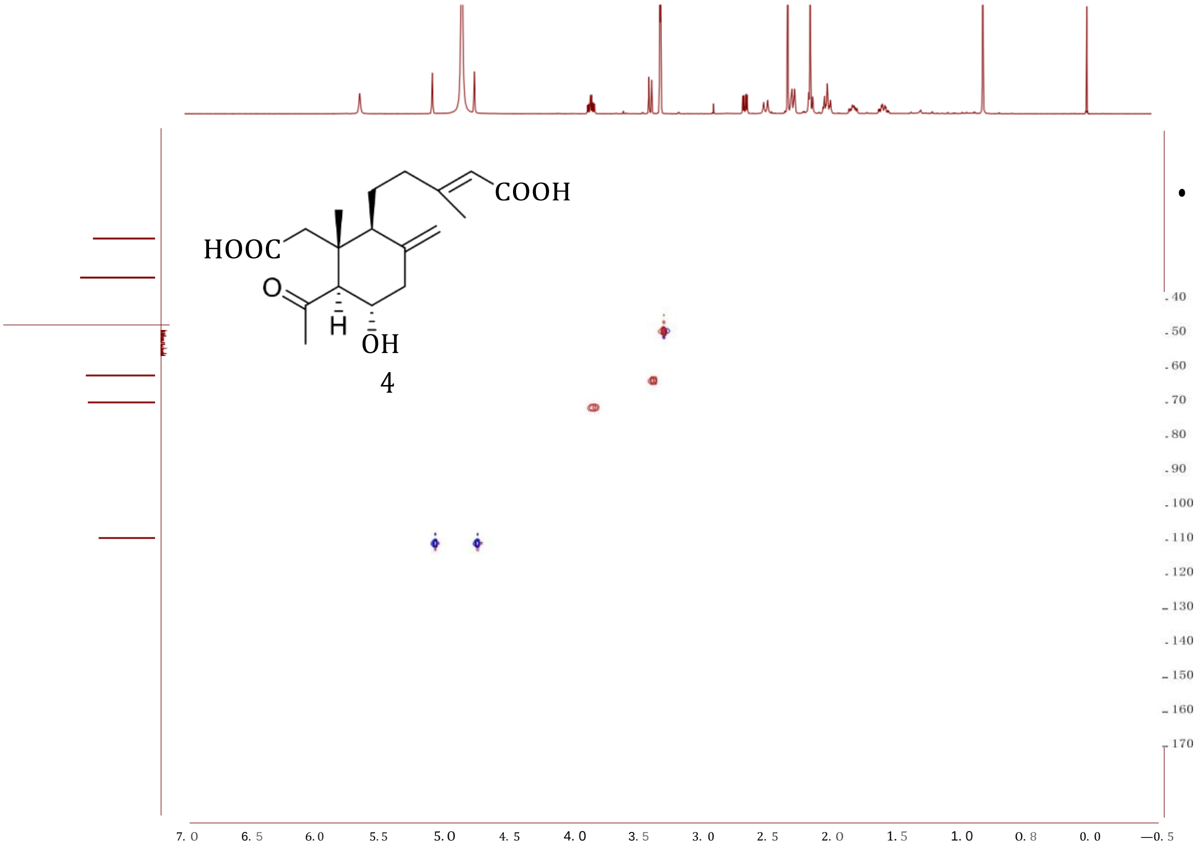

Figure S23. HMBC of **4** in CD_3OD.

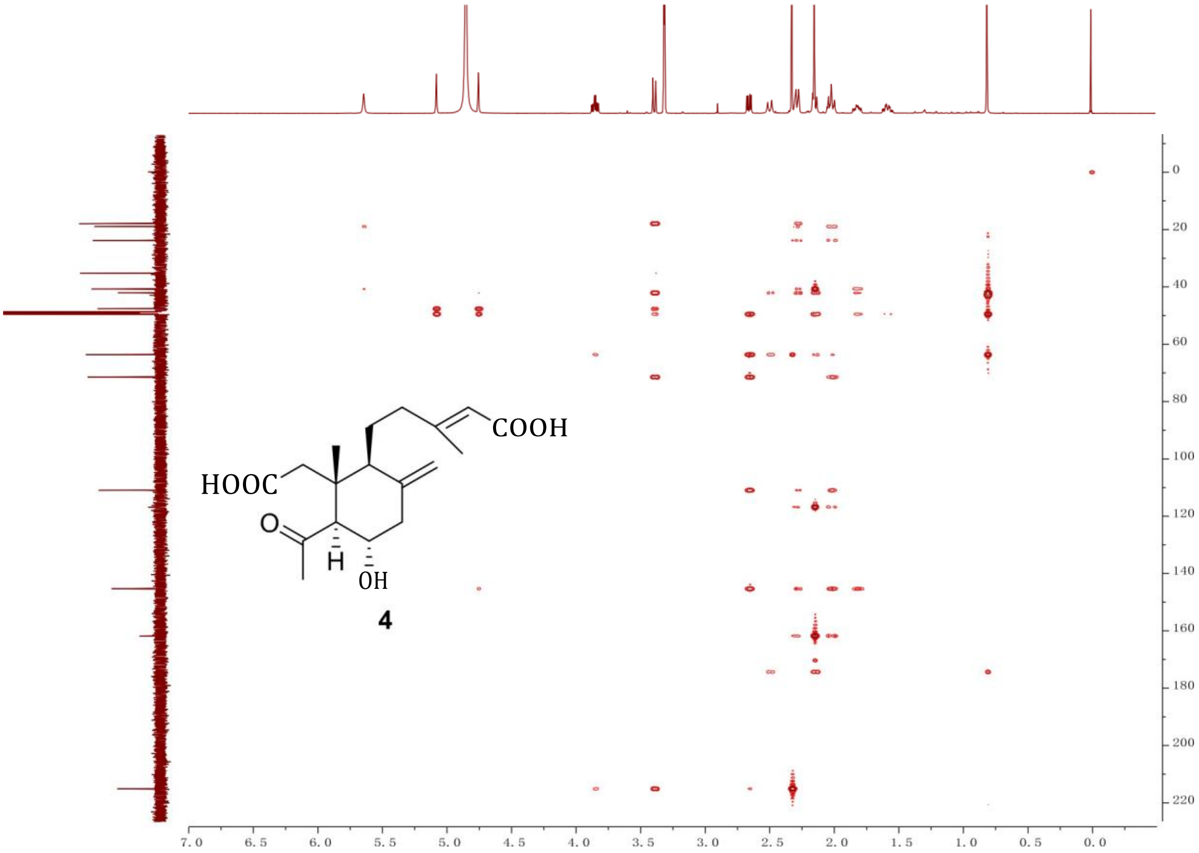

Figure S24. ^{1}H-^{1}H COSY of **4** in CD_3OD.

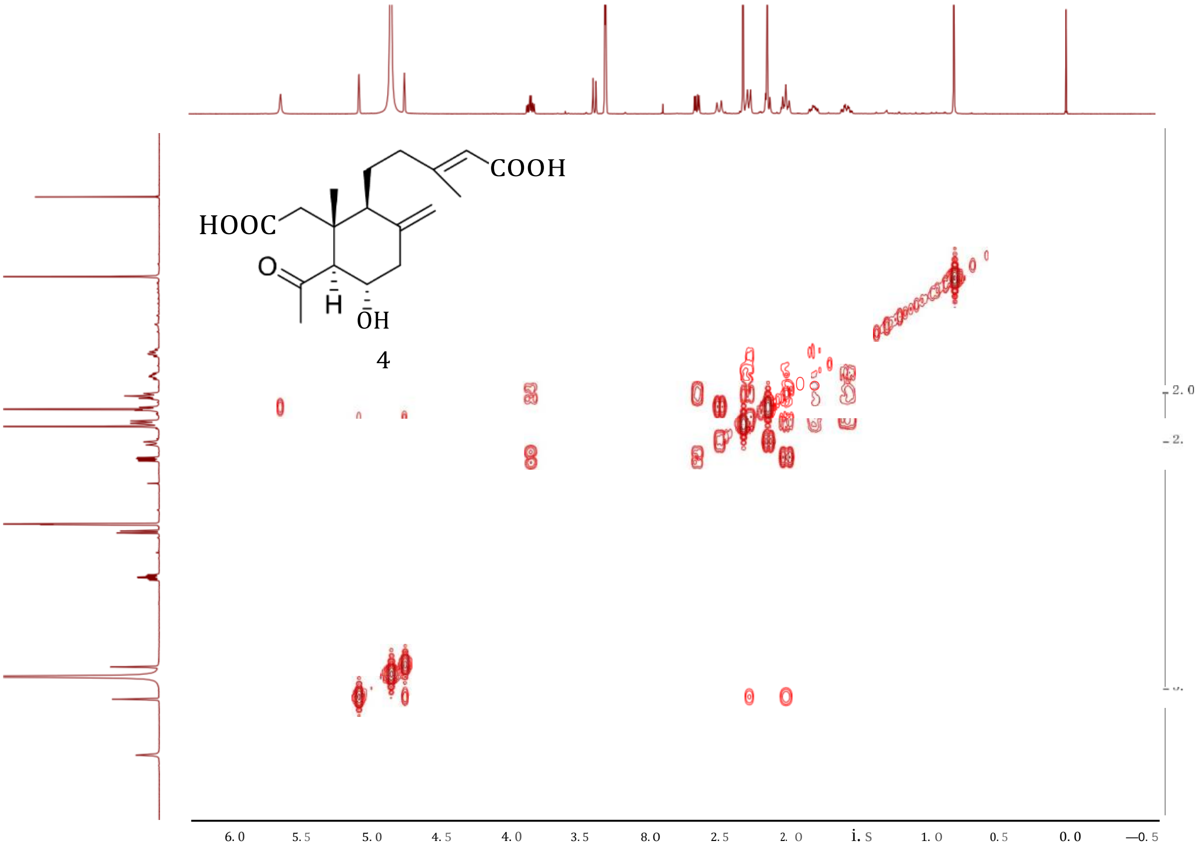

Figure S25. ROESY of **4** in CD_3OD.

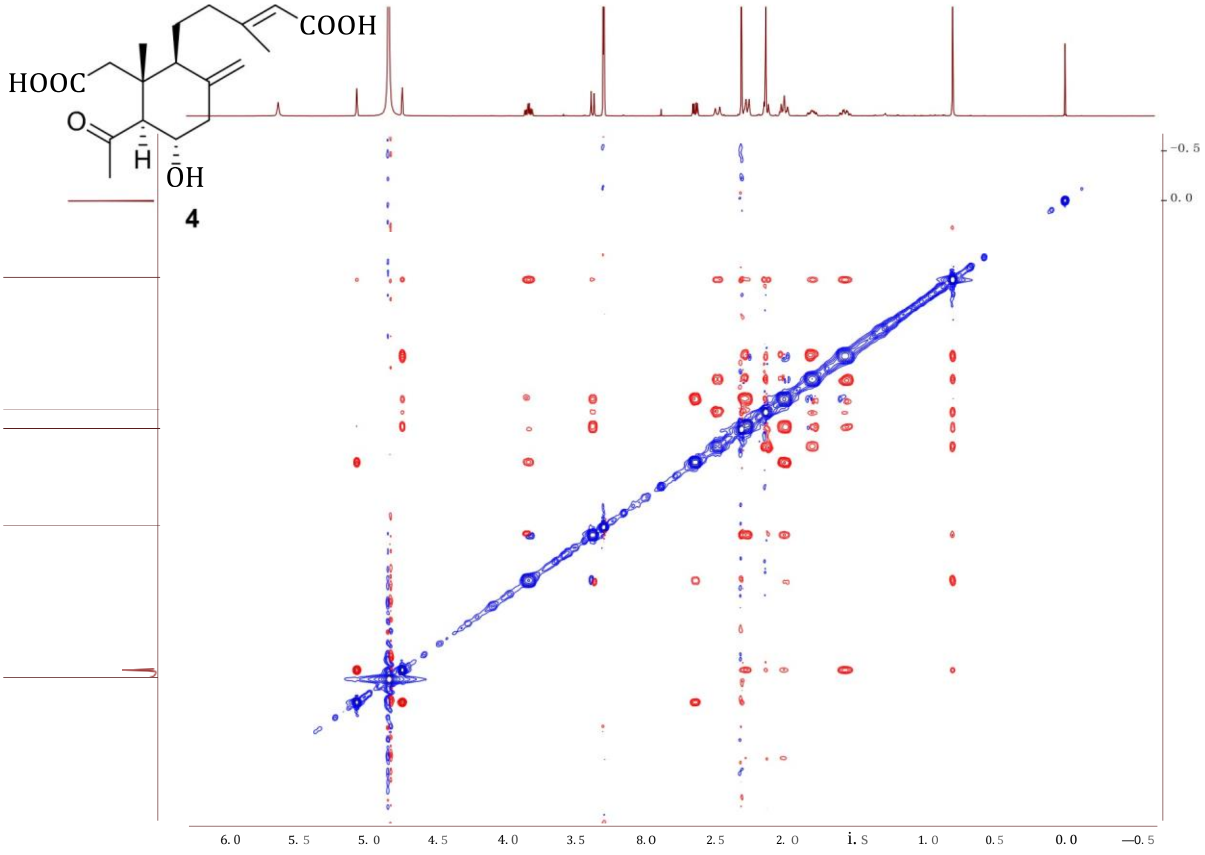

Figure S26. HRESIMS of **4**.

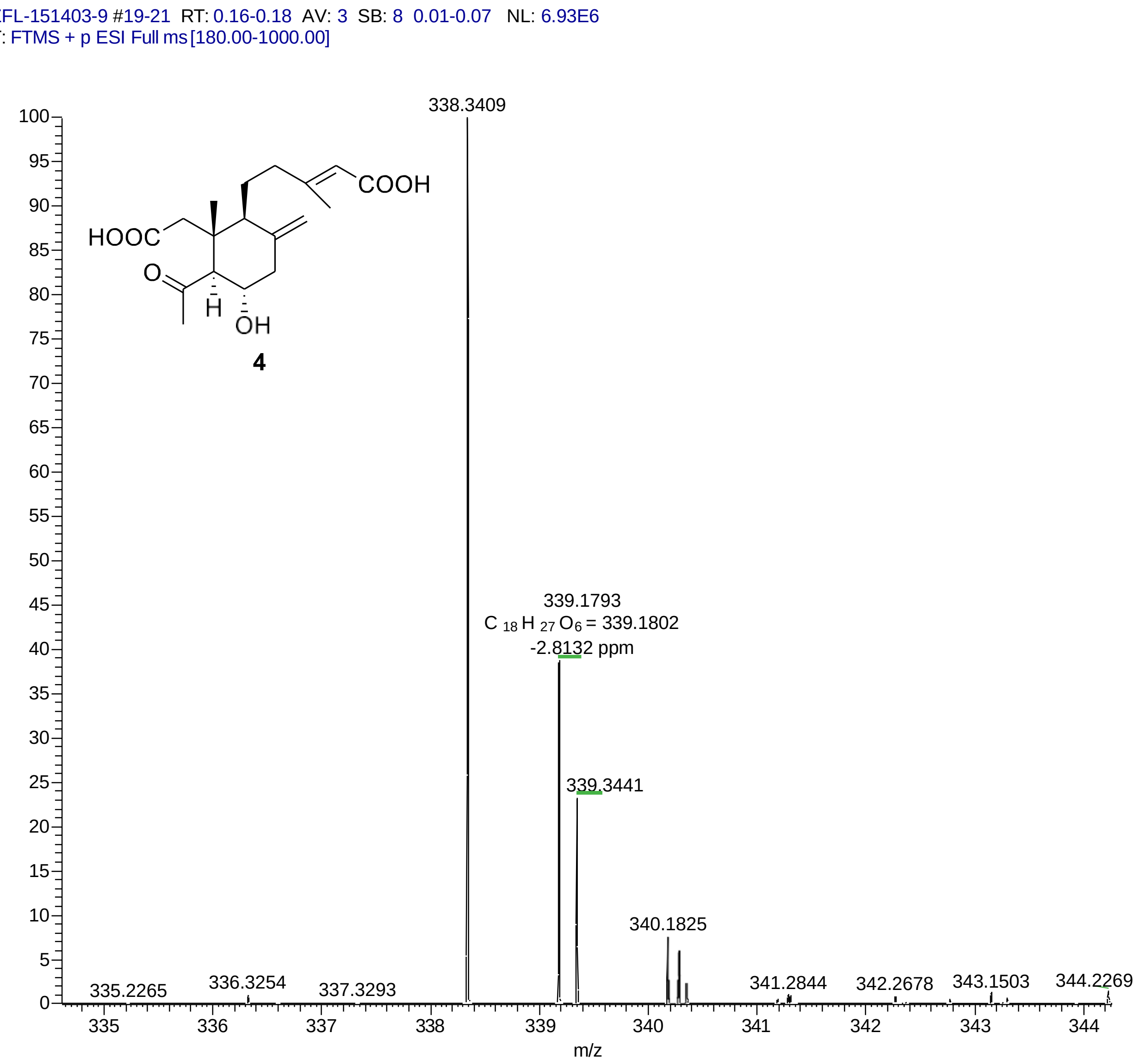

Figure S27. UV spectrum of **4**.

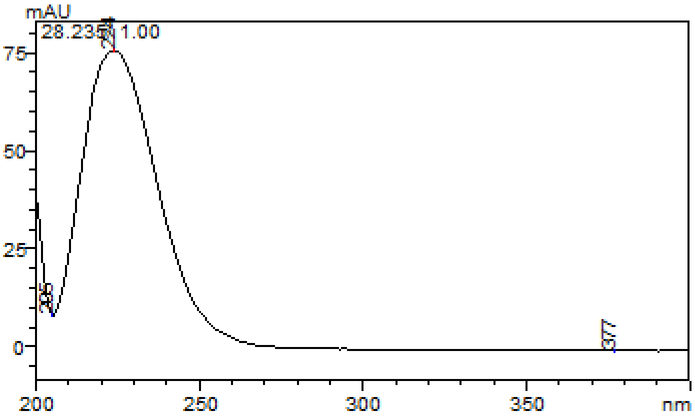

Figure S28. ^{1}H NMR of **5** in CD_3OD.

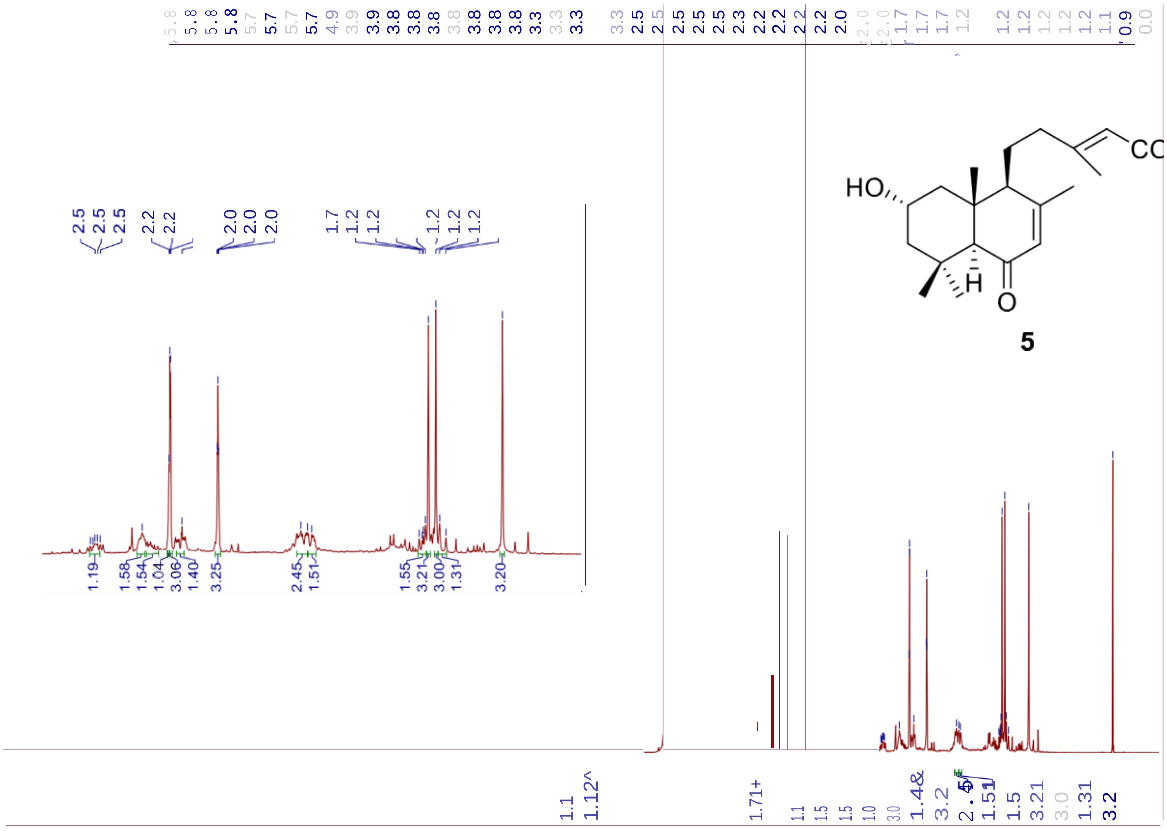

Figure S29. ^{13}C NMR of **5** in CD_3OD.

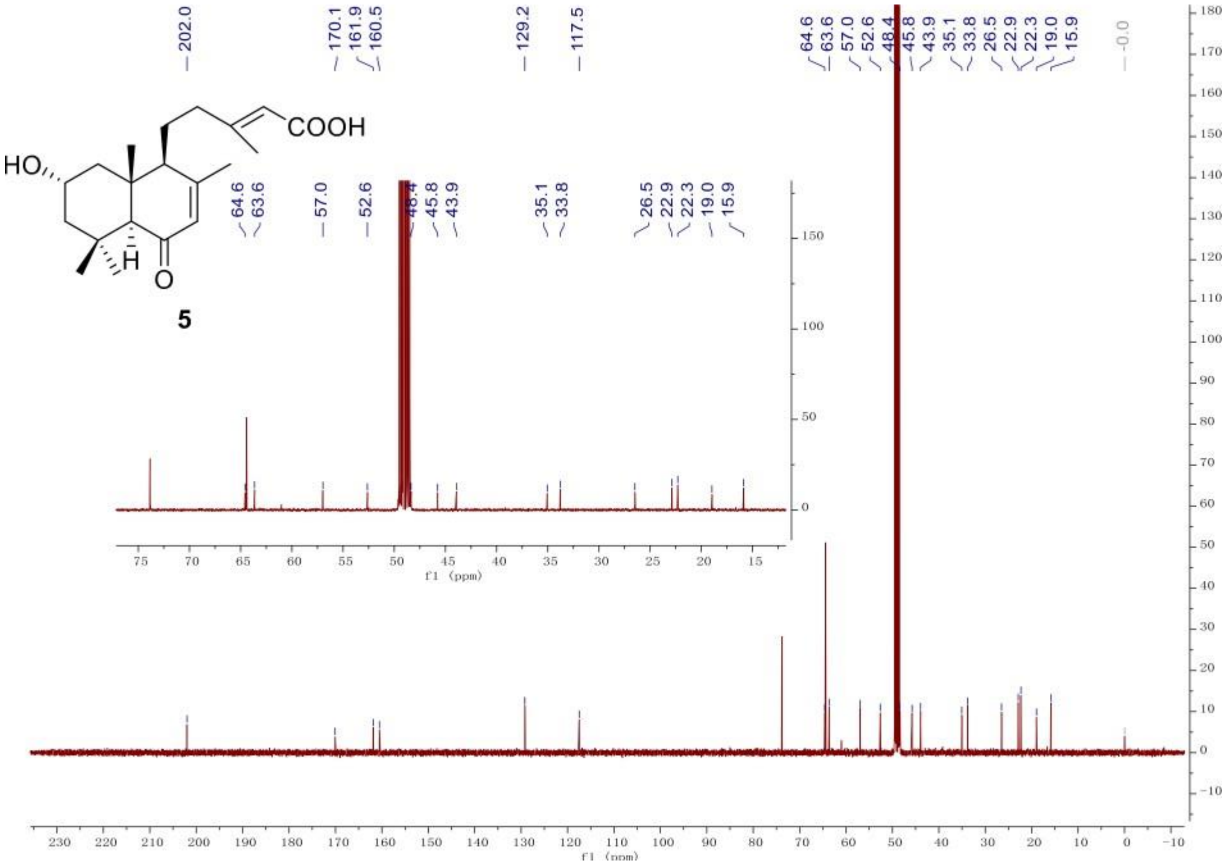

Figure S30. HSQC of **5** in CD_3OD.

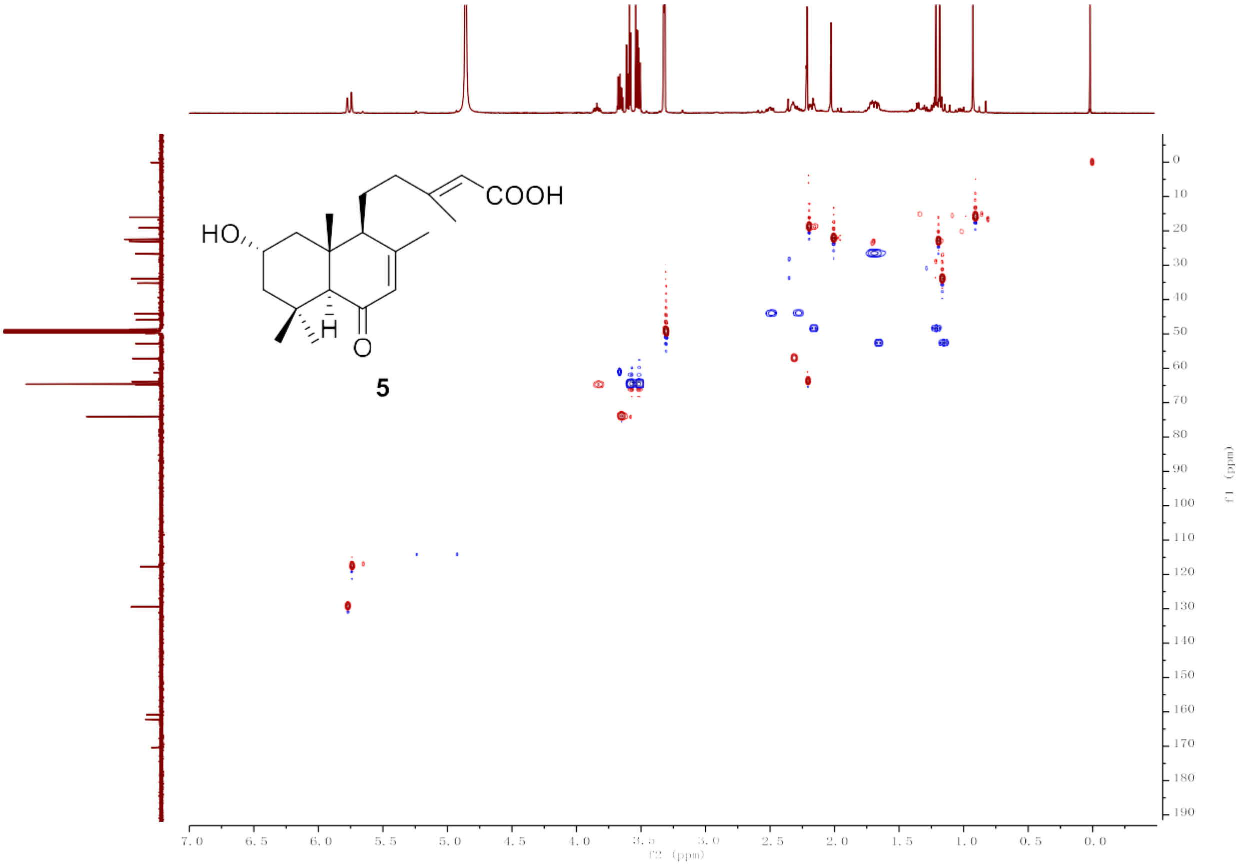

Figure S31. HMBC of **5** in CD_3OD.

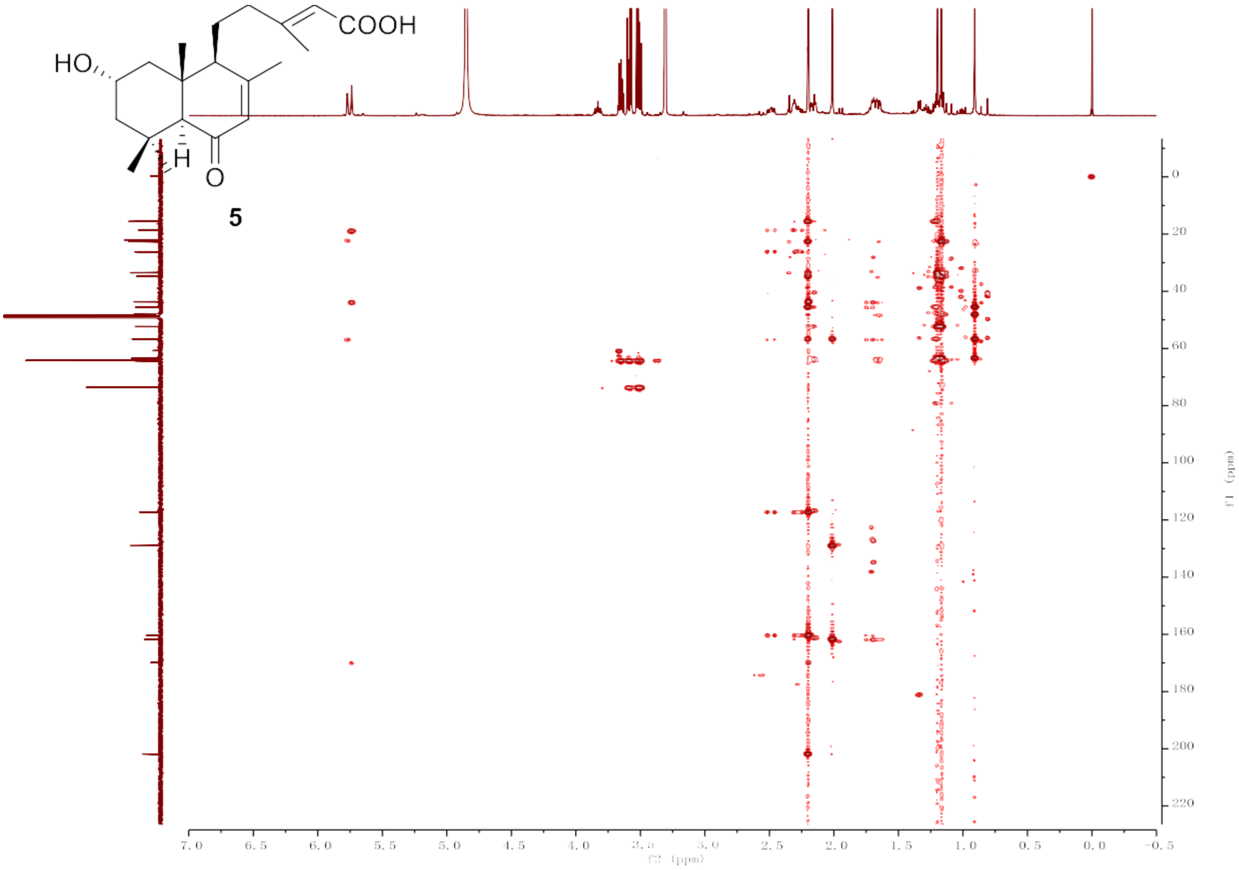

Figure S32. ^{1}H-^{1}H COSY of **5** in CD_3OD.

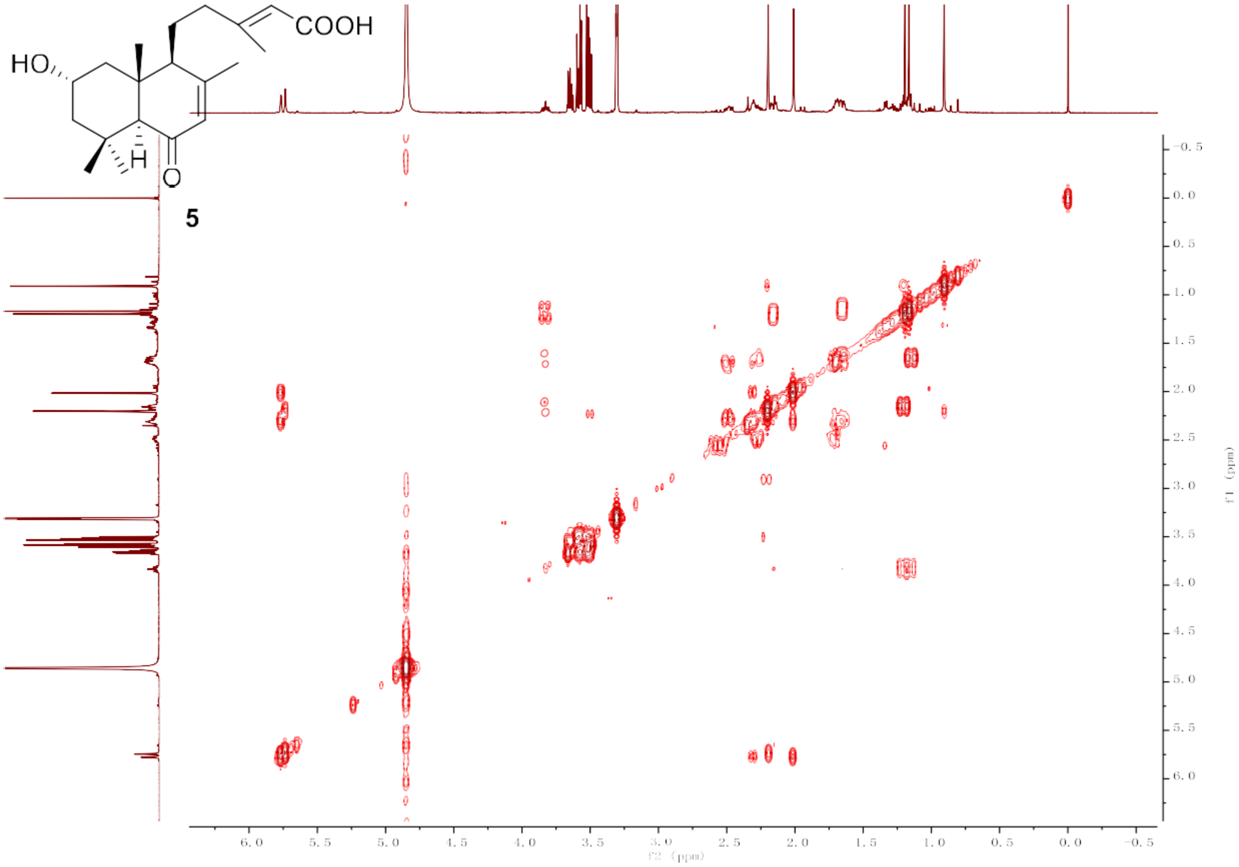

Figure S33. ROESY of **5** in CD_3OD.

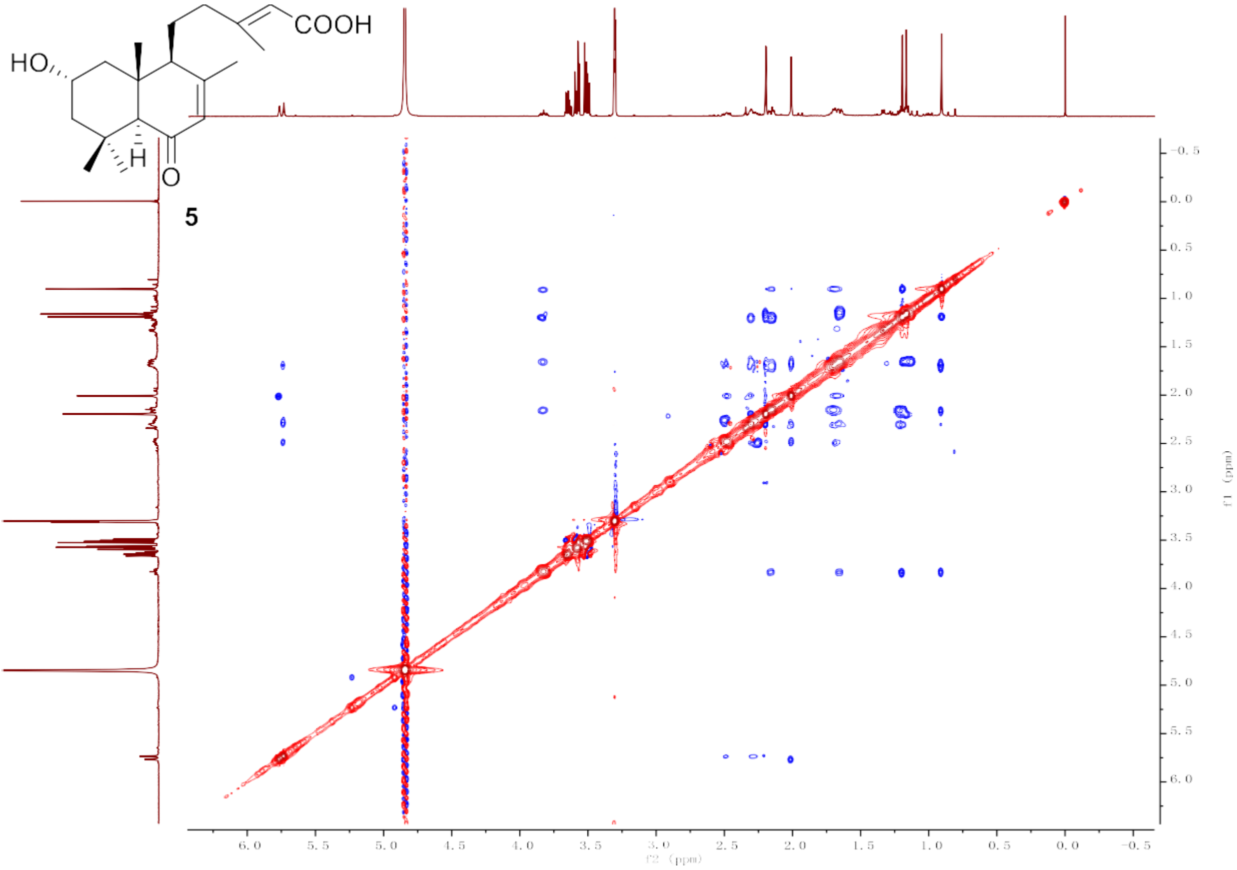

Figure S34. HRESIMS of **5**.

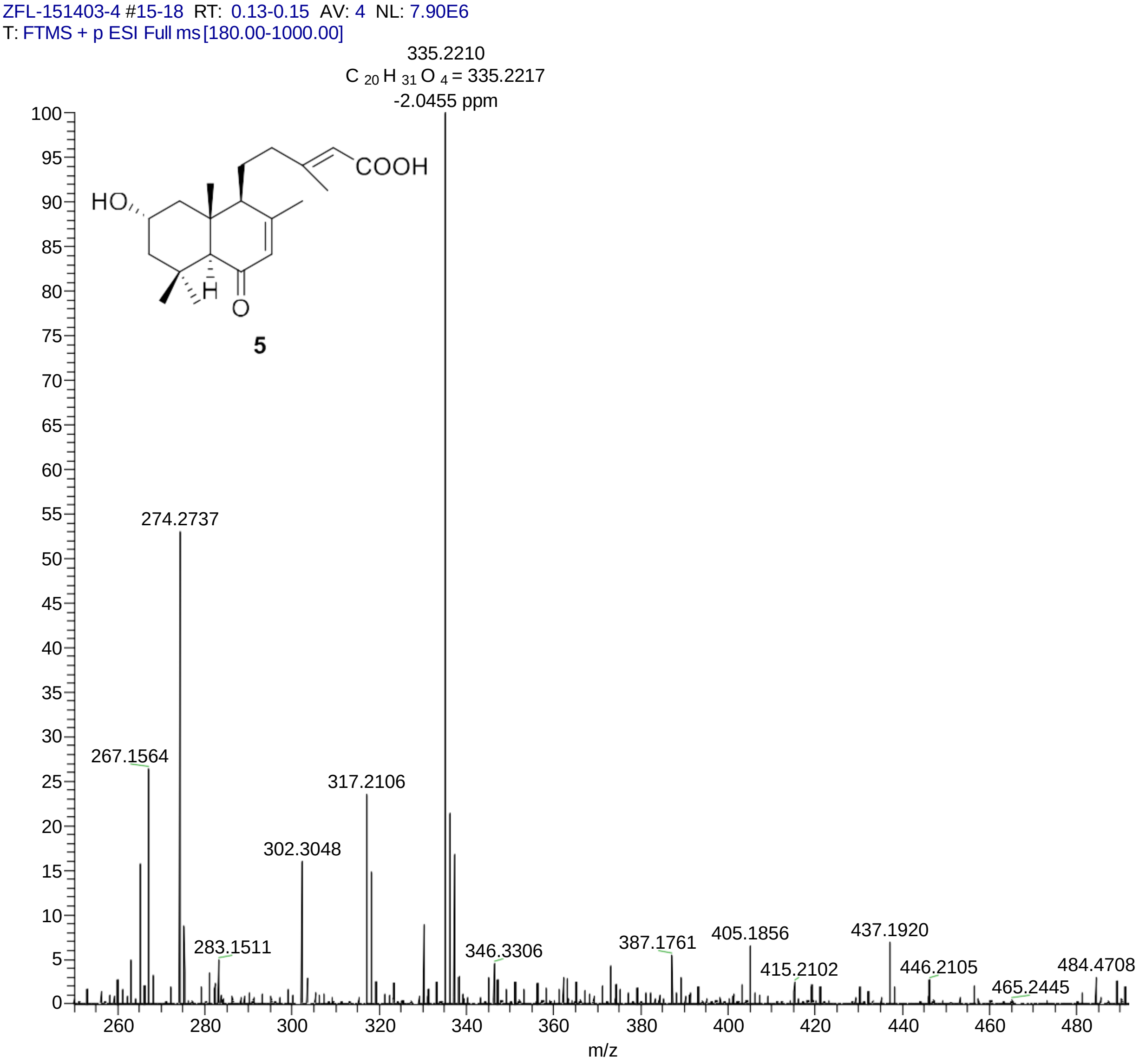

Figure S35. UV spectrum of **5**.

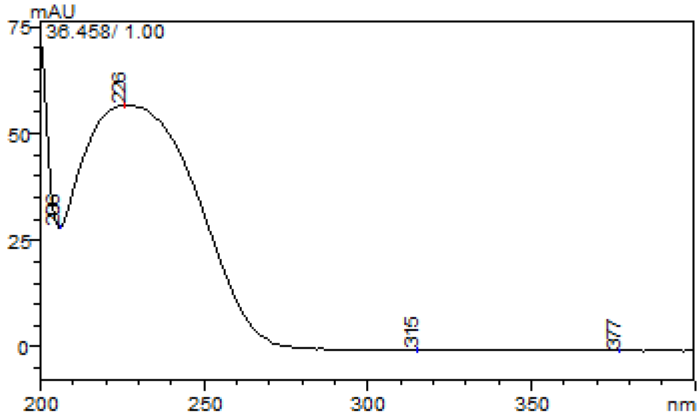

Figure S36. ^{1}H NMR of **6** in CD_3OD.

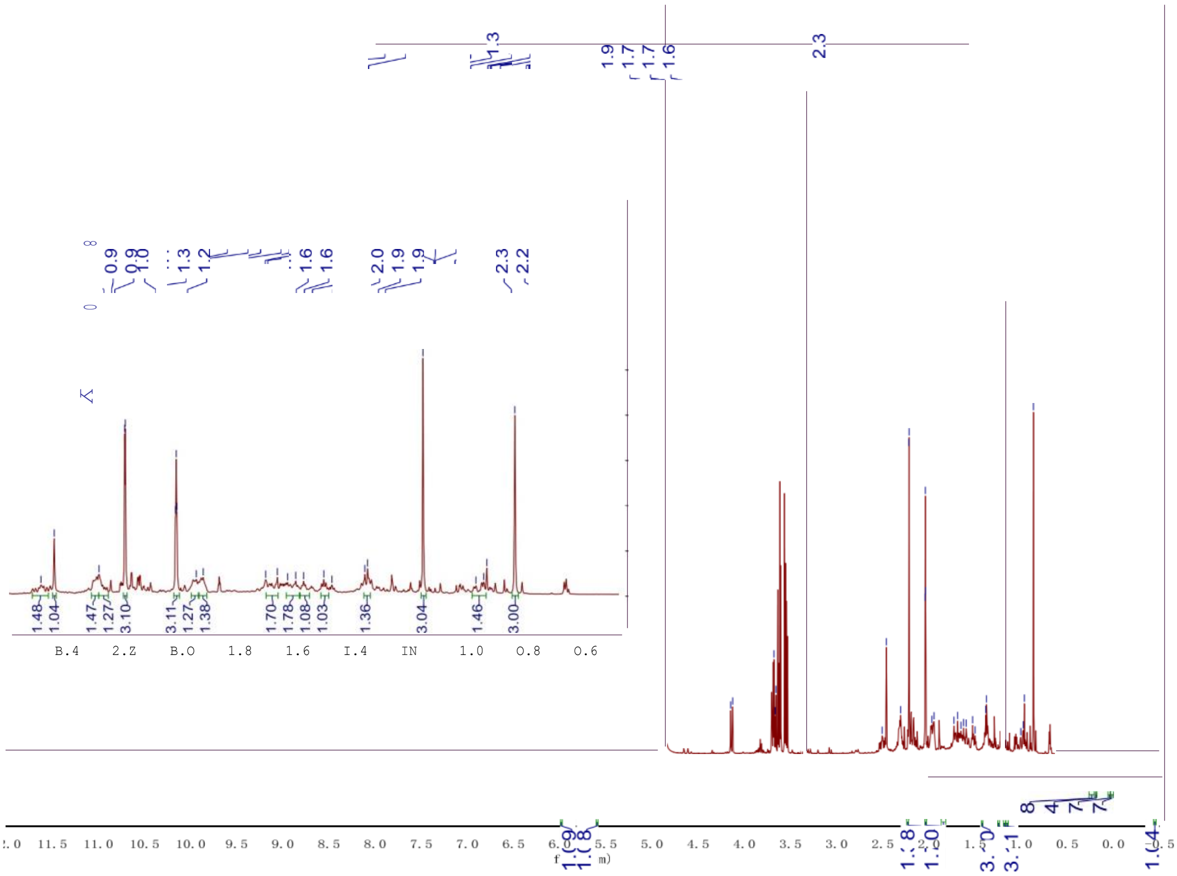

Figure S37. ^{13}C NMR of **6** in CD_3OD.

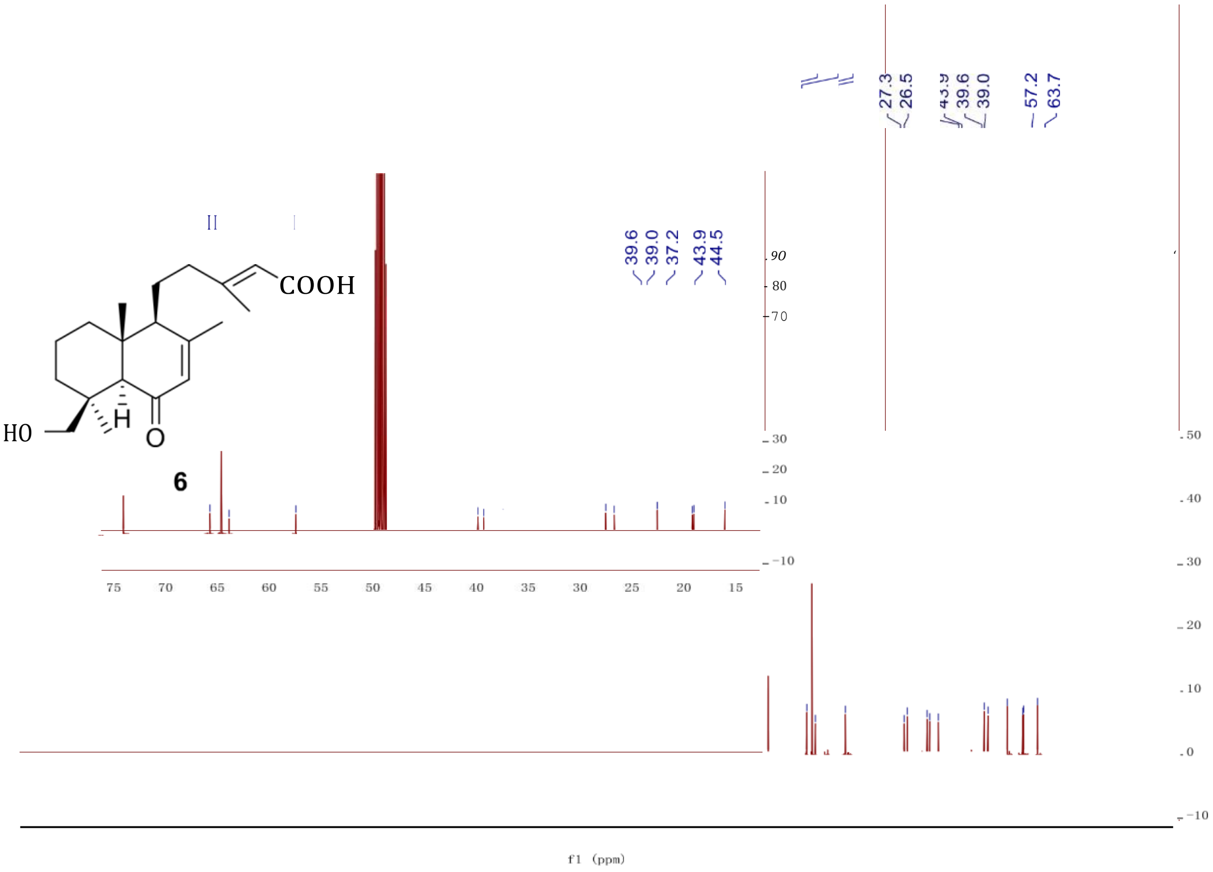

Figure S38. HSQC of **6** in CD_3OD.

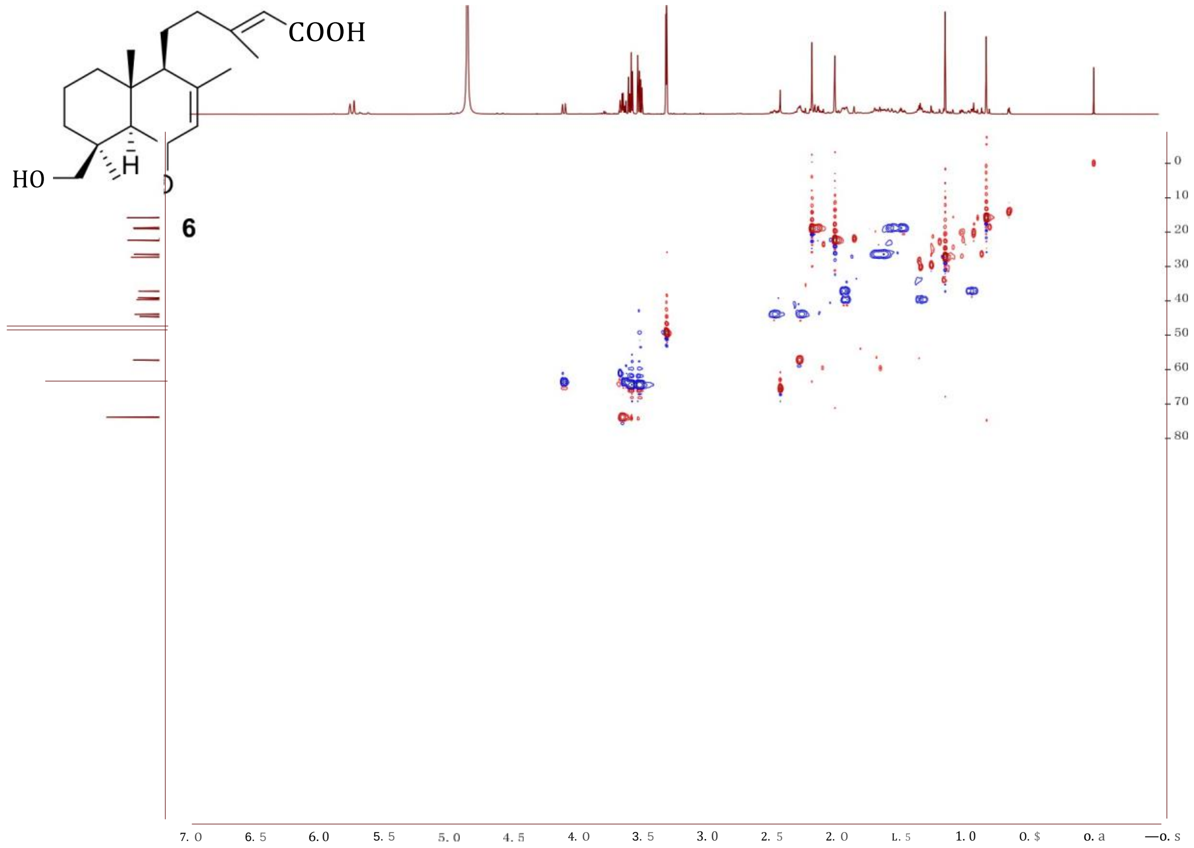

Figure S39. HMBC of **6** in CD_3OD.

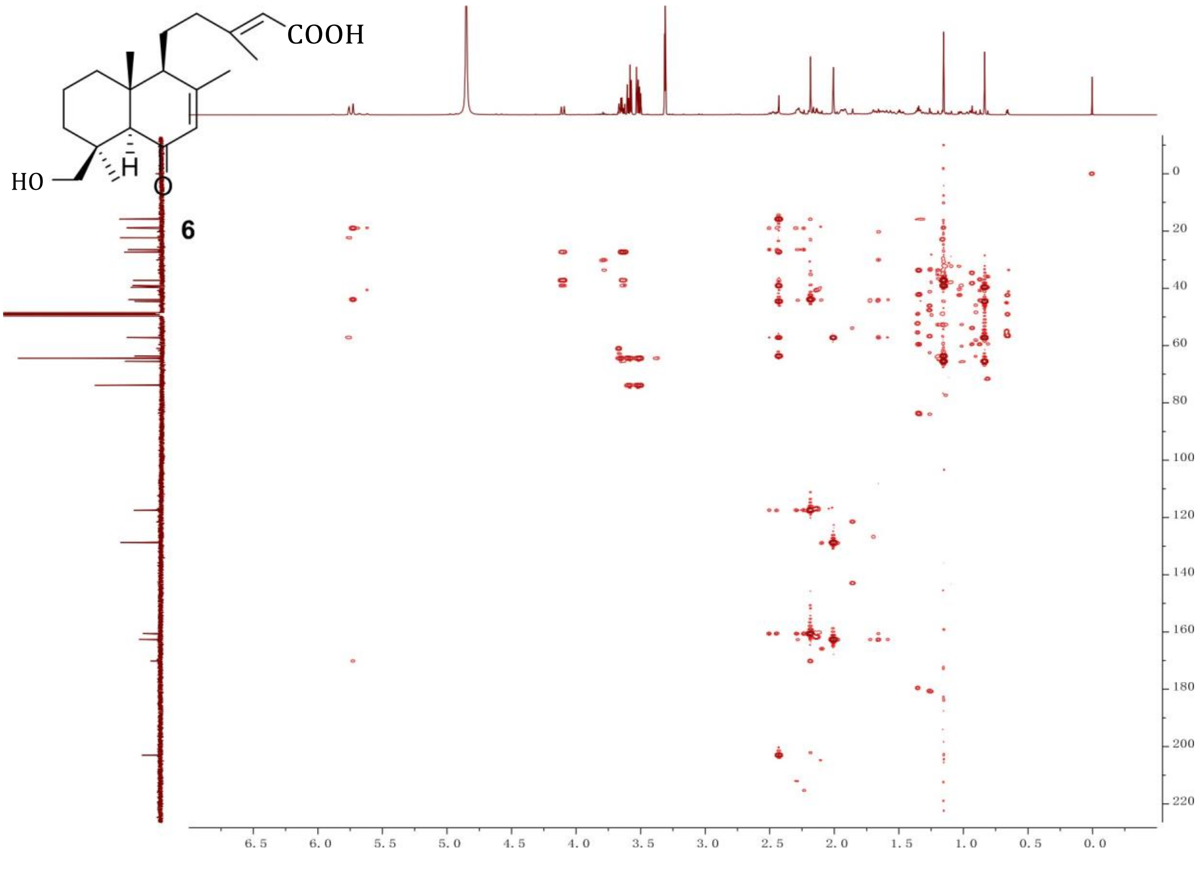

Figure S40. ^{1}H-^{1}H COSY of **6** in CD_3OD.

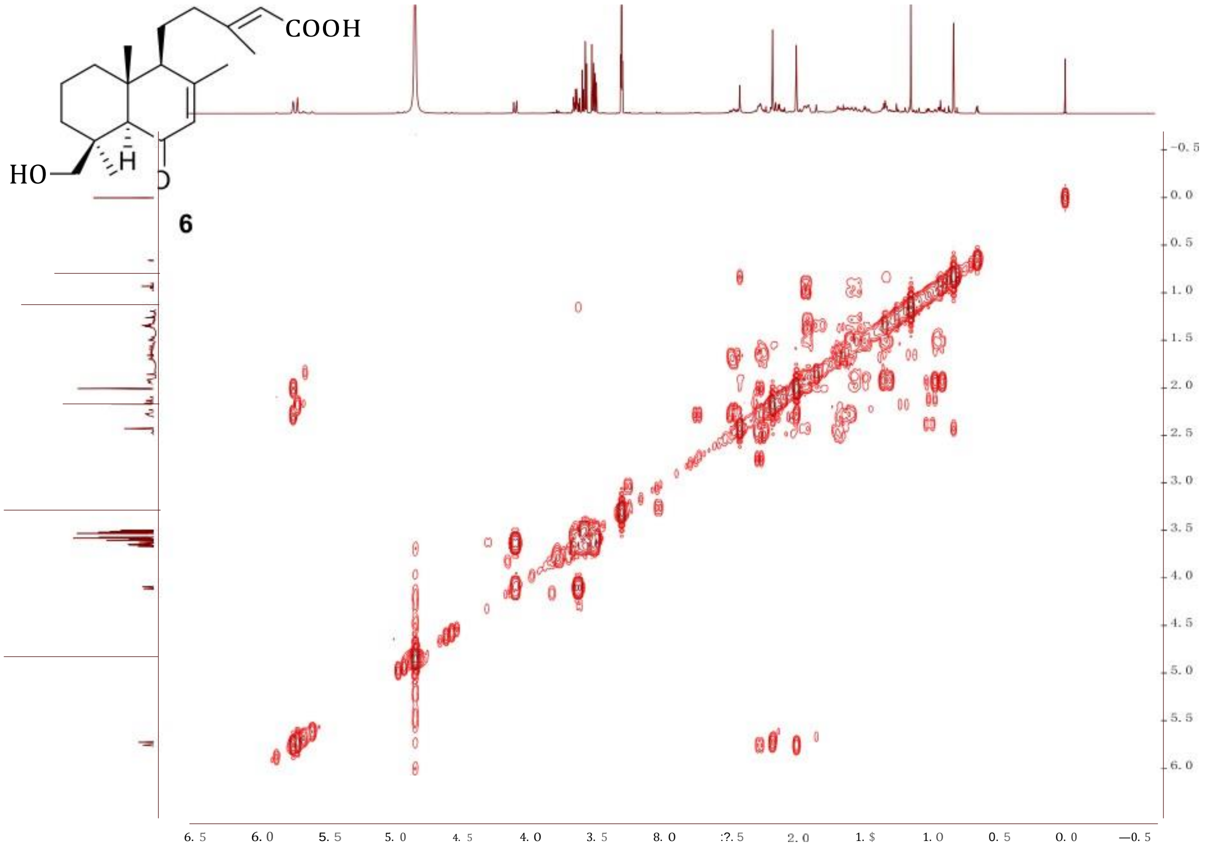

Figure S41. ROESY of **6** in CD_3OD.

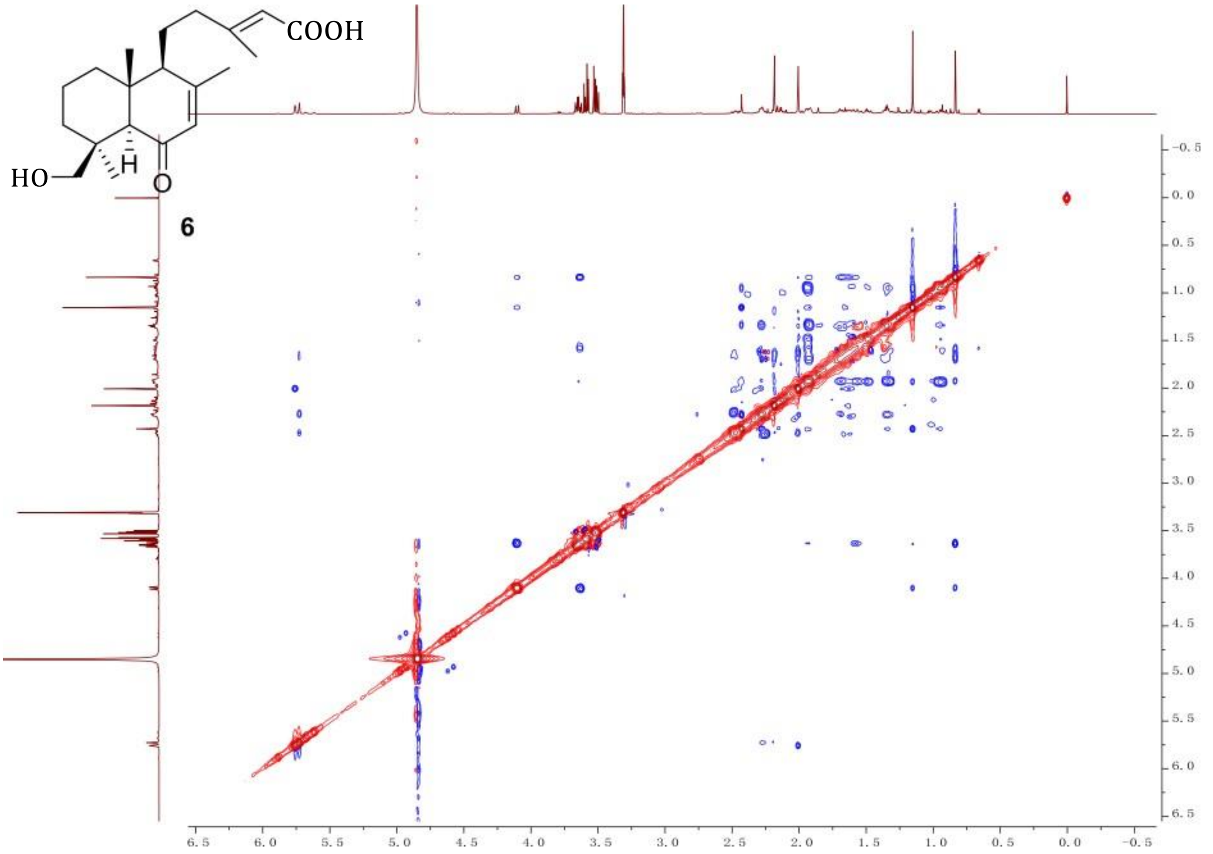

Figure S42. HRESIMS of **6**.

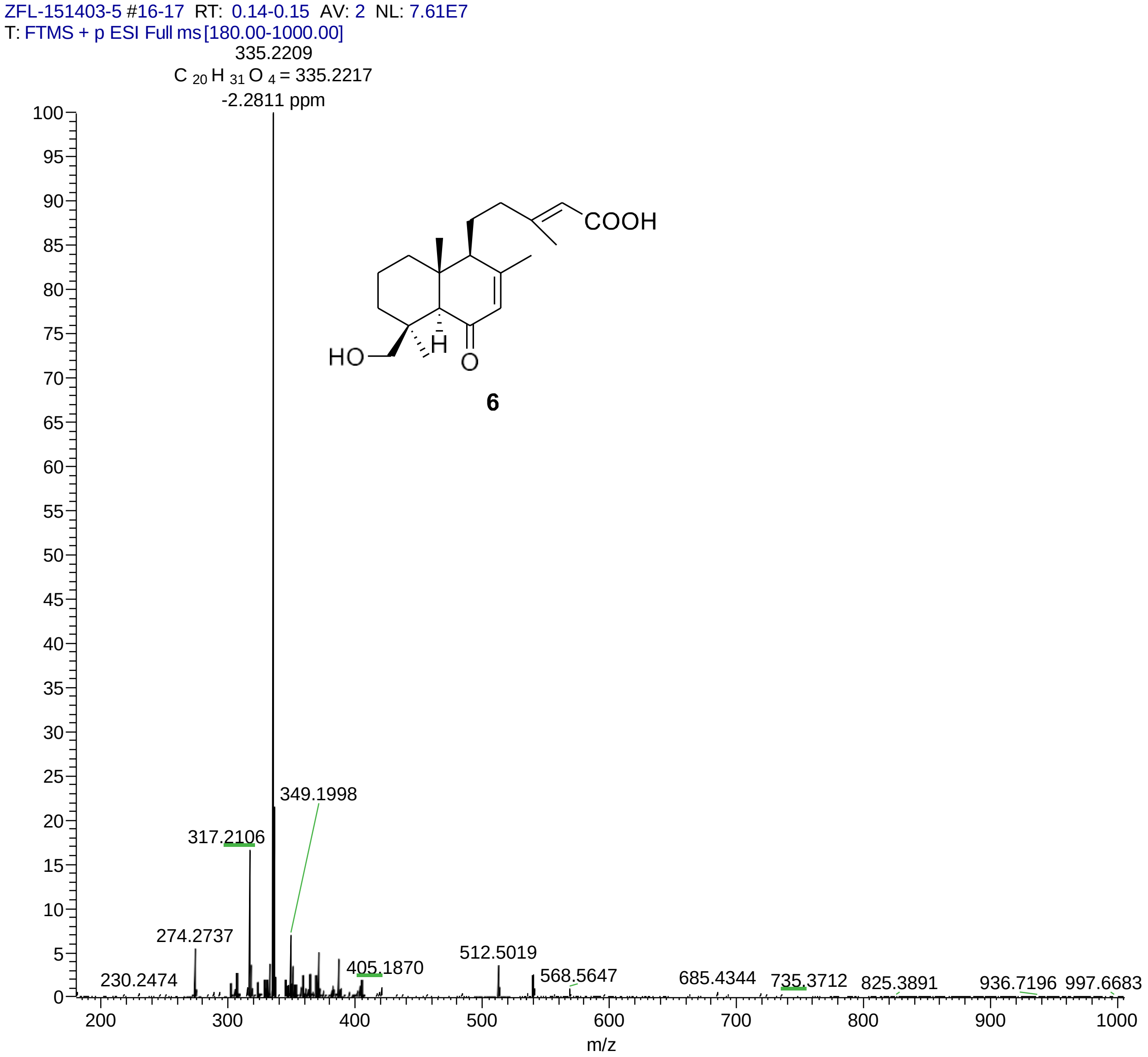

Figure S43. UV spectrum of **6**.

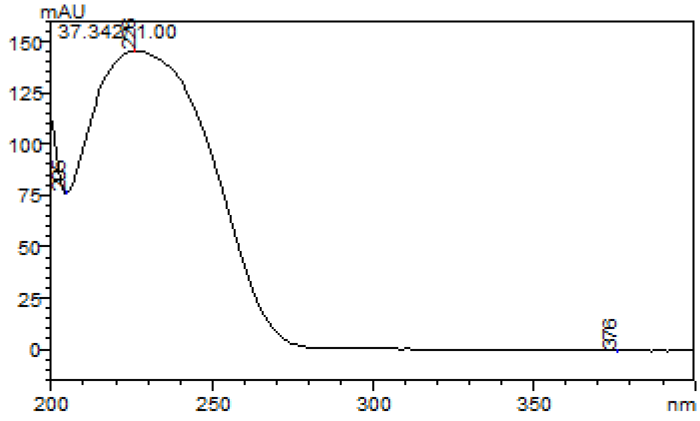

Figure S44. ^{1}H NMR of **7** in CD_3OD.

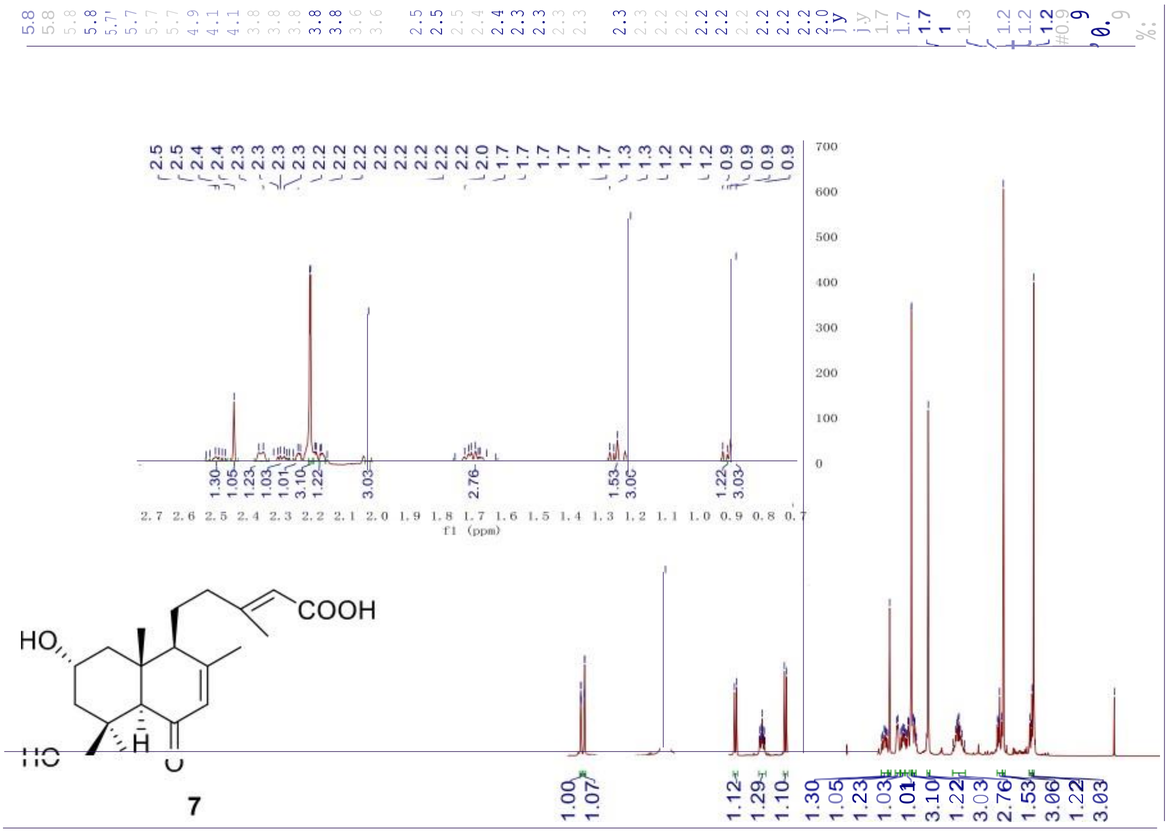

Figure S45. ^{13}C NMR of **7** in CD_3OD.

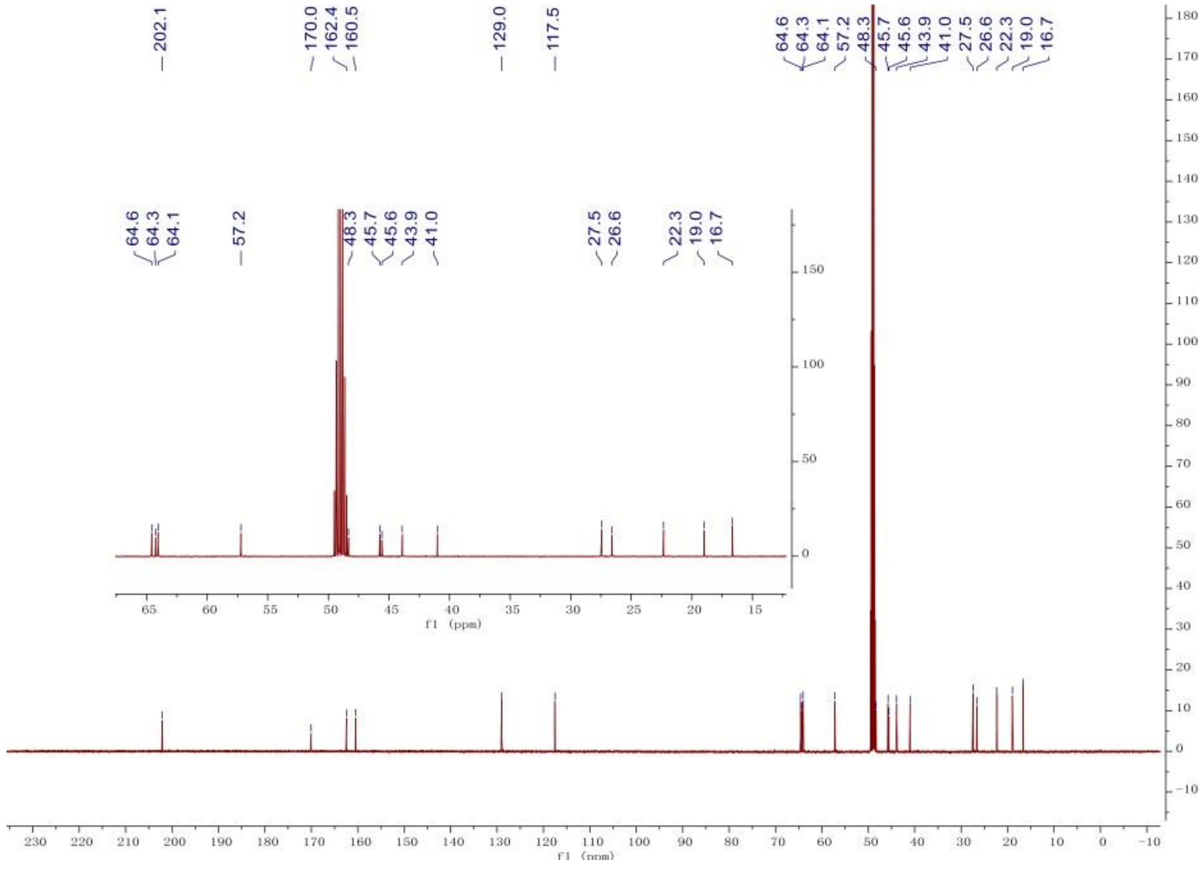

Figure S46. HSQC of **7** in CD_3OD.

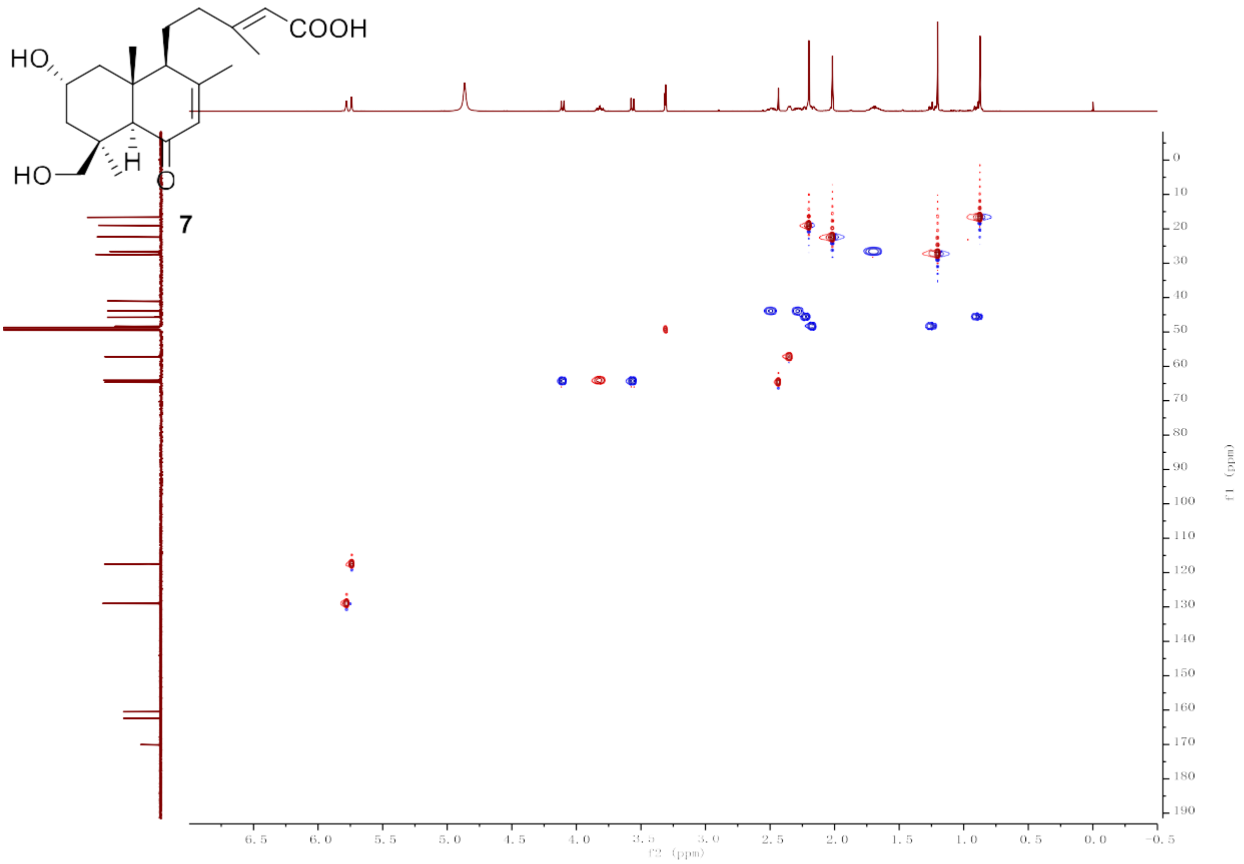

Figure S47. HMBC of **7** in CD_3OD.

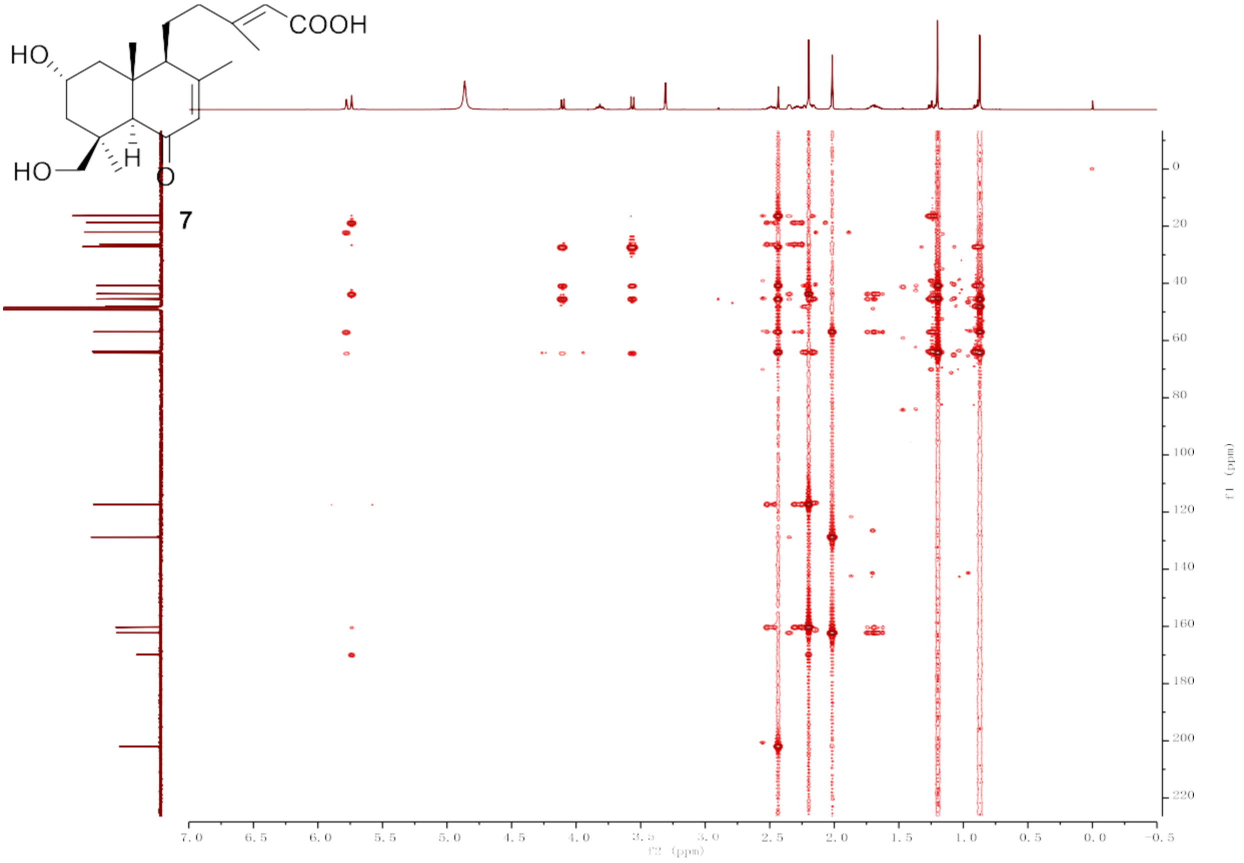

Figure S48. ^{1}H-^{1}H COSY of **7** in CD_3OD.

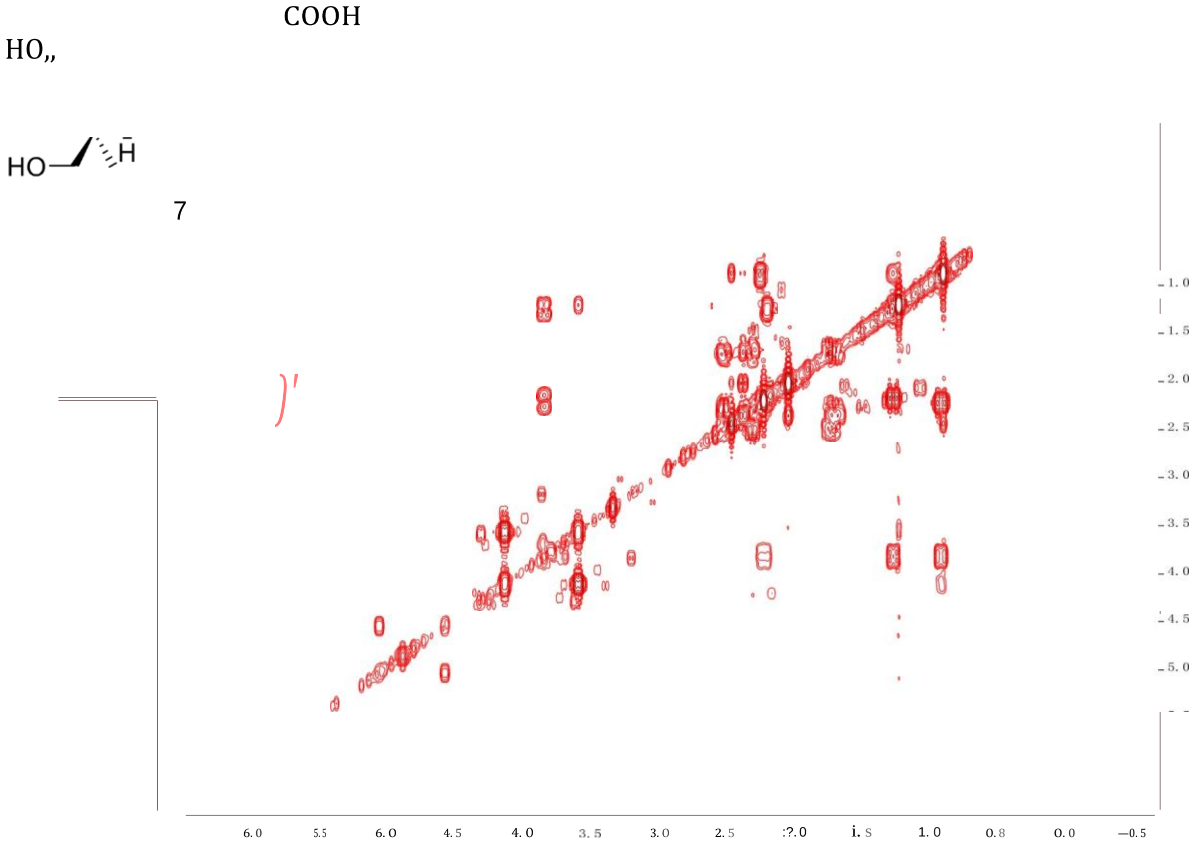

Figure S49. ROESY of **7** in CD_3OD.

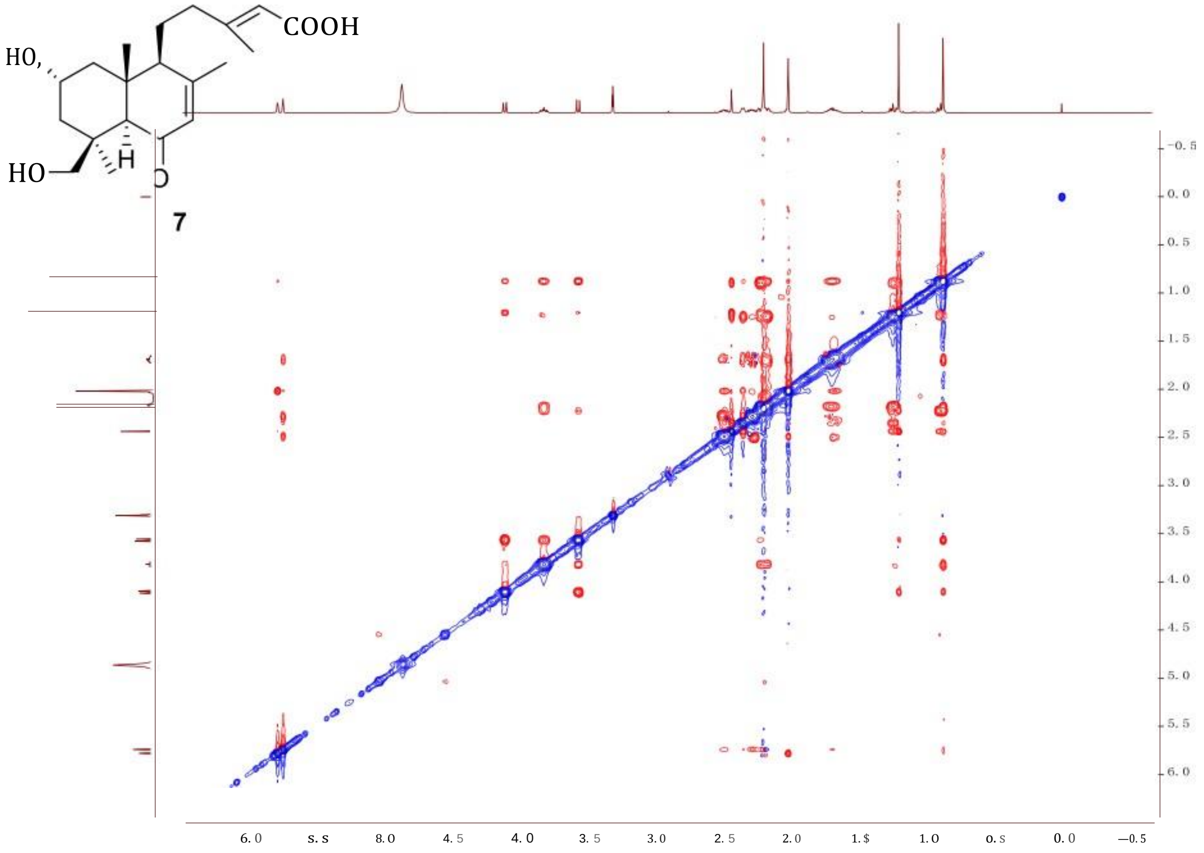

Figure S50. HRESIMS of **7**.

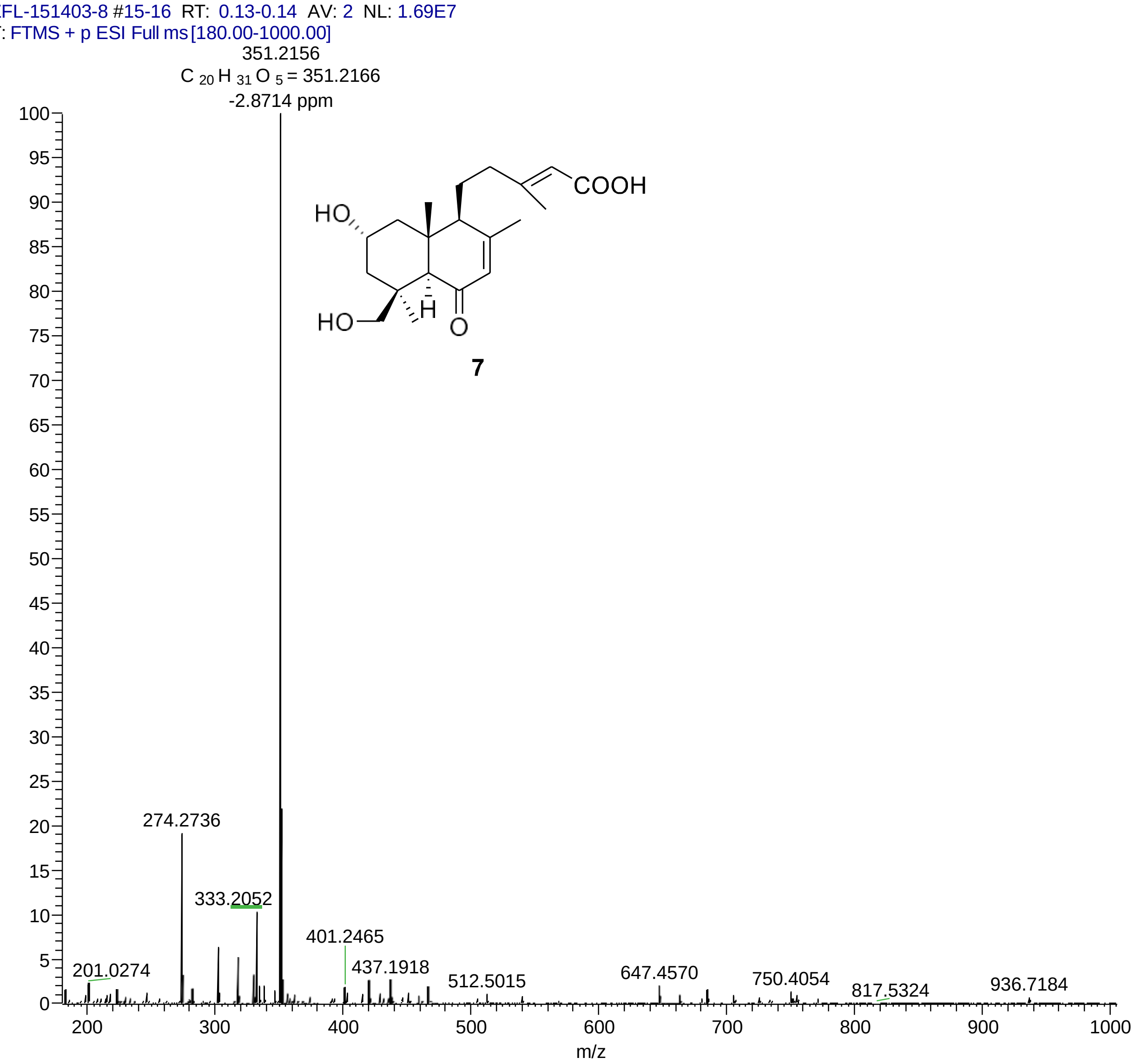

Figure S51. UV spectrum of **7**.

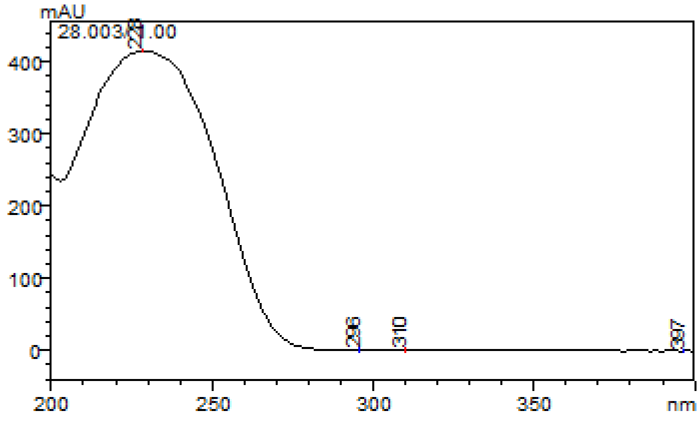

Figure S52. ^{1}H NMR of **1** in CD_3OD.

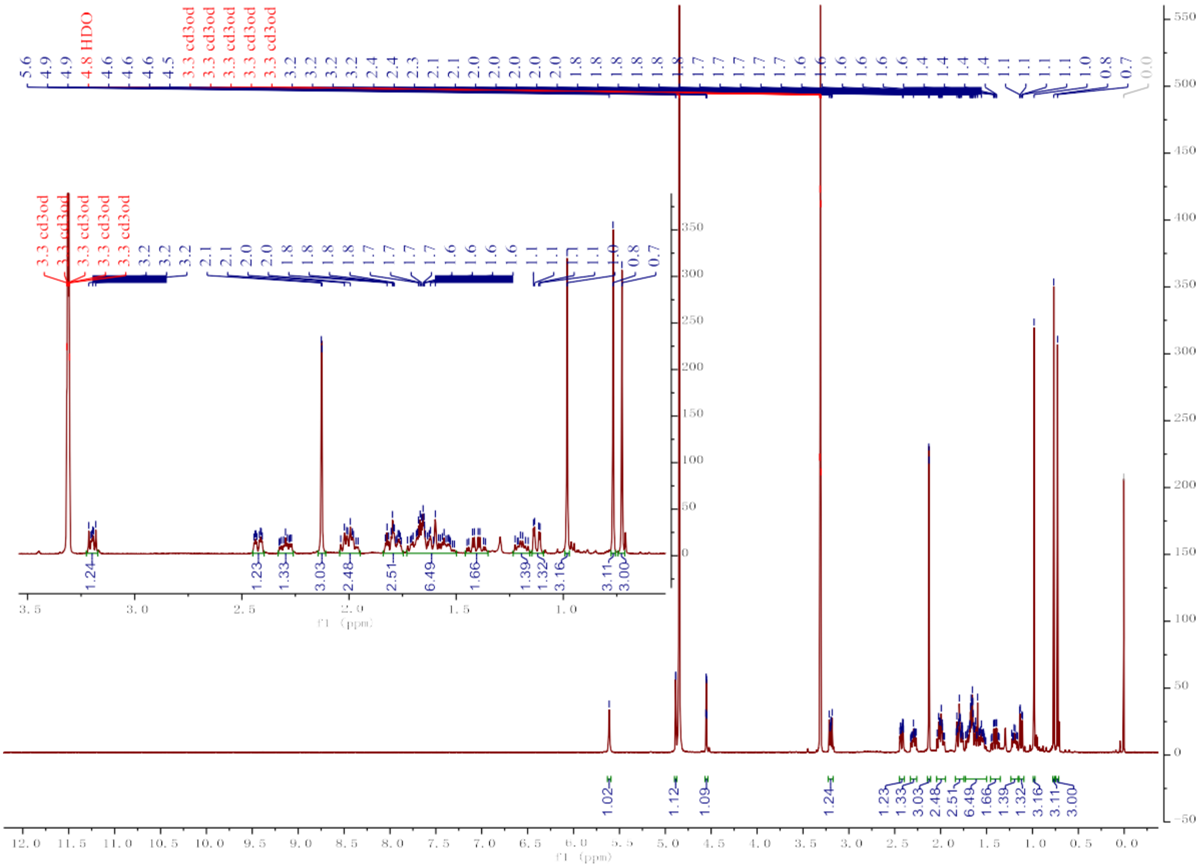

Figure S53. ^{13}C NMR of **1** in CD_3OD.

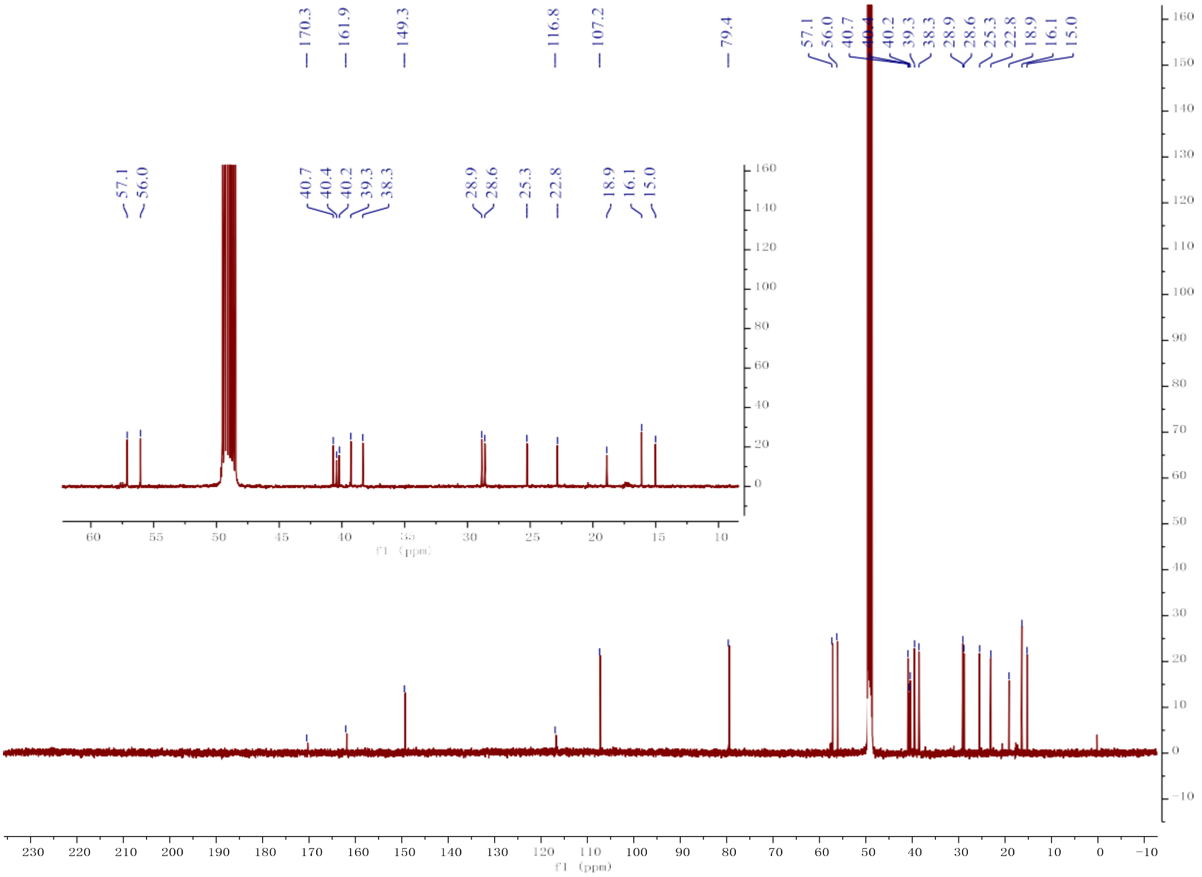

Figure S54. ^{1}H NMR of **2** in CD_3OD.

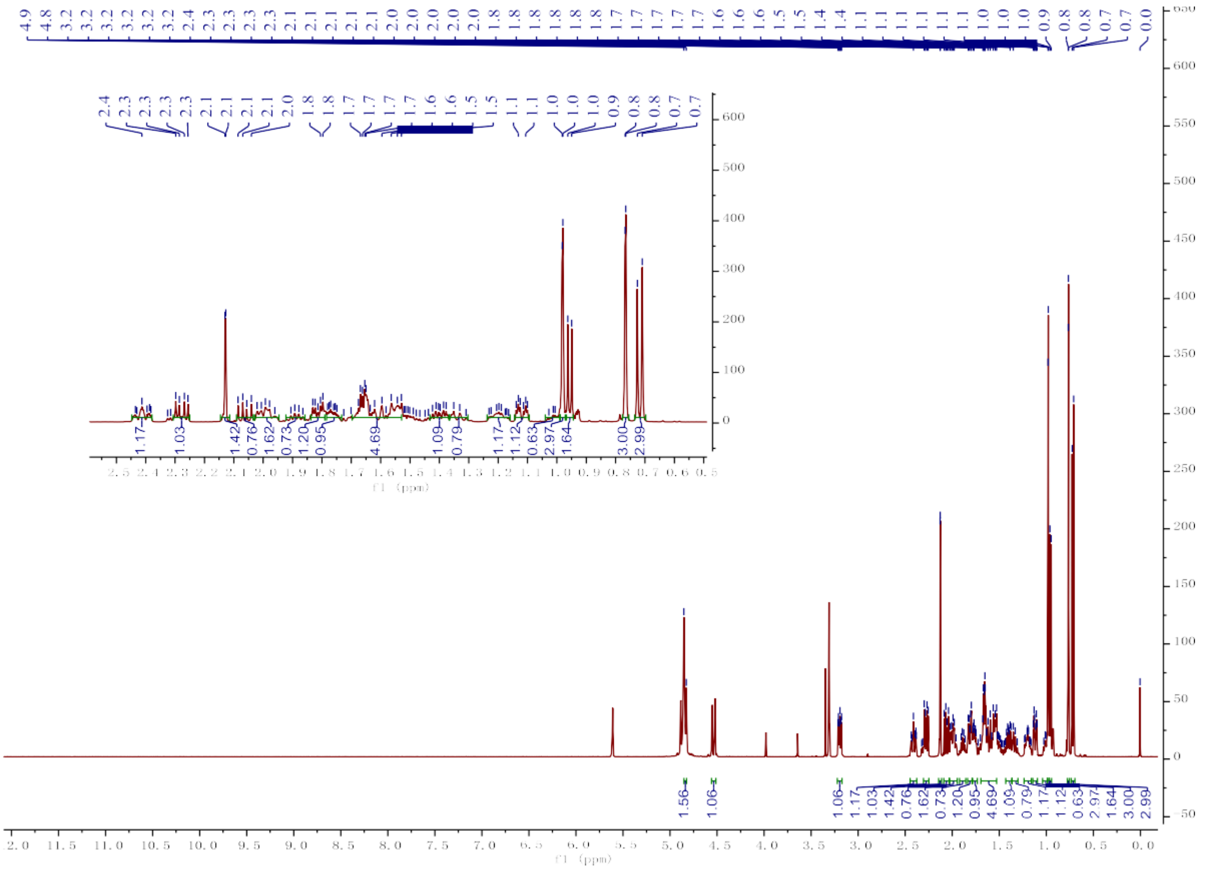

Figure S55. ^{13}C NMR of **2** in CD_3OD.

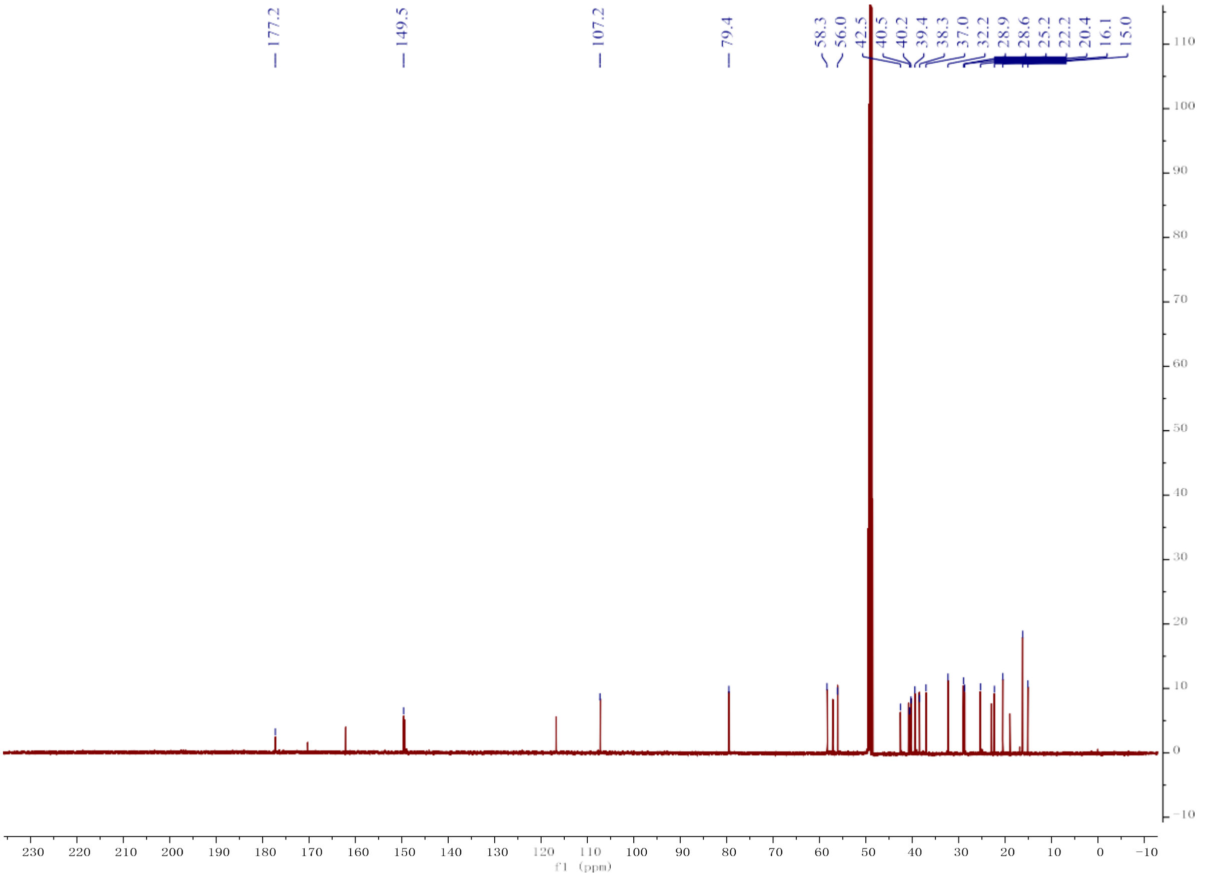

Figure S56. ^{1}H NMR of **8** in CD_3OD.

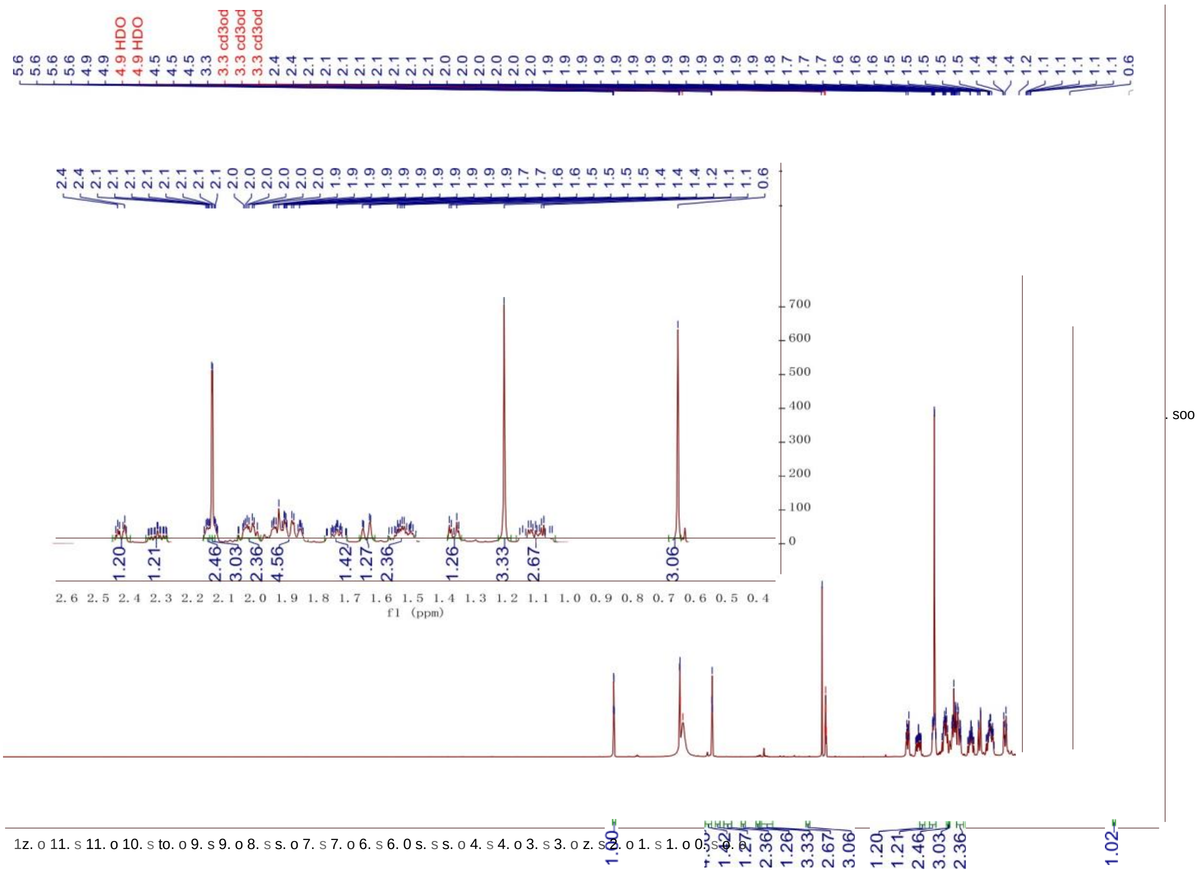

Figure S57. ^{13}C NMR of **8** in CD_3OD.

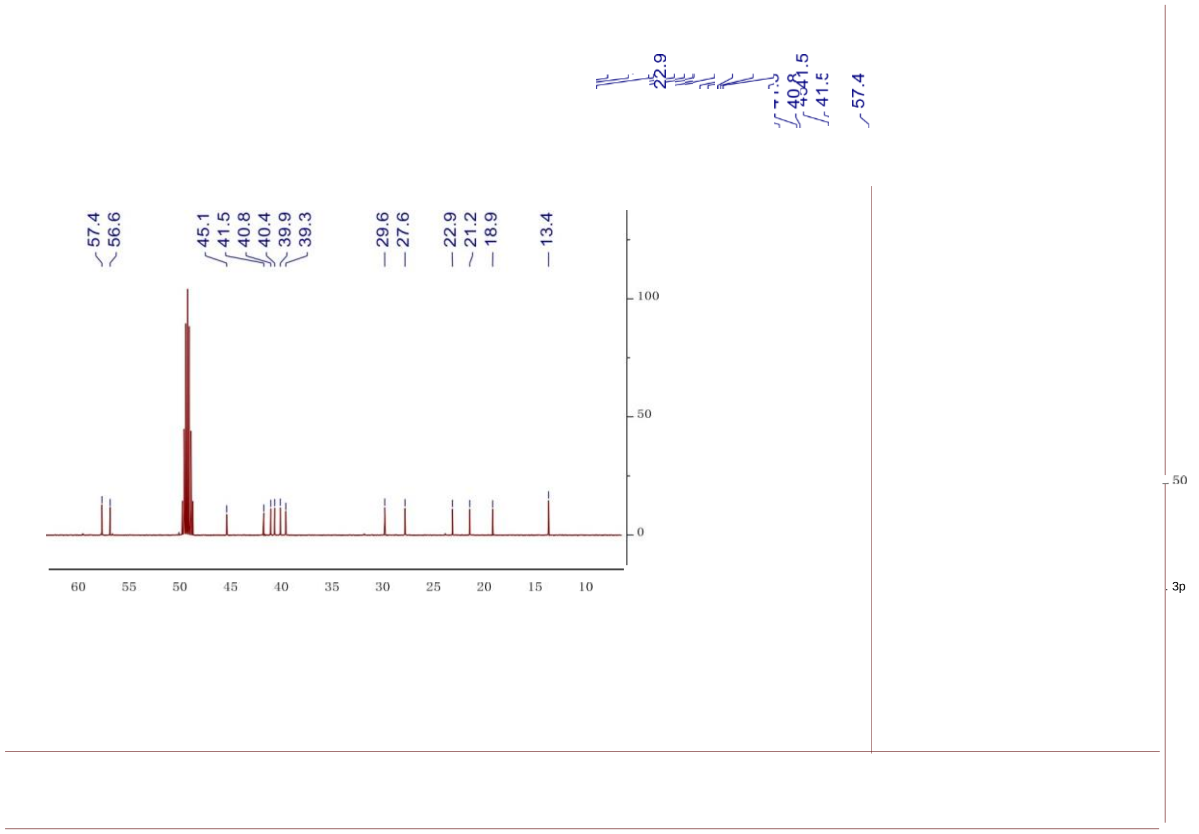

Figure S58. ^{1}H NMR of **9** in CD_3OD.

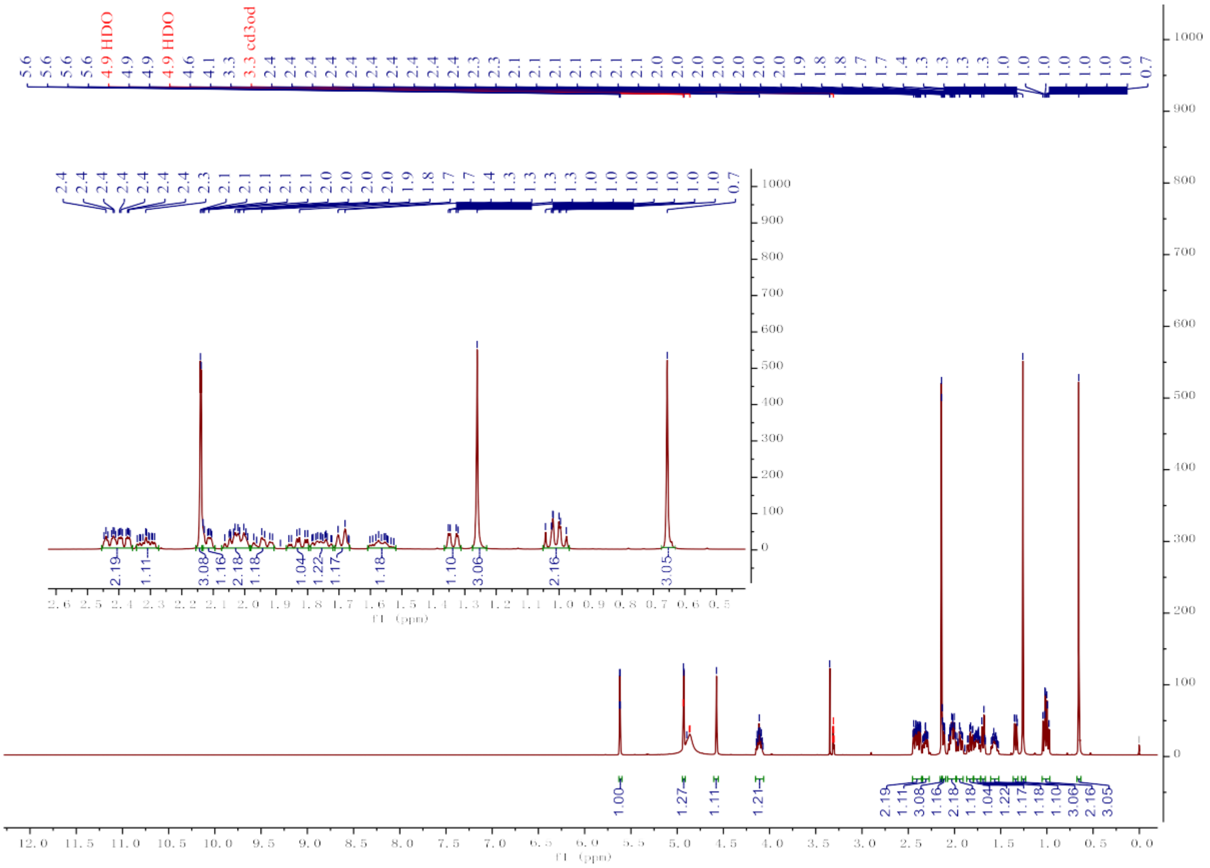

Figure S59. ^{13}C NMR of **9** in CD_3OD.

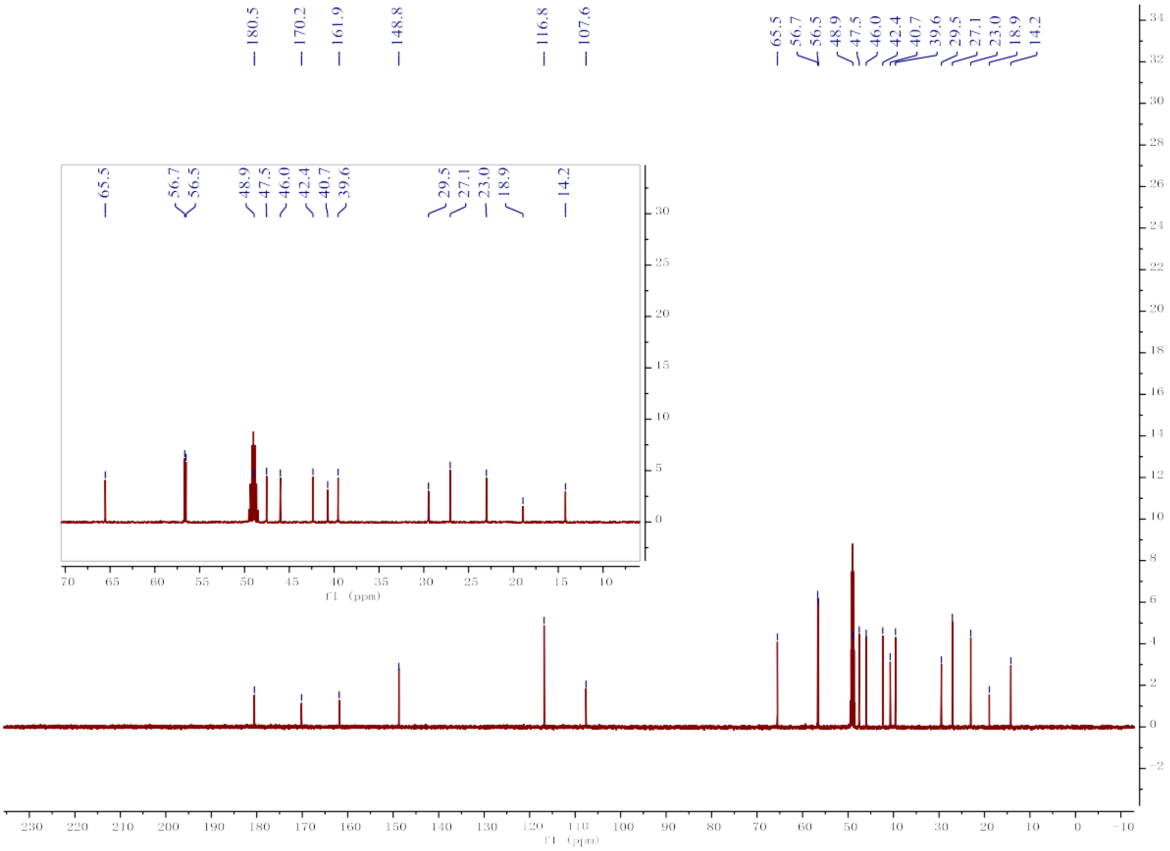

Figure S60. ^{1}H NMR of **10** in CD_3OD.

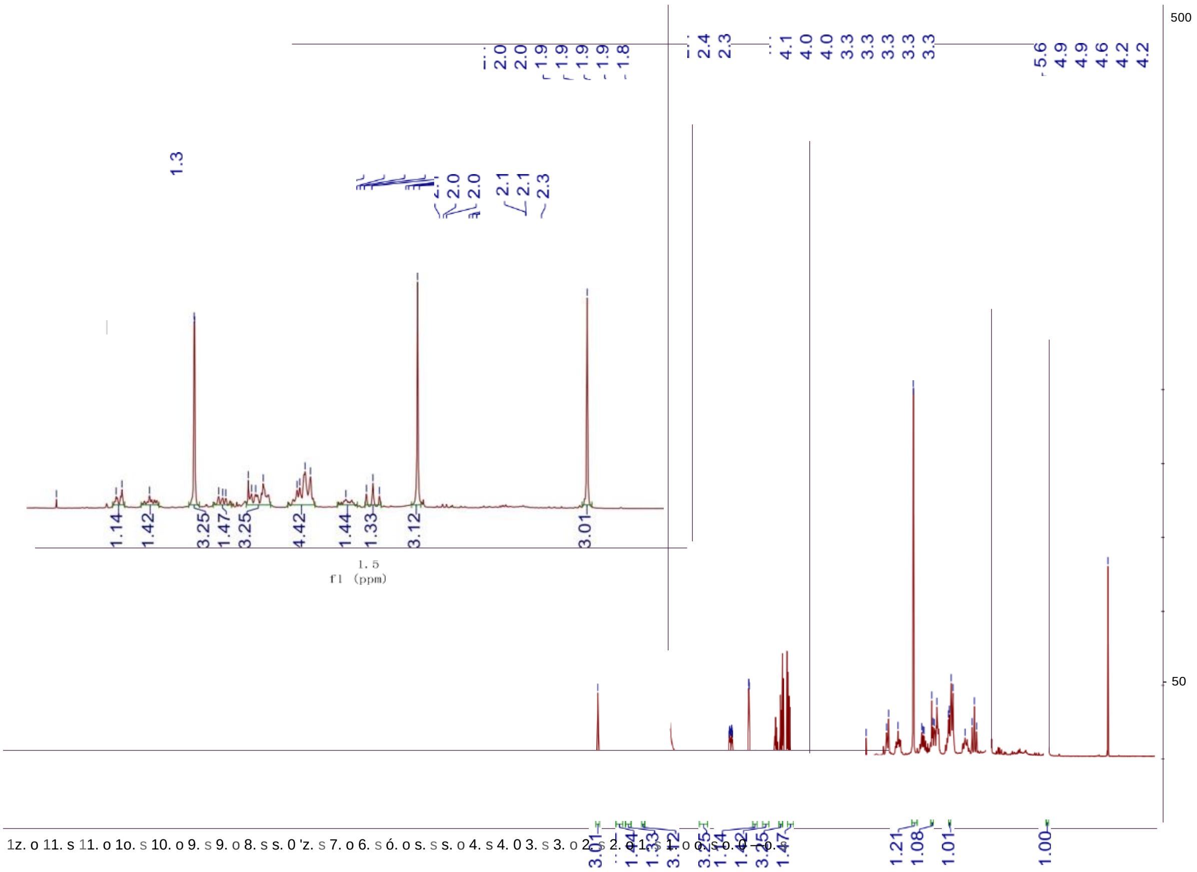

Figure S61. ^{13}C NMR of **10** in CD_3OD.

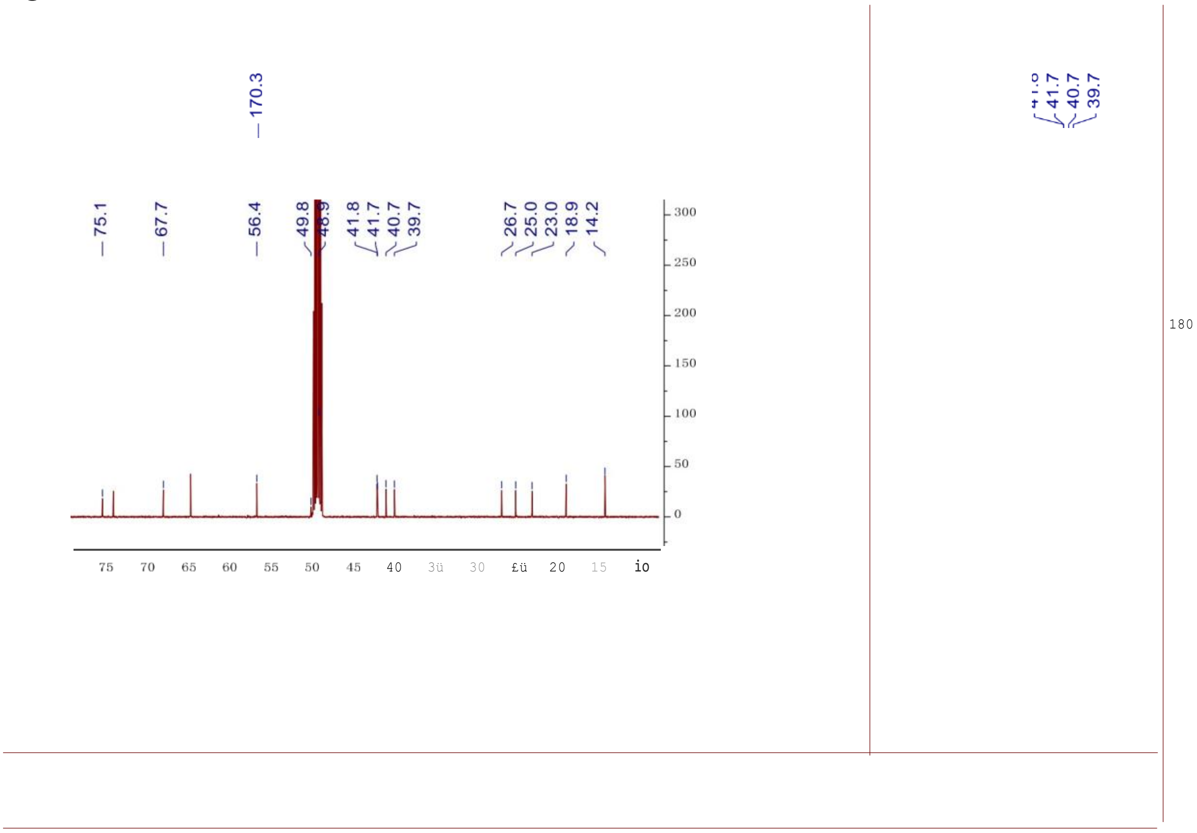

Figure S62. ^{1}H NMR of **11** in CD_3OD.

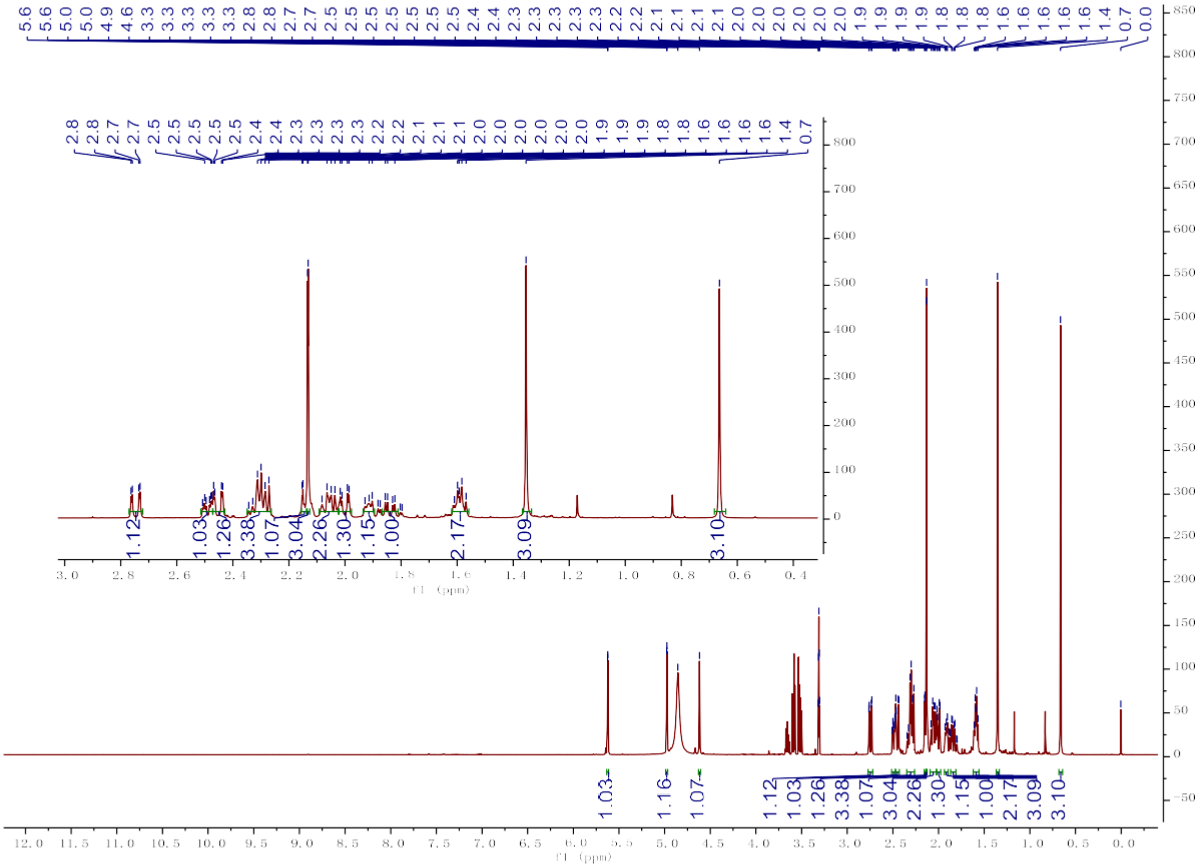

Figure S63. ^{13}C NMR of **11** in CD_3OD.

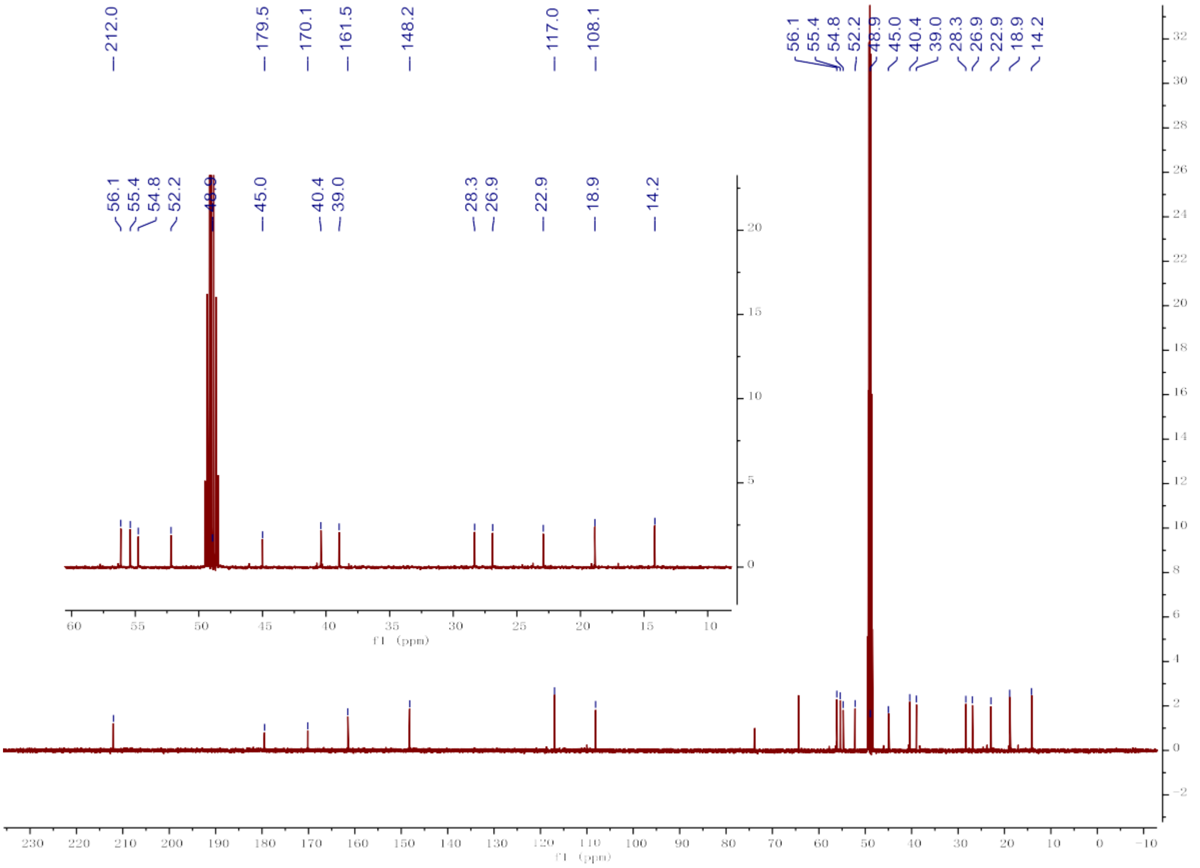

Figure S64. ^{1}H NMR of **12** in CD_3OD.

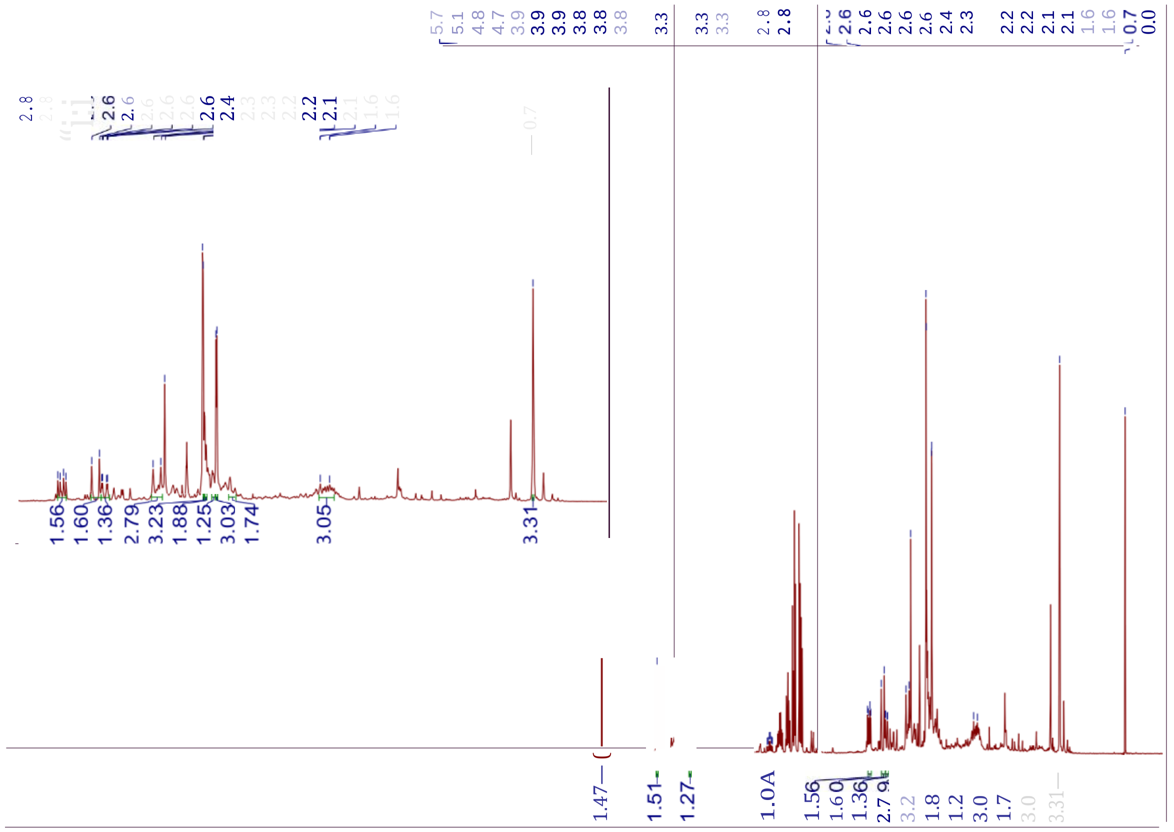

Figure S65. ^{13}C NMR of **12** in CD_3OD.

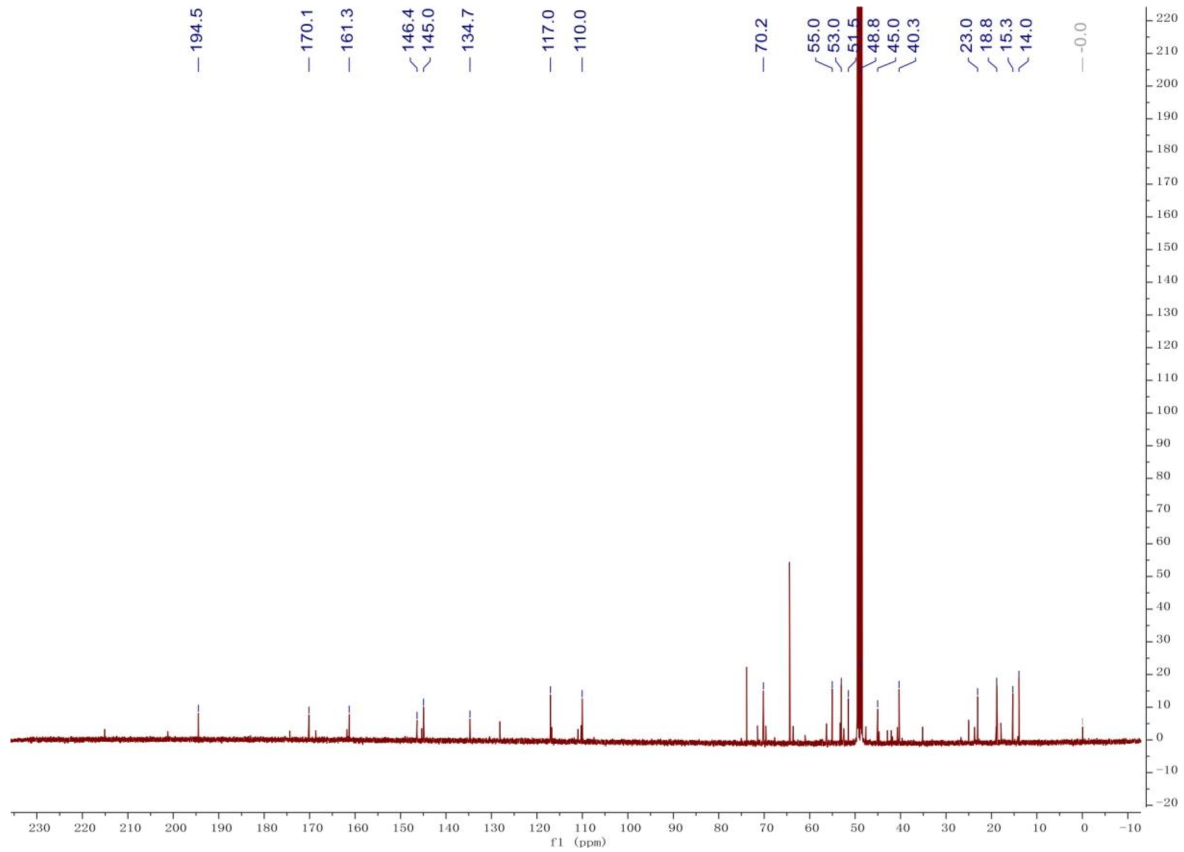

Figure S66. ^{1}H NMR of **13** in CD_3OD.

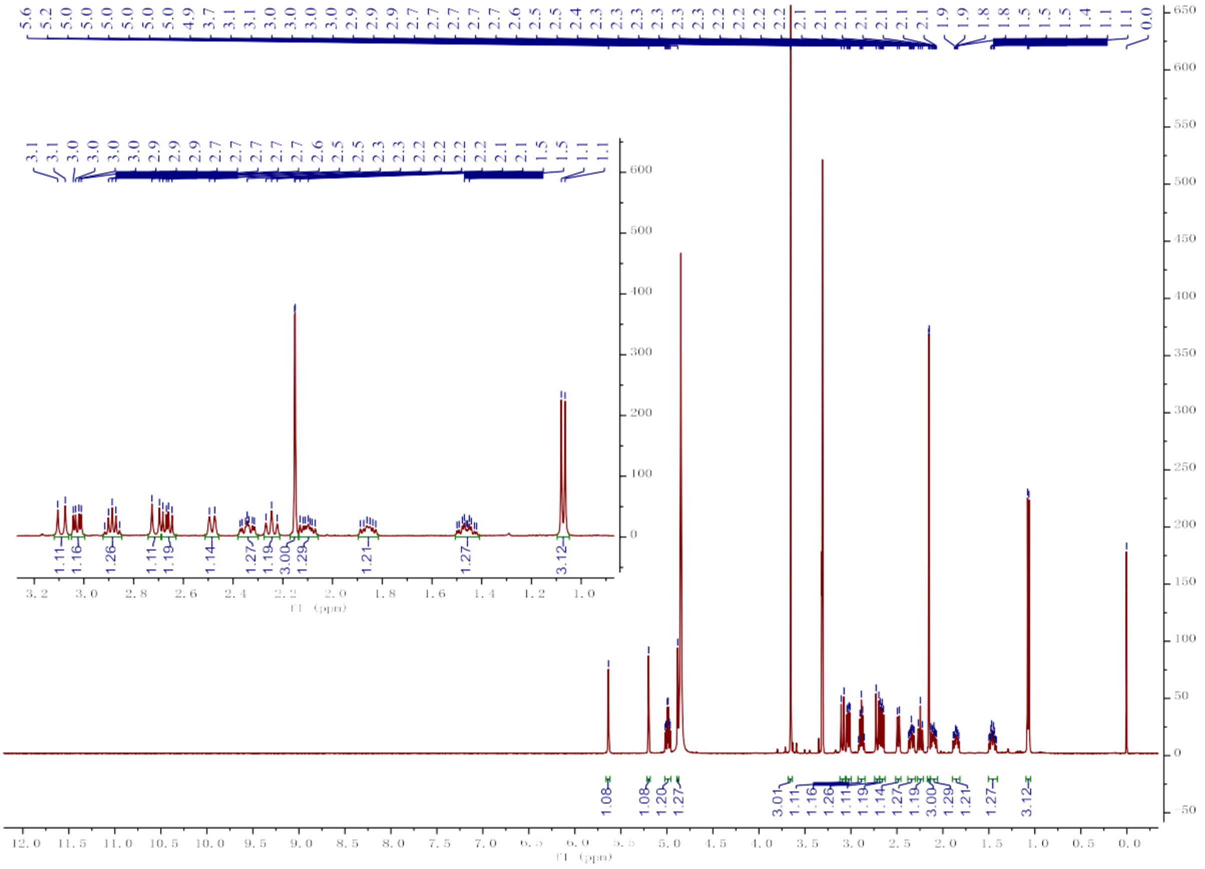

Figure S67. ^{13}C NMR of **13** in CD_3OD.

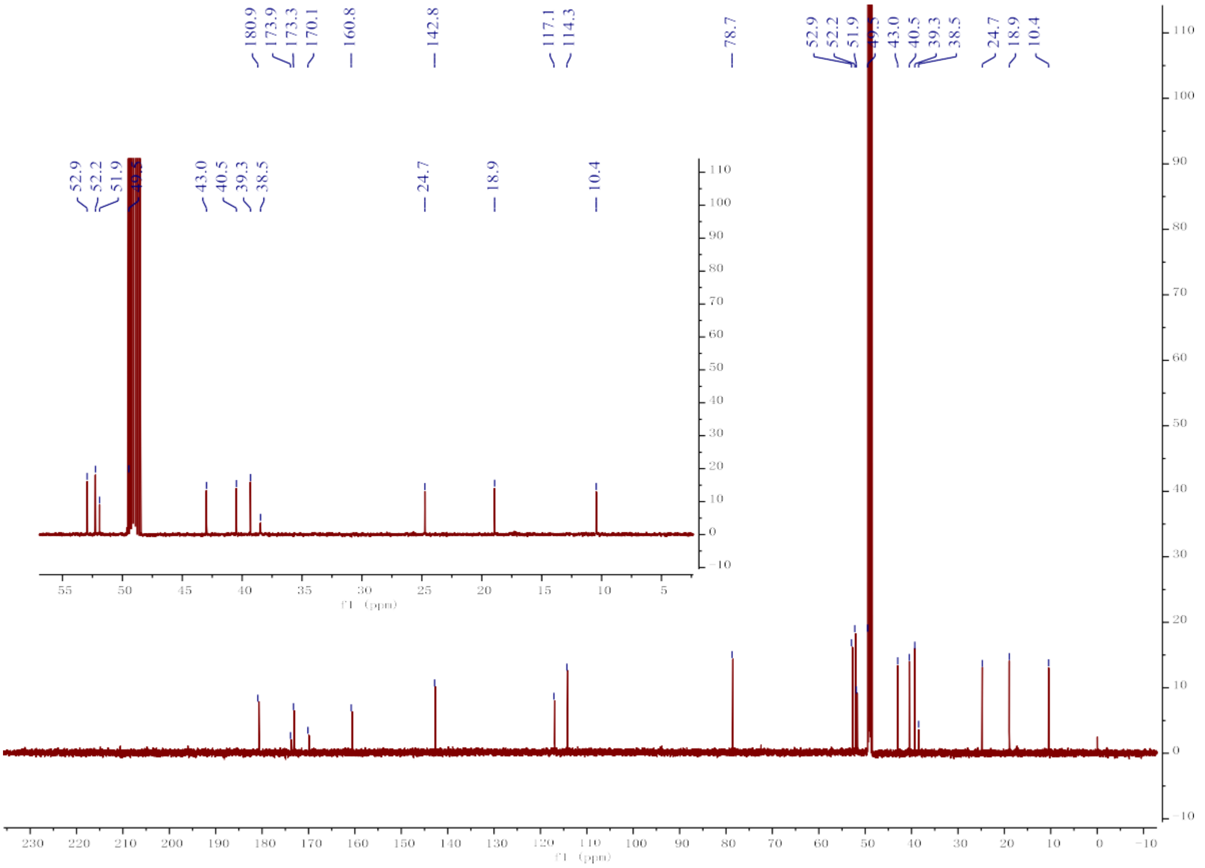

Figure S68. Main conformers of (2*R**,4*S**,5*R**,9*S**,10*R**)-**3** in ECD calculations and the energy analysis for optimized geometries of dominant conformers at B3LYP/6-31G(d)-GD3BJ level in the gas phase.

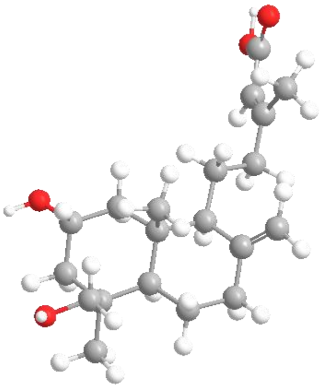	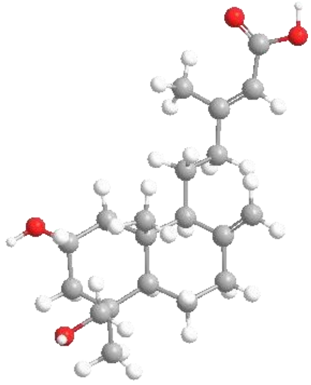	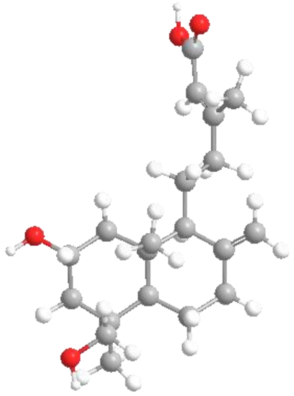	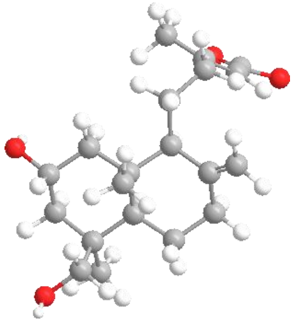
(2*R**,4*S**,5*R**,9*S**,10*R**)-**3A**	(2*R**,4*S**,5*R**,9*S**,10*R**)-**3B**	(2*R**,4*S**,5*R**,9*S**,10*R**)-**3C**	(2*R**,4*S**,5*R**,9*S**,10*R**)-**3D**
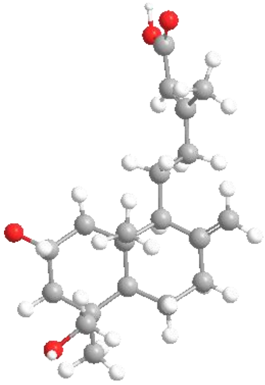	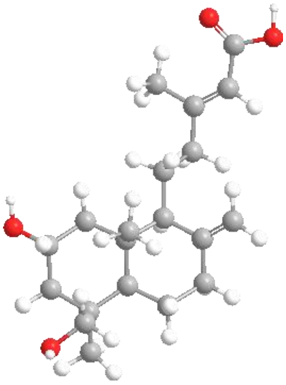	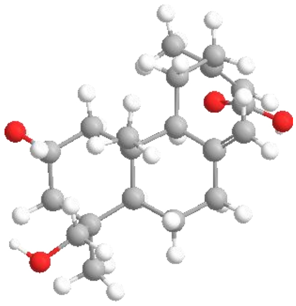	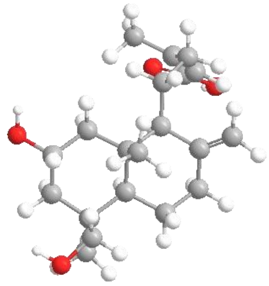
(2*R**,4*S**,5*R**,9*S**,10*R**)-**3E**	(2*R**,4*S**,5*R**,9*S**,10*R**)-**3F**	(2*R**,4*S**,5*R**,9*S**,10*R**)-**3G**	(2*R**,4*S**,5*R**,9*S**,10*R**)-**3H**
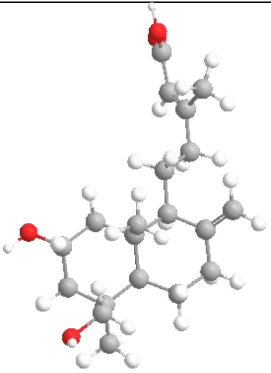	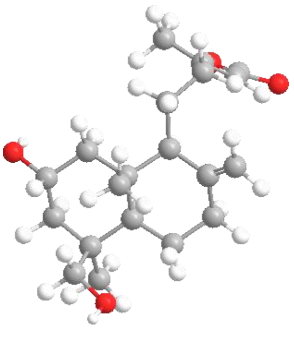	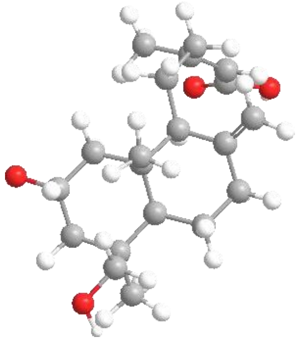	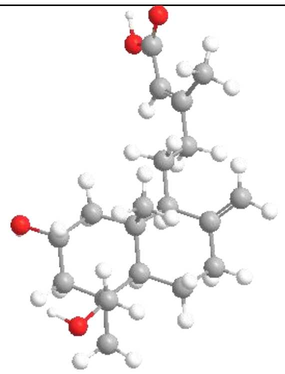
(2*R**,4*S**,5*R**,9*S**,10*R**)-**3I**	(2*R**,4*S**,5*R**,9*S**,10*R**)-**3J**	(2*R**,4*S**,5*R**,9*S**,10*R**)-**3K**	(2*R**,4*S**,5*R**,9*S**,10*R**)-**3L**
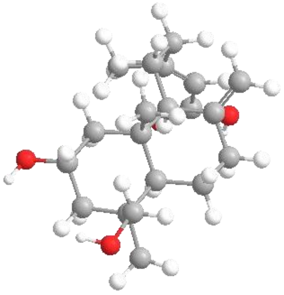	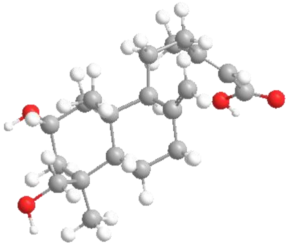		
(2*R**,4*S**,5*R**,9*S**,10*R**)-**3M**	(2*R**,4*S**,5*R**,9*S**,10*R**)-**3N**		

Continued

Energy analysis:

Species	*E′*=*E*+*ZPE*	*E*	*H*	*G*	*ΔG*	*ΔE*(kcal/mol)	*PE*%
**3A**	-1081.878860	-1081.853347	-1081.852403	-1081.934377	0.002713	1.702433	1.44%
**3B**	-1081.879043	-1081.853539	-1081.852595	-1081.934699	0.002391	1.500375	2.03%
**3C**	-1081.879732	-1081.854358	-1081.853414	-1081.935064	0.002026	1.271334	2.98%
**3D**	-1081.881048	-1081.855650	-1081.854706	-1081.936470	0.000620	0.389056	13.24%
**3E**	-1081.878262	-1081.852738	-1081.851794	-1081.933940	0.003150	1.976655	0.91%
**3F**	-1081.881685	-1081.856369	-1081.855425	-1081.937029	0.000061	0.038278	23.94%
**3G**	-1081.881034	-1081.855742	-1081.854798	-1081.936233	0.000857	0.537776	10.30%
**3H**	-1081.875604	-1081.850037	-1081.849093	-1081.931337	0.005753	3.610062	0.06%
**3I**	-1081.880545	-1081.855190	-1081.854245	-1081.935881	0.001209	0.758659	7.09%
**3J**	-1081.881763	-1081.856494	-1081.855550	-1081.937090	0	0	25.54%
**3K**	-1081.879436	-1081.853969	-1081.853025	-1081.934992	0.002098	1.316515	2.76%
**3L**	-1081.880741	-1081.855375	-1081.854431	-1081.936047	0.001043	0.654492	8.46%
**3M**	-1081.878896	-1081.853615	-1081.852671	-1081.934242	0.002848	1.787147	1.25%
**3N**	-1081.878860	-1081.853347	-1081.852403	-1081.934377	0.002713	1.702433	1.44%

E, *E′*, *H*, *G*: total energy, total energy with zero point energy (*ZPE*), enthalpy, and Gibbs free energy.

Figure S69. Main conformers of (5S*,6S*,9S*,10R*)-**4** in ECD calculations and the energy analysis for optimized geometries of dominant conformers at B3LYP/6-31G(d)-GD3BJ level in the gas phase.

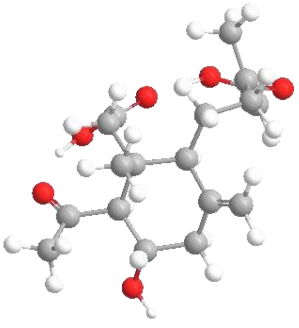	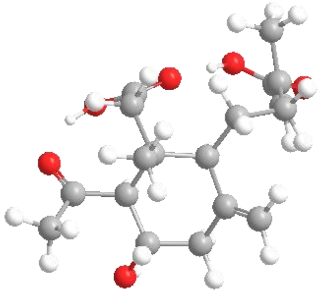	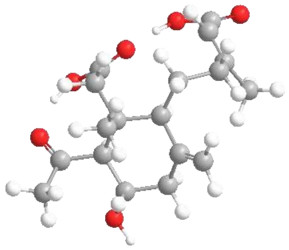	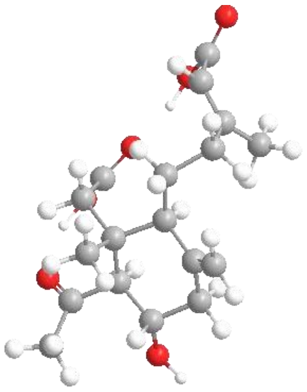
(5S*,6S*,9S*,10R*)-**4A**	(5S*,6S*,9S*,10R*)-**4B**	(5S*,6S*,9S*,10R*)-**4C**	(5S*,6S*,9S*,10R*)-**4D**
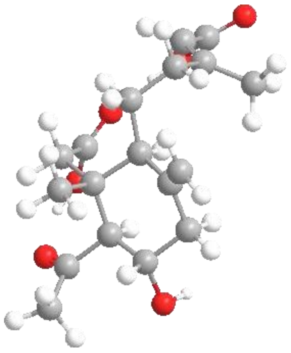			
(5S*,6S*,9S*,10R*)-**4E**			

Energy analysis:

Species	*E′=E+ZPE*	*E*	*H*	*G*	*ΔG*	*ΔE*(kcal/mol)	*PE*%
**4A**	-1152.592400	-1152.568190	-1152.567245	-1152.644168	0.000854	0.535893	15.36%
**4B**	-1152.592903	-1152.568743	-1152.567799	-1152.644605	0.000417	0.261671	24.41%
**4C**	-1152.592563	-1152.568277	-1152.567333	-1152.644509	0.000513	0.321912	22.05%
**4D**	-1152.586308	-1152.561675	-1152.560731	-1152.640050	0.004972	3.119977	0.20%
**4E**	-1152.593162	-1152.568934	-1152.567989	-1152.645022	0	0	37.98%

E, *E′*, *H*, *G*: total energy, total energy with zero point energy (*ZPE*), enthalpy, and Gibbs free energy.

Figure S70. Main conformers of (2*S**,5*S**,9*S**,10*R**)-**5** in ECD calculations and the energy analysis for optimized geometries of dominant conformers at B3LYP/6-31G(d)-GD3BJ level in the gas phase.

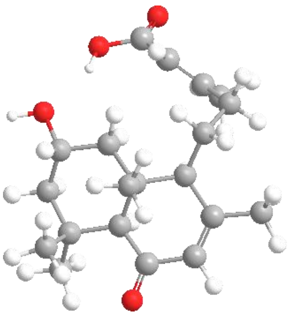	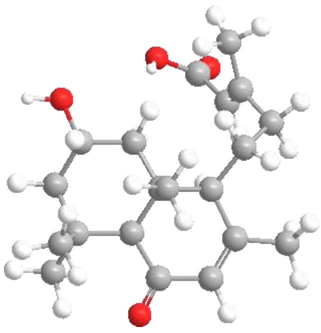	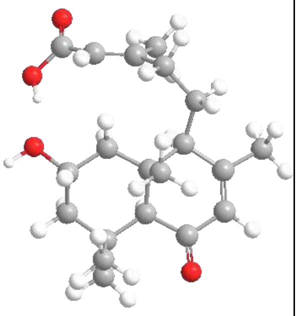	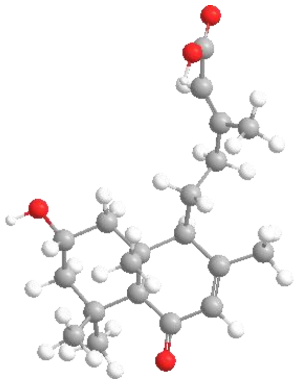
(2*S**,5*S**,9*S**,10*R**)-**5A**	(2*S**,5*S**,9*S**,10*R**)-**5B**	(2*S**,5*S**,9*S**,10*R**)-**5C**	(2*S**,5*S**,9*S**,10*R**)-**5D**
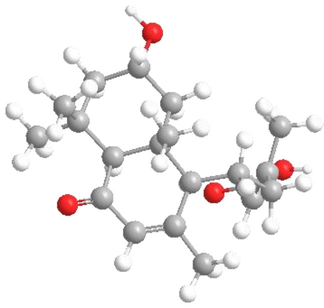	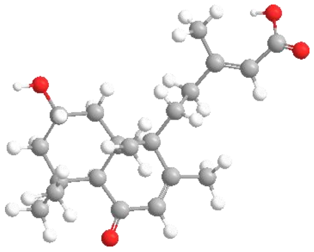	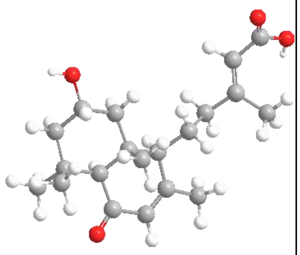	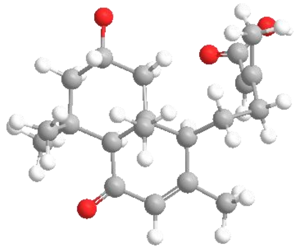
(2*S**,5*S**,9*S**,10*R**)-**5E**	(2*S**,5*S**,9*S**,10*R**)-**5F**	(2*S**,5*S**,9*S**,10*R**)-**5G**	(2*S**,5*S**,9*S**,10*R**)-**5H**
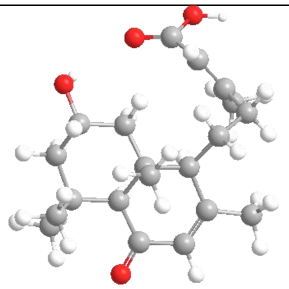			
(2*S**,5*S**,9*S**,10*R**)-**5I**			

Energy analysis:

Species	*E'*=*E*+*ZPE*	*E*	*H*	*G*	*ΔG*	*ΔE*(kcal/mol)	*PE*%
**5A**	-1080.717662	-1080.692943	-1080.691999	-1080.769658	0	0	86.88%
**5B**	-1080.715230	-1080.690514	-1080.689569	-1080.767185	0.002473	1.551831	6.32%
**5C**	-1080.715833	-1080.691458	-1080.690514	-1080.767059	0.002599	1.630897	5.53%
**5D**	-1080.706369	-1080.681167	-1080.680223	-1080.761145	0.008513	5.341988	0.01%
**5E**	-1080.709286	-1080.684143	-1080.683198	-1080.764062	0.005596	3.511543	0.23%
**5F**	-1080.706879	-1080.681663	-1080.680719	-1080.761749	0.007909	4.962973	0.02%
**5G**	-1080.706435	-1080.681248	-1080.680304	-1080.760873	0.008785	5.512671	0.01%
**5H**	-1080.712743	-1080.687952	-1080.687008	-1080.765247	0.004411	2.767944	0.81%
**5I**	-1080.712052	-1080.687422	-1080.686478	-1080.763871	0.005787	3.631397	0.19%

E, *E′*, *H*, *G*: total energy, total energy with zero point energy (*ZPE*), enthalpy, and Gibbs free energy.

Figure S71. Main conformers of (4*S**,5*R**,9*S**,10*R**)-**6** in ECD calculations and the energy analysis for optimized geometries of dominant conformers at B3LYP/6-31G(d)-GD3BJ level in the gas phase.

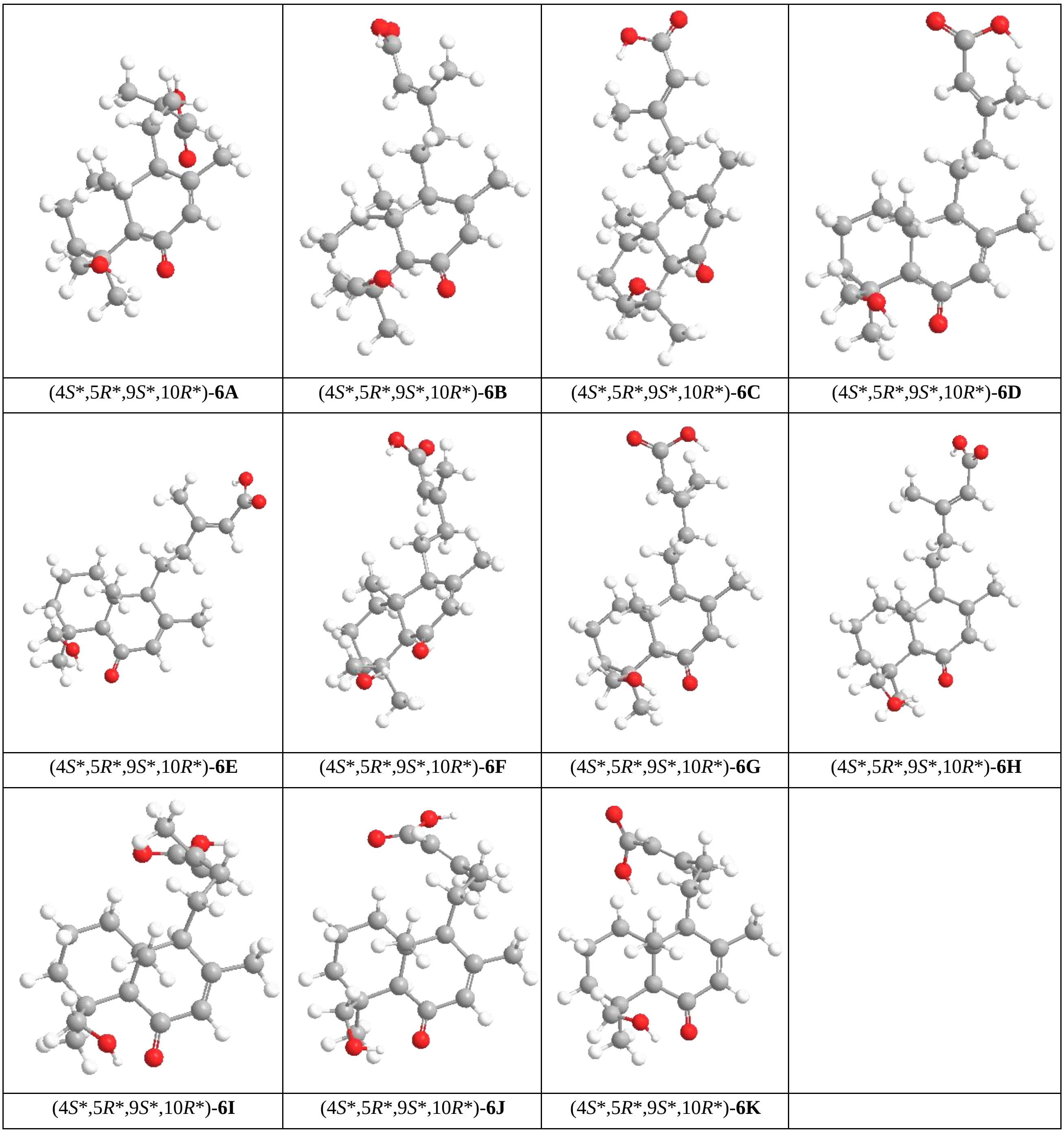

Energy analysis:

Species	*E'*=*E*+*ZPE*	*E*	*H*	*G*	*ΔG*	*ΔE*(kcal/mol)	*PE*%
**6A**	-1080.706544	-1080.682062	-1080.681118	-1080.759902	0.003619	2.270957	2.07%
**6B**	-1080.703611	-1080.679003	-1080.678059	-1080.757883	0.005638	3.537899	0.24%
**6C**	-1080.703810	-1080.679219	-1080.678275	-1080.758068	0.005453	3.421809	0.30%
**6D**	-1080.703715	-1080.679103	-1080.678159	-1080.757812	0.005709	3.582452	0.23%
**6E**	-1080.703681	-1080.679044	-1080.678100	-1080.758179	0.005342	3.352156	0.33%
**6F**	-1080.703487	-1080.678861	-1080.677917	-1080.757901	0.005620	3.526603	0.25%

**6G**	-1080.703210	-1080.678593	-1080.677649	-1080.757010	0.006511	4.085714	0.10%
**6H**	-1080.703756	-1080.679099	-1080.678155	-1080.758351	0.005170	3.244224	0.40%
**6I**	-1080.709757	-1080.685212	-1080.684267	-1080.763521	0	0	95.83%
**6J**	-1080.704489	-1080.680105	-1080.679161	-1080.757518	0.006003	3.766940	0.17%
**6K**	-1080.703570	-1080.679112	-1080.678167	-1080.756959	0.006562	4.117717	0.09%

E, E', H, G: total energy, total energy with zero point energy (*ZPE*), enthalpy, and Gibbs free energy.

Figure S72. Main conformers of (2*R**,4*S**,5*R**,9*S**,10*R**)-**7** in ECD calculations and the energy analysis for optimized geometries of dominant conformers at B3LYP/6-31G(d)-GD3BJ level in the gas phase.

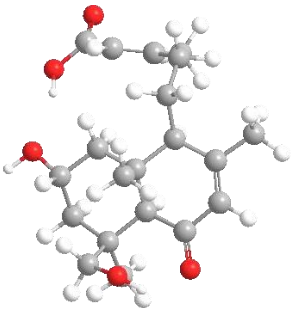	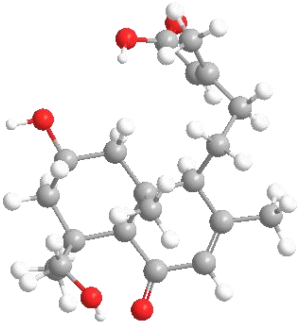	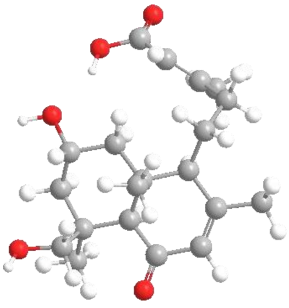	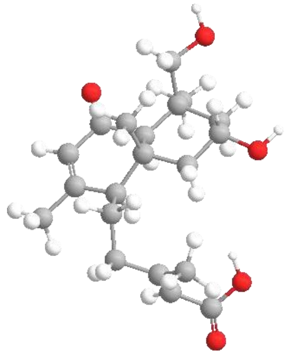
(2*R**,4*S**,5*R**,9*S**,10*R**)-**7A**	(2*R**,4*S**,5*R**,9*S**,10*R**)-**7B**	(2*R**,4*S**,5*R**,9*S**,10*R**)-**7C**	(2*R**,4*S**,5*R**,9*S**,10*R**)-**7D**
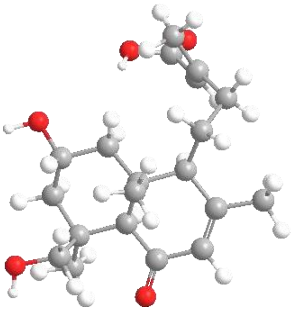	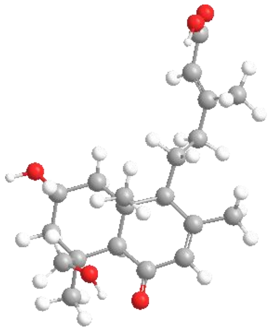	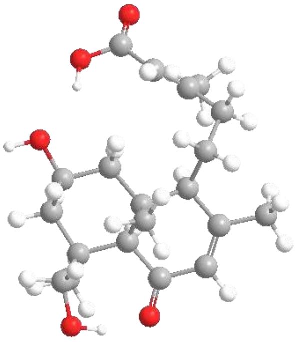	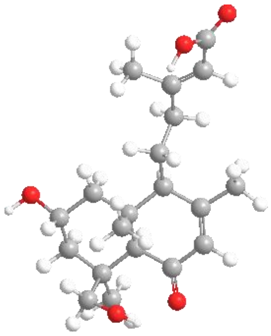
(2*R**,4*S**,5*R**,9*S**,10*R**)-**7E**	(2*R**,4*S**,5*R**,9*S**,10*R**)-**7F**	(2*R**,4*S**,5*R**,9*S**,10*R**)-**7G**	(2*R**,4*S**,5*R**,9*S**,10*R**)-**7H**
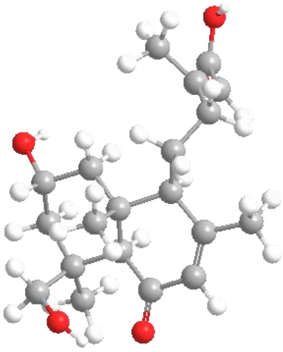	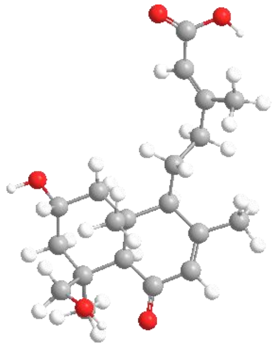	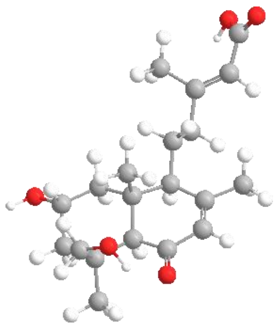	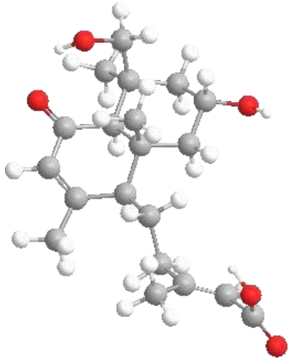
(2*R**,4*S**,5*R**,9*S**,10*R**)-**7I**	(2*R**,4*S**,5*R**,9*S**,10*R**)-**7J**	(2*R**,4*S**,5*R**,9*S**,10*R**)-**7K**	(2*R**,4*S**,5*R**,9*S**,10*R**)-**7L**
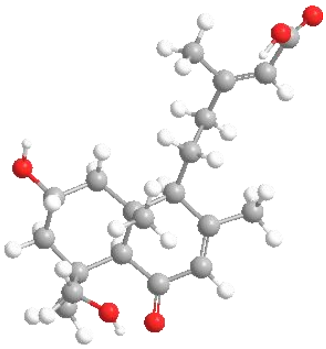	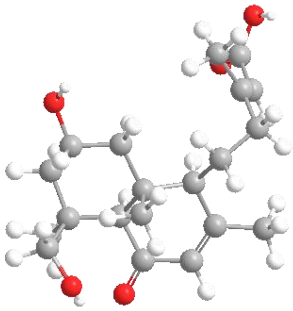	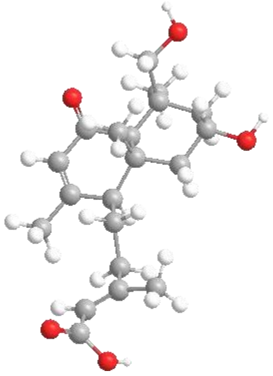	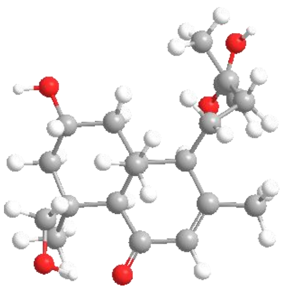
(2*R**,4*S**,5*R**,9*S**,10*R**)-**7M**	(2*R**,4*S**,5*R**,9*S**,10*R**)-**7N**	(2*R**,4*S**,5*R**,9*S**,10*R**)-**7O**	(2*R**,4*S**,5*R**,9*S**,10*R**)-**7P**

Continued

Energy analysis:

Species	*E′*=*E*+*ZPE*	*E*	*H*	*G*	*ΔG*	*ΔE*(kcal/mol)	*PE*%
**7A**	-1155.922441	-1155.896965	-1155.896021	-1155.975476	0.000342	0.214608	38.16%
**7B**	-1155.920260	-1155.894802	-1155.893858	-1155.973178	0.002640	1.656625	3.34%
**7C**	-1155.919025	-1155.893044	-1155.892100	-1155.972804	0.003014	1.891314	2.25%
**7D**	-1155.916552	-1155.890566	-1155.889621	-1155.970299	0.005519	3.463225	0.16%
**7E**	-1155.917810	-1155.891983	-1155.891039	-1155.971399	0.004419	2.772964	0.51%
**7F**	-1155.911109	-1155.885179	-1155.884235	-1155.966890	0.008928	5.602405	0.00%
**7G**	-1155.922832	-1155.897425	-1155.896481	-1155.975818	0	0	54.83%
**7H**	-1155.911435	-1155.885572	-1155.884627	-1155.966966	0.008852	5.554714	0.00%
**7I**	-1155.918138	-1155.892645	-1155.891701	-1155.971529	0.004289	2.691388	0.58%
**7J**	-1155.911082	-1155.885170	-1155.884226	-1155.966547	0.009271	5.817641	0.00%
**7K**	-1155.911489	-1155.885557	-1155.884612	-1155.967380	0.008438	5.294925	0.01%
**7L**	-1155.910551	-1155.884611	-1155.883667	-1155.966361	0.009457	5.934357	0.00%
**7M**	-1155.910572	-1155.884697	-1155.883753	-1155.966123	0.009695	6.083705	0.00%
**7N**	-1155.912793	-1155.886969	-1155.886025	-1155.968347	0.007471	4.688123	0.02%
**7O**	-1155.908053	-1155.881551	-1155.880606	-1155.964715	0.011103	6.967238	0.00%
**7P**	-1155.914765	-1155.888920	-1155.887976	-1155.970102	0.005716	3.586844	0.13%

E, *E′*, *H*, *G*: total energy, total energy with zero point energy (*ZPE*), enthalpy, and Gibbs free energy.

Supplementary S1. The Z-matrices of (2*R**,4*S**,5*R**,9*S**,10*R**)-**3** optimized at B3LYP/6-31G(d)-GD3BJ level by Gaussian.

(2*R**,4*S**,5*R**,9*S**,10*R**)-**3A**			
C	-2.5474	-0.0415	1.7368
C	-2.3198	-1.3761	2.4323
C	-0.8514	-1.6108	2.8648
C	0.0881	-1.3701	1.6265
C	-0.1390	-0.0335	0.7998
C	-1.6550	0.1045	0.5038
C	1.5869	-1.6127	1.9169
C	2.4132	-1.6394	0.6294
C	2.1650	-0.4045	-0.2017
C	0.6877	-0.1857	-0.5536
C	0.6029	1.6028	-4.0469
C	0.8994	0.5260	-3.0183
C	0.4146	0.9196	-1.6108
C	0.3299	1.2521	1.5279
C	-0.4824	-0.7552	4.0999
C	-0.7675	-3.1020	3.3002
C	-0.3330	1.3880	-4.9933
C	3.1879	0.4051	-0.5257
C	1.4365	2.8511	-3.9255
O	-3.9054	0.0334	1.3035
O	-1.3643	-1.0293	5.1860
C	-0.7594	2.3483	-6.0273
O	-0.4753	3.5236	-6.1383
O	-1.5758	1.7343	-6.9011
H	-2.3907	0.7889	2.4313
H	-3.0025	-1.4616	3.2877
H	-2.6363	-2.1774	1.7485
H	-0.1953	-2.1737	0.9253

(2*R**,4*S**,5*R**,9*S**,10*R**)-**3B**		
-3.0848	-0.7996	0.2228
-2.9761	-2.2397	-0.2574
-1.7116	-2.9728	0.2542
-0.4477	-2.0958	-0.0738
-0.5134	-0.5605	0.3263
-1.8586	0.0185	-0.1841
0.8918	-2.7389	0.3521
2.0888	-1.9876	-0.2343
2.0144	-0.5142	0.0829
0.7166	0.1537	-0.3920
1.6932	3.8677	-1.2041
1.6406	2.3570	-1.3484
0.7042	1.7053	-0.3141
-0.4211	-0.2956	1.8507
-1.8394	-3.3426	1.7510
-1.6405	-4.3075	-0.5419
2.8291	4.4602	-0.7838
3.0117	0.0792	0.7612
0.4305	4.5947	-1.5853
-4.2308	-0.1933	-0.3744
-2.9970	-4.1435	1.9765
3.0243	5.9030	-0.5533
2.1983	6.7927	-0.5494
4.3243	6.1471	-0.3122
-3.2355	-0.7626	1.3055
-3.8882	-2.7849	0.0183
-2.9816	-2.2358	-1.3573
-0.4165	-2.0871	-1.1768

(2*R**,4*S**,5*R**,9*S**,10*R**)-**3C**		
-2.9689	-0.7562	0.3710
-2.8941	-2.1813	-0.1590
-1.6358	-2.9548	0.3064
-0.3607	-2.0881	-0.0082
-0.3931	-0.5658	0.4427
-1.7346	0.0530	-0.0288
0.9726	-2.7686	0.3773
2.1750	-2.0197	-0.2014
2.1311	-0.5559	0.1628
0.8391	0.1490	-0.2711
1.7832	3.8785	-1.0516
1.7979	2.3645	-1.1646
0.8545	1.6976	-0.1466
-0.2737	-0.3521	1.9733
-1.7504	-3.3699	1.7929
-1.5964	-4.2581	-0.5414
1.2254	4.6196	-2.0300
3.1477	-0.0022	0.8459
2.4454	4.4350	0.1813
-4.1129	-0.1135	-0.1907
-2.9241	-4.1414	2.0367
1.0979	6.0882	-2.0425
1.3260	6.8800	-1.1507
0.6525	6.4900	-3.2456
-3.1026	-0.7511	1.4566
-3.8117	-2.7191	0.1129
-2.9166	-2.1399	-1.2580
-0.3436	-2.0424	-1.1106

(2*R**,4*S**,5*R**,9*S**,10*R**)-**3D**		
-2.9868	-0.1167	0.8082
-2.8894	-1.5722	0.3699
-1.6290	-2.3011	0.9007
-0.3587	-1.4375	0.5587
-0.4157	0.1059	0.9266
-1.7517	0.6833	0.3915
0.9771	-2.0800	0.9993
2.1809	-1.3467	0.4033
2.1133	0.1317	0.6983
0.8239	0.7964	0.2009
1.5238	2.7177	-2.2054
1.7970	3.0244	-0.7437
0.8172	2.3512	0.2385
-0.3323	0.3988	2.4458
-1.7681	-2.6362	2.4051
-1.5588	-3.6520	0.1321
2.4226	2.0099	-2.9189
3.1051	0.7276	1.3819
0.2343	3.2732	-2.7495
-4.1388	0.4810	0.2133
-2.8919	-3.4832	2.6319
2.2753	1.5865	-4.3226
1.2869	1.6238	-5.0271
3.4444	1.0979	-4.7708
-3.1383	-0.0574	1.8893
-3.8063	-2.0998	0.6633
-2.8942	-1.6027	-0.7296
-0.3250	-1.4517	-0.5442

(2*R**,4*S**,5*R**,9*S**,10*R**)-**3E**		
-2.9598	-0.8698	0.3045
-2.8187	-2.3173	-0.1480
-1.5449	-3.0177	0.3889
-0.2944	-2.1168	0.0705
-0.3967	-0.5794	0.4513
-1.7426	-0.0328	-0.0919
1.0519	-2.7276	0.5234
2.2426	-1.9590	-0.0531
2.1338	-0.4846	0.2493
0.8317	0.1522	-0.2524
1.6591	3.8814	-1.1583
1.7403	2.3659	-1.2026
0.7862	1.7041	-0.1912
-0.3377	-0.2988	1.9744
-1.6920	-3.3732	1.8880
-1.4300	-4.3578	-0.3933
1.1191	4.5560	-2.1931
3.1067	0.1336	0.9406
2.2398	4.5180	0.0771
-4.1217	-0.2974	-0.2958
-2.7953	-4.2523	2.0920
0.9334	6.0162	-2.2765
1.0888	6.8541	-1.4116
0.5257	6.3467	-3.5141
-3.1232	-0.8259	1.3847
-3.7241	-2.8725	0.1297
-2.8108	-2.3361	-1.2477
-0.2463	-2.1195	-1.0320

H	-1.9567	-0.6437	-0.2425	-1.8351	0.1005	-1.2798	-1.7257	0.1693	-1.1216	-1.7181	0.7388	-0.7055	-1.6995	0.0348	-1.1878
H	-1.8780	1.0764	0.0461	-2.0082	1.0425	0.1800	-1.8596	1.0676	0.3695	-1.8962	1.7167	0.7301	-1.9185	0.9929	0.2554
H	1.7340	-2.5735	2.4201	0.9531	-3.7747	0.0037	1.0097	-3.7935	-0.0054	1.0333	-3.1224	0.6704	1.1394	-3.7659	0.1883
H	1.9809	-0.8466	2.5935	0.9763	-2.7750	1.4436	1.0713	-2.8420	1.4658	1.0591	-2.0957	2.0916	1.1211	-2.7489	1.6164
H	3.4733	-1.7459	0.8893	3.0130	-2.4376	0.1478	3.0960	-2.4976	0.1535	3.1013	-1.7960	0.7953	3.1697	-2.3869	0.3468
H	2.1488	-2.5245	0.0372	2.1093	-2.1153	-1.3239	2.1788	-2.1131	-1.2947	2.2038	-1.4917	-0.6842	2.2817	-2.0964	-1.1411
H	0.3452	-1.1198	-1.0256	0.6297	-0.0765	-1.4654	0.7325	-0.0472	-1.3494	0.7361	0.5292	-0.8619	0.7673	-0.0909	-1.3245
H	0.4139	-0.4158	-3.3060	1.3001	2.1086	-2.3619	1.4973	2.0577	-2.1750	2.8286	2.7622	-0.4851	1.4919	2.0032	-2.2087
H	1.9745	0.3186	-3.0301	2.6498	1.9388	-1.2684	2.8291	2.0157	-1.0456	1.7365	4.1117	-0.6037	2.7796	2.0673	-1.0300
H	0.8648	1.8701	-1.3041	0.9591	2.0511	0.6942	1.1157	2.0029	0.8725	1.0388	2.7128	1.2491	0.9997	2.0588	0.8230
H	-0.6613	1.1047	-1.6898	-0.3040	2.0781	-0.5168	-0.1462	2.0985	-0.3366	-0.1818	2.7302	0.0036	-0.2211	2.0586	-0.4315
H	1.3453	1.1704	1.9239	-1.3269	-0.5750	2.3878	-1.1768	-0.6312	2.5151	-1.2390	0.1248	2.9837	-1.2397	-0.6080	2.5007
H	0.3170	2.1136	0.8518	0.4142	-0.8181	2.3236	0.5582	-0.9054	2.4165	0.5023	-0.1126	2.9321	0.5089	-0.7868	2.4640
H	-0.3209	1.5357	2.3543	-0.2903	0.7721	2.0566	-0.1193	0.7057	2.2116	-0.2060	1.4705	2.6329	-0.2488	0.7747	2.1727
H	-0.5536	0.3122	3.9091	-1.9397	-2.4713	2.3927	-1.8204	-2.5133	2.4573	-1.9200	-1.7517	3.0186	-1.8745	-2.4999	2.5094
H	0.5354	-0.9736	4.4390	-0.9707	-3.9167	2.0893	-0.8884	-3.9628	2.1162	-0.8832	-3.1626	2.7757	-0.7976	-3.8801	2.2632
H	0.1931	-3.3368	3.7696	-0.8664	-4.9754	-0.1508	-0.8418	-4.9607	-0.1736	-0.7955	-4.3179	0.5473	-0.6538	-5.0076	0.0233
H	-1.5490	-3.3513	4.0276	-2.5897	-4.8540	-0.4930	-2.5602	-4.7799	-0.5208	-2.5131	-4.1897	0.1779	-2.3700	-4.9212	-0.3642
H	-0.8973	-3.7747	2.4449	-1.4261	-4.1285	-1.6016	-1.3721	-4.0435	-1.5925	-1.3289	-3.4961	-0.9280	-1.1926	-4.1841	-1.4490
H	-0.8692	0.4436	-5.0212	3.7032	3.8560	-0.5577	0.7922	4.1325	-2.8990	3.3416	1.6703	-2.4490	0.7486	4.0150	-3.0592
H	3.0590	1.3212	-1.0908	2.9918	1.1259	1.0429	3.1500	1.0347	1.1625	3.0857	1.7761	1.6560	3.0613	1.1813	1.2154
H	4.2057	0.1789	-0.2192	3.8914	-0.4774	1.0731	4.0217	-0.5834	1.1276	3.9774	0.1694	1.7114	3.9917	-0.4030	1.2723
H	2.4535	2.6006	-3.6038	0.6069	5.6267	-1.8957	2.8222	5.4507	0.0436	0.2545	3.4224	-3.8312	2.5850	5.5405	-0.0902
H	1.5459	3.3866	-4.8710	-0.0491	4.1028	-2.4389	1.7475	4.4289	1.0240	-0.6002	2.6103	-2.5010	1.5025	4.5221	0.8857
H	1.0016	3.5288	-3.1846	-0.2760	4.5941	-0.7496	3.3154	3.8272	0.4540	0.0306	4.2578	-2.3143	3.1174	3.9576	0.4181
H	-4.4669	-0.0847	2.0894	-5.0032	-0.7337	-0.1327	-4.8956	-0.6126	0.1019	-4.0744	0.3660	-0.7504	-4.0495	-0.4064	-1.2592
H	-1.0443	-0.5191	5.9502	-2.9883	-4.4014	2.9146	-2.7561	-5.0444	1.7172	-2.9374	-3.6513	3.5888	-2.8484	-4.4309	3.0467
H	-1.8066	2.4445	-7.5352	4.3509	7.1165	-0.1726	0.5953	7.4637	-3.1536	3.2473	0.8463	-5.6969	0.4272	7.3205	-3.4689

(2*R**,4*S**,5*R**,9*S**,10*R**)-**3F**			
C	-3.1030	-0.7885	0.2104
C	-3.0106	-2.2323	-0.2615
C	-1.7534	-2.9797	0.2495
C	-0.4798	-2.1160	-0.0786
C	-0.5285	-0.5809	0.3218
C	-1.8649	0.0140	-0.1930
C	0.8530	-2.7742	0.3467
C	2.0588	-2.0357	-0.2373
C	2.0001	-0.5625	0.0846
C	0.7117	0.1201	-0.3925
C	1.7224	3.8255	-1.2015
C	1.6668	2.3145	-1.3404
C	0.7165	1.6717	-0.3133
C	-0.4385	-0.3196	1.8467
C	-1.8861	-3.3471	1.7470
C	-1.6945	-4.3144	-0.5477
C	2.8520	4.4159	-0.7619
C	3.0015	0.0174	0.7687
C	0.4708	4.5563	-1.6108
O	-4.2492	-0.1949	-0.3994
O	-3.0206	-4.1809	1.9672
C	3.0452	5.8591	-0.5324
O	2.2176	6.7475	-0.5366
O	4.3428	6.1041	-0.2808
H	-3.2643	-0.7539	1.2919
H	-3.9288	-2.7642	0.0204
H	-3.0189	-2.2349	-1.3616
H	-0.4484	-2.1071	-1.1816
H	-1.8376	0.0951	-1.2886

(2*R**,4*S**,5*R**,9*S**,10*R**)-**3G**		
-2.5212	0.7270	1.6165
-2.2666	-0.6009	2.3164
-0.7926	-0.8022	2.7464
0.1323	-0.5530	1.4961
-0.1212	0.7767	0.6650
-1.6414	0.8888	0.3764
1.6378	-0.7760	1.7699
2.4507	-0.7969	0.4735
2.1798	0.4355	-0.3545
0.6975	0.6314	-0.6946
0.2695	0.1607	-3.7886
0.8961	1.4016	-3.1780
0.3961	1.7274	-1.7561
0.3333	2.0703	1.3866
-0.4297	0.0731	3.9714
-0.6747	-2.2879	3.1913
1.0368	-0.9176	-4.0468
3.1885	1.2617	-0.6802
-1.2040	0.2609	-4.0818
-3.8921	0.7917	1.2282
-1.2106	-0.2625	5.1159
0.5656	-2.2077	-4.5801
-0.5748	-2.6074	-4.7010
1.6206	-2.9632	-4.9298
-2.3644	1.5558	2.3121
-2.9500	-0.7001	3.1689
-2.5636	-1.4117	1.6348
-0.1475	-1.3636	0.8016
-1.9333	0.1345	-0.3675

(2*R**,4*S**,5*R**,9*S**,10*R**)-**3H**		
-2.5226	0.7261	1.6335
-2.2709	-0.6041	2.3301
-0.7961	-0.8191	2.7510
0.1204	-0.5790	1.4925
-0.1286	0.7525	0.6641
-1.6497	0.8774	0.3869
1.6265	-0.8170	1.7526
2.4290	-0.8412	0.4498
2.1620	0.3972	-0.3703
0.6792	0.6017	-0.7017
0.2757	0.1320	-3.7982
0.8820	1.3808	-3.1837
0.3771	1.6988	-1.7617
0.3413	2.0409	1.3853
-0.4183	0.0575	3.9713
-0.6863	-2.3049	3.1960
1.0610	-0.9332	-4.0570
3.1732	1.2208	-0.6949
-1.1987	0.2101	-4.0929
-3.8962	0.7665	1.2516
-1.1518	-0.2986	5.1407
0.6169	-2.2290	-4.6006
-0.5149	-2.6351	-4.7686
1.6890	-2.9818	-4.9019
-2.3655	1.5550	2.3295
-2.9499	-0.7007	3.1864
-2.5794	-1.4110	1.6487
-0.1730	-1.3870	0.8004
-1.9565	0.1249	-0.3528

(2*R**,4*S**,5*R**,9*S**,10*R**)-**3I**		
-2.5637	-0.0391	1.7159
-2.3539	-1.3769	2.4109
-0.8891	-1.6305	2.8448
0.0548	-1.4012	1.6076
-0.1546	-0.0616	0.7811
-1.6684	0.0959	0.4836
1.5501	-1.6627	1.8996
2.3778	-1.6986	0.6131
2.1456	-0.4602	-0.2175
0.6715	-0.2241	-0.5715
0.6085	1.5630	-4.0655
0.8969	0.4848	-3.0359
0.4124	0.8835	-1.6297
0.3298	1.2178	1.5100
-0.5104	-0.7802	4.0806
-0.8247	-3.1228	3.2797
-0.3113	1.3443	-5.0273
3.1784	0.3378	-0.5390
1.4433	2.8093	-3.9380
-3.9202	0.0541	1.2816
-1.3974	-1.0426	5.1654
-0.7656	2.2532	-6.0917
-1.2847	1.8835	-7.1303
-0.6359	3.5539	-5.7993
-2.3967	0.7887	2.4110
-3.0386	-1.4542	3.2655
-2.6799	-2.1737	1.7262
-0.2379	-2.2010	0.9059
-1.9790	-0.6484	-0.2630

(2*R**,4*S**,5*R**,9*S**,10*R**)-**3J**		
-3.0103	-0.1336	0.7650
-2.9007	-1.5892	0.3324
-1.6578	-2.3161	0.9038
-0.3769	-1.4551	0.5755
-0.4413	0.0941	0.9230
-1.7719	0.6674	0.3675
0.9657	-2.0795	1.0203
2.1602	-1.3514	0.4012
2.0934	0.1283	0.6908
0.7999	0.7879	0.2001
1.4605	2.7296	-2.2064
1.7595	3.0205	-0.7464
0.7908	2.3429	0.2437
-0.3729	0.3966	2.4401
-1.9012	-2.6404	2.3984
-1.5674	-3.6767	0.1562
2.3404	2.0181	-2.9397
3.0899	0.7267	1.3655
0.1695	3.3038	-2.7263
-4.1591	0.4477	0.1497
-0.8293	-3.3754	2.9675
2.1655	1.6079	-4.3440
1.1644	1.6566	-5.0297
3.3238	1.1182	-4.8181
-3.1794	-0.0697	1.8433
-3.8220	-2.1193	0.6096
-2.8792	-1.6250	-0.7665
-0.3355	-1.4783	-0.5273
-1.7214	0.7217	-0.7289

H	-2.0015	1.0399	0.1705	-1.8848	1.8562	-0.0799	-1.8847	1.8466	-0.0701	-1.8785	1.0706	0.0259	-1.9252	1.7007	0.7023
H	0.9029	-3.8103	-0.0030	1.8026	-1.7329	2.2750	1.7863	-1.7768	2.2539	1.6846	-2.6257	2.4022	1.0280	-3.1288	0.7162
H	0.9372	-2.8123	1.4382	2.0305	-0.0020	2.4381	2.0325	-0.0483	2.4190	1.9527	-0.9021	2.5773	1.0634	-2.0614	2.1103
H	2.9777	-2.4969	0.1442	3.5146	-0.8918	0.7220	3.4940	-0.9463	0.6893	3.4362	-1.8181	0.8743	3.0864	-1.7968	0.7839
H	2.0786	-2.1605	-1.3272	2.1893	-1.6852	-0.1152	2.1552	-1.7250	-0.1402	2.1035	-2.5802	0.0200	2.1689	-1.5032	-0.6855
H	0.6249	-0.1078	-1.4664	0.3632	-0.3136	-1.1461	0.3358	-0.3410	-1.1512	0.3186	-1.1543	-1.0434	0.7115	0.5229	-0.8633
H	1.3370	2.0627	-2.3566	1.9883	1.3200	-3.1836	1.9753	1.3164	-3.1893	0.4059	-0.4544	-3.3229	2.7935	2.7500	-0.5073
H	2.6737	1.8935	-1.2471	0.6711	2.2562	-3.8297	0.6451	2.2331	-3.8343	1.9708	0.2711	-3.0457	1.7073	4.1068	-0.5949
H	0.9688	2.0135	0.6971	0.8264	2.6880	-1.4505	0.8023	2.6604	-1.4522	0.8713	1.8296	-1.3224	1.0223	2.6999	1.2539
H	-0.2861	2.0556	-0.5221	-0.6821	1.8984	-1.8272	-0.7019	1.8643	-1.8353	-0.6615	1.0791	-1.7114	-0.2105	2.7229	0.0216
H	-1.3475	-0.5948	2.3806	1.3561	2.0079	1.7668	1.3670	1.9704	1.7563	1.3433	1.1230	1.9077	-1.2680	0.0854	2.9765
H	0.3920	-0.8489	2.3206	0.2930	2.9317	0.7114	0.3007	2.9046	0.7131	0.3290	2.0794	0.8339	0.4811	-0.0785	2.9295
H	-0.3010	0.7468	2.0550	-0.3091	2.3396	2.2241	-0.2912	2.3114	2.2298	-0.3185	1.5097	2.3355	-0.2898	1.4735	2.6221
H	-2.0179	-2.4747	2.3820	-0.5723	1.1372	3.7981	-0.5953	1.1181	3.8072	-0.5669	0.2881	3.8899	-2.8045	-3.2540	2.5033
H	-1.0058	-3.8951	2.0972	0.6164	-0.0741	4.2562	0.6393	-0.0630	4.2234	0.5039	-1.0124	4.4211	-2.0628	-1.7507	3.0038
H	-0.9315	-4.9923	-0.1522	0.2911	-2.4968	3.6625	0.2803	-2.5195	3.6630	0.1325	-3.3701	3.7496	-0.7766	-4.3205	0.5525
H	-2.6510	-4.8486	-0.5061	-1.4509	-2.5503	3.9198	-1.4612	-2.5622	3.9276	-1.6098	-3.3623	4.0064	-2.5075	-4.2343	0.2451
H	-1.4713	-4.1371	-1.6059	-0.7886	-2.9693	2.3406	-0.8083	-2.9860	2.3462	-0.9624	-3.7935	2.4240	-1.3747	-3.5318	-0.9129
H	3.7204	3.8106	-0.5173	2.1008	-0.8917	-3.8274	2.1230	-0.8906	-3.8306	-0.8168	0.3834	-5.0807	3.2627	1.6641	-2.4869
H	2.9926	1.0632	1.0543	3.0431	2.1804	-1.2365	3.0296	2.1440	-1.2442	3.0608	1.2558	-1.1033	3.0726	1.7754	1.6377
H	3.8736	-0.5505	1.0816	4.2107	1.0498	-0.3778	4.1956	1.0016	-0.3983	4.1929	0.0993	-0.2310	3.9629	0.1678	1.6921
H	0.6594	5.5843	-1.9280	-1.4557	1.2699	-4.4267	-1.4649	1.2155	-4.4377	2.4441	2.5640	-3.5654	0.1763	3.4643	-3.8066
H	0.0025	4.0595	-2.4679	-1.5301	-0.4163	-4.8742	-1.5150	-0.4713	-4.8855	1.5975	3.3126	-4.8962	-0.6682	2.6467	-2.4732
H	-0.2497	4.5679	-0.7873	-1.7865	0.0530	-3.1789	-1.7786	-0.0061	-3.1904	0.9807	3.5093	-3.2357	-0.0183	4.2858	-2.2780
H	-4.3407	0.7042	-0.0408	-4.0649	0.0670	0.6023	-4.0517	1.6067	0.7869	-4.4842	-0.0616	2.0661	-4.0331	0.4179	-0.8146
H	-3.0386	-4.4002	2.9144	-2.1003	0.1096	4.9912	-2.0806	-0.0467	5.0045	-1.0711	-0.5380	5.9306	-1.0640	-3.5562	3.8940
H	4.3694	7.0738	-0.1433	1.2117	-3.7874	-5.2664	1.2963	-3.8105	-5.2467	-1.0157	4.0056	-6.5802	3.1089	0.8741	-5.7421

(2*R**,4*S**,5*R**,9*S**,10*R**)-**3K**			
C	-2.8813	-0.8871	-0.7626
C	-3.1232	-0.8220	0.7399
C	-2.5623	0.4575	1.4106
C	-1.0562	0.6359	0.9886
C	-0.7199	0.5142	-0.5585
C	-1.3942	-0.7719	-1.1019
C	-0.3648	1.8623	1.6284
C	1.1503	1.8332	1.4142
C	1.4865	1.6618	-0.0468
C	0.8661	0.4079	-0.6748
C	3.1684	-1.6513	-1.3200
C	2.8683	-0.4060	-2.1352
C	1.3926	0.0396	-2.0911
C	-1.2041	1.7145	-1.4106
C	-3.4434	1.6903	1.0929
C	-2.6403	0.2069	2.9437
C	3.9775	-1.5671	-0.2447
C	2.2226	2.5900	-0.6815
C	2.5276	-2.9185	-1.8208
O	-3.3670	-2.1330	-1.2637
O	-4.7909	1.5053	1.5178
C	4.3151	-2.6685	0.6738
O	3.8047	-3.7669	0.7617
O	5.3192	-2.2890	1.4827
H	-3.4560	-0.1087	-1.2719
H	-4.1970	-0.9350	0.9382
H	-2.6637	-1.7083	1.2014
H	-0.5548	-0.2359	1.4429
H	-0.8715	-1.6565	-0.7120

(2*R**,4*S**,5*R**,9*S**,10*R**)-**3L**		
-2.5447	-0.0646	1.7544
-2.3128	-1.4034	2.4411
-0.8473	-1.6241	2.8896
0.0991	-1.3673	1.6569
-0.1313	-0.0268	0.8370
-1.6462	0.1025	0.5283
1.5982	-1.6030	1.9534
2.4319	-1.6164	0.6706
2.1823	-0.3757	-0.1514
0.7071	-0.1619	-0.5115
0.6263	1.6551	-3.9906
0.9378	0.5777	-2.9671
0.4347	0.9514	-1.5604
0.3230	1.2550	1.5803
-0.4949	-0.7690	4.1319
-0.7498	-3.1166	3.3164
-0.2862	1.4220	-4.9553
3.2028	0.4433	-0.4590
1.4205	2.9264	-3.8441
-3.9095	0.0182	1.3487
-1.3012	-1.1094	5.2572
-0.7232	2.3799	-5.9874
-0.4715	3.5638	-6.0826
-1.5087	1.7513	-6.8790
-2.3892	0.7541	2.4621
-3.0093	-1.5066	3.2825
-2.6075	-2.2028	1.7452
-0.1748	-2.1678	0.9484
-1.9343	-0.6406	-0.2281

(2*R**,4*S**,5*R**,9*S**,10*R**)-**3M**		
-2.4845	0.7873	1.6548
-2.2833	-0.5750	2.3054
-0.8146	-0.8681	2.7012
0.1033	-0.6197	1.4462
-0.0957	0.7513	0.6705
-1.6114	0.9476	0.4103
1.6000	-0.9277	1.6867
2.3951	-0.9331	0.3789
2.1722	0.3446	-0.3925
0.6965	0.6180	-0.7060
0.2799	0.2611	-3.8107
0.9166	1.4766	-3.1608
0.4292	1.7611	-1.7257
0.4304	1.9926	1.4343
-0.3863	-0.0601	3.9510
-0.7670	-2.3738	3.0883
1.0425	-0.8081	-4.1165
3.2120	1.1411	-0.6930
-1.1966	0.3765	-4.0821
-3.8464	0.9173	1.2499
-1.1604	-0.4022	5.0984
0.5696	-2.0762	-4.6995
-0.5705	-2.4596	-4.8650
1.6251	-2.8339	-5.0445
-2.2904	1.5900	2.3719
-2.9535	-0.6699	3.1690
-2.6357	-1.3443	1.6023
-0.2270	-1.3887	0.7269
-1.9517	0.2353	-0.3543

(2*R**,4*S**,5*R**,9*S**,10*R**)-**3N**		
-2.7758	0.9694	0.8485
-2.5831	-0.0207	1.9887
-1.1919	0.0681	2.6638
-0.0813	-0.0136	1.5523
-0.2543	0.9333	0.2888
-1.7081	0.7904	-0.2314
1.3637	0.0252	2.1013
2.3884	-0.3333	1.0220
2.1986	0.5180	-0.2096
0.7879	0.4264	-0.8048
0.9683	-1.1074	-3.5526
1.3776	0.3399	-3.3449
0.6134	1.0587	-2.2148
0.0118	2.4331	0.5754
-1.0805	1.3268	3.5571
-1.0860	-1.1752	3.5920
1.8589	-2.0931	-3.3189
3.2009	1.2962	-0.6520
-0.4364	-1.3124	-4.0532
-4.0495	0.7410	0.2457
-2.1134	1.3713	4.5386
1.6667	-3.5486	-3.4102
2.5705	-4.3489	-3.5757
0.4044	-3.9502	-3.2121
-2.7823	1.9970	1.2230
-3.3879	0.1097	2.7238
-2.7369	-1.0350	1.5917
-0.1980	-1.0323	1.1443
-1.8396	-0.1951	-0.6997

H	-1.2971	-0.8350	-2.1928
H	-0.5427	1.8882	2.7081
H	-0.7760	2.7942	1.2253
H	1.5857	2.7521	1.8248
H	1.5899	1.0021	1.9800
H	1.1585	-0.4310	-0.0273
H	3.5194	0.4186	-1.8260
H	3.1394	-0.6060	-3.1802
H	1.2661	0.8822	-2.7804
H	0.7994	-0.7769	-2.5134
H	-0.9112	2.6798	-0.9900
H	-0.7937	1.6675	-2.4248
H	-2.2848	1.7328	-1.5467
H	-3.4979	1.8945	0.0270
H	-3.0648	2.5950	1.5795
H	-2.4345	1.1171	3.5160
H	-3.6343	-0.1452	3.2432
H	-1.9227	-0.5588	3.2599
H	4.4194	-0.6126	0.0281
H	2.4555	2.5363	-1.7387
H	2.6070	3.4607	-0.1570
H	1.4944	-2.9879	-1.4669
H	2.5158	-2.9316	-2.9164
H	3.0645	-3.8196	-1.5164
H	-2.9151	-2.8487	-0.7851
H	-4.8181	1.6151	2.4832
H	5.4695	-3.0762	2.0461

-1.8742	1.0773	0.0797
1.7483	-2.5660	2.4516
1.9848	-0.8384	2.6359
3.4910	-1.7201	0.9357
2.1749	-2.4980	0.0699
0.3735	-1.0935	-0.9944
0.4742	-0.3720	-3.2648
2.0172	0.3935	-2.9724
0.8729	1.9028	-1.2392
-0.6421	1.1270	-1.6482
1.3413	1.1820	1.9705
0.2949	2.1252	0.9157
-0.3266	1.5173	2.4143
-0.6200	0.2992	3.9699
0.5438	-0.9339	4.4337
0.2071	-3.3411	3.7983
-1.5387	-3.3811	4.0302
-0.8580	-3.7857	2.4552
-0.7923	0.4621	-5.0031
3.0734	1.3652	-1.0145
4.2193	0.2205	-0.1455
2.4376	2.7034	-3.5031
1.5335	3.4711	-4.7840
0.9509	3.5861	-3.1081
-4.0825	-0.6974	0.7130
-2.1788	-0.7107	5.1284
-1.7468	2.4601	-7.5122

-1.8109	1.9412	-0.0102
1.7235	-1.9120	2.1493
2.0395	-0.2026	2.3802
3.4564	-1.0877	0.6078
2.0847	-1.7828	-0.2421
0.3142	-0.2947	-1.1852
2.0082	1.3891	-3.1761
0.6942	2.3541	-3.7824
0.8884	2.6962	-1.3852
-0.6439	1.9657	-1.7881
1.4509	1.8634	1.8038
0.4306	2.8790	0.7912
-0.1920	2.2638	2.2862
-0.4802	1.0157	3.8210
0.6564	-0.2685	4.2088
0.1942	-2.6499	3.5335
-1.5439	-2.6236	3.8204
-0.9301	-3.0154	2.2149
2.1077	-0.7915	-3.9025
3.0992	2.0892	-1.2059
4.2270	0.8730	-0.4122
-1.4467	1.3958	-4.3965
-1.5379	-0.2774	-4.8873
-1.7684	0.1478	-3.1775
-4.4080	0.7780	2.0312
-2.0237	0.0363	5.0217
1.2133	-3.6423	-5.4141

-1.9130	1.5186	-1.0261
1.4863	-0.6906	2.9203
1.5947	1.0120	2.5168
3.3970	-0.2235	1.4384
2.2811	-1.3893	0.7439
0.5848	-0.6456	-0.9396
2.4569	0.4108	-3.1725
1.2036	0.8844	-4.2824
0.9205	2.1108	-2.2063
-0.4439	1.0627	-2.4966
0.9480	2.6040	1.1126
0.0655	3.0078	-0.3553
-0.7844	2.9058	1.1496
-1.1776	2.2474	2.9885
-0.1202	1.3684	4.0815
-0.2110	-1.1207	4.2476
-1.9670	-1.2709	4.2372
-1.0138	-2.1017	3.0111
2.8734	-1.8414	-3.0197
3.1010	1.9574	-1.5050
4.1664	1.3102	-0.1535
-0.7226	-0.5015	-4.7322
-0.5493	-2.2358	-4.6273
-1.1421	-1.3224	-3.2170
-4.7227	0.8821	0.9343
-1.8758	0.7476	5.2460
0.4581	-4.9256	-3.2714

Supplementary S2. The Z-matrices of (5*S**,6*S**,9*S**,10*R**)-**4** optimized at B3LYP/6-31G(d)-GD3BJ level by Gaussian.

(5*S**,6*S**,9*S**,10*R**)-**4A**			
O	3.4050	-1.4303	-1.3344
C	2.3377	-1.6695	-1.9170
C	1.0138	-1.4380	-1.1761
C	0.6714	0.1006	-1.0195
C	1.8483	0.8452	-0.3031
C	-0.1396	-2.2874	-1.7391
C	-1.4138	-2.0898	-0.9164
C	-1.7750	-0.6297	-0.8437
C	-0.6851	0.2420	-0.2027
C	-1.3805	1.7527	2.5886
C	-2.0877	1.8705	1.2523
C	-1.1253	1.7137	0.0585
C	0.5141	0.7642	-2.4114
C	2.3410	-2.1241	-3.3539
C	-1.4884	0.6245	3.3179
C	-2.9574	-0.2132	-1.3295
C	-0.6150	2.9758	3.0275
O	0.2344	-3.6650	-1.6779
C	2.1876	0.3288	1.0787
O	1.5895	0.6640	2.0942
O	3.1936	-0.5656	1.1061
C	-0.8053	0.3777	4.5984
O	-1.4047	0.0502	5.6085
O	0.5334	0.4809	4.5113
H	1.2047	-1.8462	-0.1715
H	2.7573	0.8390	-0.9157
H	1.6467	1.9166	-0.1991
H	-0.3336	-2.0479	-2.7903

(5*S**,6*S**,9*S**,10*R**)-**4B**		
3.3965	-1.4316	-1.3599
2.3268	-1.6641	-1.9407
1.0092	-1.4359	-1.1877
0.6574	0.1016	-1.0434
1.8326	0.8562	-0.3341
-0.1391	-2.3022	-1.7317
-1.4247	-2.0977	-0.9285
-1.7853	-0.6367	-0.8648
-0.6989	0.2406	-0.2265
-1.4017	1.7409	2.5616
-2.1079	1.8669	1.2253
-1.1444	1.7113	0.0321
0.4942	0.7552	-2.4392
2.3202	-2.1137	-3.3791
-1.5134	0.6093	3.2849
-2.9661	-0.2244	-1.3578
-0.6331	2.9599	3.0066
0.2315	-3.6816	-1.6671
2.1790	0.3414	1.0465
1.5736	0.6608	2.0626
3.1942	-0.5431	1.0703
-0.8287	0.3482	4.5619
-1.4257	-0.0009	5.5661
0.5096	0.4578	4.4761
1.2113	-1.8326	-0.1810
2.7392	0.8515	-0.9505
1.6260	1.9266	-0.2312
-0.3328	-2.0769	-2.7859

(5*S**,6*S**,9*S**,10*R**)-**4C**		
1.8292	0.3811	3.5188
1.8292	1.2674	2.6526
1.3972	0.9141	1.2235
-0.1667	0.6842	1.1060
-0.6446	-0.4032	2.1239
1.9775	1.8825	0.1732
1.5669	1.4798	-1.2457
0.0701	1.3469	-1.3338
-0.4949	0.2747	-0.3959
-1.4907	-2.0840	-2.2290
-2.2557	-0.7944	-2.0146
-1.9809	-0.1005	-0.6617
-0.9313	1.9817	1.4809
2.1893	2.6850	3.0159
-1.7373	-3.1547	-1.4507
-0.6321	2.1558	-2.1453
-0.4689	-2.0530	-3.3346
3.4032	1.8527	0.2646
-0.0113	-1.7687	1.9736
-0.4350	-2.6337	1.2161
1.0784	-1.9674	2.7379
-1.0070	-4.4292	-1.5505
-0.9970	-5.1408	-2.5389
-0.3652	-4.7079	-0.4000
1.8944	-0.0494	1.0316
-0.5086	-0.0696	3.1595
-1.7297	-0.5483	2.0669
1.6607	2.9129	0.3687

(5*S**,6*S**,9*S**,10*R**)-**4D**		
-0.4553	-3.7733	-1.0983
-1.3909	-2.9634	-1.1666
-1.2906	-1.6416	-0.3952
-0.2744	-0.6268	-1.0637
1.1305	-1.2943	-1.2338
-2.6686	-1.0344	-0.0717
-2.5255	0.2389	0.7657
-1.6101	1.2197	0.0818
-0.1959	0.6732	-0.1522
2.1085	2.1991	1.5361
1.2337	2.7731	0.4394
0.8298	1.7388	-0.6318
-0.7480	-0.2441	-2.4893
-2.5829	-3.2491	-2.0433
3.4159	1.9687	1.3009
-2.0744	2.4283	-0.2793
1.4397	1.9777	2.8682
-3.4255	-1.9893	0.6744
1.7857	-1.7477	0.0523
2.4408	-1.0069	0.7758
1.5704	-3.0405	0.3606
4.3349	1.3831	2.2886
5.2530	2.0054	2.7933
4.0694	0.0850	2.5232
-0.8750	-1.9424	0.5791
1.0921	-2.1413	-1.9285
1.8375	-0.6198	-1.7298
-3.2302	-0.8161	-0.9866

(5*S**,6*S**,9*S**,10*R**)-**4E**		
1.8242	0.4097	3.5210
1.8197	1.2926	2.6516
1.3933	0.9248	1.2242
-0.1722	0.7099	1.1013
-0.6617	-0.3700	2.1220
1.9926	1.8744	0.1692
1.5722	1.4931	-1.2529
0.0743	1.3681	-1.3375
-0.4972	0.2992	-0.4006
-1.4829	-2.0481	-2.2455
-2.2554	-0.7635	-2.0263
-1.9833	-0.0726	-0.6712
-0.9276	2.0147	1.4688
2.1749	2.7138	3.0050
-1.7165	-3.1200	-1.4647
-0.6238	2.1829	-2.1465
-0.4685	-2.0092	-3.3575
3.4194	1.8407	0.2560
-0.0288	-1.7366	1.9819
-0.4385	-2.6003	1.2156
1.0522	-1.9343	2.7592
-0.9753	-4.3884	-1.5638
-0.9440	-5.0929	-2.5569
-0.3450	-4.6687	-0.4071
1.8812	-0.0457	1.0457
-0.5294	-0.0309	3.1565
-1.7468	-0.5125	2.0590
1.6908	2.9093	0.3631

H	-1.2770	-2.4870	0.0982
H	-2.2216	-2.6913	-1.3526
H	-0.4975	-0.2117	0.7786
H	-2.9066	1.1454	1.1855
H	-2.5656	2.8569	1.1964
H	-1.5990	2.1328	-0.8371
H	-0.2638	2.3583	0.2420
H	1.4450	0.7124	-2.9859
H	-0.2697	0.2936	-3.0120
H	0.2649	1.8269	-2.3199
H	2.0751	-3.1806	-3.4183
H	1.6539	-1.5195	-3.9491
H	3.3456	-1.9956	-3.7683
H	-2.1227	-0.1974	3.0034
H	-3.6681	-0.9111	-1.7657
H	-3.2775	0.8225	-1.3149
H	-1.2501	3.8647	2.9488
H	-0.2815	2.9219	4.0675
H	0.2669	3.1273	2.3983
H	-0.5344	-4.2020	-1.9430
H	3.5440	-0.8279	0.2165
H	0.8727	0.6219	3.5911

-1.3040	-2.4859	0.0916
-2.2248	-2.7035	-1.3725
-0.5091	-0.2104	0.7556
-2.9290	1.1446	1.1545
-2.5825	2.8550	1.1729
-1.6188	2.1280	-0.8641
-0.2851	2.3587	0.2159
1.4267	0.7126	-3.0118
-0.2823	0.2709	-3.0384
0.2315	1.8152	-2.3542
2.0547	-3.1703	-3.4442
1.6279	-1.5088	-3.9677
3.3216	-1.9827	-3.8003
-2.1530	-0.2071	2.9662
-3.6727	-0.9270	-1.7941
-3.2879	0.8108	-1.3527
-1.2660	3.8508	2.9321
-0.3004	2.8999	4.0464
0.2493	3.1123	2.3784
0.2008	-3.9762	-0.7384
3.5545	-0.7914	0.1806
0.8490	0.6177	3.5593

2.0422	0.5309	-1.5267
1.9562	2.2180	-1.9584
0.0884	-0.6346	-0.5958
-2.0626	-0.1081	-2.8455
-3.3301	-1.0151	-2.0755
-2.6180	0.7892	-0.5931
-2.3433	-0.7770	0.1169
-0.7550	2.2596	2.5257
-0.6391	2.8364	0.8643
-2.0132	1.8512	1.3722
2.2498	2.7719	4.1051
3.1615	2.9494	2.5964
1.4202	3.3768	2.6670
-2.4752	-3.1223	-0.6569
-0.1409	2.8968	-2.7710
-1.7137	2.1261	-2.2191
0.1247	-2.9679	-3.4041
-0.9656	-1.9034	-4.2988
0.2337	-1.2283	-3.1776
3.7669	2.3849	-0.4662
1.3655	-1.1821	3.2711
-0.3917	-3.9664	0.2570

-2.1304	-0.0028	1.7613
-3.5206	0.6645	0.9480
0.1510	0.3286	0.8288
0.3551	3.2599	0.8752
1.7892	3.5785	-0.0606
0.4385	2.2733	-1.5057
1.7502	1.2634	-0.9795
-0.7737	-1.1184	-3.1484
-1.7477	0.1993	-2.4946
-0.0664	0.4761	-2.9545
-3.4540	-3.4909	-1.4319
-2.7952	-2.3967	-2.6915
-2.3605	-4.1093	-2.6821
3.8834	2.2140	0.3544
-3.0992	2.7270	-0.0723
-1.4694	3.1693	-0.7900
2.1364	1.6666	3.6517
0.9717	2.9072	3.2098
0.6641	1.2112	2.7943
-4.2578	-1.5667	0.9542
0.9669	-3.5235	-0.2603
3.3739	-0.2906	1.9250

2.0358	0.5431	-1.5495
1.9668	2.2371	-1.9564
0.0840	-0.6121	-0.5972
-2.0662	-0.0728	-2.8545
-3.3286	-0.9899	-2.0878
-2.6194	0.8178	-0.6024
-2.3485	-0.7504	0.1051
-0.7603	2.2903	2.5157
-0.6198	2.8667	0.8562
-2.0095	1.8951	1.3485
2.2318	2.8092	4.0937
3.1478	2.9768	2.5859
1.4058	3.4016	2.6482
-2.4513	-3.0917	-0.6676
-0.1272	2.9229	-2.7696
-1.7056	2.1619	-2.2189
0.1337	-2.9181	-3.4294
-0.9726	-1.8658	-4.3188
0.2272	-1.1776	-3.2062
3.7406	1.0023	-0.1236
1.3275	-1.1519	3.3030
-0.3898	-3.9339	0.2559

Supplementary S3. The Z-matrices of (2*S**,5*S**,9*S**,10*R**)-**5** optimized at B3LYP/6-31G(d)-GD3BJ level by Gaussian.

(2*S**,5*S**,9*S**,10*R**)-**5A**			
C	-2.4516	0.2773	0.6814
C	-2.7153	1.0713	-0.5925
C	-2.2029	0.3701	-1.8806
C	-0.6957	-0.0208	-1.6841
C	-0.3203	-0.7735	-0.3642
C	-0.9784	-0.0502	0.8262
C	-0.0124	-0.7265	-2.8580
C	1.4037	-1.1364	-2.6647
C	2.0057	-1.0827	-1.4620
C	1.2597	-0.6654	-0.1892
C	2.1503	0.7946	2.5094
C	2.7287	-0.5027	1.9602
C	1.7666	-1.3610	1.1142
C	-0.7638	-2.2586	-0.3711
C	-3.1404	-0.8019	-2.2299
C	-2.3272	1.4085	-3.0263
C	1.0786	0.8010	3.3306
C	3.4853	-1.3534	-1.3774
C	2.8871	2.0388	2.0798
O	-0.5294	-0.9209	-3.9531
O	-2.7768	1.0631	1.8343
C	0.5073	2.0186	3.9389
O	1.1590	2.8295	4.5740
O	-0.8202	2.1204	3.7381
H	-3.0616	-0.6300	0.7198
H	-3.7901	1.2841	-0.6697
H	-2.2400	2.0578	-0.4925
H	-0.1755	0.9515	-1.6345

(2*S**,5*S**,9*S**,10*R**)-**5B**		
-2.3235	0.2063	1.2149
-2.6128	1.5410	0.5382
-2.4039	1.5184	-0.9967
-0.9762	0.9463	-1.3061
-0.5561	-0.3812	-0.5851
-0.9059	-0.2678	0.9204
-0.6031	0.8438	-2.7862
0.7389	0.2967	-3.1020
1.5070	-0.2900	-2.1666
1.0265	-0.5219	-0.7256
2.5993	-1.0314	2.0913
2.8487	-1.6666	0.7320
1.5670	-1.8468	-0.1015
-1.2572	-1.6279	-1.1836
-3.5760	0.7634	-1.6551
-2.4890	2.9922	-1.4753
2.9757	0.2426	2.3222
2.9215	-0.6615	-2.5329
2.0159	-1.9517	3.1340
-1.3013	1.2369	-3.7147
-2.4221	0.3656	2.6300
2.8197	0.9939	3.5815
3.7231	1.6546	4.0639
1.5719	0.9470	4.0827
-3.0533	-0.5545	0.9228
-3.6357	1.8576	0.7836
-1.9665	2.3070	0.9908
-0.2908	1.7217	-0.9219

(2*S**,5*S**,9*S**,10*R**)-**5C**		
-2.2949	-0.7981	-0.1321
-2.1677	-1.1155	-1.6141
-0.9012	-1.9419	-1.9514
0.3534	-1.1995	-1.3692
0.3102	-0.7378	0.1310
-1.0588	-0.0903	0.3919
1.7017	-1.8615	-1.6359
2.8963	-1.1299	-1.1593
2.7964	-0.0968	-0.3043
1.4618	0.3595	0.3365
-0.2052	2.6384	2.0099
0.6071	1.5211	2.6451
1.7181	0.8650	1.7980
0.5120	-1.9029	1.1275
-1.0951	-3.3910	-1.4636
-0.8075	-1.9912	-3.4994
-1.5536	2.5845	2.0544
4.0472	0.6943	-0.0064
0.5811	3.7611	1.3858
1.8645	-2.9064	-2.2558
-3.3875	0.1115	0.0819
-2.4806	3.5218	1.3948
-2.4413	4.7334	1.5031
-3.3932	2.8548	0.6566
-2.5013	-1.6994	0.4531
-3.0733	-1.6365	-1.9525
-2.1593	-0.1705	-2.1762
0.4014	-0.2691	-1.9620

(2*S**,5*S**,9*S**,10*R**)-**5D**		
-2.5136	1.5740	0.9578
-2.0365	3.0185	0.8689
-1.2031	3.3187	-0.4011
-0.0525	2.2575	-0.5197
-0.4350	0.7457	-0.3427
-1.3379	0.6021	0.9128
0.8561	2.4017	-1.7410
1.9380	1.4010	-1.8947
2.0021	0.2944	-1.1333
0.9190	-0.0702	-0.1042
1.2583	-3.7755	1.1807
1.5565	-2.2917	1.0577
0.6691	-1.6059	0.0052
-1.1818	0.1724	-1.5751
-2.1502	3.4071	-1.6152
-0.5908	4.7309	-0.2018
0.6996	-4.2576	2.3096
3.2217	-0.5813	-1.2607
1.7117	-4.6218	0.0187
0.7948	3.3142	-2.5583
-3.1876	1.3802	2.1998
0.3645	-5.6682	2.5797
0.6762	-6.2358	3.6113
-0.3864	-6.2219	1.6086
-3.2321	1.3442	0.1657
-2.9026	3.6915	0.9297
-1.4432	3.2440	1.7669
0.6155	2.4925	0.3271

(2*S**,5*S**,9*S**,10*R**)-**5E**		
-2.6446	-0.0297	1.1256
-2.9181	1.3475	0.5342
-2.5837	1.4487	-0.9742
-1.1191	0.9348	-1.2083
-0.7093	-0.4290	-0.5475
-1.1856	-0.4315	0.9307
-0.6395	0.9479	-2.6596
0.7305	0.4467	-2.9184
1.4504	-0.1886	-1.9766
0.8888	-0.5201	-0.5831
2.7665	-0.9092	1.9706
2.7521	-1.8164	0.7525
1.4133	-1.8747	-0.0095
-1.3194	-1.6541	-1.2767
-3.6776	0.7213	-1.7823
-2.6734	2.9533	-1.3446
3.4549	0.2500	1.9300
2.8829	-0.5256	-2.2948
1.9742	-1.3986	3.1557
-1.2771	1.3954	-3.6072
-2.9022	0.0005	2.5284
3.5850	1.2329	3.0211
3.3883	2.4241	2.8599
4.0317	0.6870	4.1674
-3.3111	-0.7836	0.6969
-3.9668	1.6196	0.7157
-2.3344	2.0899	1.0975
-0.4881	1.6984	-0.7215

H	-0.4510	0.8975	0.9965	-0.2077	0.4272	1.4071	-1.0582	0.9173	-0.0414	-0.7337	0.7466	1.8198	-0.5564	0.2481	1.5226
H	-0.8653	-0.6273	1.7500	-0.7583	-1.2240	1.4350	-1.2154	0.0128	1.4651	-1.7396	-0.4140	1.0022	-1.0495	-1.4168	1.3910
H	1.9223	-1.4315	-3.5710	1.0688	0.4553	-4.1236	3.8380	-1.4559	-1.5882	2.6941	1.6565	-2.6301	1.1191	0.6736	-3.9058
H	1.4862	0.4060	-0.0932	1.4615	0.3126	-0.1579	1.1677	1.2421	-0.2489	1.3200	0.2634	0.8648	1.2549	0.2845	0.0680
H	3.6288	-0.2882	1.3730	3.5904	-1.0897	0.1701	1.0889	1.9462	3.5373	1.4003	-1.7949	2.0242	3.5635	-1.5445	0.0714
H	3.0759	-1.1041	2.8110	3.3078	-2.6510	0.8899	-0.0443	0.7344	3.0416	2.6187	-2.1707	0.8292	3.0012	-2.8329	1.0862
H	2.2616	-2.3091	0.8738	1.7580	-2.5827	-0.8920	2.5118	1.6223	1.7598	0.7969	-2.0782	-0.9765	1.5102	-2.6055	-0.8231
H	0.9409	-1.6513	1.7686	0.8243	-2.3233	0.5438	2.1454	0.0430	2.3879	-0.3651	-1.8142	0.2948	0.6857	-2.3170	0.6755
H	-1.8465	-2.3697	-0.3193	-2.3353	-1.6153	-1.0321	0.4952	-1.5471	2.1630	-2.1443	0.6514	-1.7448	-2.4066	-1.6749	-1.2278
H	-0.4209	-2.7975	-1.2588	-1.0790	-1.7342	-2.2575	-0.2792	-2.6487	1.0646	-0.6028	0.2682	-2.4983	-1.0399	-1.6960	-2.3334
H	-0.3778	-2.8038	0.4956	-0.9139	-2.5544	-0.7133	1.4649	-2.4198	0.9824	-1.4087	-0.8914	-1.4513	-0.9929	-2.5958	-0.8236
H	-4.1324	-0.4282	-2.5130	-4.5099	1.3270	-1.5354	-0.1989	-4.0016	-1.5972	-2.8122	4.2773	-1.5212	-4.6393	1.2393	-1.6763
H	-2.7735	-1.3925	-3.0731	-3.4323	0.6186	-2.7284	-1.3894	-3.4557	-0.4164	-1.6138	3.5229	-2.5598	-3.4578	0.6867	-2.8519
H	-3.3079	-1.4807	-1.3943	-3.7636	-0.2127	-1.2086	-1.8978	-3.8810	-2.0290	-2.8100	2.5454	-1.7101	-3.8547	-0.2995	-1.4458
H	-2.0934	0.9766	-4.0035	-2.4631	3.0756	-2.5655	-0.0013	-2.6447	-3.8448	-0.0699	5.0866	-1.0953	-2.5592	3.1230	-2.4190
H	-3.3477	1.8053	-3.0877	-3.4223	3.4608	-1.1405	-1.7379	-2.3722	-3.9373	-1.3694	5.4678	0.0291	-3.6452	3.3717	-1.0561
H	-1.6562	2.2603	-2.8674	-1.6632	3.5905	-1.0741	-0.6342	-0.9944	-3.9206	0.1238	4.7425	0.6290	-1.9025	3.5377	-0.8297
H	0.5861	-0.1189	3.6240	3.4972	0.8158	1.5604	-2.0598	1.7680	2.5596	0.4955	-3.6093	3.1573	3.9367	0.5815	1.0143
H	3.6930	-2.2508	-0.7885	3.6299	-0.1349	-1.8865	4.8819	0.4078	-0.6561	3.7557	-0.6256	-0.3068	3.5540	-0.0299	-1.5871
H	3.9310	-1.5095	-2.3663	3.0884	-1.7385	-2.4478	3.8693	1.7625	-0.1686	2.9552	-1.5936	-1.5766	3.0517	-1.6056	-2.2658
H	4.0016	-0.5006	-0.9265	3.1657	-0.3833	-3.5645	4.3730	0.5345	1.0260	3.9308	-0.1960	-2.0025	3.1748	-0.1877	-3.2958
H	3.8876	2.0536	2.5239	0.9540	-2.1280	2.9418	1.1923	3.3969	0.5556	1.0341	-4.4976	-0.8314	0.9584	-1.6737	2.8594
H	2.9939	2.0561	0.9898	2.5333	-2.9170	3.1207	-0.0443	4.5637	0.9895	2.7187	-4.3271	-0.2957	2.4557	-2.2810	3.5890
H	2.3782	2.9649	2.3577	2.1182	-1.5614	4.1504	1.2474	4.2069	2.1320	1.7618	-5.6865	0.2634	1.8712	-0.6460	3.9417
H	-3.7063	1.3449	1.7367	-3.3251	0.6721	2.8281	-4.1841	-0.3233	-0.2814	-3.9390	1.9981	2.2267	-3.8226	0.2905	2.6528
H	-1.2389	1.4958	3.1048	0.9342	0.4610	3.5257	-3.2617	1.8789	0.5778	-0.6267	-5.5882	0.9075	4.2207	-0.2663	4.0874

(2*S**,5*S**,9*S**,10*R**)-**5F**			
C	-3.1903	0.2455	-0.0763
C	-3.4145	0.8187	-1.4703
C	-2.5205	0.1741	-2.5578
C	-1.0252	0.2171	-2.0818
C	-0.7127	-0.2662	-0.6217
C	-1.7322	0.3886	0.3503
C	-0.0086	-0.4022	-3.0418
C	1.4084	-0.4092	-2.6081
C	1.7756	-0.0821	-1.3564
C	0.7569	0.2408	-0.2505
C	2.4923	0.3221	3.2816
C	1.9885	0.8294	1.9425
C	1.2096	-0.2425	1.1614
C	-0.7875	-1.8085	-0.4764
C	-3.0673	-1.2284	-2.8935
C	-2.6871	1.0418	-3.8340
C	3.7800	-0.0583	3.4070
C	3.2489	0.0251	-1.0574
C	1.4622	0.2669	4.3803
O	-0.2702	-0.8274	-4.1623
O	-3.9884	0.9698	0.8585
C	4.4307	-0.5558	4.6329
O	5.1067	-1.5687	4.6559
O	4.2736	0.2731	5.6821
H	-3.5054	-0.8006	-0.0221
H	-4.4759	0.7216	-1.7363
H	-3.2335	1.9027	-1.4328
H	-0.7711	1.2912	-2.0966
H	-1.5053	1.4590	0.4563

(2*S**,5*S**,9*S**,10*R**)-**5G**		
-2.8065	0.8214	1.1983
-3.4550	1.5264	0.0131
-3.2200	0.8111	-1.3397
-1.6831	0.5452	-1.5165
-0.9119	-0.0942	-0.3086
-1.3031	0.6623	0.9904
-1.2813	-0.1761	-2.8035
0.1599	-0.4665	-2.9890
1.0552	-0.3052	-1.9986
0.6516	0.1060	-0.5727
3.6047	-0.4090	1.9772
2.7589	0.2680	0.9144
1.5269	-0.5675	0.5281
-1.2253	-1.6031	-0.1349
-4.1206	-0.4389	-1.4116
-3.7107	1.7860	-2.4435
3.4723	-0.0466	3.2694
2.5150	-0.4795	-2.3294
4.5310	-1.4842	1.4708
-2.0491	-0.4676	-3.7146
-3.0005	1.6115	2.3700
4.2294	-0.5980	4.4081
3.6952	-1.0009	5.4255
5.5624	-0.5237	4.2312
-3.2727	-0.1501	1.3862
-4.5302	1.6447	0.2051
-3.0649	2.5534	-0.0353
-1.2503	1.5530	-1.6410
-0.8461	1.6621	0.9864

(2*S**,5*S**,9*S**,10*R**)-**5H**		
-2.5147	0.0913	1.1944
-2.8178	1.4375	0.5470
-2.5628	1.4611	-0.9805
-1.1154	0.9240	-1.2647
-0.6818	-0.4109	-0.5618
-1.0721	-0.3366	0.9400
-0.7079	0.8651	-2.7378
0.6478	0.3469	-3.0386
1.4092	-0.2481	-2.1032
0.9084	-0.5210	-0.6754
2.6641	-0.9520	2.0017
2.7776	-1.7306	0.7020
1.4547	-1.8567	-0.0785
-1.3425	-1.6596	-1.2015
-3.7018	0.7046	-1.6939
-2.6625	2.9462	-1.4205
3.1296	0.3117	2.0698
2.8317	-0.5922	-2.4608
2.0027	-1.6858	3.1410
-1.3884	1.2733	-3.6730
-2.7221	0.1867	2.6028
3.0695	1.1872	3.2527
2.5691	2.2973	3.2247
3.6877	0.6569	4.3238
-3.2077	-0.6742	0.8347
-3.8549	1.7216	0.7718
-2.2038	2.2071	1.0369
-0.4555	1.7039	-0.8466
-0.4055	0.3655	1.4602

(2*S**,5*S**,9*S**,10*R**)-**5I**		
-2.4546	-0.9033	0.9835
-3.0910	-0.6478	-0.3765
-2.3069	-1.2839	-1.5532
-0.8011	-0.8494	-1.4616
-0.0888	-0.9790	-0.0689
-1.0194	-0.3895	1.0244
0.1225	-1.4088	-2.5454
1.5555	-1.0333	-2.4674
2.0739	-0.4074	-1.3957
1.2494	-0.1065	-0.1354
1.7735	2.2901	1.8363
2.7395	1.1340	1.6353
2.0805	-0.1879	1.1849
0.2573	-2.4457	0.2935
-2.5524	-2.8062	-1.5613
-2.9342	-0.7186	-2.8553
0.7690	2.1887	2.7300
3.4980	0.0768	-1.4721
1.9968	3.4683	0.9233
-0.2439	-2.0830	-3.5024
-3.2158	-0.2432	1.9930
-0.3000	3.1822	2.9215
-1.4786	2.9133	2.7656
0.1686	4.3743	3.3311
-2.4861	-1.9688	1.2269
-4.1291	-1.0065	-0.3638
-3.1711	0.4386	-0.5256
-0.8251	0.2324	-1.6799
-1.0466	0.7034	0.9232

H	-1.6381	-0.0198	1.3633	-0.8918	0.1695	1.8791	-0.9151	-1.2989	1.4403	-0.6269	-0.5782	2.0297
H	2.1226	-0.6505	-3.3888	0.4383	-0.7690	-3.9933	0.9924	0.5328	-4.0506	2.1341	-1.2567	-3.3578
H	0.7100	1.3398	-0.2159	0.8425	1.1886	-0.5220	1.3150	0.3054	-0.0783	0.9245	0.9345	-0.2633
H	1.3458	1.7029	2.1126	2.4195	1.2471	1.2784	3.5542	-1.2931	0.0675	3.5220	1.4222	0.9266
H	2.8318	1.1994	1.3520	3.3925	0.4809	0.0490	3.1397	-2.7395	0.9400	3.2657	0.9568	2.5829
H	1.8023	-1.1623	1.0795	1.8213	-1.5753	0.2118	1.5902	-2.5929	-0.8811	2.8593	-0.9556	1.0929
H	0.3455	-0.5116	1.7734	0.9548	-0.7111	1.4495	0.7284	-2.3158	0.5964	1.4609	-0.5271	2.0193
H	-1.7891	-2.1993	-0.6462	-2.2682	-1.7907	0.1141	-2.4255	-1.6647	-1.0932	0.8039	-2.5094	1.2399
H	-0.1141	-2.3278	-1.1644	-0.9945	-2.1850	-1.0319	-1.1212	-1.7502	-2.2689	-0.6276	-3.0647	0.4316
H	-0.5208	-2.1333	0.5344	-0.6512	-2.0438	0.6864	-1.0030	-2.5859	-0.7275	0.8790	-2.9304	-0.4647
H	-4.0507	-1.1506	-3.3742	-5.1775	-0.1469	-1.4552	-4.6508	1.2428	-1.5760	-1.9681	-3.3211	-2.3277
H	-2.4232	-1.7764	-3.5851	-3.9268	-1.0466	-2.2985	-3.5309	0.6032	-2.7681	-2.3389	-3.2820	-0.6050
H	-3.2192	-1.8528	-2.0137	-4.0307	-1.0846	-0.5387	-3.8771	-0.2914	-1.2888	-3.6073	-3.0224	-1.7723
H	-2.1827	0.6057	-4.7008	-3.6936	1.3297	-3.4372	-2.6066	3.0611	-2.5067	-2.5212	-1.1888	-3.7522
H	-3.7455	1.1488	-4.1008	-4.7432	2.1043	-2.2557	-3.6141	3.3877	-1.1006	-4.0175	-0.8880	-2.8751
H	-2.2860	2.0508	-3.6858	-3.0935	2.6908	-2.4799	-1.8605	3.5483	-0.9786	-2.7728	0.3620	-2.9396
H	4.4428	-0.0766	2.5463	2.7167	0.6722	3.5749	3.5279	0.8128	1.1921	0.6158	1.2839	3.3104
H	3.5492	-0.6681	-0.2672	2.9675	-1.2778	-1.7350	3.5252	-0.0562	-1.8058	4.1231	-0.4034	-0.7144
H	3.8654	-0.2094	-1.9328	2.6708	-0.7430	-3.3818	3.0150	-1.6671	-2.3818	3.9555	-0.1392	-2.4443
H	3.5005	1.0471	-0.7591	3.0558	0.4553	-2.1546	3.0801	-0.3039	-3.4887	3.5354	1.1622	-1.3386
H	1.1755	1.2810	4.6764	3.9896	-2.1923	0.8355	1.0398	-2.1016	2.8311	2.0605	3.1359	-0.1182
H	0.5639	-0.2595	4.0434	5.3353	-1.0372	0.8779	2.6407	-2.5105	3.4744	1.1944	4.2088	0.9692
H	1.8138	-0.2615	5.2703	4.9845	-2.0716	2.2735	1.7991	-1.0453	4.0031	2.9350	3.9689	1.1830
H	-4.9189	0.8620	0.5948	-3.9592	1.6808	2.5220	-2.1715	0.9135	2.9436	-3.0650	0.7188	1.9236
H	3.7817	1.0843	5.4558	5.8099	-0.0843	3.3966	4.0938	-0.2109	4.1394	1.1365	4.3799	3.4561

Supplementary S4. The Z-matrices of (4*S**,5*R**,9*S**,10*R**)-**6** optimized at B3LYP/6-31G(d)-GD3BJ level by Gaussian.

(4*S**,5*R**,9*S**,10*R**)-**6A**			
C	-2.6213	-0.8604	0.8885
C	-2.0764	-1.1379	2.2835
C	-1.2086	0.0181	2.8526
C	-0.0970	0.3649	1.7959
C	-0.5730	0.6261	0.3247
C	-1.5078	-0.5429	-0.1032
C	0.9053	1.4350	2.2252
C	1.8894	1.8982	1.2229
C	1.8423	1.5114	-0.0635
C	0.7160	0.6278	-0.6224
C	1.0248	-1.2914	-3.1814
C	1.1822	0.2192	-3.1774
C	0.3537	0.9552	-2.1065
C	-1.3328	1.9686	0.1819
C	-2.1526	1.1726	3.2920
C	-0.5472	-0.5498	4.1412
C	2.0218	-2.0688	-2.7117
C	2.9832	1.9147	-0.9601
C	-0.2841	-1.8058	-3.7237
O	0.9987	1.8733	3.3708
O	-1.5317	2.4405	3.3956
C	2.0209	-3.5413	-2.6567
O	2.2704	-4.1632	-1.6398
O	1.8001	-4.1068	-3.8582
H	-3.3542	-0.0480	0.9095
H	-3.1659	-1.7459	0.5382
H	-2.9147	-1.3528	2.9589
H	-1.4777	-2.0582	2.2388

(4*S**,5*R**,9*S**,10*R**)-**6B**		
-2.1854	-1.8806	1.3586
-1.6769	-1.9430	2.7932
-1.0979	-0.5961	3.3075
-0.0239	-0.0945	2.2741
-0.4609	-0.0378	0.7693
-1.1047	-1.4049	0.3939
0.7110	1.1881	2.6613
1.6299	1.7826	1.6661
1.7344	1.3108	0.4119
0.8481	0.1754	-0.1221
1.1599	-0.2251	-4.0260
1.5308	-0.3601	-2.5597
0.5081	0.3244	-1.6376
-1.4752	1.1007	0.4942
-2.2816	0.3627	3.6188
-0.4074	-0.9271	4.6618
0.7737	-1.3122	-4.7244
2.8124	1.8875	-0.4694
1.3487	1.1506	-4.6124
0.6522	1.7067	3.7751
-1.9402	1.7361	3.6418
0.3946	-1.3470	-6.1493
0.8249	-2.1814	-6.9255
-0.5334	-0.4253	-6.4685
-3.0696	-1.2400	1.2850
-2.5150	-2.8820	1.0558
-2.4910	-2.2818	3.4470
-0.9011	-2.7194	2.8460

(4*S**,5*R**,9*S**,10*R**)-**6C**		
-1.6473	-2.7805	0.4776
-1.2399	-3.1121	1.9076
-1.1209	-1.8645	2.8260
-0.1767	-0.8223	2.1220
-0.5097	-0.4570	0.6339
-0.6938	-1.7799	-0.1663
0.1181	0.4439	2.9253
0.8825	1.5205	2.2584
1.2058	1.4718	0.9546
0.7343	0.3413	0.0270
1.4557	1.2559	-3.7314
1.7068	0.6992	-2.3413
0.4704	0.8179	-1.4348
-1.7946	0.4005	0.5128
-2.5531	-1.3851	3.1946
-0.4553	-2.3722	4.1374
2.0874	2.3797	-4.1275
2.1194	2.5430	0.4159
0.5547	0.4237	-4.6077
-0.1649	0.5874	4.1136
-2.6356	-0.0364	3.6158
1.9594	3.0374	-5.4413
2.9220	3.4060	-6.0903
0.6794	3.2610	-5.7937
-2.6744	-2.4048	0.4410
-1.6448	-3.7039	-0.1144
-1.9591	-3.8270	2.3279
-0.2744	-3.6357	1.8739

(4*S**,5*R**,9*S**,10*R**)-**6D**		
-2.5340	-1.5096	1.2668
-2.0245	-1.8142	2.6695
-1.1808	-0.6657	3.2884
-0.0461	-0.2825	2.2692
-0.4882	0.0094	0.7932
-1.3963	-1.1608	0.3134
0.9353	0.7853	2.7506
1.9327	1.2919	1.7828
1.9213	0.9386	0.4860
0.8229	0.0516	-0.1198
0.9876	-0.0421	-4.0546
1.3555	-0.3655	-2.6175
0.4946	0.4068	-1.6032
-1.2612	1.3459	0.6633
-2.1444	0.4705	3.7325
-0.5476	-1.2609	4.5790
0.2127	-0.8961	-4.7538
3.0713	1.3904	-0.3765
1.5233	1.2661	-4.5765
1.0045	1.1840	3.9122
-1.5338	1.7383	3.8852
-0.2200	-0.7382	-6.1544
-1.3786	-0.8704	-6.5059
0.8076	-0.5281	-6.9985
-3.2744	-0.7041	1.2864
-3.0609	-2.3915	0.8821
-2.8789	-2.0505	3.3170
-1.4178	-2.7290	2.6202

(4*S**,5*R**,9*S**,10*R**)-**6E**		
-2.3396	-1.7803	1.4335
-1.8022	-1.8606	2.8565
-1.1442	-0.5420	3.3489
-0.0705	-0.1002	2.2882
-0.5386	-0.0301	0.7932
-1.2585	-1.3652	0.4416
0.7369	1.1456	2.6508
1.6545	1.6947	1.6287
1.7070	1.2124	0.3751
0.7595	0.1122	-0.1273
1.0575	-0.2915	-4.0323
1.3594	-0.5137	-2.5613
0.3985	0.2547	-1.6384
-1.5001	1.1567	0.5326
-2.2700	0.4773	3.6809
-0.4406	-0.9012	4.6893
1.8036	0.5811	-4.7399
2.7820	1.7431	-0.5386
-0.1060	-1.0773	-4.5796
0.7361	1.6659	3.7654
-1.8570	1.8308	3.6976
1.6554	0.8991	-6.1720
1.6054	2.0410	-6.5925
1.6770	-0.1956	-6.9554
-3.1918	-1.0963	1.3757
-2.7261	-2.7654	1.1448
-2.6173	-2.1541	3.5307
-1.0657	-2.6749	2.8969

H	0.5233	-0.5469	1.7438
H	-1.9793	-0.3357	-1.0693
H	-0.9088	-1.4529	-0.2424
H	2.6807	2.5259	1.6197
H	1.1190	-0.3929	-0.5923
H	2.2393	0.4862	-3.0922
H	0.8796	0.5934	-4.1647
H	0.4457	2.0356	-2.2779
H	-0.6956	0.7315	-2.3137
H	-0.7293	2.8235	0.5010
H	-1.6321	2.1569	-0.8540
H	-2.2582	1.9897	0.7563
H	-2.6037	0.9476	4.2661
H	-2.9970	1.2921	2.6101
H	0.1881	-1.3268	3.9038
H	-0.0443	0.2203	4.7315
H	-1.2984	-1.0057	4.7978
H	2.9081	-1.6321	-2.2599
H	2.6354	2.5242	-1.7985
H	3.7355	2.5100	-0.4297
H	3.4963	1.0265	-1.3405
H	-0.3473	-1.6083	-4.7985
H	-0.4207	-2.8791	-3.5672
H	-1.1290	-1.3134	-3.2343
H	-0.6776	2.3304	3.8650
H	1.7067	-3.4526	-4.5756

0.7731	-0.8573	2.3171
-1.5478	-1.3712	-0.6070
-0.3221	-2.1745	0.3560
2.2503	2.5886	2.0447
1.4541	-0.7367	-0.0139
1.5952	-1.4207	-2.2833
2.5364	0.0445	-2.4183
0.4004	1.3848	-1.8965
-0.4609	-0.1281	-1.8679
-2.4154	0.9628	1.0268
-1.0821	2.0831	0.7721
-1.7492	1.1535	-0.5643
-2.7271	0.1183	4.5908
-3.0955	0.2537	2.8990
0.4847	-1.5462	4.5147
-0.1086	-0.0316	5.2125
-1.0842	-1.4865	5.3193
0.7647	-2.2979	-4.2674
3.3835	2.6738	0.0378
3.5283	1.1089	-0.7489
2.3928	2.3385	-1.3727
0.5441	1.8176	-4.2884
2.3034	1.5758	-4.2842
1.3708	1.1462	-5.7057
-1.1150	1.8424	4.1612
-0.8384	0.0908	-5.6995

0.8056	-1.3249	2.0856
-1.0591	-1.5813	-1.1794
0.2822	-2.2697	-0.2839
1.2087	2.3203	2.9155
1.5748	-0.3682	-0.0113
1.9990	-0.3553	-2.4275
2.5647	1.2041	-1.8888
0.0901	1.8472	-1.4394
-0.3159	0.2189	-1.9012
-1.7298	1.3290	1.0877
-1.9998	0.6809	-0.5252
-2.6855	-0.1293	0.8478
-2.9704	-2.0061	3.9966
-3.2483	-1.4961	2.3599
0.5878	-2.6631	3.9696
-0.4723	-1.6253	4.9352
-0.9786	-3.2549	4.5251
2.8105	2.8730	-3.4839
1.6507	3.0972	-0.4016
2.3868	3.2803	1.1816
3.0560	2.0998	0.0652
-0.4903	0.5479	-4.3079
0.6396	0.6795	-5.6675
0.8154	-0.6368	-4.5224
-1.9153	0.1326	4.2597
0.0423	2.9927	-5.1060

0.5843	-1.1869	2.2103
-1.8411	-0.9412	-0.6629
-0.7825	-2.0608	0.1743
2.7029	1.9239	2.2131
1.2412	-0.9662	-0.1158
1.2315	-1.4415	-2.4366
2.4214	-0.1654	-2.4805
0.5838	1.4884	-1.7597
-0.5460	0.1677	-1.8406
-0.6779	2.1996	1.0209
-1.5338	1.5571	-0.3757
-2.2024	1.3414	1.2116
-2.6194	0.2175	4.6883
-2.9714	0.6023	3.0317
0.2016	-2.0244	4.3410
-0.0683	-0.5019	5.2025
-1.3110	-1.7409	5.2035
-0.2137	-1.7774	-4.2823
2.7272	1.9995	-1.2166
3.7899	2.0022	0.1810
3.6222	0.5243	-0.7550
2.6106	1.2075	-4.6889
1.0951	1.5494	-5.5415
1.2936	2.0821	-3.8843
-0.6948	1.6223	4.3794
1.6752	-0.5482	-6.5538

0.6881	-0.9018	2.3181
-1.7223	-1.3152	-0.5492
-0.5164	-2.1733	0.3913
2.3167	2.4771	1.9856
1.3256	-0.8254	-0.0184
1.2987	-1.5882	-2.3448
2.3970	-0.2348	-2.3562
0.3570	1.3132	-1.9242
-0.6035	-0.1335	-1.8353
-2.4362	1.0665	1.0823
-1.0544	2.1191	0.8007
-1.7893	1.2210	-0.5211
-2.7097	0.2558	4.6610
-3.1016	0.4114	2.9762
0.4161	-1.5646	4.5257
-0.0854	-0.0196	5.2288
-1.1294	-1.4234	5.3647
2.5691	1.1818	-4.2563
3.3912	2.5149	-0.0539
3.4649	0.9385	-0.8265
2.3576	2.1988	-1.4371
0.1434	-2.1427	-4.6117
-0.9905	-0.9505	-3.9479
-0.3980	-0.7653	-5.5857
-1.0140	1.8935	4.1947
1.8209	-1.0194	-6.4539

(4*S**,5*R**,9*S**,10*R**)-**6F**			
C	-2.1124	-2.2946	0.6529
C	-1.7875	-2.5414	2.1163
C	-1.5534	-1.2396	2.9310
C	-0.4850	-0.3693	2.1712
C	-0.6384	-0.1808	0.6164
C	-0.9746	-1.5497	-0.0360
C	-0.1829	0.9934	2.7840
C	0.8615	1.8087	2.1228
C	1.3317	1.4989	0.9011
C	0.7735	0.3334	0.0644
C	1.9501	0.5060	-3.6880
C	2.0253	0.2286	-2.1969
C	0.7362	0.6375	-1.4645
C	-1.7520	0.8326	0.2358
C	-2.9243	-0.5513	3.1847
C	-0.9673	-1.6906	4.2979
C	1.9041	-0.5208	-4.5611
C	2.4926	2.2974	0.3671
C	2.0266	1.9607	-4.0757
O	-0.7197	1.4360	3.7946
O	-2.9582	0.2693	4.3436
C	1.8433	-0.4172	-6.0312
O	2.5587	-1.0721	-6.7679
O	0.8637	0.4008	-6.4601
H	-3.0573	-1.7511	0.5534
H	-2.2587	-3.2593	0.1522
H	-2.5909	-3.1368	2.5688
H	-0.8885	-3.1717	2.1636
H	0.4546	-0.9326	2.3065

(4*S**,5*R**,9*S**,10*R**)-**6G**		
-2.1735	-2.3308	0.7254
-1.9458	-2.4802	2.2241
-1.6907	-1.1318	2.9523
-0.5294	-0.3793	2.2049
-0.6737	-0.2181	0.6517
-1.0147	-1.6109	0.0445
-0.0895	0.9405	2.8368
0.8948	1.7631	2.1003
1.3099	1.4512	0.8605
0.7331	0.2675	0.0683
1.9999	0.3068	-3.7179
1.9468	0.0780	-2.2119
0.6730	0.5274	-1.4704
-1.7801	0.7957	0.2656
-3.0452	-0.3812	3.0897
-1.2296	-1.5129	4.3885
0.9843	0.8404	-4.4279
2.4290	2.2683	0.2680
3.2891	-0.1819	-4.3380
-0.4346	1.3179	3.9555
-2.9297	1.0138	3.3003
0.9831	1.0907	-5.8823
0.0897	0.7120	-6.6181
2.0208	1.8518	-6.2792
-3.1154	-1.8116	0.5232
-2.2804	-3.3285	0.2822
-2.8080	-2.9952	2.6674
-1.0842	-3.1458	2.3729
0.3528	-1.0272	2.3493

(4*S**,5*R**,9*S**,10*R**)-**6H**		
-2.1605	-1.3748	2.0302
-1.6610	-0.8021	3.3455
-1.2880	0.7040	3.2686
-0.3002	0.9060	2.0604
-0.6442	0.2185	0.6871
-1.0978	-1.2445	0.9450
0.1147	2.3456	1.7779
1.0825	2.5615	0.6779
1.3859	1.5873	-0.1989
0.7004	0.2087	-0.1811
1.4699	-1.7775	-3.4914
1.6553	-1.2704	-2.0728
0.4841	-0.3900	-1.6044
-1.7685	0.9523	-0.0935
-2.5947	1.5448	3.2140
-0.5467	1.0277	4.5957
2.1096	-1.1620	-4.5066
2.4817	1.8528	-1.1988
0.5344	-2.9492	-3.6422
-0.2706	3.3184	2.4188
-2.4577	2.8649	3.7205
2.0493	-1.5344	-5.9319
1.8269	-0.7265	-6.8157
2.3549	-2.8277	-6.1477
-3.0947	-0.8909	1.7283
-2.3959	-2.4368	2.1699
-2.4177	-0.9696	4.1225
-0.7819	-1.3832	3.6573
0.6329	0.4260	2.4026

(4*S**,5*R**,9*S**,10*R**)-**6I**		
-2.2517	-1.8364	-0.4763
-3.2325	-0.7131	-0.1680
-2.9047	0.6161	-0.9018
-1.4049	0.9853	-0.6061
-0.3345	-0.1325	-0.8608
-0.8145	-1.4372	-0.1608
-0.9312	2.3154	-1.1890
0.5050	2.6474	-1.0713
1.4085	1.7777	-0.5877
1.0437	0.3363	-0.1954
2.6647	-1.3616	1.9834
3.2319	-0.8298	0.6813
2.1978	-0.6859	-0.4521
-0.1202	-0.4119	-2.3694
-3.3154	0.4700	-2.3940
-3.8425	1.6846	-0.2697
2.5804	-0.5624	3.0652
2.8134	2.2754	-0.3736
2.2609	-2.8143	1.9714
-1.6802	3.1629	-1.6726
-2.6895	1.3856	-3.2739
1.9782	-0.9768	4.3477
0.9235	-1.5744	4.4573
2.7391	-0.5875	5.3887
-2.3373	-2.1548	-1.5198
-2.5163	-2.7135	0.1270
-4.2481	-1.0461	-0.4186
-3.2267	-0.5438	0.9177
-1.3789	1.1702	0.4820

(4*S**,5*R**,9*S**,10*R**)-**6J**		
-2.3314	-1.6469	-0.4243
-3.1918	-0.5010	0.0801
-2.8873	0.8573	-0.6097
-1.3389	1.1218	-0.5131
-0.3523	-0.0539	-0.8618
-0.8537	-1.3592	-0.1850
-0.8368	2.3812	-1.2094
0.6068	2.6863	-1.0847
1.4837	1.7786	-0.6197
1.0789	0.3384	-0.2572
2.7043	-1.4653	1.8083
3.2433	-0.9657	0.4799
2.1757	-0.7158	-0.6041
-0.2265	-0.3074	-2.3884
-3.4783	0.8343	-2.0475
-3.6428	1.9350	0.2166
2.1253	-2.6806	1.8875
2.9054	2.2203	-0.3936
2.8446	-0.5012	2.9578
-1.5488	3.1652	-1.8293
-3.7907	2.1179	-2.5703
1.5471	-3.2877	3.0991
0.4201	-3.7482	3.1304
2.4206	-3.3455	4.1214
-2.5293	-1.8419	-1.4830
-2.6055	-2.5626	0.1134
-4.2504	-0.7636	-0.0422
-3.0326	-0.4072	1.1635
-1.1766	1.3317	0.5583

H	-1.2453	-1.4282	-1.0904	-1.2583	-1.5292	-1.0199	-1.4957	-1.7010	0.0322	-0.1751	-2.2846	-0.4286	-0.2843	-2.2278	-0.5330
H	-0.0834	-2.1911	-0.0201	-0.1298	-2.2584	0.1069	-0.2299	-1.8503	1.2377	-0.7279	-1.3162	0.9273	-0.6879	-1.2960	0.8985
H	1.2384	2.6395	2.7103	1.2952	2.6019	2.6607	1.5475	3.5417	0.6591	0.7601	3.6633	-1.3552	0.8891	3.6974	-1.3590
H	1.4857	-0.4925	0.2122	1.4422	-0.5585	0.2295	1.4048	-0.4552	0.3427	0.8656	0.3742	0.8876	0.9546	0.3460	0.8335
H	2.2029	-0.8405	-2.0209	2.0776	-0.9976	-2.0330	1.7637	-2.1303	-1.3991	3.7479	0.1184	0.8530	3.8520	-0.0712	0.6392
H	2.9000	0.7448	-1.7927	2.8201	0.5741	-1.7794	2.6007	-0.7248	-2.0018	4.0189	-1.5178	0.3447	3.9489	-1.7154	0.0957
H	0.5169	1.6993	-1.6308	0.4676	1.5870	-1.6657	0.2943	0.4106	-2.3305	2.7283	-0.4177	-1.3750	2.6854	-0.4279	-1.5328
H	-0.0776	0.0914	-1.9510	-0.1555	-0.0255	-1.9209	-0.4095	-1.0185	-1.6263	1.8010	-1.6874	-0.6388	1.7246	-1.6879	-0.8221
H	-1.6374	1.7965	0.7395	-2.7725	0.4728	0.5777	-1.9056	0.5346	-1.0959	0.1867	0.4831	-2.9187	0.0316	0.5963	-2.9475
H	-1.7635	1.0376	-0.8394	-1.6062	1.7851	0.6989	-2.7427	0.8551	0.3859	0.6523	-1.1699	-2.5342	0.5457	-1.0498	-2.6135
H	-2.7514	0.4598	0.4597	-1.8482	0.9289	-0.8187	-1.5679	2.0196	-0.2218	-1.0139	-0.8020	-2.8550	-1.1397	-0.7146	-2.8220
H	-3.7000	-1.3095	3.3408	-3.6277	-0.7922	3.9232	-3.3686	1.0740	3.8307	-4.3992	0.5961	-2.5060	-4.4227	0.2784	-2.0545
H	-3.2565	0.0551	2.3462	-3.6775	-0.5147	2.2095	-3.0088	1.6215	2.2120	-3.1055	-0.5295	-2.7801	-2.8294	0.3395	-2.7655
H	-0.0462	-2.2699	4.1641	-0.2409	-1.9854	4.3786	0.3327	0.3865	4.7267	-3.5655	1.8947	0.7695	-3.3297	1.9227	1.2671
H	-0.7223	-0.8466	4.9490	-1.1842	-0.6514	5.0595	-0.1965	2.0630	4.6400	-3.8348	2.6302	-0.8178	-3.4698	2.9485	-0.1567
H	-1.6790	-2.3280	4.8356	-1.9248	-2.2279	4.8455	-1.2029	0.8630	5.4584	-4.8828	1.3367	-0.2629	-4.7240	1.7547	0.1956
H	1.9642	-1.5504	-4.2193	0.0309	1.0910	-3.9751	2.6974	-0.2646	-4.3344	2.9095	0.4702	3.0243	1.9855	-3.2948	1.0025
H	2.2319	2.8052	-0.5655	2.1262	2.7450	-0.6680	2.8998	2.8607	-1.0944	2.9301	3.3247	-0.6684	3.0506	3.2811	-0.6282
H	2.8171	3.0740	1.0693	2.7527	3.0725	0.9389	3.3079	1.1514	-1.0489	3.0771	2.2162	0.6866	3.1795	2.0891	0.6573
H	3.3539	1.6442	0.1982	3.3041	1.6367	0.0892	2.1158	1.7691	-2.2256	3.5336	1.7029	-0.9643	3.5997	1.6594	-1.0254
H	1.0728	2.4592	-3.8783	4.1176	0.4705	-4.0444	-0.4174	-2.7520	-3.1395	1.3125	-2.9454	1.4439	3.8962	-0.4177	3.2501
H	2.8075	2.4673	-3.4982	3.5118	-1.1982	-3.9950	0.2926	-3.1726	-4.6846	3.0235	-3.4146	1.4635	2.4890	0.4932	2.6695
H	2.2773	2.1059	-5.1302	3.2555	-0.2237	-5.4298	0.9797	-3.8440	-3.1959	2.1551	-3.2371	2.9741	2.2665	-0.7960	3.8375
H	-2.1993	0.8905	4.2986	-2.2445	1.1693	3.9844	-1.6946	3.2857	3.2683	-2.7166	2.2766	-2.8652	-2.9995	2.6892	-2.4649
H	0.3228	0.7618	-5.7337	2.5867	2.1383	-5.5385	2.6007	-3.3023	-5.3321	3.6013	-0.2102	5.1347	3.3059	-3.0109	3.8858

(4*S**,5*R**,9*S**,10*R**)-**6K**			
C	-2.6742	-0.7617	0.6753
C	-2.2270	-1.0861	2.0948
C	-1.3378	0.0159	2.7342
C	-0.1520	0.3266	1.7489
C	-0.5302	0.6342	0.2583
C	-1.4889	-0.4860	-0.2432
C	0.8748	1.3383	2.2558
C	1.9335	1.7725	1.3188
C	1.9413	1.4112	0.0243
C	0.8092	0.5947	-0.6155
C	1.2537	-1.2738	-3.1567
C	1.4051	0.2371	-3.1544
C	0.5441	0.9701	-2.1077
C	-1.2213	2.0114	0.0989
C	-2.2474	1.2084	3.1434
C	-0.7796	-0.6065	4.0464
C	0.0979	-1.8317	-3.5716
C	3.1471	1.7777	-0.8005
C	2.4933	-2.0400	-2.7728
O	0.9283	1.7434	3.4158
O	-1.5704	2.4418	3.2971
C	-0.2087	-3.2723	-3.6419
O	-0.7199	-3.7914	-4.6178
O	0.0459	-3.9187	-2.4885
H	-3.3666	0.0856	0.6676
H	-3.2391	-1.6141	0.2784
H	-3.1133	-1.2684	2.7163
H	-1.6730	-2.0347	2.0688
H	0.4262	-0.6134	1.7138

H	-1.8892	-0.2490	-1.2344
H	-0.9241	-1.4210	-0.3559
H	2.7291	2.3563	1.7710
H	1.1608	-0.4449	-0.5887
H	2.4589	0.5074	-3.0481
H	1.1235	0.6113	-4.1486
H	0.6736	2.0513	-2.2460
H	-0.4975	0.7774	-2.3792
H	-0.5977	2.8331	0.4635
H	-1.4571	2.2279	-0.9478
H	-2.1739	2.0664	0.6243
H	-2.7587	0.9935	4.0896
H	-3.0484	1.3778	2.4208
H	-0.0688	-1.4133	3.8352
H	-0.2766	0.1279	4.6806
H	-1.5882	-1.0377	4.6493
H	-0.7125	-1.2145	-3.9505
H	2.8746	2.4292	-1.6353
H	3.9013	2.3155	-0.2145
H	3.6313	0.8740	-1.1829
H	2.7078	-1.9098	-1.7079
H	2.4163	-3.1112	-2.9765
H	3.3537	-1.6758	-3.3447
H	-0.7577	2.2869	3.8238
H	0.3687	-3.3286	-1.7833

Supplementary S5. The Z-matrices of (2*R**,4*S**,5*R**,9*S**,10*R**)-**7** optimized at B3LYP/6-31G(d)-GD3BJ level by Gaussian.

(2*R**,4*S**,5*R**,9*S**,10*R**)-**7A**			
C	-2.2338	-0.2594	-1.1926
C	-1.5608	-1.1612	-2.2232
C	-0.6772	-2.2774	-1.5908
C	0.3368	-1.6007	-0.5970
C	-0.2718	-0.6348	0.4713
C	-1.2200	0.3489	-0.2435
C	1.3591	-2.5245	0.0678
C	2.2407	-1.9387	1.1083
C	2.0761	-0.6834	1.5638
C	0.9225	0.2113	1.0985
C	0.6252	3.3627	0.6866
C	0.9933	2.6632	1.9882
C	0.4294	1.2391	2.1669
C	-1.0474	-1.3851	1.5809
C	-1.6078	-3.3726	-1.0029
C	0.1021	-2.9201	-2.7724
C	-0.6602	3.6018	0.3502
C	3.1136	-0.1077	2.4924
C	1.8056	3.7757	-0.1569
O	1.5418	-3.6944	-0.2636
O	-2.8588	0.8545	-1.8405
O	-1.0097	-4.1777	-0.0061
C	-1.0781	4.3225	-0.8684
O	-0.6559	5.4207	-1.1863
O	-1.9985	3.6424	-1.5781
H	-3.0120	-0.7930	-0.6395
H	-2.3283	-1.5989	-2.8757
H	-0.9493	-0.5342	-2.8880

(2*R**,4*S**,5*R**,9*S**,10*R**)-**7B**		
-2.1406	0.2354	-1.4603
-1.3793	-0.1897	-2.7124
-0.5073	-1.4594	-2.5107
0.4179	-1.2253	-1.2611
-0.2832	-0.7523	0.0577
-1.2028	0.4505	-0.2791
1.4145	-2.3432	-0.9523
2.2249	-2.2183	0.2807
2.0064	-1.2481	1.1854
0.8468	-0.2495	1.0626
0.3320	2.7446	2.2705
0.8644	1.5075	2.9775
0.2647	0.1945	2.4421
-1.1214	-1.8777	0.7127
-1.4429	-2.6994	-2.4759
0.3664	-1.5789	-3.7918
1.1158	3.4195	1.4055
2.9908	-1.0932	2.3170
-1.0499	3.1797	2.6894
1.6399	-3.2898	-1.7040
-2.7878	1.4832	-1.7079
-0.8967	-3.8313	-1.8278
0.7563	4.6363	0.6538
1.4969	5.6015	0.5811
-0.4117	4.5334	-0.0062
-2.9205	-0.4864	-1.2015
-2.0915	-0.3374	-3.5357
-0.7465	0.6497	-3.0355

(2*R**,4*S**,5*R**,9*S**,10*R**)-**7C**		
-2.0878	0.5837	-1.2113
-1.4268	0.0760	-2.4841
-0.8130	-1.3454	-2.3321
0.1542	-1.3416	-1.0963
-0.3864	-0.7279	0.2413
-1.0942	0.6050	-0.0648
0.8420	-2.6647	-0.7649
1.7607	-2.6657	0.4043
1.8112	-1.6345	1.2689
0.8823	-0.4182	1.1543
1.1528	2.6741	1.9279
1.3080	1.4933	2.8770
0.4942	0.2333	2.5198
-1.3761	-1.6613	0.9874
-1.9168	-2.4284	-2.2677
0.0058	-1.5949	-3.6293
-0.0480	3.2478	1.6997
2.8771	-1.6294	2.3337
2.4418	3.1472	1.3033
0.7127	-3.6970	-1.4135
-2.5002	1.9458	-1.3736
-2.8474	-2.2581	-3.3352
-0.2520	4.4291	0.8383
0.3671	5.4720	0.9608
-1.2180	4.2377	-0.0798
-2.9752	-0.0050	-0.9605
-2.1466	0.1176	-3.3113
-0.6402	0.7885	-2.7734

(2*R**,4*S**,5*R**,9*S**,10*R**)-**7D**		
2.0301	1.1650	-1.0620
2.1071	0.0523	-2.0967
0.7520	-0.2246	-2.7988
-0.3335	-0.4789	-1.6954
-0.4149	0.5339	-0.4969
1.0150	0.8249	0.0245
-1.7504	-0.7354	-2.2026
-2.7835	-1.0148	-1.1757
-2.5544	-0.8041	0.1335
-1.2490	-0.1826	0.6597
0.2085	-0.2522	3.5778
-1.2525	0.0166	3.2522
-1.4573	0.7339	1.9056
-1.0874	1.8778	-0.8855
0.3985	0.9055	-3.7970
0.9600	-1.5233	-3.6279
0.6937	-1.5090	3.5207
-3.5914	-1.2678	1.1246
0.9936	0.9458	4.0500
-2.0747	-0.7643	-3.3844
3.2969	1.2990	-0.4188
1.5067	1.1842	-4.6515
2.0721	-1.9385	3.8218
2.3155	-2.9094	4.5170
3.0110	-1.2144	3.1861
1.7979	2.1261	-1.5302
2.8971	0.2809	-2.8234
2.4582	-0.8617	-1.5951

(2*R**,4*S**,5*R**,9*S**,10*R**)-**7E**		
-2.2002	0.4045	-1.2682
-1.5367	-0.0945	-2.5447
-0.8188	-1.4591	-2.3726
0.1680	-1.3471	-1.1578
-0.3816	-0.7373	0.1803
-1.1818	0.5503	-0.1419
0.9571	-2.6128	-0.8314
1.9008	-2.5372	0.3101
1.9053	-1.4928	1.1586
0.8846	-0.3458	1.0687
0.9107	2.7404	2.1304
1.3042	1.4747	2.8763
0.4848	0.2401	2.4592
-1.2958	-1.7188	0.9608
-1.8544	-2.6045	-2.2506
-0.0065	-1.6823	-3.6791
1.7310	3.2546	1.1918
3.0047	-1.4119	2.1871
-0.3695	3.3887	2.5941
0.8995	-3.6535	-1.4775
-2.7482	1.7015	-1.5017
-2.7482	-2.6129	-3.3627
1.5078	4.4751	0.3945
2.3780	5.3116	0.2257
0.2976	4.5207	-0.1913
-3.0247	-0.2484	-0.9674
-2.2810	-0.1348	-3.3505
-0.8140	0.6648	-2.8784

H	0.9660	-0.9675	-1.2468	1.0722	-0.3885	-1.5621	0.9858	-0.6893	-1.4143	-0.0362	-1.4438	-1.2494	0.9419	-0.6422	-1.5079
H	-0.6160	1.0579	-0.8250	-0.5845	1.3331	-0.4940	-0.3343	1.3586	-0.3087	1.3884	-0.0463	0.5801	-0.4862	1.3559	-0.4143
H	-1.7795	0.9530	0.4782	-1.8143	0.7366	0.5839	-1.6221	0.9836	0.8171	1.0124	1.6451	0.7518	-1.7112	0.9198	0.7438
H	3.0565	-2.5781	1.4298	3.0412	-2.9285	0.3650	2.4102	-3.5317	0.4785	-3.7061	-1.4418	-1.5550	2.6122	-3.3542	0.3735
H	1.3583	0.7886	0.2709	1.3023	0.6355	0.5964	1.4843	0.3198	0.6052	-0.6485	-1.0448	0.9823	1.4135	0.4453	0.5188
H	2.0833	2.6344	2.0981	1.9575	1.4766	2.9242	2.3666	1.2232	2.9634	-1.8283	-0.9142	3.2789	2.3748	1.2779	2.7588
H	0.6364	3.2862	2.8191	0.6296	1.5924	4.0464	1.0121	1.8266	3.8807	-1.6749	0.6399	4.0509	1.1588	1.6477	3.9504
H	0.6737	0.8890	3.1764	0.4178	-0.5959	3.1865	0.5995	-0.4936	3.3335	-2.4638	1.1693	1.8855	0.5856	-0.5301	3.2334
H	-0.6597	1.3276	2.1750	-0.8198	0.3248	2.4021	-0.5611	0.5162	2.5502	-0.7834	1.5947	1.8909	-0.5704	0.5246	2.4896
H	-0.4392	-2.1510	2.0708	-0.5234	-2.7675	0.9308	-1.5497	-1.3358	2.0174	-1.2932	2.5010	-0.0101	-1.5143	-1.3600	1.9710
H	-1.3946	-0.7071	2.3665	-1.5703	-1.5573	1.6577	-2.3642	-1.6712	0.5247	-0.4513	2.4933	-1.5218	-2.2722	-1.8422	0.4927
H	-1.9459	-1.8724	1.2013	-1.9591	-2.1900	0.0895	-1.0260	-2.6958	1.0428	-2.0408	1.7406	-1.4031	-0.8531	-2.7127	1.0702
H	-1.9673	-4.0393	-1.7960	-1.7192	-3.0020	-3.4932	-2.4949	-2.3864	-1.3484	0.1492	1.8412	-3.3044	-2.4850	-2.4972	-1.3722
H	-2.5095	-2.9443	-0.5597	-2.3900	-2.4804	-1.9779	-1.5003	-3.4355	-2.3563	-0.4423	0.6306	-4.4392	-1.3903	-3.5923	-2.1946
H	0.6382	-3.8262	-2.4785	0.9062	-2.5279	-3.8454	0.4287	-2.6035	-3.6602	0.0765	-1.7770	-4.2214	0.4415	-2.6794	-3.7189
H	-0.5811	-3.2081	-3.5808	-0.2534	-1.5198	-4.6949	-0.6178	-1.4774	-4.5231	1.8003	-1.4213	-4.3244	-0.6392	-1.5760	-4.5678
H	0.8301	-2.2206	-3.1984	1.1018	-0.7687	-3.8512	0.8364	-0.8854	-3.7187	1.1794	-2.3793	-2.9796	0.8031	-0.9496	-3.7749
H	-1.4781	3.2982	0.9935	2.1456	3.1174	1.2353	-0.9492	2.8833	2.1790	0.0430	-2.3464	3.2836	2.6961	2.7991	0.9870
H	3.5288	0.8136	2.0732	3.4459	-0.0988	2.2894	3.4817	-0.7204	2.2605	-4.4435	-1.7505	0.6325	3.5876	-0.4969	2.0461
H	2.6921	0.1040	3.4786	2.5133	-1.2443	3.2886	2.4406	-1.6901	3.3341	-3.1589	-2.0065	1.8061	2.6048	-1.4312	3.2042
H	3.9538	-0.7946	2.6453	3.8102	-1.8181	2.2513	3.5629	-2.4781	2.2314	-3.9925	-0.4332	1.7054	3.7059	-2.2504	2.1061
H	2.4746	2.9225	-0.3128	-1.1421	3.1521	3.7805	2.9676	2.3050	0.8406	0.4103	1.5194	4.7783	-0.4072	3.4155	3.6885
H	1.5270	4.1384	-1.1492	-1.2829	4.2042	2.3864	2.2981	3.8936	0.5184	1.9267	0.6717	4.5499	-0.4671	4.4245	2.2573
H	2.3689	4.5666	0.3489	-1.8084	2.5174	2.2633	3.0927	3.5802	2.0698	1.2365	1.6023	3.2099	-1.2371	2.8292	2.2335
H	-3.4905	0.4942	-2.4917	-3.4113	1.3519	-2.4445	-3.0821	1.9716	-2.1579	3.9532	1.4893	-1.1138	-3.4110	1.6098	-2.2104
H	-0.1215	-4.4560	-0.3165	0.0179	-3.9690	-2.1554	-3.3763	-3.0741	-3.3817	1.1698	1.7363	-5.3791	-2.3036	-3.0953	-4.0823
H	-2.1814	2.7118	-1.3199	-0.8387	3.6586	0.0675	-1.5707	3.3273	-0.1969	2.6534	-0.5345	2.5838	-0.2507	3.7267	-0.0433

(2R*,4S*,5R*,9S*,10R*)-**7F**			
C	2.6778	0.8501	-1.2788
C	2.8194	-0.1771	-2.3977
C	1.4798	-0.5119	-3.1072
C	0.4272	-0.9135	-2.0091
C	0.2519	0.0710	-0.8015
C	1.6608	0.4015	-0.2331
C	-0.9387	-1.3507	-2.5372
C	-2.0077	-1.6100	-1.5485
C	-1.8546	-1.3464	-0.2393
C	-0.5956	-0.6743	0.3292
C	-1.0666	0.4581	4.0701
C	-0.8213	-0.4768	2.8991
C	-0.8920	0.2540	1.5477
C	-0.4576	1.3856	-1.2116
C	1.1102	0.6731	-4.0428
C	1.7807	-1.7378	-4.0162
C	-0.0627	0.7435	4.9242
C	-2.9439	-1.7940	0.7012
C	-2.4886	0.9328	4.2278
O	-1.1773	-1.5781	-3.7221
O	3.9360	0.9973	-0.6235
O	-0.2630	0.7611	-4.3691
C	-0.1500	1.6195	6.1075
O	0.2718	1.2943	7.2028
O	-0.6626	2.8320	5.8237
H	2.4078	1.8342	-1.6721
H	3.5624	0.1791	-3.1244
H	3.2572	-1.0928	-1.9744
H	0.8315	-1.8429	-1.5713

(2R*,4S*,5R*,9S*,10R*)-**7G**		
-2.1661	0.1847	-1.3196
-1.3566	-0.1032	-2.5756
-0.4464	-1.3622	-2.4620
0.4309	-1.2205	-1.1630
-0.2882	-0.7970	0.1656
-1.2490	0.3691	-0.1267
1.3622	-2.3831	-0.8322
2.2194	-2.2368	0.3720
2.0114	-1.2570	1.2717
0.8431	-0.2685	1.1575
0.4185	2.7615	2.0774
0.7748	1.5919	2.9849
0.2649	0.2106	2.5269
-1.0900	-1.9518	0.8221
-1.3345	-2.6337	-2.5330
0.4785	-1.3356	-3.7096
-0.8648	3.0841	1.8087
3.0104	-1.0660	2.3834
1.6062	3.5221	1.5423
1.4565	-3.4033	-1.5071
-2.8624	1.4280	-1.4518
-0.6531	-3.7981	-2.9714
-1.2745	4.2371	0.9829
-0.8896	5.3781	1.1709
-2.1419	3.8918	0.0125
-2.9095	-0.5969	-1.1368
-2.0353	-0.1905	-3.4346
-0.7420	0.7808	-2.8002
1.1221	-0.3952	-1.4067

(2R*,4S*,5R*,9S*,10R*)-**7H**		
-2.2583	-0.5307	-2.1504
-1.5066	-1.4022	-3.1517
-0.5342	-2.4141	-2.4879
0.4069	-1.6225	-1.5065
-0.2876	-0.6706	-0.4721
-1.2984	0.2285	-1.2387
1.4860	-2.4560	-0.8161
2.3103	-1.7940	0.2183
2.0538	-0.5481	0.6528
0.8357	0.2600	0.1792
0.3455	3.4787	2.3888
0.8725	2.6064	1.2632
0.2548	1.1984	1.2818
-1.0341	-1.4520	0.6379
-1.3717	-3.5755	-1.8833
0.3078	-3.0124	-3.6509
1.1614	3.8279	3.4042
3.0470	0.0882	1.5915
-1.0712	3.9651	2.2183
1.7631	-3.6138	-1.1250
-3.0313	0.4325	-2.8639
-0.7246	-4.2950	-0.8520
0.8088	4.6716	4.5614
1.5145	5.5854	4.9497
-0.3134	4.2662	5.1851
-2.9594	-1.1215	-1.5543
-2.2304	-1.9256	-3.7913
-0.9514	-0.7442	-3.8360
0.9923	-0.9587	-2.1665

(2R*,4S*,5R*,9S*,10R*)-**7I**		
-2.3622	0.6182	-1.2606
-1.9103	-0.0969	-2.5302
-1.3367	-1.5163	-2.2716
-0.2142	-1.4011	-1.1753
-0.5879	-0.6435	0.1462
-1.2360	0.7177	-0.2355
0.5242	-2.6993	-0.8486
1.4991	-2.6749	0.2642
1.6484	-1.6051	1.0643
0.7606	-0.3569	0.9511
1.4274	2.6792	1.8417
1.5066	1.4332	2.7077
0.4946	0.3354	2.3260
-1.5695	-1.4534	1.0293
-2.5199	-2.4794	-1.9740
-0.7062	-1.9669	-3.6203
2.3542	2.8927	0.8859
2.7829	-1.6164	2.0562
0.2638	3.5944	2.1290
0.4212	-3.7342	-1.5051
-2.7988	1.9343	-1.5956
-2.1609	-3.6496	-1.2639
2.3833	4.0378	-0.0397
2.4133	3.9040	-1.2500
2.4459	5.2205	0.5987
-3.2272	0.1153	-0.8201
-2.7496	-0.1408	-3.2377
-1.1530	0.5243	-3.0302
0.5687	-0.7860	-1.6525

(2R*,4S*,5R*,9S*,10R*)-**7J**		
-2.3010	0.7749	-1.9692
-1.8178	0.1231	-3.2609
-1.1710	-1.2723	-3.0506
-0.0527	-1.1368	-1.9523
-0.4605	-0.4412	-0.6076
-1.1734	0.8979	-0.9482
0.7476	-2.4076	-1.6693
1.7173	-2.3762	-0.5528
1.8155	-1.3291	0.2842
0.8730	-0.1179	0.2098
1.2495	2.2167	3.3572
1.5585	1.6205	1.9961
0.5699	0.5091	1.6057
-1.4033	-1.3228	0.2488
-2.3032	-2.3032	-2.7847
-0.5224	-1.6444	-4.4147
0.5704	3.3788	3.4402
2.9393	-1.3323	1.2884
1.7270	1.4076	4.5354
0.6958	-3.4218	-2.3631
-2.7638	2.0914	-2.2646
-1.8872	-3.4766	-2.1137
0.2098	4.0893	4.6809
-0.9184	4.4847	4.9132
1.2727	4.3256	5.4732
-3.1449	0.2295	-1.5375
-2.6557	0.0573	-3.9685
-1.0968	0.7999	-3.7421
0.6985	-0.4693	-2.4095

H	2.0637	-0.4801	0.2856
H	1.6095	1.1802	0.5366
H	-2.8985	-2.0795	-1.9535
H	0.0297	-1.5020	0.6965
H	0.1673	-0.9453	2.9919
H	-1.5442	-1.2951	2.9529
H	-1.8661	0.7418	1.4208
H	-0.1662	1.0705	1.6039
H	-0.5991	2.0543	-0.3566
H	0.1159	1.9632	-1.9358
H	-1.4463	1.2061	-1.6443
H	1.6700	0.6095	-4.9838
H	1.3910	1.6360	-3.6106
H	0.9596	-1.9660	-4.7005
H	2.6650	-1.5553	-4.6391
H	1.9842	-2.6357	-3.4219
H	0.9149	0.2817	4.8171
H	-3.7733	-2.2775	0.1721
H	-2.5517	-2.5289	1.4104
H	-3.3715	-0.9502	1.2494
H	-3.1852	0.0997	4.0842
H	-2.6921	1.3399	5.2222
H	-2.7173	1.7059	3.4880
H	4.5763	1.3239	-1.2795
H	-0.5782	-0.1297	-4.6338
H	-0.8501	2.9518	4.8742

-0.6591	1.2773	-0.3058
-1.8812	0.5878	0.7407
3.0377	-2.9453	0.4470
1.2911	0.6098	0.6715
1.8610	1.5462	3.1228
0.3658	1.8016	3.9822
0.4853	-0.5225	3.3117
-0.8266	0.2645	2.5109
-1.4023	-1.7063	1.8416
-2.0165	-2.1675	0.2875
-0.5200	-2.8827	0.8869
-2.1459	-2.4797	-3.2535
-1.8129	-2.8700	-1.5860
1.1932	-2.1635	-3.7207
-0.1106	-1.3955	-4.6323
1.0608	-0.4079	-3.7530
-1.6890	2.5168	2.2256
3.4079	-0.0468	2.3644
2.5596	-1.2567	3.3609
3.8657	-1.7443	2.2871
2.3182	2.8312	1.0779
1.3446	4.2589	0.7791
2.1157	4.0438	2.3589
-3.4199	1.3655	-2.2508
0.1772	-3.8892	-2.4555
-2.2947	2.9344	-0.1474

-0.7498	0.9615	-1.8473
-1.9008	0.8315	-0.5495
3.1667	-2.3695	0.5550
1.2157	0.9055	-0.6271
0.6533	3.0919	0.3035
1.9630	2.5480	1.3189
0.3716	0.7429	2.2733
-0.8224	1.3289	1.1510
-0.3706	-2.1240	1.1904
-1.4908	-0.7797	1.3711
-1.8555	-2.0522	0.2480
-1.6512	-4.2947	-2.6628
-2.3193	-3.2223	-1.4708
0.9141	-3.8656	-3.3360
-0.3411	-3.3716	-4.4591
0.9793	-2.2630	-4.0849
2.2068	3.5317	3.4096
3.4948	0.9712	1.1261
2.5797	0.3732	2.5378
3.8718	-0.5895	1.8406
-1.7798	3.1596	2.4335
-1.3091	4.8131	2.8665
-1.2355	4.3049	1.1900
-3.6718	-0.0502	-3.4150
0.1865	-4.5099	-1.1461
-0.6932	3.4561	4.7977

-0.4653	1.3929	-0.6330
-1.6337	1.2314	0.6469
2.1216	-3.5604	0.3436
1.3433	0.3379	0.3325
2.5249	1.0328	2.6995
1.3250	1.7297	3.7492
0.4753	-0.4177	3.1238
-0.4982	0.7906	2.3566
-1.7995	-0.9340	1.9647
-2.5327	-1.6137	0.5461
-1.1706	-2.4356	1.2996
-3.0033	-2.7944	-2.9067
-3.3082	-1.9923	-1.3963
-0.4174	-3.0210	-3.6188
-1.4166	-1.8387	-4.4463
0.1825	-1.3738	-3.8639
3.1270	2.1586	0.6768
3.4773	-0.7979	1.8442
2.4191	-1.5269	3.0831
3.3641	-2.5444	2.0075
0.3902	4.0619	3.1108
0.1503	4.3897	1.3876
-0.6783	3.0391	2.1332
-2.0585	2.4057	-2.0168
-1.3618	-4.0348	-1.6826
2.4816	5.1291	1.5694

-0.4371	1.6190	-1.3311
-1.5875	1.3707	-0.0502
2.3768	-3.2364	-0.4983
1.4231	0.6370	-0.3720
1.5234	2.4069	1.2302
2.5914	1.2618	2.0004
0.5384	-0.2741	2.3725
-0.4242	0.9654	1.6227
-1.6587	-0.8413	1.1980
-2.3573	-1.5152	-0.2409
-0.9580	-2.2922	0.4917
-2.7719	-2.6108	-3.7274
-3.1140	-1.8741	-2.1921
-0.1811	-2.6821	-4.4496
-1.2405	-1.5241	-5.2350
0.3348	-0.9995	-4.6388
0.1749	3.8608	2.5501
3.6125	-0.4899	1.1044
2.5619	-1.2795	2.3131
3.5474	-2.2420	1.2241
2.8202	1.4312	4.5877
1.3318	1.7664	5.4894
1.4107	0.3641	4.4410
-3.5336	2.0093	-2.8541
-1.0684	-3.8074	-2.5410
2.1124	4.0212	5.0817

(2*R**,4*S**,5*R**,9*S**,10*R**)-**7K**			
C	2.6729	0.9586	-1.4113
C	2.8093	-0.0909	-2.5097
C	1.4593	-0.4797	-3.1703
C	0.4508	-0.8820	-2.0319
C	0.2843	0.1270	-0.8433
C	1.6999	0.5085	-0.3252
C	-0.9174	-1.3694	-2.5080
C	-1.9525	-1.6260	-1.4829
C	-1.7689	-1.3260	-0.1855
C	-0.5085	-0.6134	0.3287
C	-0.9744	0.5273	4.0677
C	-0.6163	-0.3719	2.8982
C	-0.7862	0.3295	1.5401
C	-0.4732	1.4115	-1.2631
C	1.0292	0.6709	-4.1225
C	1.7679	-1.7191	-4.0582
C	-2.1746	0.3919	4.6675
C	-2.8242	-1.7681	0.7961
C	0.0744	1.5402	4.4486
O	-1.1811	-1.6398	-3.6785
O	3.9464	1.1534	-0.7988
O	-0.3534	0.7078	-4.4169
C	-2.6707	1.1665	5.8198
O	-3.7709	1.6888	5.8387
O	-1.8232	1.1495	6.8657
H	2.3649	1.9257	-1.8188
H	3.5201	0.2677	-3.2668
H	3.2850	-0.9840	-2.0788
H	0.8933	-1.7891	-1.5842

(2*R**,4*S**,5*R**,9*S**,10*R**)-**7L**		
2.7043	0.8329	-1.3124
2.8340	-0.1961	-2.4304
1.4905	-0.5202	-3.1361
0.4408	-0.9173	-2.0342
0.2762	0.0666	-0.8239
1.6894	0.3916	-0.2606
-0.9297	-1.3463	-2.5574
-1.9946	-1.6039	-1.5638
-1.8343	-1.3437	-0.2548
-0.5705	-0.6768	0.3092
-1.0605	0.4474	4.0500
-0.7874	-0.4808	2.8796
-0.8611	0.2514	1.5292
-0.4286	1.3852	-1.2295
1.1246	0.6690	-4.0677
1.7797	-1.7461	-4.0489
-0.0642	0.7675	4.9006
-2.9210	-1.7907	0.6892
-2.4982	0.8706	4.2117
-1.1769	-1.5683	-3.7414
3.9813	0.9648	-0.6916
-0.2521	0.7698	-4.3782
-0.1691	1.6402	6.0853
0.2984	1.3385	7.1688
-0.7475	2.8257	5.8159
2.4438	1.8154	-1.7160
3.5785	0.1541	-3.1586
3.2652	-1.1157	-2.0085
0.8417	-1.8495	-1.5993

(2*R**,4*S**,5*R**,9*S**,10*R**)-**7M**		
-3.1401	-0.2695	0.5265
-3.4785	-1.4848	-0.3304
-2.3911	-2.5913	-0.2970
-1.0092	-1.9325	-0.6575
-0.6036	-0.6492	0.1480
-1.8010	0.3436	0.1240
0.1739	-2.8960	-0.7479
1.5190	-2.3157	-0.9516
1.7421	-0.9904	-0.9234
0.6408	0.0281	-0.5907
2.2109	3.5858	0.0085
1.5974	2.4241	-0.7528
1.1738	1.2761	0.1787
-0.2341	-0.9702	1.6180
-2.4878	-3.3353	1.0642
-2.7909	-3.6075	-1.4048
3.5219	3.8656	-0.1388
3.1175	-0.5011	-1.2994
1.2449	4.4067	0.8238
0.0667	-4.1211	-0.7434
-4.1669	0.7008	0.3315
-1.3006	-3.9969	1.4576
4.2610	4.9604	0.5174
5.0081	5.7073	-0.0887
4.0886	4.9830	1.8525
-3.1454	-0.5287	1.5890
-4.4508	-1.8886	-0.0159
-3.6362	-1.1477	-1.3653
-1.1324	-1.5944	-1.7013

(2*R**,4*S**,5*R**,9*S**,10*R**)-**7N**		
-2.6300	0.7840	0.8454
-3.2478	0.0347	-0.3297
-2.7069	-1.4100	-0.4967
-1.1362	-1.3434	-0.5496
-0.4191	-0.5486	0.5976
-1.1082	0.8393	0.7353
-0.4321	-2.6783	-0.7881
1.0443	-2.7005	-0.7107
1.7608	-1.6349	-0.3133
1.1083	-0.3306	0.1718
2.5872	2.4952	-0.0695
3.0472	1.3398	0.8018
1.9288	0.3957	1.2856
-0.4860	-1.2866	1.9578
-3.3419	-2.3028	0.6060
-3.2555	-1.9089	-1.8641
2.8636	2.4861	-1.3897
3.2604	-1.7226	-0.4173
1.8301	3.5859	0.6430
-1.0092	-3.7139	-1.1153
-3.1429	2.1146	0.8347
-2.6102	-3.4757	0.9083
2.5024	3.5317	-2.3638
1.9326	3.2866	-3.4119
2.9473	4.7514	-2.0086
-2.9338	0.3361	1.7957
-4.3410	0.0282	-0.2194
-3.0617	0.6118	-1.2473
-0.9182	-0.7804	-1.4739

(2*R**,4*S**,5*R**,9*S**,10*R**)-**7O**		
2.4849	1.0166	-1.5908
2.6890	-0.1658	-2.5259
1.3654	-0.6984	-3.1325
0.3651	-0.9961	-1.9601
0.1838	0.1003	-0.8478
1.5801	0.6298	-0.4224
-1.0159	-1.4900	-2.3827
-1.9761	-1.7968	-1.2962
-1.7441	-1.4376	-0.0205
-0.5137	-0.6137	0.4014
-0.9051	0.7222	4.0827
-0.5370	-0.2247	2.9548
-0.8062	0.3801	1.5665
-0.6802	1.3026	-1.3154
0.8184	0.2693	-4.2116
1.7322	-2.0284	-3.8498
-2.0719	0.5545	4.7376
-2.7060	-1.9178	1.0358
0.0939	1.8160	4.3598
-1.3626	-1.6886	-3.5418
3.7450	1.4089	-1.0497
1.8431	0.6133	-5.1437
-2.5720	1.3702	5.8594
-3.7000	1.8288	5.8890
-1.6884	1.4679	6.8704
2.0825	1.8802	-2.1284
3.4095	0.1044	-3.3084
3.1893	-0.9682	-1.9638
0.8089	-1.8665	-1.4468

H	2.1418	-0.3494	0.2014
H	1.6502	1.3038	0.4274
H	-2.8447	-2.1226	-1.8508
H	0.1477	-1.4192	0.6911
H	0.4227	-0.7069	3.0134
H	-1.2213	-1.2825	2.9396
H	-1.7867	0.7727	1.4600
H	-0.0978	1.1778	1.5363
H	-0.6132	2.0946	-0.4194
H	0.0648	1.9889	-2.0141
H	-1.4667	1.1945	-1.6666
H	1.5680	0.6028	-5.0754
H	1.2897	1.6520	-3.7194
H	0.9346	-1.9861	-4.7131
H	2.6289	-1.5283	-4.7106
H	2.0128	-2.5963	-3.4488
H	-2.9174	-0.2998	4.2796
H	-3.6611	-2.2745	0.3014
H	-2.4019	-2.4819	1.5094
H	-3.2489	-0.9198	1.3393
H	0.4033	2.1062	3.5717
H	-0.2842	2.2757	5.1735
H	0.9453	1.0347	4.8777
H	4.5525	1.4951	-1.4792
H	-0.6489	-0.1992	-4.6461
H	-1.0319	0.6025	6.7053

2.0908	-0.4906	0.2579
1.6432	1.1720	0.5077
-2.8887	-2.0697	-1.9658
0.0533	-1.5066	0.6742
0.2096	-0.9299	2.9789
-1.4932	-1.3142	2.9265
-1.8343	0.7417	1.4053
-0.1331	1.0661	1.5841
-0.5641	2.0532	-0.3730
0.1457	1.9611	-1.9545
-1.4195	1.2111	-1.6591
1.6735	0.6006	-5.0147
1.4183	1.6292	-3.6381
0.9542	-1.9674	-4.7302
2.6626	-1.5679	-4.6751
1.9799	-2.6466	-3.4574
0.9270	0.3361	4.7896
-3.7550	-2.2684	0.1621
-2.5286	-2.5303	1.3933
-3.3424	-0.9476	1.2430
-3.1652	0.0136	4.0687
-2.7138	1.2683	5.2073
-2.7563	1.6359	3.4734
3.9266	1.6946	-0.0507
-0.5725	-0.1160	-4.6533
-0.9709	2.9365	4.8735

-1.9054	0.7699	-0.8838
-1.6184	1.2056	0.7759
2.2960	-3.0382	-1.1807
0.2729	0.3822	-1.5656
0.7237	2.7854	-1.3103
2.2992	2.0649	-1.5105
2.0031	0.9933	0.8394
0.4039	1.6809	0.8408
0.5865	-1.6899	1.6921
0.0766	-0.0738	2.1639
-1.0744	-1.3732	2.1825
-3.2905	-4.0822	1.0398
-2.7532	-2.6599	1.8804
-2.1970	-4.5243	-1.3688
-3.8398	-3.9101	-1.2980
-2.6805	-3.1728	-2.4047
4.1421	3.3006	-0.8293
3.8028	-1.3272	-1.5219
3.0658	0.1163	-2.2009
3.5744	0.0746	-0.4900
0.3313	4.5985	0.2509
0.9726	3.8759	1.7411
1.6481	5.3847	1.1011
-4.0059	1.4325	0.9519
-0.9715	-4.5203	0.6956
3.5399	4.2460	2.1785

-0.8457	1.4688	-0.1265
-0.7337	1.3843	1.6090
1.5026	-3.6242	-1.0496
1.0894	0.3222	-0.7107
3.8296	0.7721	0.2906
3.5459	1.7612	1.6851
2.3728	-0.3490	1.9592
1.2751	0.9930	1.9260
0.0376	-0.7330	2.7439
-1.5066	-1.4057	2.3203
-0.0358	-2.2826	1.9130
-4.3543	-2.6101	0.3173
-3.4667	-1.7629	1.5470
-3.0857	-2.9771	-2.0212
-4.3390	-1.7515	-1.9318
-2.7979	-1.3678	-2.6999
3.3431	1.6299	-1.8559
3.6374	-0.9559	-1.1005
3.7338	-1.6080	0.5617
3.5921	-2.6887	-0.8150
2.4980	4.1209	1.3256
1.3860	4.3155	-0.0392
1.0050	3.1690	1.2271
-2.8102	2.5678	1.6281
-2.3700	-3.9154	0.0646
3.4648	4.7430	-1.1821

2.0960	-0.1307	0.1806
1.4926	1.5007	0.2381
-2.8492	-2.3639	-1.6021
0.2084	-1.3515	0.7828
0.5250	-0.4877	3.0440
-1.0809	-1.1661	3.0765
-1.8375	0.7497	1.5046
-0.1757	1.2686	1.4828
-0.9140	1.9804	-0.4884
-0.1698	1.9249	-2.0499
-1.6352	0.9943	-1.7498
0.4568	1.2053	-3.7948
0.0025	-0.1798	-4.7836
0.8765	-2.4601	-4.3776
2.5264	-1.8764	-4.5900
2.0937	-2.7788	-3.1374
-2.7850	-0.2023	4.4224
-3.5244	-2.5093	0.6095
-2.1893	-2.5637	1.7517
-3.1668	-1.0815	1.5681
0.3559	2.3443	3.4380
-0.2817	2.5731	5.0531
1.0089	1.3894	4.7831
4.3217	1.6370	-1.8005
1.4048	1.0526	-5.8937
-0.8716	0.9588	6.7129

(2*R**,4*S**,5*R**,9*S**,10*R**)-**7P**			
C	-2.6300	0.7322	0.8115
C	-3.1532	-0.0215	-0.4015
C	-2.6066	-1.4698	-0.5155
C	-1.0363	-1.4114	-0.4277
C	-0.3782	-0.5230	0.6926
C	-1.1092	0.8434	0.7559
C	-0.3223	-2.7578	-0.4392
C	1.1577	-2.7313	-0.4177
C	1.8402	-1.6078	-0.1311
C	1.1490	-0.2899	0.2665
C	2.5103	2.5670	-0.1554
C	3.0194	1.4888	0.7855
C	1.9416	0.5315	1.3318
C	-0.4372	-1.1740	2.1008
C	-3.3059	-2.3547	0.5539
C	-3.0217	-1.9744	-1.9252
C	2.7949	2.4913	-1.4717
C	3.3392	-1.6395	-0.2687
C	1.6948	3.6595	0.4874
O	-0.8901	-3.8443	-0.4911
O	-3.1598	2.0553	0.8098
O	-3.3714	-3.7322	0.2178
C	2.3888	3.4521	-2.5132
O	1.8556	3.1076	-3.5527
O	2.7536	4.7164	-2.2305
H	-2.9557	0.2559	1.7410
H	-4.2516	-0.0217	-0.3887
H	-2.8903	0.5507	-1.3033
H	-0.7389	-0.9467	-1.3836

H	-0.8347	1.4524	-0.1169
H	-0.7849	1.4289	1.6240
H	1.6396	-3.6666	-0.6831
H	1.1188	0.3022	-0.6575
H	3.8225	0.9206	0.3073
H	3.5019	1.9852	1.6383
H	2.4181	-0.1540	2.0447
H	1.2652	1.1391	1.9383
H	-1.4515	-1.2230	2.4971
H	-0.0259	-2.1871	2.1173
H	0.1249	-0.5923	2.8384
H	-4.3464	-2.0348	0.6810
H	-2.8435	-2.2801	1.5348
H	-2.6517	-2.9820	-2.1363
H	-4.1132	-1.9946	-2.0260
H	-2.6328	-1.3186	-2.7128
H	3.3196	1.6327	-1.8816
H	3.6667	-0.9018	-1.0071
H	3.8287	-1.4421	0.6890
H	3.7021	-2.6145	-0.6139
H	2.3328	4.2726	1.1318
H	1.2125	4.3178	-0.2398
H	0.8928	3.2388	1.0999
H	-4.1306	1.9881	0.8097
H	-2.4669	-4.0315	-0.0202
H	3.2493	4.7929	-1.3942
